# MRI-Based Genetic Studies Reveal Specific Genetic Variants and Disease Risks Associated With Fat Distribution Across Anatomical Sites

**DOI:** 10.1155/jobe/7792701

**Published:** 2025-08-31

**Authors:** Altayeb Ahmed, Madeleine Cule, Afreen Naz, Marjola Thanaj, Elena P. Sorokin, Chiemela S. Odoemelam, Brandon Whitcher, Naveed Sattar, Jimmy D. Bell, E. Louise Thomas, Hanieh Yaghootkar

**Affiliations:** ^1^School of Natural Sciences, University of Lincoln, Lincoln, UK; ^2^Calico Life Sciences LLC, South San Francisco, California, USA; ^3^Research Centre for Optimal Health, University of Westminster, London, UK; ^4^School of Cardiovascular and Metabolic Health, University of Glasgow, Glasgow, UK; ^5^School of Human Development and Health, Faculty of Medicine, University of Southampton, Southampton, UK

**Keywords:** bone marrow fat, cardiovascular diseases, ectopic fat, liver fat, MRI scan, pancreas fat, subcutaneous fat, Type 2 diabetes, visceral fat

## Abstract

**Objective:** To investigate the genetic determinants of fat distribution across anatomical sites and their implications for health outcomes.

**Methods:** We analyzed neck-to-knee MRI data from the UK Biobank (*n* = 37,589) to measure fat at various locations and used Mendelian randomization to assess effects on 26 obesity-related diseases and 94 biomarkers from FinnGen and other consortia.

**Result:** We identified genetic loci associated with 10 fat depots: abdominal subcutaneous adipose tissue (*n* = 2 loci), thigh subcutaneous adipose tissue (25), thigh intermuscular adipose tissue (15), visceral adipose tissue (7), liver proton density fat fraction (PDFF) (8), pancreas PDFF (11), paraspinal adipose tissue (9), pelvic bone marrow fat (28), thigh bone marrow fat (27), and vertebrae bone marrow fat (5). Genetically higher abdominal subcutaneous adipose tissue was associated with an adverse metabolic profile and higher risks of Type 2 diabetes, and cardiovascular outcomes. Conversely, higher thigh subcutaneous adipose tissue was associated with a favorable profile and lower risks of Type 2 diabetes and cardiovascular outcomes. Higher visceral adipose tissue was associated with gallstones; higher liver PDFF was associated with elevated tyrosine levels, higher Type 2 diabetes risk, and fatty liver disease; pancreas PDFF was associated with thrombotic events; and thigh bone marrow fat was associated with osteoporosis.

**Conclusion:** These results further suggest a unique contribution of fat deposition in different anatomical locations to disease risk, emphasizing the potential, beyond weight loss per se, for future research into depot-specific therapeutic strategies.

## 1. Introduction

Obesity is a major global public health issue, significantly increasing the risk of chronic diseases such as Type 2 diabetes, cardiovascular conditions, and certain cancers. According to recent WHO statistics, approximately 1 in 8 people worldwide is affected by obesity [1]. Traditionally, body mass index (BMI) has been used as a primary metric for assessing obesity; however, it does not account for fat distribution and may not accurately reflect health risks. Subsequently, the waist–hip ratio was introduced as a more refined measure, offering some insight into fat distribution. While waist–hip ratio is a better proxy than BMI, it still falls short of fully capturing the complexities of fat distribution across different anatomical sites [2, 3].

Emerging evidence suggests that the distribution of fat, beyond overall fat mass, plays a crucial role in determining metabolic profiles and disease risk. For example, visceral adipose tissue has been associated with higher risks of insulin resistance, Type 2 diabetes [4], and chronic kidney disease [5, 6]. Conversely, gluteofemoral adipose tissue has been linked to more favorable metabolic and inflammatory profiles and likely offers protective effects against cardiometabolic disorders including Type 2 diabetes [7]. However, while studies often use dual-energy X-ray absorptiometry (DEXA) to quantify fat, this method is less accurate for measuring organ-specific fat, such as liver or pancreas fat, compared to high-resolution MRI. Using MRI scans of liver proton density fat fraction (PDFF), we were able to dissect different mechanisms that lead to higher fat accumulation in the liver, which have different consequences on the risk of cardiovascular disease and cancer [8].

These findings highlight the importance of understanding how specific fat depots impact health outcomes beyond what is measured by BMI and waist–hip ratio. Current literature primarily focuses on the effects of subcutaneous adipose tissue, visceral adipose tissue, and liver fat, leaving a significant gap in our understanding of how fat deposited in other anatomical locations impacts human health. A detailed analysis of fat distribution patterns and their underlying mechanisms is essential for developing targeted therapeutic strategies that address the specific roles of various fat depots.

In this study, we aimed to address these gaps by leveraging high-resolution MRI data from the UK Biobank to precisely measure fat deposition across various anatomical sites. We conducted a Genome-Wide Association Study (GWAS) involving up to 37,358 participants to identify genetic variants associated with 10 distinct fat depots, including abdominal subcutaneous adipose tissue, thigh subcutaneous adipose tissue, thigh intermuscular adipose tissue, visceral adipose tissue, liver PDFF, pancreas PDFF, paraspinal adipose tissue, pelvic bone marrow fat, thigh bone marrow fat, and vertebrae bone marrow fat. We included bone marrow and pancreas PDFF due to their understudied nature and potential involvement in metabolic diseases that are not adequately captured by more common fat depots, such as subcutaneous or visceral fat. Through genetic correlation and Mendelian randomization analyses, we investigated the relationship and the potential causal association of these fat depots with 120 obesity-related diseases and biomarkers, using data from FinnGen (https://www.finngen.fi/en) [9] and other consortia. We employed functional annotation tools to further elucidate the biological mechanisms underlying these associations. Our hypothesis was that there would be distinct associations of different fat depots with different diseases.

## 2. Methods

### 2.1. Image-Derived Measures of Fat Distribution

We used MRI data from the UK Biobank abdominal protocol to measure fat deposited throughout the body. The UK Biobank has approval from the Northwest Multicenter Research Ethics Committee (MREC) to obtain and disseminate data and samples from participants (http://www.ukbiobank.ac.uk/ethics/), and these ethical regulations cover the work in this study. In this study, we included data for up to 37,589 participants of White British ancestry who had MRI scans. We employed neck-to-knee Dixon MRI and single-slice multiecho MRI techniques for abdominal imaging, as outlined in the UK Biobank imaging protocol [9]. Organ, tissue, and bone segmentation was conducted using deep-learning models. Performance metrics for the single-slice multiecho liver, abdominal subcutaneous, and visceral adipose tissues are available in Liu et al. [10], and for the thigh muscle and single-slice multiecho paraspinal muscle, metrics are available in Thanaj et al. 2024 [11]. For pelvic and thigh bones, manual annotations from 110 to 120 participants were utilized, with Dice similarity coefficients of 0.82 and 0.91, respectively, on 20% out-of-sample test data. For the single-slice multiecho vertebrae bone marrow data, manual annotations from 120 participants yielded a Dice similarity coefficient of 0.83 on 20% out-of-sample test data. Quality control was performed by analyzing univariable distributions and visually inspecting scans for anomalies. PDFF was estimated voxelwise in the liver and pancreas [12], providing anatomical coverage for the liver, pancreas, paraspinal muscles, and vertebrae bone marrow. The median statistic was used to summarize the PDFF values for these organs and tissues, producing a single average PDFF value for each.

### 2.2. Genetic Association Studies

We conducted the GWAS using REGENIE Version v3.1.1 [13] on each image-derived phenotype (IDP) of the MRI-measured fat depots. Our GWAS included participants self-identified as “White British,” who clustered with this group in principal component analysis (PCA). We excluded participants with sex chromosome anomalies, sex discrepancies, heterozygosity outliers, and genotype call rate outliers. Covariates included age, squared age, sex, genotyping array, imaging center, and the first 10 genotype-related principal components. IDPs were inverse normal transformed before analysis. Imputed SNPs, filtered by a minor allele frequency (MAF) > 0.01 and an INFO score > 0.9, resulted in 9,788,243 SNPs for the final analysis.

### 2.3. Functional Mapping and Annotation of Genetic Association

Functional mapping, gene set enrichment analysis, and tissue and cell-type annotations were conducted using FUMA v1.5.2 (https://fuma.ctglab.nl/), with default MAGMA settings v1.5.2, to provide functional context for the identified genetic variants within each genomic locus. We prioritized genes using a scoring system based on data from multiple sources: (1) We used the RegulomeDB, which provides functional annotations, especially those in noncoding regions. (2) Combined Annotation Dependent Depletion (CADD) scores were used to predict the functional impact of variants. A score greater than 12.37 indicates a higher likelihood of functional significance, and nearby genes associated with these SNPs were prioritized. (3) We incorporated data from the GTEx v8 project (https://gtexportal.org/) to link genetic variants with gene expression levels (expression quantitative trait loci [eQTLs]) in relevant tissues. (4) Chromatin interaction mapping (ciMap) was used to identify physical interactions between genomic risk loci and gene promoters or enhancers. Genes with strong evidence of such interactions were prioritized, as they suggest potential regulatory relationships. For each of the four criteria, the presence was scored as 1, and the absence as 0. In addition, we integrated protein quantitative trait loci (pQTL) data from the UK Biobank Pharma Proteomics Project (UKB-PPP) (https://metabolomips.org/ukbbpgwas/) [14]. Independent significant SNPs at the genomic risk loci for each IDP were mapped to cis-QTLs to provide deeper functional insight.

### 2.4. Genetic Correlation

We used LDSC [12] to compute the genetic correlation between adiposity IDPs and various anthropometric traits including body fat–free mass, body fat percentage, WHR, BMI, height, and childhood measures of obesity, to understand how our adiposity IDPs relate to these traits. We applied a multiple testing correction, setting the significance level at *p* < 0.006 (0.05/9, the number of anthropometric traits).

### 2.5. Mendelian Randomization

We used Mendelian randomization to assess the causal effect of each individual fat depot on various biomarkers and disease outcomes (Supporting Table 1). For each IDP, we selected the genetic variants as instrumental variables based on the following criteria: *p* value ≤ 5 × 10^−8^, linkage disequilibrium pruning *r*^2^ > 0.001 within a 10 Mb window, using the European ancestry group from the 1000 Genomes reference panel (https://www.internationalgenome.org/). Given the differences in fat distribution between men and women, we first compared the effects of each IDP's genetic instrument across both sexes. We calculated Cochran's Q-statistic to quantify variation in effect sizes between males and females. A Bonferroni correction was applied to determine the significance threshold (*p*=0.05/137 [number of variants] = 4 × 10^−4^).

We used the inverse variance weighted (IVW) method as our primary Mendelian randomization test. However, IVW estimates can be susceptible to biases, including instrumental variable bias, where weak associations between genetic instruments and exposures may distort causal effect estimates, and horizontal pleiotropy, where genetic instruments may influence outcomes through pathways other than the exposure of interest. To address these potential biases, we (1) ensured that each genetic instrument had a robust association with its respective exposure (F-statistics > 10); (2) employed different sensitivity tests, including MR-Egger, weighted median, simple mode, weighted mode methods, and MR-PRESSO to further validate the robustness of our findings [15]. To account for multiple testing, we used Benjamini–Hochberg–adjusted *p* value (BHP) (< 0.05) to correct for and classify significant IVW causal associations.

Given the strong phenotypic correlations among the 10 adiposity IDPs, we used multivariable Mendelian randomization (MVMR) with the IVW method to estimate the effects of each individual fat depot on the outcomes independent of the other nine IDPs. Multivariable MR is an advanced technique that estimates the causal relationships of multiple correlated exposures simultaneously, providing direct estimates of each exposure's effect while accounting for the influence of other exposures in the model. We followed the workflow described by Sanderson et al. [16]. We created exposure data from the 10 IDPs using the mv_extract_exposures_local function from the TwoSampleMR package. We selected the genetic variants as instrumental variables based on the following criteria: *p* value ≤ 5 × 10^−8^, linkage disequilibrium pruning *r*^2^ > 0.001 within a 10 Mb window, using the European ancestry group from the 1000 Genomes reference panel. We used LDSC to estimate the genetic covariance between IDPs. We calculated the conditional F-statistics to assess the strength of the genetic instruments in explaining the exposure after accounting for the correlation and contributions of other exposures in the model. This approach enhances the precision of causal estimates by addressing potential confounding effects from correlated fat depots [17, 18].

## 3. Results

We used high-resolution MRI data from up to 37,589 UK Biobank participants to quantify fat deposition across 10 different anatomical locations including abdominal subcutaneous adipose tissue, thigh subcutaneous adipose tissue, thigh intermuscular adipose tissue, visceral adipose tissue, liver PDFF, pancreas PDFF, paraspinal adipose tissue, pelvic bone marrow fat, thigh bone marrow fat, and vertebrae bone marrow fat. The detailed characteristics of the study sample, including demographic and baseline health metrics, are provided in Supporting Table 2.

There was a strong correlation among several adiposity measures (Supporting Figure 1). For instance, abdominal subcutaneous adipose tissue had a strong correlation with thigh subcutaneous adipose tissue (*rg* = 0.74), thigh intermuscular adipose tissue (0.58), visceral adipose tissue (0.77), liver PDFF (0.60), pancreas PDFF (0.50), and paraspinal adipose tissue (0.61). In contrast, pelvic bone marrow fat, thigh bone marrow fat, and vertebrae bone marrow fat demonstrated strong intercorrelations among themselves but were less correlated with other fat depots (Supporting Figure 1).

Sex-based differences were observed in fat distribution. Women had significantly higher levels of abdominal (*p* value < e−200) and thigh subcutaneous adipose tissue (*p* < e − 200), paraspinal adipose tissue (4.7 × 10^−91^), vertebrae bone marrow fat (8.6 × 10^−99^) but lower levels of pancreas PDFF (*p* < e − 200), liver PDFF (5.2 × 10^−106^), and visceral adipose tissue (*p* < e − 200) compared to men (Supporting Figure 2). Age was strongly associated with variations in adiposity IDPs. Specifically, abdominal and thigh subcutaneous adipose tissue decreased with age, while other fat depots increased with age. Liver PDFF levels increased up to around 60 years of age before decreasing in men, whereas liver PDFF levels plateaued in women beyond this age (Supporting Figure 3).

### 3.1. Genetic Architecture of Fat Depots

We identified 137 genetic loci associated with at least one fat depot: abdominal subcutaneous adipose tissue (*n* = 2 loci), thigh subcutaneous adipose tissue (19), thigh intermuscular adipose tissue (15), visceral adipose tissue (7), liver PDFF (8), pancreas PDFF (11), paraspinal adipose tissue (9), pelvic bone marrow fat (20), thigh bone marrow fat (21), and vertebrae bone marrow fat (5). Some loci were associated with more than one IDP, for example, rs1421085 (in/near *FTO*) was associated with abdominal subcutaneous, thigh subcutaneous, visceral, and paraspinal adipose tissues; rs33823 (*PEPD*) was associated with thigh intermuscular, visceral, and paraspinal adipose tissues and pancreas PDFF; rs17036160 (*PPARG*) was associated with thigh intermuscular adipose tissue, paraspinal adipose tissue, and pelvic and thigh bone marrow fat (Supporting Figures 4A- 4J).

Several of these genomic loci have been associated with various adiposity measures in prior studies. However, their precise roles in influencing fat distribution across different anatomical depots remain poorly understood. In this section, we highlight some interesting findings related to specific fat depots (Supporting Table 3).

Most of the thigh subcutaneous adipose tissue–increasing alleles either had no association or were associated with lower liver PDFF (18/25; *p* value = 0.021; Supporting Figure 4B). This includes Allele G at rs72959041 with the biggest effect on increasing thigh subcutaneous adipose tissue and was associated with both lower expression levels of *RSPO3* (*p*=1.7E − 7) in adipose tissue and its protein levels in the blood (*p*=6E − 89) and lower liver PDFF (*p*=0.039). The other example is rs13389219 (*COBLL1*) where the thigh subcutaneous adipose tissue–increasing allele was associated with lower liver PDFF (*p*=1.1E − 5). The thigh subcutaneous adipose tissue–increasing allele at rs6029180 (*MAFB*) was also associated with lower liver PDFF (0.018).

Among the 15 alleles associated with increased thigh intermuscular adipose tissue, 12 had a negative effect on liver PDFF (*p*=0.018). rs17036160-T within *PPARG* had the biggest effect on thigh intermuscular adipose tissue. This variant is associated with higher *TIMP4* expression in visceral adipose tissue (*p*=2E − 6) and its blood protein levels (*p*=3E − 181). Studies on *TIMP4*-deficient mice fed a high-fat diet demonstrated impaired fat absorption and reduced adipocyte fat accumulation, fibrosis, and steatosis levels [22]. rs77481804 (*MAML2*) and rs151804 (*ARL15*) have not been reported before to be associated with any traits. rs151804 is a cis-pQTL for FST (*p*=1.7E − 14), which is involved in adipogenesis and muscle growth and regeneration [23]. rs33823 was associated with thigh intermuscular adipose tissue, pancreas PDFF, paraspinal adipose tissue, and *PEPD* expression levels in subcutaneous adipose tissue (*p*=2.4E − 24) and PEPD in the blood (*p*=3E − 312).

Variants associated with bone marrow fat were specific to these fat depots and had a weaker association with other adiposity IDPs. Among these variants, rs76428106 had the biggest effect on pelvic bone marrow fat and was associated with FLT3 blood protein levels. The second most significant variant affecting pelvic bone marrow fat was rs10493013, which was also associated with thigh bone marrow fat. This variant is intergenic but resides near another independent variant (rs55938609) in the 5′ UTR of *WNT4*.

The gene set enrichment analysis revealed several enriched pathways for different fat depots (Supporting Tables 4). For thigh subcutaneous fat, key pathways included PI3K-AKT signaling, which is well-established in regulating insulin sensitivity, lipid metabolism, and adipogenesis. This pathway is known to enhance glucose uptake and promote the production of metabolically protective adipokines such as adiponectin, which is highly expressed in subcutaneous adipose tissue [24], and Notch signaling, which regulates fat distribution and inflammation response. Pathways for thigh intermuscular adipose tissue included muscle tumors, adipogenesis, and connective tissue regulation. For liver fat, pathways were related to cell growth, acyl chain remodeling, and estrogen receptor regulation. Pancreas fat pathways involved epigenetic regulation, adipogenesis, and DNA repair. Pelvic bone marrow fat was linked to responses to oxygen levels and chondrocyte differentiation, while pathways for thigh bone marrow fat included fat cell differentiation and regulation.

Given the sex-specific patterns in fat distribution, we conducted sex-stratified GWAS and identified seven loci with sex-specific associations. These included rs1275519 (*SLC5A6*) associated with liver PDFF in male, rs2596827 (*TSEN2*) associated with pancreas PDFF in female, rs1208931 (*LUZP1*) and rs727479 (*CYP19A1*) associated with pelvic bone marrow fat in male, rs10782417 (*SOS2*) associated with pelvic bone marrow fat in female, rs7491585 (*NALCN*) associated with thigh subcutaneous adipose tissue in female, and rs1812736 (*PKMP4*) associated with visceral adipose tissue in male. Full details of these variants are provided in Supporting Table 5.

### 3.2. Genetic Correlation With Anthropometric Measures

We conducted a genetic correlation analysis to explore the relationships between individual fat depots and various anthropometric traits (Supporting Figure 5). Our analysis revealed that abdominal subcutaneous, thigh subcutaneous, thigh intermuscular, and visceral and paraspinal adipose tissues, in addition to liver and pancreas PDFFs, were positively correlated with several measures: whole body fat–free mass, body fat percentage, waist–hip ratio (except for thigh subcutaneous fat in females), childhood obesity, and adult BMI. In contrast, pelvic, thigh, and vertebrae bone marrow fat were positively correlated with adult height. Thigh bone marrow fat was negatively correlated with waist–hip ratio, body fat percentage, and adult BMI. None of the adiposity IDPs were correlated with birth weight.

### 3.3. Causal Associations of Fat Depots With Health Outcomes

Given the differences in fat distribution between men and women, we first compared the effects of each IDP's genetic instrument across both sexes. Our analysis revealed consistent effects for both sexes, indicating the robustness and suitability of the genetic instruments used in our Mendelian randomization analysis in men and women combined (Supporting Table 6; Supporting Figures 6A–6J).

The univariable Mendelian randomization analysis indicated that the genetic instruments were strong for all exposures, as demonstrated by F-statistics > 10 (Supporting Table 7). This supports the robustness of the instruments when estimating the total effect of each exposure on the outcomes. However, when exposures were combined in the multivariable model, the conditional F-statistics substantially decreased below the threshold of 10 for all exposures (Supporting Table 7), indicating weak instrument bias. Given this, the MVMR estimates may be unreliable, and the results should be interpreted with caution. To account for potential weak instrument bias in our MVMR analyses, we applied a robust IVW method based on the framework by Sanderson et al. [16]. This estimator helps mitigate bias in the presence of instrument heterogeneity and collinearity between exposures. However, given the limited conditional F-statistics and overlapping genetic architecture among fat depots, we interpret MVMR results with caution and rely primarily on findings from univariable MR.

**Figure 1:**
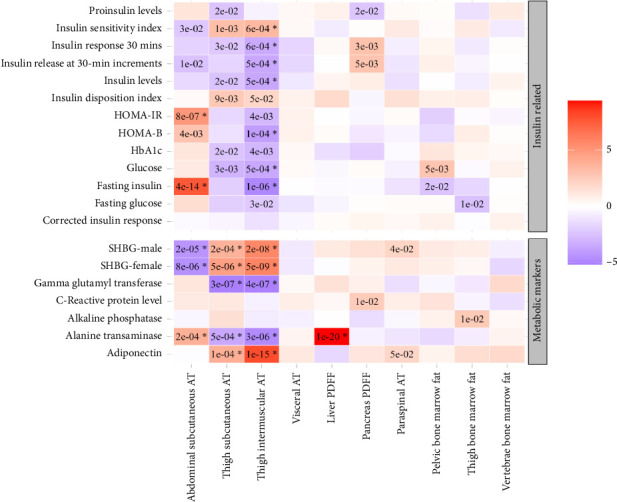
The association between genetically predicted higher IDPs and insulin and metabolic markers from published GWAS. Results are shown from the inverse variance weighted method (IVW) from univariable Mendelian randomization (UV). The color and its intensity correspond to the direction and value of Z-scores (from the IVW test). The asterisk represents Benjamini–Hochberg *p* values < 0.05.

**Figure 2:**
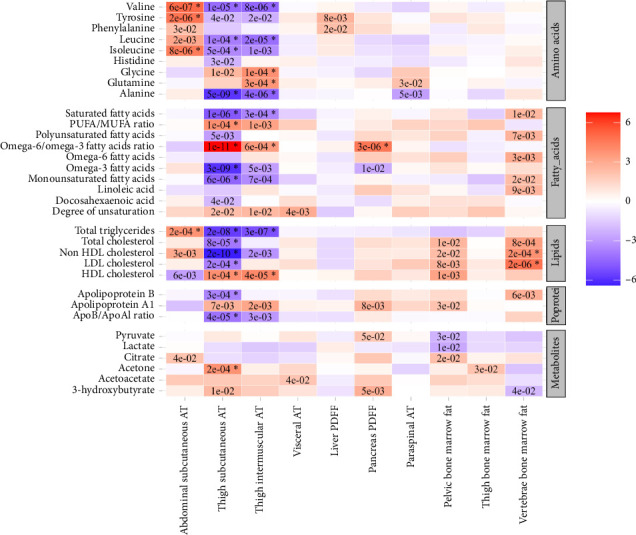
The association between genetically predicted higher IDPs and amino acids, fatty acids, lipids, and metabolites from published GWAS. Results are shown from the inverse variance weighted method (IVW) from univariable Mendelian randomization (UV). The color and its intensity correspond to the direction and value of Z-scores (from the IVW test). The asterisk represents Benjamini–Hochberg *p* values < 0.05.

**Figure 3:**
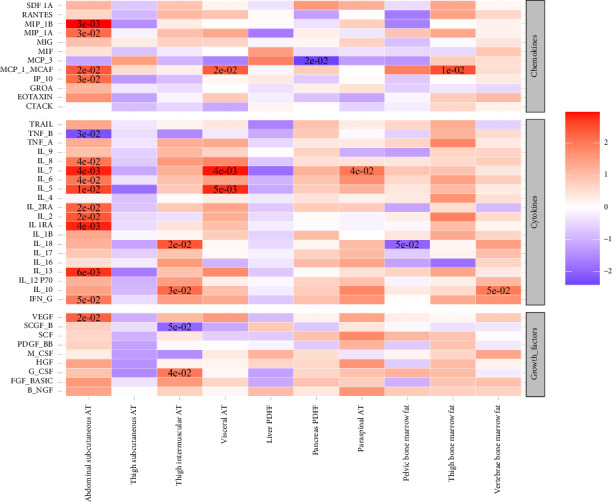
The association between genetically predicted higher IDPs and cytokines, chemokines, and growth factors from published GWAS. Results are shown from the inverse variance weighted method (IVW) from univariable Mendelian randomization (UV). The color and its intensity correspond to the direction and value of Z-scores (from the IVW test). The asterisk represents Benjamini–Hochberg *p* values < 0.05.

The univariable Mendelian randomization analysis highlighted contrasting causal effects of abdominal versus thigh subcutaneous adipose tissues on biomarkers and health outcomes (Figures 1, 2, and 3, Supporting Table 8). Genetically higher abdominal subcutaneous adipose tissue was associated with higher levels of adverse markers, such as increased insulin resistance, higher triglycerides, lower HDL-C, and higher branched-chain amino acids (BCAAs), along with a trend towards higher levels of inflammatory markers. In contrast, genetically higher thigh subcutaneous adipose tissue was associated with higher levels of favorable markers, including higher insulin sensitivity, higher sex hormone–binding globulin (SHBG) and adiponectin, lower BCAA, lower alanine, and higher HDL-cholesterol levels, as well as a trend towards reduced levels of inflammatory markers.

**Figure 4:**
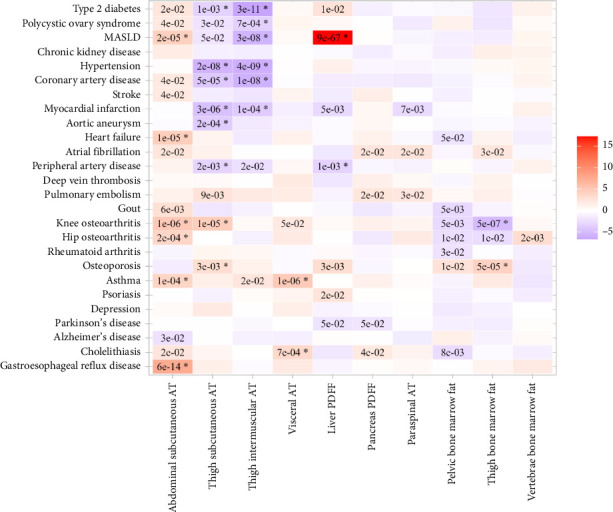
The association between genetically predicted higher IDPs and the risk of 26 disease outcomes, meta-analyzed from FinnGen and published GWAS. Results are shown from the inverse variance weighted method (IVW) from univariable Mendelian randomization (UV). The color and its intensity correspond to the direction and value of Z-scores (from the IVW test). The asterisk represents Benjamini–Hochberg *p* values < 0.05.

These contrasting effects were also evident when assessing disease risk (Figure 4; Supporting Table 9). For instance, genetically higher abdominal subcutaneous adipose tissue was associated with higher risk of Type 2 diabetes (odds ratio [OR] per 1 standard deviation increase: 3.6 [95% confidence interval [CI]: 1.2–11.2]), heart failure (1.6 [1.30–2.0]), and suggested associations (*p* < 0.05 but BHP > 0.05) with higher risk of stroke and atrial fibrillation. In addition, it was associated with a higher risk of knee osteoarthritis (1.9 [1.4–2.4]), hip osteoarthritis (2.1 [1.4–3.1]), asthma (1.3 [1.1–1.5]), and gastroesophageal reflux disease (GERD) (1.1 [0.8–1.5]). The magnitude and direction of effect remained consistent after adjusting for the phenotypic correlation between the IDPs (Figure 4; Supporting Table 10). We were unable to perform sensitivity analyses for abdominal subcutaneous adipose tissue, as the genetic instrument consisted of only two variants. However, the instrument was strong (F-statistic = 55), supporting the reliability of the univariable Mendelian randomization IVW estimate, but pleiotropy could not be assessed.

Genetically higher thigh subcutaneous adipose tissue was associated with lower risk of Type 2 diabetes (0.62 [0.47–0.83]), hypertension (0.92 [0.90–0.95]), coronary artery disease (0.76 [0.67–0.87]), myocardial infarction (0.73 [0.65–0.83]), aortic aneurysm (0.71 [0.59–0.85]), and peripheral artery disease (0.81 [0.71–0.92]). The association with a higher risk of knee osteoarthritis was noted (Figure 4; Supporting Tables 9-10). These results were consistent across all sensitivity analyses (Supporting Table 11).

Genetically higher thigh intermuscular adipose tissue had a similar pattern to thigh subcutaneous adipose tissue, with associations with lower risks for Type 2 diabetes and peripheral artery disease remaining significant after correction for other fat depots and consistent across all sensitivity analyses (Figure 4, Supporting Tables 9-11).

Other fat depots had specific associations with disease outcomes. Genetically higher visceral adipose tissue was associated with a higher risk of cholelithiasis (1.5 [1.2–1.9]; consistent across all sensitivity tests except MR–Egger; Supporting Table 11). Higher liver PDFF was associated with higher levels of alanine transaminase and tyrosine (1.1 [1.1–1.1]), higher risk of Type 2 diabetes (1.2 [1.0–1.4]) and metabolic associated steatotic liver disease (MASLD) (3.8 [3.3–4.4]), and lower risk of myocardial infarction (0.81 [0.70–0.94]) and peripheral artery disease (0.87 [0.79–0.94]), which is mainly driven by the genes blocking triglycerides export from the liver. These results were consistent across all sensitivity tests (Supporting Table 11). Higher pancreas PDFF was associated with a higher risk of deep vein thrombosis (1.01 [0.99–1.02]) and pulmonary embolism (1.8 [1.1–2.9]). None of the associations for paraspinal adipose tissue and pelvic, thigh, and vertebrae bone marrow fat reached our significant levels, but there was a suggestive association between higher pelvic and thigh bone marrow fat with lower risk of knee and hip osteoarthritis and higher risk of osteoporosis, which was diluted in the multivariable analysis (Figure 4; Supporting Tables 9-10).

There was evidence of pleiotropy indicated by Egger intercept *p* value and Cochran's Q for some outcomes including coronary heart disease, peripheral artery disease, heart failure, and aortic aneurysm (Supporting Table 12). To adjust for these pleiotropic effects, we conducted MR-PRESSO, which identified a number of outlier (between 1 and 7) variants. However, there was no significant change in the estimated causal effect for those corrected outcomes (Supporting Table 13).

## 4. Discussion

In this study, we used MRI scans from 37,589 UK Biobank participants to measure fat deposition across 10 distinct anatomical locations. Our genetic analyses identified specific genetic determinants of fat distribution and their implications for health outcomes. These findings reveal how fat accumulation in different anatomical sites may uniquely influence disease risk, offering a more nuanced assessment of metabolic and cardiovascular health. We observed distinct potential effects of abdominal versus thigh subcutaneous adipose tissue on various cardiovascular outcomes, highlighting the potential importance of considering fat distribution in mediating and evaluating disease risk.

Our analysis revealed significant sex-specific patterns in fat distribution, suggesting that sex hormones (or related pubertal-linked development of fat depots) play a critical role in determining fat deposition in men and women. Women had higher levels of abdominal subcutaneous, thigh subcutaneous, and paraspinal adipose tissues and vertebrae bone marrow fat. In contrast, men had elevated levels of visceral adipose tissue and liver and pancreas fat. These observations are consistent with the known effects of sex hormones on fat distribution. Estrogens are associated with increased lipid accumulation in subcutaneous fat depots, contributing to the higher levels observed in women. Conversely, androgens such as testosterone influence fat distribution in men by promoting visceral fat deposition and reducing overall body fat [25]. These hormonal effects may help explain the observed differences in fat distribution between sexes and underscore the need for sex-specific considerations in metabolic and cardiovascular risk assessments; the causal role of sex hormones, including androgens such as testosterone, requires further investigation.

We identified 137 loci associated with at least one fat depot, enhancing our understanding of the genetic underpinnings of fat distribution. These findings offer greater precision compared to previous genetic studies that relied on broader metrics such as BMI and body fat percentage to assess adiposity [19, 21, 26–28]. We characterized several variants that influence multiple fat depots, including those in or near genes such as *FTO, PEPD,* and *PPARG*. This suggests that, despite the distinct phenotypic characteristics of each individual fat depot, there are shared genetic factors that may play a role in shaping overall fat distribution patterns. These common genetic components may help explain the coordination (and thus correlations) of fat deposition across different anatomical regions.

Our findings are consistent with previous research that underscores the risks associated with central adiposity, which has been linked to increased risk of cardiometabolic diseases [20, 29]. However, this study advances our understanding by identifying distinct genetic loci associated with various specific fat depots, offering a more nuanced view of how genetic factors influence fat distribution. Our results highlight the protective role of thigh subcutaneous adipose tissue against adverse health outcomes, including cardiometabolic diseases, which contrasts with the detrimental effects associated with abdominal subcutaneous adipose tissue. This observation is consistent with previous work [30], which demonstrated a differential impact of abdominal versus gluteofemoral fat depots on cardiometabolic health. This paradoxical effect is thought to be related to differences in lipid turnover rates, fat storage capacity, and, potentially, the inflammatory profiles of these fat depots, which appear to be at least in part influenced by specific sets of genes [30].

We extended these findings by identifying distinct genetic loci that contribute to the fat distribution across various anatomical sites. Most of the thigh subcutaneous adipose tissue–increasing alleles either had no association or were associated with lower liver PDFF and higher levels of associated favorable biomarkers, which may clarify their protective roles for cardiometabolic outcomes. The examples include variants in/near *RSPO3*, *COBLL1,* and *MAFB*. Human cellular studies show that *RSPO3* suppresses adipogenesis, increases gluteal adipocyte apoptosis, limiting adipose tissue expansion, and promotes upper-body fat distribution by stimulating abdominal adipose progenitor proliferation [31]. Knocking down *COBLL1* impaired the breakdown of stress fibers in subcutaneous fat cells, which reduced their ability to store fat effectively. This led to disruptions in metabolic processes, including insulin responsiveness and lipid metabolism [32]. *MAFB* deficiency in the hematopoietic system accelerates weight gain, enhances the body fat storage, and increases the adipocyte size [33, 34].

In addition, we found that genetically higher thigh subcutaneous and thigh intermuscular adipose tissues are associated with increased adiponectin secretion. Adiponectin, a plasma hormone produced by adipocytes, is inversely regulated by upper-body fat accumulation. Reduced levels of adiponectin, or hypoadiponectinemia, have been associated with adverse cardiovascular outcomes, diminished insulin sensitivity, and an elevated risk of Type 2 diabetes [35, 36], though whether adiponectin is causally related to outcomes, or rather a downstream marker of insulin action, remains debated [37, 38]. Furthermore, our findings from pathway analysis, associated with these fat depots, highlight several pathways including the PI3K-AKT signaling pathway enriched in the GWAS of thigh subcutaneous fat. PI3K-AKT transmits the effect of some adipokines including adiponectin from fat tissues to metabolic organs.

Our results provide no evidence for an association between genetically higher visceral adipose tissue and the risk of cardiovascular disease. This contrasts with findings from epidemiological studies that suggest a link between increased visceral adipose tissue and a higher risk of cardiovascular disease [39, 40]. The lack of a significant association in our study may be due to several factors. First, while the study had sufficient power to detect associations (F-statistic > 10), the null results may reflect sample-specific limitations or residual confounding by other adiposity-related factors. Visceral adipose tissue is highly correlated with other adiposity measures, such as abdominal subcutaneous fat. Prior observational studies may have been confounded by unaccounted factors such as overall obesity, which could obscure the true effect of visceral adipose tissue on cardiovascular disease. Therefore, while our study does not rule out the possibility of residual confounding or other study-specific limitations influencing these results, further studies with larger and more diverse samples, as well as refined models to address confounding, are needed to clarify the role of visceral adipose tissue in cardiovascular risk.

We found evidence suggesting that higher visceral adipose tissue is associated with an increased risk of cholelithiasis. Visceral obesity may impair gallbladder motility through several mechanisms, one of which is mechanical compression of the gallbladder and liver. The accumulation of visceral fat, particularly in these areas, can lead to physical compression that restricts the normal movement of the gallbladder, impairing its ability to contract effectively. This restricted motility can result in bile stasis, which increases the risk of gallstone formation.

The relationship between elevated liver fat and a reduced risk of myocardial infarction has previously been attributed to diverse and complex mechanisms underlying liver fat accumulation [8]. Variants increasing liver PDFF, such as those in *PNPLA3* and *TM6SF2*, impair hepatic lipid export, promoting fat accumulation without systemic dyslipidemia. This may explain the paradoxical association with lower myocardial infarction and peripheral artery disease, despite increased Type 2 diabetes and MASLD risk.

Our study highlights a strong association between higher liver PDFF levels and higher tyrosine, an essential amino acid. This finding provides insights into the metabolic implications of liver fat accumulation. However, further clinical validation through diagnostic accuracy studies is needed to confirm tyrosine's utility as a reliable biomarker for liver fat.

The association between pancreatic fat and Type 2 diabetes remains debated. Although observational studies have suggested a potential role for pancreatic fat in β-cell dysfunction, our analysis did not reveal any causal associations between pancreatic PDFF and most biomarkers or disease risk factors, including Type 2 diabetes. These findings may reflect confounding by other ectopic fat depots [41] or the limitations of MRI in capturing functionally relevant regional fat [42, 43]. In contrast, we observed associations with thrombotic events. This specific and unexpected association warrants attention and requires further validation and exploration, as pancreatic fat accumulation may contribute to pancreatic inflammation or pancreatitis [44].

Bone marrow fat was an intriguing and underexplored adipose depot that showed unique patterns in our analyses. Bone marrow fat demonstrated a strong correlation among its various anatomical locations, such as pelvic, thigh, and vertebrae bone marrow fat, yet showed a weak or absent correlation with other fat depots, such as subcutaneous and visceral fat. This observation is not due to measurement error but likely reflects the distinct biological nature of bone marrow fat. Bone marrow fat depots, including pelvic, thigh, and vertebrae, showed suggestive associations with osteoarthritis and osteoporosis. Functional annotation identified genes such as *WNT4* and *FLT3. WNT4* functions as a promoter in osteogenic differentiation within bone marrow stromal cells, contributing to bone homeostasis and influencing osteoporotic conditions [45]. This pathway is critical in regulating the balance between bone and fat within the marrow, and lower WNT activity has been associated with increased bone marrow adiposity, particularly in conditions such as osteoporosis [46]. The genetic association observed in our study supports this mechanistic framework and highlights the relevance of WNT signaling in skeletal fat distribution. *FLT3* regulates lymphoid‐primed multipotent progenitors in bone marrow [47]. A recent population-based study, using MRI data [48], has shown that higher bone marrow adiposity is associated with higher cardiometabolic risk, suggesting a systemic role of bone marrow fat in metabolism and inflammation. Unlike other fat depots, bone marrow fat is less influenced by environmental factors such as diet and exercise, which may contribute to its unique properties and functional role. Bone marrow fat's regulation and function are still not fully understood, and ongoing research is focusing on characterizing its biological mechanisms. This includes exploring its role in hematopoiesis and potential implications for metabolic health. While bone marrow fat's specific role in metabolic diseases requires further exploration, the current data suggest that it may have distinct molecular and genetic pathways. Future studies focusing on bone marrow fat will help clarify its contribution to health outcomes and its potential for therapeutic targeting, particularly in the context of metabolic disorders.

While this study provides valuable insights into the genetic underpinnings of different fat depots, there are important limitations to consider. The first key limitation is the lower statistical power of the genetic instrument used for abdominal subcutaneous adipose tissue in our Mendelian randomization analysis. This limitation arises from the genetic variants explaining only a small proportion of the variance in abdominal adipose tissue, potentially leading to a weak instrument. Consequently, this weakness could diminish the observed effect and introduce bias, driving the results toward the null hypothesis. Second, the generalizability of our findings is constrained by the specific characteristics of the genetic instruments and the population studied, which could affect the robustness of the associations identified. Third, we had a substantially weakened instrument even after adjusting for the genetic covariance between IDPs in our multivariable analysis, which could result in attenuated or inflated causal estimates. One possible explanation is the collinearity among the IDPs due to shared genetic determinants and pathways. This collinearity reduces the independent variation explained by each genetic instrument, reducing the conditional F-statistics. Addressing these limitations in future research will be crucial for validating and refining our understanding of the genetic influences on fat distribution and their implications for health outcomes.

## 5. Conclusion

The findings of this study uncover the genetic architecture that governs fat distribution across different anatomical locations and their potential impacts on several health outcomes. These findings further challenge the traditional focus on overall adiposity and support the need for more precision phenotyping in the study of obesity and its multifold associated health risks.

## Data Availability

The data that support the findings of this study are available from UKBiobank. Restrictions apply to the availability of these data, which were used under license for this study. Data are available from https://www.ukbiobank.ac.uk with the permission of UKBiobank. GWAS summary statistics generated in this study have been submitted to the GWAS Catalog (https://www.ebi.ac.uk/gwas) under accession numbers GCT90590312–GCT90590321 and will be made publicly available following curation and embargo release.

## Supporting Information

Supporting table 1: Details of different GWAS used for 129 outcomes. Supporting Table 2: Detailed characteristics of the study population and their baseline health metrics. Supporting Table 3: Annotation and prioritizing of genes associated with IDPs. Supporting Table 4: Gene set analysis results using MAGMA. Supporting Table 5: Genetic loci with distinct sex-specific effects. Supporting Table 6: Summary of the genetic instruments selected for the 10 IDPs. Supporting Table 7: Strength of genetic instruments for the 10 IDPs used in univariable and multivariable Mendelian randomization. Supporting Table 8: The univariable and multivariable Mendelian randomization IVW result. Supporting Table 9: The univariable Mendelian randomization IVW result for the association of 10 IDPs with 26 disease outcomes. Supporting Table 10: The multivariable Mendelian randomization IVW result of the association of 10 IDPs with 26 disease outcomes and sensitivity analysis. Supporting Table 11: Sensitivity results from different Mendelian randomization tests for the effect of IDPs on disease outcomes. Supporting Table 12: Mendelian randomization pleiotropy and heterogeneity test results. Supporting Table 13: MR-PRESSO results for the effect of 10 IDPs on 26 disease outcomes from FinnGen and published GWAS. Supporting Figure 1: The genetic correlation between the 10 IDPs. Supporting Figure 2: The difference in fat volume across the 10 fat depots stratified by sex. Supporting Figure 3: The association between age and adiposity. Supporting Figure 4: The effect of the genetic loci associated with each IDP on other IDPs. Supporting Figure 5: Genetic correlation between IDPs and anthropometric traits. Supporting Figure 6: The effect of genetic variants associated with the IDPs, stratified by sex.

## References

[1] Anderer S. (2024). One in 8 People Worldwide Are Obese. *JAMA*.

[2] Darbandi M., Pasdar Y., Moradi S., Mohamed H. J. J., Hamzeh B., Salimi Y. (2020). Discriminatory Capacity of Anthropometric Indices for Cardiovascular Disease in Adults: A Systematic Review and Meta-Analysis. *Preventing Chronic Disease*.

[3] Abraham A., Yaghootkar H. (2023). Identifying Obesity Subtypes: A Review of Studies Utilising Clinical Biomarkers and Genetic Data. *Diabetic Medicine*.

[4] Feng Y., Yang X., Li Y. (2022). Metabolic Score for Visceral Fat: A Novel Predictor for the Risk of Type 2 Diabetes Mellitus. *British Journal of Nutrition*.

[5] Peng W., Han M., Xu G. (2023). The Association Between Visceral Adiposity Index and Chronic Kidney Disease in the Elderly: A Cross-Sectional Analysis of NHANES 2011–2018. *Preventive Medicine Reports*.

[6] de Mutsert R., Gast K., Widya R. (2018). Associations of Abdominal Subcutaneous and Visceral Fat With Insulin Resistance and Secretion Differ Between Men and Women: The Netherlands Epidemiology of Obesity Study. *Metabolic Syndrome and Related Disorders*.

[7] Lotta L. A., Wittemans L. B. L., Zuber V. (2018). Association of Genetic Variants Related to Gluteofemoral vs Abdominal Fat Distribution With Type 2 Diabetes, Coronary Disease, and Cardiovascular Risk Factors. *JAMA*.

[8] Ahmed A., Cule M., Bell J. D., Sattar N., Yaghootkar H. (2024). Differing Genetic Variants Associated With Liver Fat and Their Contrasting Relationships With Cardiovascular Diseases and Cancer. *Journal of Hepatology*.

[9] Kurki M. I., Karjalainen J., Palta P. (2023). FinnGen Provides Genetic Insights From a Well-Phenotyped Isolated Population. *Nature*.

[10] Liu Y., Basty N., Whitcher B. (2021). Genetic Architecture of 11 Organ Traits Derived from Abdominal MRI Using Deep Learning. *eLife*.

[11] Thanaj M., Basty N., Whitcher B (2024). Precision MRI Phenotyping of Muscle Volume and Quality at a Population Scale. *Frontiers in Physiology*.

[12] Bulik-Sullivan B., Finucane H. K., Anttila V. (2015). An Atlas of Genetic Correlations Across Human Diseases and Traits. *Nature Genetics*.

[13] Mbatchou J., Barnard L., Backman J. (2021). Computationally Efficient Whole-Genome Regression for Quantitative and Binary Traits. *Nature Genetics*.

[14] Sun B. B., Chiou J., Traylor M. (2023). Plasma Proteomic Associations With Genetics and Health in the UK Biobank. *Nature*.

[15] Burgess S., Thompson S. G. (2017). Interpreting Findings From Mendelian Randomization Using the MR-Egger Method. *European Journal of Epidemiology*.

[16] Sanderson E., Spiller W., Bowden J. (2021). Testing and Correcting for Weak and Pleiotropic Instruments in Two-Sample Multivariable Mendelian Randomization. *Statistics in Medicine*.

[17] Burgess S., Thompson S. G. (2015). Multivariable Mendelian Randomization: The Use of Pleiotropic Genetic Variants to Estimate Causal Effects. *American Journal of Epidemiology*.

[18] Sanderson E., Davey Smith G., Windmeijer F., Bowden J. (2019). An Examination of Multivariable Mendelian Randomization in the Single-Sample and Two-Sample Summary Data Settings. *International Journal of Epidemiology*.

[19] Zhang J., Xu L., Li J., Sun L., Qin W. (2020). Association Between Obesity-Related Anthropometric Indices and Multimorbidity Among Older Adults in Shandong, China: A Cross-Sectional Study. *BMJ Open*.

[20] Rønn P. F., Andersen G. S., Lauritzen T. (2020). Abdominal Visceral and Subcutaneous Adipose Tissue and Associations With Cardiometabolic Risk in Inuit, Africans and Europeans: A Cross-Sectional Study. *BMJ Open*.

[21] Li Y., Wang H., Wang K. (2017). Optimal Body Fat Percentage Cut-Off Values for Identifying Cardiovascular Risk Factors in Mongolian and Han Adults: A Population-Based Cross-Sectional Study in Inner Mongolia, China. *BMJ Open*.

[22] Sakamuri S., Watts R., Takawale A. (2017). Absence of Tissue Inhibitor of Metalloproteinase-4 (TIMP4) Ameliorates High Fat Diet-Induced Obesity in Mice Due to Defective Lipid Absorption. *Scientific Reports*.

[23] Zheng H., Qiao C., Tang R. (2017). Follistatin N Terminus Differentially Regulates Muscle Size and Fat In Vivo. *Experimental and Molecular Medicine*.

[24] Huang X., Liu G., Guo J., Su Z. (2018). The PI3K/AKT Pathway in Obesity and Type 2 Diabetes. *International Journal of Biological Sciences*.

[25] Palmer B. F., Clegg D. J. (2015). The Sexual Dimorphism of Obesity. *Molecular and Cellular Endocrinology*.

[26] Locke A. E., Kahali B., Berndt S. I. (2015). Genetic Studies of Body Mass Index Yield New Insights for Obesity Biology. *Nature*.

[27] Di Angelantonio E., Bhupathiraju S. N., Wormser D. (2016). Body-Mass Index and All-Cause Mortality: Individual-Participant-Data Meta-Analysis of 239 Prospective Studies in Four Continents. *The Lancet*.

[28] Lu Y., Day F. R., Gustafsson S. (2016). New Loci for Body Fat Percentage Reveal Link between Adiposity and Cardiometabolic Disease Risk. *Nature Communications*.

[29] Yan Y., Liu J., Zhao X., Cheng H., Huang G., Mi J. (2019). Abdominal Visceral and Subcutaneous Adipose Tissues in Association with Cardiometabolic Risk in Children and Adolescents: the China Child and Adolescent Cardiovascular Health (CCACH) Study. *BMJ Open Diabetes Research & Care*.

[30] Karpe F., Pinnick K. E. (2015). Biology of Upper-Body and Lower-Body Adipose Tissue--Link to Whole-Body Phenotypes. *Nature Reviews Endocrinology*.

[31] Loh N. Y., Minchin J. E. N., Pinnick K. E. (2020). RSPO3 Impacts Body Fat Distribution and Regulates Adipose Cell Biology In Vitro. *Nature Communications*.

[32] Glunk V., Laber S., Sinnott-Armstrong N. (2023). A Non-Coding Variant Linked to Metabolic Obesity With Normal Weight Affects Actin Remodelling in Subcutaneous Adipocytes. *Nature Metabolism*.

[33] Tran M. T., Hamada M., Nakamura M. (2016). MafB Deficiency Accelerates the Development of Obesity in Mice. *FEBS Open Bio*.

[34] Lopez-Jaramillo P. (2016). The Role of Adiponectin in Cardiometabolic Diseases: Effects of Nutritional Interventions. *The Journal of Nutrition*.

[35] Zhao S., Kusminski C. M., Scherer P. E. (2021). Adiponectin, Leptin and Cardiovascular Disorders. *Circulation Research*.

[36] Lee C. H., Lui D. T. W., Cheung C. Y. Y. (2020). Higher Circulating Adiponectin Concentrations Predict Incident Cancer in Type 2 Diabetes the Adiponectin Paradox. *Journal of Clinical Endocrinology and Metabolism*.

[37] Ortega Moreno L., Copetti M., Fontana A. (2016). Evidence of a Causal Relationship Between High Serum Adiponectin Levels and Increased Cardiovascular Mortality Rate in Patients With Type 2 Diabetes. *Cardiovascular Diabetology*.

[38] Chen Z., Bai Y., Long X. (2020). Effects of Adiponectin on T2DM and Glucose Homeostasis: A Mendelian Randomization Study. *Diabetes, Metabolic Syndrome and Obesity: Targets and Therapy*.

[39] Ballin M., Nordström P., Niklasson J., Nordström A. (2021). Associations of Visceral Adipose Tissue and Skeletal Muscle Density With Incident Stroke, Myocardial Infarction, and All-Cause Mortality in Community-Dwelling 70-Year-Old Individuals: A Prospective Cohort Study. *Journal of the American Heart Association*.

[40] Emamat H., Jamshidi A., Farhadi A., Ghalandari H., Ghasemi M., Tangestani H. (2024). The Association between the Visceral to Subcutaneous Abdominal Fat Ratio and the Risk of Cardiovascular Diseases: a Systematic Review. *BMC Public Health*.

[41] Karaevren T. T., Yorulmaz R., Cerit M. (2025). Fatty Pancreas Disease in Newly Diagnosed Type 2 Diabetes Patients: a Case-Control Study on Relationships with Glycemic Control and Exocrine Function. *Diabetology & Metabolic Syndrome*.

[42] Skudder-Hill L., Sequeira I. R., Cho J., Ko J., Poppitt S. D., Petrov M. S. (2022). Fat Distribution Within the Pancreas According to Diabetes Status and Insulin Traits. *Diabetes*.

[43] Martin S., Sorokin E. P., Thomas E. L. (2022). Estimating the Effect of Liver and Pancreas Volume and Fat Content on Risk of Diabetes: A Mendelian Randomization Study. *Diabetes Care*.

[44] Caldart F., de Pretis N., Luchini C., Ciccocioppo R., Frulloni L. (2023). Pancreatic Steatosis and Metabolic Pancreatic Disease: a New Entity?. *Internal and Emergency Medicine*.

[45] Zhang Q., Pan Y., Ji J., Xu Y., Zhang Q., Qin L. (2021). Roles and Action Mechanisms of WNT4 in Cell Differentiation and Human Diseases: A Review. *Cell Death Discovery*.

[46] Song L., Liu M., Ono N., Bringhurst F. R., Kronenberg H. M., Guo J. (2012). Loss of Wnt/β-Catenin Signaling Causes Cell Fate Shift of Preosteoblasts From Osteoblasts to Adipocytes. *Journal of Bone and Mineral Research*.

[47] Zriwil A., Böiers C., Kristiansen T. A. (2018). Direct Role of FLT3 in Regulation of Early Lymphoid Progenitors. *British Journal of Haematology*.

[48] Grune E., Nattenmüller J., Kiefer L. S. (2025). Subphenotypes of Body Composition and Their Association With Cardiometabolic Risk-Magnetic Resonance Imaging in a Population-Based Sample. *Metabolism*.

