# Supplementary material for: MRI-Based Genetic Studies Reveal Specific Genetic Variants and Disease Risks Associated With Fat Distribution Across Anatomical Sites
Source: J Obes. 2025 Aug 31;2025:7792701. doi: 10.1155/jobe/7792701 (PMC12414625; doi:10.1155/jobe/7792701)
Supplement: Supporting Information — Additional supporting information can be found online in the Supporting Information section. [file 7792701.f1.docx]

**MRI-Based genetic studies reveal specific genetic variants and disease risks associated with fat distribution across anatomical sites**

Altayeb Ahmed, School of Natural Science, College of Health and Science, University of Lincoln, Joseph Banks Laboratories, Green Lane, Lincoln, UK., Email: [28404091@students.lincoln.ac.uk](mailto:28404091@students.lincoln.ac.uk)

Madeleine Cule, Calico Life Sciences LLC, South San Francisco, CA, United States., Email: [cule@calicolabs.com](mailto:cule@calicolabs.com)

Afreen Naz, School of Natural Science, College of Health and Science, University of Lincoln, Joseph Banks Laboratories, Green Lane, Lincoln, UK., Email: [ANaz@lincoln.ac.uk](mailto:ANaz@lincoln.ac.uk)

Marjola Thanaj, Research Centre for Optimal Health, School of Life Sciences, University of Westminster, London, UK., Email: [thanajm@my.westminster.ac.uk](mailto:thanajm@my.westminster.ac.uk)

Elena P Sorokin, Calico Life Sciences LLC, South San Francisco, CA, United States., Email: [sorokin@calicolabs.com](mailto:sorokin@calicolabs.com)

Chiemela S. Odoemelam, School of Natural Science, College of Health and Science, University of Lincoln, Joseph Banks Laboratories, Green Lane, Lincoln, UK., Email: [COdoemelam@lincoln.ac.uk](mailto:COdoemelam@lincoln.ac.uk)

Brandon Whitcher, Research Centre for Optimal Health, School of Life Sciences, University of Westminster, London, UK., Email: [b.whitcher@westminster.ac.uk](mailto:b.whitcher@westminster.ac.uk)

Naveed Sattar, School of Cardiovascular and Metabolic Health, University of Glasgow, Glasgow, U.K., Email: [Naveed.Sattar@glasgow.ac.uk](mailto:Naveed.Sattar@glasgow.ac.uk)

Jimmy D Bell, Research Centre for Optimal Health, School of Life Sciences, University of Westminster, London, UK., Email: [J.Bell@westminster.ac.uk](mailto:J.Bell@westminster.ac.uk)

E Louise Thomas, Research Centre for Optimal Health, School of Life Sciences, University of Westminster, London, UK., Email: [l.thomas3@westminster.ac.uk](mailto:l.thomas3@westminster.ac.uk)

Hanieh Yaghootkar, School of Natural Science, College of Health and Science, University of Lincoln, Joseph Banks Laboratories, Green Lane, Lincoln, UK., Email: [HYaghootkar@lincoln.ac.uk](mailto:HYaghootkar@lincoln.ac.uk)

**Corresponding author:**

Hanieh Yaghootkar, School of Natural Science, College of Health and Science, University of Lincoln, Joseph Banks Laboratories, Green Lane, Lincoln, UK., Email: [HYaghootkar@lincoln.ac.uk](mailto:HYaghootkar@lincoln.ac.uk)

**Supplementary table. 1. Details of different GWAS used for 129 outcomes.** Disease outcome data is from FinnGen and published GWAS in European population including both males and females. Data for biomarkers is from published GWAS only. Phenocode: phenotype identifier, Sample size: number of participants for continuous traits; Case: number of cases, Control: number of controls, author: the author of the study for published GWAS and year of publication; Data Freeze (DF) version of FinnGen data release.

| **Trait/disease** | **Phenocode/Pubgwas** | **Phenocode/FinnGen** | **Sample size (Case; control (Pubgwas)** | **Case; control (FinnGen)** | **Author/Pubgwas** | **Data Freeze/**  **FinnGen** | **PMID/**  **Pubgwas** |
| --- | --- | --- | --- | --- | --- | --- | --- |
| Type 2 diabetes | NA | T2D | 80154; 853816; | 65085;335112 | Mahajan A 2022 | DF10 | 35551307 |
| Polycystic ovary syndrome | ebi-a-GCST90044902 | E4_PCOS | 797;140558 | 2544;408430 | Tyrmi JS | DF10 | 34791234 |
| MASLD | ebi-a-GCST90091033 | NAFLD | 8434;770180 | 2568;409613 | Ghodsian N(2021) | DF10 | 34841290 |
| Chronic kidney disease | Wuttke, 2019 | N14_CHRONKIDNEYDIS | 41395;439303 | 10039;396706 | Wuttke (2019) | DF10 | 31152163 |
| Hypertension | ebi-a-GCST90038604 | I9_HYPTENS | 129909;354689 | 122996;289117 | Dönertaş et al. (2021) | DF10 | 33959723 |
| Coronary heart disease | ebi-a-GCST003116 | I9_ATHSCLE | 22233;64762 | 16243;381977 | Nickpay 2015 | DF10 | 26343387 |
| Stroke | ebi-a-GCST006908 | I9_STR | 34217;406111 | 27497;371723 | Malik R. 2018 | DF10 | 26343387 |
| Myocardial infarction | ebi-a-GCST011364 | I9_MI_STRICT | 14825;2680 | 26060;343079 | Hartiala JA et al. 2021 | DF10 | 33532862 |
| Aortic aneurysm | ebi-a-GCST90018783 | I9_AORTANEUR | 3230;475964 | 8125;381977 | Sakaue S (2021) | DF10 | 34594039 |
| Heart failure | ebi-a-GCST009541 | I9_HEARTFAIL | 47309;930014 | 29672;382509 | Shah et al. 2020 | DF10 | 31919418 |
| Atrial fibrillation | ebi-a-GCST006414 | I9_AF | 60620;970216 | 50743;210652 | Nielsen JB et al. 2018 | DF10 | 30061737 |
| Peripheral artery disease | ebi-a-GCST90018890 | I9_PAD | 7114;475964 | 11924;288638 | Sakaue S (2021) | DF7 | 34594039 |
| Deep vein thrombosis | ebi-a-GCST90038615 | I9_PHLETHROMBDVTLOW | 9529;475069 | 6501;357111 | Dönertaş et al. (2021) | DF10 | 33959723 |
| Pulmonary embolism | ebi-a-GCST90013937 | I9_PULMEMB | 407;746 | 10046;401128 | Mbatchou J (2021) | DF10 | 34017140 |
| Gout | ieu-a-1054 | M13_GOUT | 2115;69374 | 9568;262844 | Kottgen (2013) | DF10 | 23263486 |
| Knee osteoarthritis | ebi-a-GCST007090 | M13_ARTHROSIS_KNEE | 24955;378169 | 48836;262844 | Tachmazidou (2019) | DF10 | 30664745 |
| Hip osteoarthritis | ebi-a-GCST007091 | M13_ARTHROSIS_COX | 15704;378169 | 80598;262844 | Tachmazidou (2019) | DF10 | 30664745 |
| Rheumatoid arthritis | ebi-a-GCST90013534 | M13_RHEUMA | 14361;43923 | 13621;262844 | Ha E (2020) | DF10 | 33310728 |
| Osteoporosis | ebi-a-GCST90038656 | M13_OSTEOPOROSIS | 7751;476847 | 8017;391037 | Dönertaş et al. (2021) | DF10 | 33959723 |
| Asthma | ebi-a-GCST90014325 | J10_ASTHMA_MAIN_EXMORE | 56167;352255 | 37760;219734 | Valette K (2021) | DF10 | 34103634 |
| Psoriasis | ebi-a-GCST90019017 | L12_PSORIASIS | 15967; 28169 | 10312;397564 | Stuart PE (2021) | DF10 | 34927100 |
| Depression | ebi-a-GCST90018833 | F5_DEPRESSIO | 13559;435855 | 47696;359290 | Sakaue S (2021) | DF10 | 34594039 |
| Parkinson's disease | ieu-b-7 | G6_PARKINSON | 33674;449056 | 4681;407500 | Nalls MA (2019) | DF10 | 31701892 |
| Alzheimer's disease | ebi-a-GCST90027158 | G6_ALZHEIMER | 39106;46828 | 10520;401661 | Bellenguez C (2022) | DF10 | 35379992 |
| Cholelithiasis | ebi-a-GCST90018819 | K11_CHOLELITH | 26122;461431 | 40191;361641 | Sakaue S (2021) | DF10 | 34594039 |
| Gastroesophageal reflux disease | ebi-a-GCST90000514 | K11_REFLUX | 129080;473524 | 28859;350064 | Ong Js (2021) | DF10 | 34187846 |
| Cytokines and growth factors | NA | NA | 8293 | NA | Ahola-Olli AV (2017); Kalaoja (2021) | NA | 27989323;33491305 |
| Metabolites | metd-* | NA | 115078 | NA | Borges CM (2022) | NA | 35692035 |
| Childhood obesity | NA | NA | 24160 | NA | Bradfield JP (2019) | NA | 31504550 |
| Childhood BMI | ebi-a-GCST90002409 | NA | 39620 | NA | Vogelezang S (2020) | NA | 33045005 |
| HbA1c | ebi-a-GCST90002244 | NA | 146806 | NA | Chen J (2021) | NA | 34059833 |
| Adiponectin | ieu-a-1 | NA | 29347 |  | Dastani Z (2012) | NA | 22479202 |
| HOMA-B, HOMA-IR | ieu-b-117/118 | NA | 46186 |  | Dupius J (2010) | NA | 20081858 |
| Lipids |  | NA | 1320000 |  | Graham SE (2021); Kanoni S (2022); Ramdas S(2022) | NA | 34887591;36575460;35931049 |
| Leptin | ieu-a-1002 | NA | 32161 |  | Kilpel€ainen TO (2016) | NA | 26833098 |
| Fasting glucose, fasting insulin |  | NA | 140595; 98210 |  | Lagou V (2021) | NA | 33558525 |
| Liver enzymes (ALP, ALT, GGT) |  | NA | 437438;437267;437194 |  | Pazoki R (2021) | NA | 33972514 |
| Disposition index; corrected insulin response; insulin at 30 min; incremental insulin at 30 min |  | NA | 5318 |  | Prokopenko I (2014) | NA | 24699409 |
| Adult BMI; waist-to-hip ratio (female); waist-to-hip ratio (male) |  | NA | 806834; 379501; 315284 |  | Pulit SL (2018) | NA | 30239722 |
| Fasting proinsulin |  | NA | 45861 |  | Kelsy A. Broadway (2023) | NA | 36693378 |
| Insulin sensitivity index |  | NA | 55,535 |  | Williamson (2023) | NA | 37291194 |
| Birth weight |  | NA | 298142 |  | Warrington NM (2019) | NA | 31043758 |
| Adult height |  | NA | 4080687 |  | Yengo L (2022) | NA | 36224396 |
| BFP |  | NA | 454633 |  | Elsworth B (2018) | NA | NA |
| C-reactive protein |  | NA | 204402 |  | Ligthart S (2018) | NA | 30388399 |
| Whole-body fat-free mass |  | NA | 454850 |  | Elsworth B (2018) | NA | NA |

**Supplementary table. 2. Detailed characteristics of the study population and their baseline health metrics.**

|  | **Male** | **Female** | **Combined** |
| --- | --- | --- | --- |
| No of Participants | 18320 | 19269 | 37589 |
| Age (Years) Mean ± SD | 65.37±7.72 | 63.98±7.46 | 64.66±7.62 |
| BMI (Mean ± SD) | 26.82±3.73 | 26.02±4.63 | 26.41±4.23 |
| Abdominal subcutaneous AT (Mean ± SD) L | 6.94 ± 3.14 | 9.84 ± 4.30 | 8.42±4.05 |
| Thigh slabs subcutaneous AT (Mean ± SD) L | 1.24 ± 0.45 | 2.06 ± 0.76 | 1.66±0.75 |
| Visceral AT (Mean ± SD) L | 5.14 ± 2.31 | 2.83 ± 1.55 | 3.96±2.27 |
| Liver PDFF (Mean ± SD) % | 5.48 ± 5.00 | 4.18 ± 4.49 | 4.82±4.79 |
| Paraspinal PDFF (Mean ± SD) % | 7.01 ± 3.72 | 8.03 ± 4.17 | 7.53±3.99 |
| Pancreas PDFF (Mean ± SD) % | 12.92 ± 8.76 | 8.53 ± 6.85 | 10.67±8.15 |
| Thigh intermuscular AT (Mean ± SD) | 0.02 ± 0.02 | 0.02 ± 0.02 | 0.02±0.02 |
| Pelvic bone marrow fat (Mean ± SD) % | 69.44 ± 6.08 | 68.27 ± 5.92 | 68.84 ± 6.03 |
| Thigh bone marrow fat (Mean ± SD) % | 88.85 ± 1.83 | 88.14 ± 1.95 | 88.49 ± 1.93 |
| Vertebrae bone marrow fat (Mean ± SD) % | 37.41 ± 8.76 | 39.92 ± 8.96 | 38.68 ± 8.95 |

**Supplementary table. 3. Annotation and prioritising of genes associated with IDPs.** CADD: Combined Annotation Dependent Depletion score, indicating the deleteriousness of the variant (>12.37 indicate high probability of deleteriousness); RDB: RegulomeDB score, lower scores indicate stronger evidence of regulatory function; FUNC: functional annotation of the variant; ciMap: evidence of chromatin interactions.

| **IDP** | **SNP** | **CADD** | **RDB** | **FUNC** | **eQTL Gene** | **Cis eQTL tissue (p-value)** | **Cis pQTL (p-value)** | **ciMap** |
| --- | --- | --- | --- | --- | --- | --- | --- | --- |
| Abdominal subcutaneous adipose tissue | rs1421085 | 21.4 | 5 | intronic | *FTO* | GTEx/v8/Muscle Skeletal (p= 1.3E-22) | NA | Yes |
| Thigh intermuscular adipose tissue | rs33823 | 16.4 | NA | intergenic | *PEPD* | GTEx/v8/Adipose Subcutaneous (2.4E-24); Adipose Visceral Omentum (2E-10) | PEPD_P12955_OID30688_v1_Inflammation_II (3E-312) | Yes |
| Thigh intermuscular adipose tissue | rs17036160 | 16.2 | 3a | intronic | *TIMP4* | Adipose Visceral Omentum (2E-6) | TIMP4_Q99727_OID21147_v1_Neurology(3E-181) | No |
| Thigh intermuscular adipose tissue | rs77481804 | 16.1 | 6 | intronic | *MAML2* | NA | NA | Yes |
| Thigh intermuscular adipose tissue | rs151804 | 18.8 | 7 | intronic | *ARL15* | NA | FST_P19883_OID20770_v1_Inflammation (1.7E-14) | No |
| Thigh subcutaneous adipose tissue | rs72959041 | 15.6 | 6 | intronic | *RSPO3* | GTEx/v8/Adipose Subcutaneous (1.7E-7) | RSPO3_Q9BXY4_OID21415_v1_Oncology (6E-89) | No |
| Thigh subcutaneous adipose tissue | rs13389219 | 18.1 | 7 | intergenic | *COBLL1* | GTEx/v8/Adipose Visceral Omentum (1.5E-16); Adipose subcutaneous (2.7E-13) | NA | No |
| Thigh subcutaneous adipose tissue | rs6029180 | 19.9 | 3a | intergenic | *MAFB* | NA | NA | Yes |
| Pelvic bone marrow fat | rs76428106 | 11.7 | 5 | intronic | *FLT3* | NA | FLT3_P36888_OID21272_v1_Oncology(1E-19) | No |

**Supplementary table 4. Gene Set Analysis Results using MAGMA.** NGENES: the total number of genes in the set; BETA: the effect size of the gene set association with the trait; BETA_STD: the standard deviation of the effect; SE: the standard error of BETA; P: the p-value for the association of the gene set with the trait.

|  | **nGENES** | **BETA** | **STD** | **SE** | **P** | **FULL_NAME** |
| --- | --- | --- | --- | --- | --- | --- |
| **Abdominal Subcutaneous adipose tissue** | | | | | |  |
| 54 | 0.443 | 0.024 | 0.119 | 9.89E-05 | KEGG_ACUTE_MYELOID_LEUKEMIA |  |
| 130 | 0.256 | 0.021 | 0.07 | 1.18E-04 | NOUZOVA_TRETINOIN_AND_H4_ACETYLATION |  |
| 8 | 1.062 | 0.022 | 0.295 | 1.63E-04 | GOBP_NEGATIVE_REGULATION_OF_GRANULOCYTE_DIFFERENTIATION |  |
| 318 | 0.172 | 0.022 | 0.048 | 1.66E-04 | GRESHOCK_CANCER_COPY_NUMBER_UP |  |
| 451 | 0.15 | 0.023 | 0.042 | 1.66E-04 | DACOSTA_UV_RESPONSE_VIA_ERCC3_COMMON_DN |  |
| 57 | 0.388 | 0.021 | 0.109 | 1.82E-04 | REACTOME_SIGNALING_BY_ERBB4 |  |
| 16 | 0.725 | 0.021 | 0.204 | 1.85E-04 | BIOCARTA_CDMAC_PATHWAY |  |
| 15 | 0.763 | 0.022 | 0.215 | 1.92E-04 | GOBP_REGULATION_OF_GRANULOCYTE_DIFFERENTIATION |  |
| 6 | 1.11 | 0.02 | 0.321 | 2.75E-04 | GOMF_ADP_RIBOSE_DIPHOSPHATASE_ACTIVITY |  |
| **Thigh subcutaneous adipose tissue** | | | | | |  |
| 21 | 0.746 | 0.025 | 0.178 | 1.45E-05 | REACTOME_FORMATION_OF_PARAXIAL_MESODERM |  |
| 5 | 1.656 | 0.027 | 0.42 | 4.00E-05 | GOBP_POSITIVE_REGULATION_OF_MAST_CELL_CHEMOTAXIS |  |
| 7 | 1.154 | 0.022 | 0.292 | 4.01E-05 | GOBP_POSITIVE_REGULATION_OF_TRANSCRIPTION_OF_NOTCH_RECEPTOR_TARGET |  |
| 19 | 0.823 | 0.026 | 0.214 | 5.93E-05 | WP_HYPERTROPHY_MODEL |  |
| 326 | 0.188 | 0.024 | 0.049 | 6.06E-05 | WP_PI3KAKT_SIGNALING_PATHWAY |  |
| 24 | 0.684 | 0.024 | 0.182 | 8.55E-05 | REACTOME_NOTCH_HLH_TRANSCRIPTION_PATHWAY |  |
| 212 | 0.221 | 0.023 | 0.06 | 1.11E-04 | GOBP_RESPONSE_TO_CARBOHYDRATE |  |
| 560 | 0.138 | 0.023 | 0.038 | 1.61E-04 | GOBP_CELLULAR_RESPONSE_TO_LIPID |  |
| 24 | 0.652 | 0.023 | 0.182 | 1.68E-04 | REACTOME_NOTCH3_INTRACELLULAR_DOMAIN_REGULATES_TRANSCRIPTION |  |
| **Thigh intermuscular adipose tissue** | | | | | |  |
| 95 | 0.445 | 0.031 | 0.089 | 3.26E-07 | REN_ALVEOLAR_RHABDOMYOSARCOMA_UP |  |
| 13 | 1.275 | 0.033 | 0.294 | 7.28E-06 | WP_TRANSCRIPTIONAL_CASCADE_REGULATING_ADIPOGENESIS |  |
| 31 | 0.603 | 0.024 | 0.142 | 1.04E-05 | WP_INITIATION_OF_TRANSCRIPTION_AND_TRANSLATION_ELONGATION_AT_THE_HIV1_LTR |  |
| 5 | 1.63 | 0.027 | 0.387 | 1.27E-05 | GOBP_REGULATION_OF_CONNECTIVE_TISSUE_REPLACEMENT |  |
| 54 | 0.496 | 0.026 | 0.118 | 1.39E-05 | REACTOME_SIGNALING_BY_NOTCH1_PEST_DOMAIN_MUTANTS_IN_CANCER |  |
| 10 | 1.167 | 0.027 | 0.31 | 8.22E-05 | GOBP_POLYOL_TRANSMEMBRANE_TRANSPORT |  |
| 107 | 0.306 | 0.023 | 0.081 | 8.34E-05 | GOMF_TRANSCRIPTION_COREGULATOR_BINDING |  |
| 215 | 0.215 | 0.023 | 0.06 | 1.64E-04 | GOBP_OSTEOBLAST_DIFFERENTIATION |  |
| 7 | 1.036 | 0.02 | 0.289 | 1.74E-04 | GOBP_POSITIVE_REGULATION_OF_TRANSCRIPTION_OF_NOTCH_RECEPTOR_TARGET |  |
| **Visceral adipose tissue** | | | | | |  |
| 6 | 1.332 | 0.024 | 0.317 | 1.33E-05 | GOBP_AXONEMAL_CENTRAL_APPARATUS_ASSEMBLY |  |
| 68 | 0.405 | 0.024 | 0.105 | 5.29E-05 | GOBP_POSITIVE_REGULATION_OF_AXONOGENESIS |  |
| 235 | 0.214 | 0.024 | 0.056 | 7.47E-05 | GOBP_ANATOMICAL_STRUCTURE_MATURATION |  |
| 89 | 0.314 | 0.022 | 0.087 | 1.54E-04 | KAYO_CALORIE_RESTRICTION_MUSCLE_UP |  |
| 116 | 0.292 | 0.023 | 0.081 | 1.65E-04 | GOBP_POSITIVE_REGULATION_OF_TRANSPORTER_ACTIVITY |  |
| 161 | 0.243 | 0.022 | 0.068 | 1.90E-04 | GOBP_POSITIVE_REGULATION_OF_MONOATOMIC_ION_TRANSMEMBRANE_TRANSPORT |  |
| 39 | 0.474 | 0.022 | 0.135 | 2.26E-04 | GOBP_ESTABLISHMENT_OF_MITOTIC_SPINDLE_LOCALIZATION |  |
| 862 | 0.102 | 0.021 | 0.029 | 2.34E-04 | GOBP_NEGATIVE_REGULATION_OF_TRANSCRIPTION_BY_RNA_POLYMERASE_II |  |
| 42 | 0.435 | 0.021 | 0.132 | 4.94E-04 | PAPASPYRIDONOS_UNSTABLE_ATEROSCLEROTIC_PLAQUE_DN |  |
| **Liver PDFF** | | | | | |  |
| 95 | 0.352 | 0.025 | 0.087 | 2.65E-05 | GOBP_REGULATION_OF_EXTENT_OF_CELL_GROWTH |  |
| 5 | 1.428 | 0.023 | 0.367 | 5.08E-05 | REACTOME_ACYL_CHAIN_REMODELING_OF_DAG_AND_TAG |  |
| 6 | 1.442 | 0.026 | 0.382 | 8.11E-05 | REACTOME_RUNX1_REGULATES_ESTROGEN_RECEPTOR_MEDIATED_TRANSCRIPTION |  |
| 163 | 0.246 | 0.023 | 0.067 | 1.09E-04 | GOBP_REGULATION_OF_CELL_SIZE |  |
| 287 | 0.192 | 0.024 | 0.052 | 1.20E-04 | GOBP_REGULATION_OF_DEVELOPMENTAL_GROWTH |  |
| 5 | 1.479 | 0.024 | 0.409 | 1.49E-04 | GOMF_DIACYLGLYCEROL_O_ACYLTRANSFERASE_ACTIVITY |  |
| 155 | 0.413 | 0.037 | 0.115 | 1.66E-04 | NIKOLSKY_BREAST_CANCER_11Q12_Q14_AMPLICON |  |
| 9 | 1.083 | 0.024 | 0.304 | 1.81E-04 | GOBP_DIACYLGLYCEROL_BIOSYNTHETIC_PROCESS |  |
| 6 | 1.495 | 0.027 | 0.421 | 1.95E-04 | GOCC_BASEMENT_MEMBRANE_COLLAGEN_TRIMER |  |
| **Pancreas PDFF** | | | | | |  |
| 2 | 3.292 | 0.034 | 0.679 | 6.37E-07 | GOMF_HISTONE_H3K27ME2_H3K27ME3_DEMETHYLASE_ACTIVITY |  |
| 50 | 0.594 | 0.031 | 0.123 | 6.74E-07 | WU_SILENCED_BY_METHYLATION_IN_BLADDER_CANCER |  |
| 13 | 1.377 | 0.036 | 0.291 | 1.09E-06 | WP_TRANSCRIPTIONAL_CASCADE_REGULATING_ADIPOGENESIS |  |
| 5 | 1.28 | 0.021 | 0.283 | 3.06E-06 | GOBP_DNA_LIGATION_INVOLVED_IN_DNA_REPAIR |  |
| 25 | 0.731 | 0.027 | 0.181 | 2.81E-05 | WP_DIFFERENTIATION_OF_WHITE_AND_BROWN_ADIPOCYTE |  |
| 27 | 0.673 | 0.025 | 0.169 | 3.46E-05 | WANG_CLASSIC_ADIPOGENIC_TARGETS_OF_PPARG |  |
| 14 | 1.038 | 0.028 | 0.262 | 3.77E-05 | GOBP_VENTRICULAR_TRABECULA_MYOCARDIUM_MORPHOGENESIS |  |
| 37 | 0.491 | 0.022 | 0.128 | 6.42E-05 | RASHI_RESPONSE_TO_IONIZING_RADIATION_1 |  |
| 12 | 0.958 | 0.024 | 0.254 | 7.99E-05 | REACTOME_TRIGLYCERIDE_BIOSYNTHESIS |  |
| **Paraspinal adipose tissue** | | | | | |  |
| 10 | 1.067 | 0.025 | 0.278 | 6.27E-05 | GOBP_NEGATIVE_REGULATION_OF_PATHWAY_RESTRICTED_SMAD_PROTEIN_PHOSPHORYLATION |  |
|  | 13 | 1.049 | 0.028 | 0.285 | 1.20E-04 | WP_TRANSCRIPTIONAL_CASCADE_REGULATING_ADIPOGENESIS |
|  | 13 | 0.927 | 0.024 | 0.254 | 1.31E-04 | GOBP_RETINAL_CONE_CELL_DIFFERENTIATION |
|  | 107 | 0.3 | 0.023 | 0.082 | 1.33E-04 | GOMF_TRANSCRIPTION_COREGULATOR_BINDING |
|  | 5 | 1.736 | 0.028 | 0.499 | 2.54E-04 | GOMF_LBD_DOMAIN_BINDING |
|  | 144 | 0.255 | 0.022 | 0.074 | 2.89E-04 | GOBP_POSITIVE_REGULATION_OF_DEVELOPMENTAL_GROWTH |
|  | 5 | 1.349 | 0.022 | 0.405 | 4.38E-04 | GOCC_CIA_COMPLEX |
|  | 32 | 0.466 | 0.019 | 0.14 | 4.42E-04 | WP_MELATONIN_METABOLISM_AND_EFFECTS |
|  | 20 | 0.692 | 0.023 | 0.209 | 4.67E-04 | GOBP_SPINAL_CORD_PATTERNING |
|  | **Pelvic bone marrow fat** | | | | | |
| 148 | 0.341 | 0.03 | 0.07 | 6.29E-07 | GOBP_CELLULAR_RESPONSE_TO_OXYGEN_LEVELS |  |
| 59 | 0.536 | 0.03 | 0.116 | 2.07E-06 | GOBP_REGULATION_OF_MORPHOGENESIS_OF_AN_EPITHELIUM |  |
| 53 | 0.606 | 0.032 | 0.133 | 2.61E-06 | GOBP_REGULATION_OF_CHONDROCYTE_DIFFERENTIATION |  |
| 13 | 1.368 | 0.036 | 0.308 | 4.38E-06 | WP_TRANSCRIPTIONAL_CASCADE_REGULATING_ADIPOGENESIS |  |
| 353 | 0.206 | 0.028 | 0.047 | 7.22E-06 | GOBP_REGULATION_OF_BINDING |  |
| 16 | 1.141 | 0.033 | 0.267 | 9.83E-06 | GOBP_URETER_DEVELOPMENT |  |
| 9 | 1.388 | 0.03 | 0.333 | 1.53E-05 | GOBP_NEGATIVE_REGULATION_OF_MUSCLE_ADAPTATION |  |
| 24 | 0.724 | 0.026 | 0.174 | 1.66E-05 | COLLER_MYC_TARGETS_UP |  |
| 31 | 0.631 | 0.026 | 0.157 | 2.82E-05 | GOBP_POSITIVE_REGULATION_OF_MORPHOGENESIS_OF_AN_EPITHELIUM |  |
| **Thigh bone marrow fat** | | | | | |  |
| 5 | 2.225 | 0.036 | 0.471 | 1.19E-06 | GOBP_REGULATION_OF_BRANCHING_INVOLVED_IN_PROSTATE_GLAND_MORPHOGENESIS |  |
| 5 | 2.097 | 0.034 | 0.462 | 2.84E-06 | GOBP_AMELOBLAST_DIFFERENTIATION |  |
| 226 | 0.271 | 0.029 | 0.06 | 3.20E-06 | GOBP_FAT_CELL_DIFFERENTIATION |  |
| 128 | 0.358 | 0.029 | 0.08 | 3.39E-06 | GOBP_REGULATION_OF_FAT_CELL_DIFFERENTIATION |  |
| 151 | 0.302 | 0.027 | 0.07 | 7.11E-06 | WP_BREAST_CANCER_PATHWAY |  |
| 266 | 0.21 | 0.025 | 0.054 | 4.56E-05 | HELLER_HDAC_TARGETS_SILENCED_BY_METHYLATION_DN |  |
| 9 | 0.947 | 0.021 | 0.243 | 4.77E-05 | GOMF_ANAPHASE_PROMOTING_COMPLEX_BINDING |  |
| 207 | 0.222 | 0.023 | 0.057 | 4.98E-05 | BENPORATH_MYC_TARGETS_WITH_EBOX |  |
| 7 | 1.376 | 0.026 | 0.355 | 5.24E-05 | GOBP_POSITIVE_REGULATION_OF_FEVER_GENERATION |  |
| **Vertebrae bone marrow fat** | | | | | |  |
| 107 | 0.307 | 0.023 | 0.077 | 3.53E-05 | GOCC_PIGMENT_GRANULE |  |
| 16 | 0.809 | 0.024 | 0.22 | 1.16E-04 | GOBP_OPSONIZATION |  |
| 209 | 0.221 | 0.023 | 0.061 | 1.36E-04 | REACTOME_BIOLOGICAL_OXIDATIONS |  |
| 8 | 1.199 | 0.025 | 0.34 | 2.10E-04 | GOBP_REGULATION_OF_PEPTIDYL_CYSTEINE_S_NITROSYLATION |  |
| 12 | 1.089 | 0.027 | 0.324 | 3.80E-04 | REACTOME_BUTYROPHILIN_BTN_FAMILY_INTERACTIONS |  |
| 44 | 0.384 | 0.019 | 0.115 | 4.12E-04 | PID_ERBB2_ERBB3_PATHWAY |  |
| 6 | 1.236 | 0.022 | 0.37 | 4.19E-04 | GOBP_SUCCINATE_TRANSMEMBRANE_TRANSPORT |  |
| 6 | 1.236 | 0.022 | 0.37 | 4.19E-04 | GOMF_SUCCINATE_TRANSMEMBRANE_TRANSPORTER_ACTIVITY |  |
| 1452 | 0.076 | 0.02 | 0.023 | 6.17E-04 | GOMF_SEQUENCE_SPECIFIC_DNA_BINDING |  |

**Supplementary table 5. Sex specific loci.** IDP: fat depot; Chr: chromosome; P: statistical significance.

| SNP | IDP | Chr | Position | Effect allele | Beta | P | Nearest Gene | eQTL gene | eQTL tissue (P) | Other phenotypes |
| --- | --- | --- | --- | --- | --- | --- | --- | --- | --- | --- |
| rs1275519 | Liver PDFF- male | 2 | 27423646 | C | 0.119 | 3.6E-8 | *SLC5A6* | *GTF3C2* | Thyroid (9.3E-8) | Triglycerides |
| rs2596827 | Pancreas PDFF- female | 3 | 12546172 | G | -0.066 | 1E-8 | *TSEN2* | *TSEN2* | Brain - Cerebellum (1.6E-10); Pancreas (7.7E-6) | Type 2 diabetes |
| rs1208931 | Pelvic BM fat- male | 1 | 23429222 | G | 0.086 | 8.6E-9 | *LUZP1* | *LACTBL1* | Testis (6.6E-22) | BMI |
| rs727479 | Pelvic BM fat- male | 15 | 51534547 | A | 0.056 | 1.3E-8 | *CYP19A1* | *SPPL2A* | Adipose-Subcutaneous (1E-9); Cells-Cultured fibroblasts (2.9E-9) | Bone mineral density, sex hormones, WHR |
| rs10782417 | Pelvic BM fat- female | 14 | 50640692 | T | 0.050 | 3.5E-8 | *SOS2* | *VCPKMT* | Thyroid (5.6E-14); Cells-Cultured fibroblasts (2E-9); Brain-Frontal Cortex (3.4E-10) | Novel |
| rs7491585 | Thigh subcutaneous AT- female | 13 | 102039737 | T | 0.055 | 4.1E-8 | *NALCN* | *NALCN* | Cells - Cultured fibroblasts /2.2E-24 | Novel |
| rs1812736 | Visceral AT- male | 8 | 76299138 | A | 0.077 | 1.6E-8 | *PKMP4* | *HNF4G* | Brain - Cerebellum /9.3E-5 | BMI |

**Supplementary table. 6. Summary of the genetic instruments selected for the ten IDPs.** CHR: chromosome; POS: position (GRch37); EAF: effect allele frequency; SE: the standard error of beta; HetIsq: the variation in effect estimate between sexes; HetPVal: the p-value for Cochran's Q test, a p-value <4 x 10^-4^ indicates significant heterogeneity in the genetic effects between males and females.

| **IDP** | **rsID** | **CHR** | **POS** | **Effect allele** | **Other allele** | **EAF** | **BETA** | **SE** | **P.value** | **HetISq** | **HetPVal** |
| --- | --- | --- | --- | --- | --- | --- | --- | --- | --- | --- | --- |
| Abdominal subcutaneous AT | rs1421085 | 16 | 53800954 | C | T | 0.40 | 0.07 | 0.01 | 1.3E-21 | 0 | 8.0E-01 |
| Abdominal subcutaneous AT | rs62120394 | 19 | 18338709 | A | G | 0.29 | 0.05 | 0.01 | 5.5E-10 | 0 | 9.2E-01 |
| Thigh subcutaneous AT | rs62161459 | 2 | 112250665 | T | C | 0.21 | 0.04 | 0.01 | 2.0E-09 | 9.3 | 2.9E-01 |
| Thigh subcutaneous AT | rs13389219 | 2 | 165528876 | T | C | 0.40 | 0.05 | 0.01 | 2.3E-19 | 73.8 | 5.1E-02 |
| Thigh subcutaneous AT | rs78058190 | 2 | 219699999 | G | A | 0.95 | 0.09 | 0.01 | 1.1E-09 | 0 | 5.3E-01 |
| Thigh subcutaneous AT | rs2972146 | 2 | 227100698 | G | T | 0.35 | 0.03 | 0.01 | 3.3E-08 | 0 | 3.5E-01 |
| Thigh subcutaneous AT | rs66815886 | 3 | 64703394 | T | G | 0.27 | 0.04 | 0.01 | 1.3E-09 | 26.9 | 2.4E-01 |
| Thigh subcutaneous AT | rs9837325 | 3 | 129315831 | A | C | 0.20 | 0.04 | 0.01 | 3.7E-08 | 55.6 | 1.3E-01 |
| Thigh subcutaneous AT | rs10049088 | 3 | 156797648 | T | C | 0.39 | 0.04 | 0.01 | 3.9E-10 | 0 | 6.4E-01 |
| Thigh subcutaneous AT | rs7660000 | 4 | 89751858 | T | C | 0.28 | 0.04 | 0.01 | 3.0E-08 | 0 | 5.5E-01 |
| Thigh subcutaneous AT | rs13107325 | 4 | 103188709 | T | C | 0.07 | 0.08 | 0.01 | 1.0E-12 | 0 | 7.7E-01 |
| Thigh subcutaneous AT | rs3936511 | 5 | 55860781 | A | G | 0.81 | 0.06 | 0.01 | 1.3E-15 | 83 | 1.5E-02 |
| Thigh subcutaneous AT | rs55646464 | 5 | 173324971 | G | T | 0.70 | 0.04 | 0.01 | 8.0E-10 | 0 | 7.5E-01 |
| Thigh subcutaneous AT | rs113176001 | 6 | 32571483 | G | C | 0.58 | 0.03 | 0.01 | 3.5E-08 | 40.5 | 2.0E-01 |
| Thigh subcutaneous AT | rs4711750 | 6 | 43757082 | T | A | 0.50 | 0.06 | 0.01 | 1.8E-23 | 0 | 5.2E-01 |
| Thigh subcutaneous AT | rs72959041 | 6 | 127454893 | G | A | 0.95 | 0.12 | 0.01 | 2.9E-19 | 0 | 9.0E-01 |
| Thigh subcutaneous AT | rs487060 | 6 | 160774459 | T | C | 0.47 | 0.04 | 0.01 | 1.7E-11 | 0 | 8.8E-01 |
| Thigh subcutaneous AT | rs12549728 | 8 | 72463034 | T | G | 0.95 | 0.08 | 0.01 | 1.1E-09 | 12 | 2.9E-01 |
| Thigh subcutaneous AT | rs1962883 | 9 | 107722705 | C | T | 0.53 | 0.04 | 0.01 | 1.6E-12 | 78.7 | 3.0E-02 |
| Thigh subcutaneous AT | rs7133378 | 12 | 124409502 | A | G | 0.32 | 0.06 | 0.01 | 1.6E-22 | 86.8 | 5.9E-03 |
| Thigh subcutaneous AT | rs275182 | 15 | 39446211 | G | A | 0.16 | 0.05 | 0.01 | 9.7E-11 | 64.2 | 9.5E-02 |
| Thigh subcutaneous AT | rs9937053 | 16 | 53799507 | A | G | 0.42 | 0.03 | 0.01 | 8.1E-09 | 0 | 6.3E-01 |
| Thigh subcutaneous AT | rs644710 | 18 | 11983680 | T | C | 0.29 | 0.04 | 0.01 | 1.8E-08 | 0 | 4.2E-01 |
| Thigh subcutaneous AT | rs6029180 | 20 | 39178923 | G | A | 0.33 | 0.04 | 0.01 | 1.5E-11 | 0 | 9.6E-01 |
| Thigh subcutaneous AT | rs4820323 | 22 | 38599767 | C | G | 0.42 | 0.05 | 0.01 | 3.3E-15 | 75.5 | 4.3E-02 |
| Thigh intermuscular AT | rs11096542 | 2 | 18707873 | G | A | 0.41 | 0.04 | 0.01 | 3.3E-08 | 0 | 5.6E-01 |
| Thigh intermuscular AT | rs4848620 | 2 | 121442017 | T | C | 0.06 | 0.08 | 0.01 | 2.8E-09 | 0 | 6.8E-01 |
| Thigh intermuscular AT | rs2138157 | 2 | 227103717 | A | C | 0.35 | 0.05 | 0.01 | 2.1E-11 | 86.9 | 5.8E-03 |
| Thigh intermuscular AT | rs17036160 | 3 | 12329783 | T | C | 0.12 | 0.09 | 0.01 | 3.1E-16 | 15.6 | 2.8E-01 |
| Thigh intermuscular AT | rs6536204 | 4 | 157668779 | G | C | 0.32 | 0.05 | 0.01 | 4.4E-10 | 0 | 9.6E-01 |
| Thigh intermuscular AT | rs151804 | 5 | 53452060 | G | A | 0.27 | 0.05 | 0.01 | 1.9E-11 | 0 | 5.7E-01 |
| Thigh intermuscular AT | rs1651274 | 5 | 158020425 | A | G | 0.77 | 0.06 | 0.01 | 1.3E-12 | 38.1 | 2.0E-01 |
| Thigh intermuscular AT | rs7764488 | 6 | 133812872 | A | G | 0.32 | 0.05 | 0.01 | 3.9E-10 | 0 | 9.4E-01 |
| Thigh intermuscular AT | rs2167330 | 10 | 36463771 | A | G | 0.62 | 0.05 | 0.01 | 3.8E-11 | 0 | 8.7E-01 |
| Thigh intermuscular AT | rs77481804 | 11 | 95924924 | G | A | 0.11 | 0.06 | 0.01 | 6.9E-09 | 13.1 | 2.8E-01 |
| Thigh intermuscular AT | rs12369443 | 12 | 20582651 | G | A | 0.20 | 0.05 | 0.01 | 4.8E-09 | 0 | 8.8E-01 |
| Thigh intermuscular AT | rs9545468 | 13 | 81122878 | A | G | 0.34 | 0.05 | 0.01 | 1.7E-11 | 0 | 5.9E-01 |
| Thigh intermuscular AT | rs33823 | 19 | 34000725 | T | C | 0.64 | 0.06 | 0.01 | 5.3E-19 | 0 | 5.4E-01 |
| Thigh intermuscular AT | rs6018185 | 20 | 45557451 | T | C | 0.41 | 0.05 | 0.01 | 1.6E-11 | 66.3 | 8.5E-02 |
| Visceral AT | rs935166 | 2 | 26949366 | G | A | 0.49 | 0.03 | 0.01 | 2.2E-08 | 51.1 | 1.5E-01 |
| Visceral AT | rs455660 | 5 | 55816888 | T | C | 0.19 | 0.05 | 0.01 | 4.2E-09 | 70 | 6.8E-02 |
| Visceral AT | rs73221948 | 8 | 25464670 | G | T | 0.71 | 0.05 | 0.01 | 2.1E-12 | 0 | 8.9E-01 |
| Visceral AT | rs62033399 | 16 | 53810943 | T | C | 0.39 | 0.04 | 0.01 | 6.6E-09 | 0 | 7.7E-01 |
| Visceral AT | rs11666808 | 19 | 18383506 | T | C | 0.37 | 0.04 | 0.01 | 2.5E-09 | 0 | 9.1E-01 |
| Visceral AT | rs153701 | 19 | 34003255 | T | C | 0.45 | 0.04 | 0.01 | 5.9E-12 | 36.5 | 2.1E-01 |
| Visceral AT | rs9979882 | 21 | 45508682 | G | A | 0.83 | 0.05 | 0.01 | 2.2E-09 | 0 | 7.7E-01 |
| Liver PDFF | rs1260326 | 2 | 27730940 | T | C | 0.39 | 0.05 | 0.01 | 1.2E-11 | 0 | 6.8E-01 |
| Liver PDFF | rs1229984 | 4 | 100239319 | C | T | 0.98 | 0.14 | 0.02 | 1.4E-08 | 59.2 | 1.2E-01 |
| Liver PDFF | rs112875651 | 8 | 126506694 | G | A | 0.61 | 0.05 | 0.01 | 1.3E-11 | 0 | 9.7E-01 |
| Liver PDFF | rs7029757 | 9 | 132566666 | G | A | 0.90 | 0.07 | 0.01 | 1.5E-08 | 0 | 5.6E-01 |
| Liver PDFF | rs7096937 | 10 | 113950418 | T | C | 0.27 | 0.06 | 0.01 | 1.1E-10 | 83.8 | 1.3E-02 |
| Liver PDFF | rs58542926 | 19 | 19379549 | T | C | 0.07 | 0.28 | 0.01 | 3.2E-84 | 93.3 | 1.2E-04 |
| Liver PDFF | rs188247550 | 19 | 19396616 | T | C | 0.01 | 0.27 | 0.03 | 1.1E-16 | 27.1 | 2.4E-01 |
| Liver PDFF | rs429358 | 19 | 45411941 | T | C | 0.85 | 0.11 | 0.01 | 6.8E-27 | 76.6 | 3.9E-02 |
| Liver PDFF | rs738408 | 22 | 44324730 | T | C | 0.21 | 0.23 | 0.01 | 9.1E-139 | 0 | 4.4E-01 |
| Pancreas PDFF | rs1341982 | 1 | 51443441 | C | T | 0.67 | 0.07 | 0.01 | 4.9E-17 | 5 | 3.1E-01 |
| Pancreas PDFF | rs11893393 | 2 | 208924910 | A | G | 0.76 | 0.05 | 0.01 | 8.2E-09 | 43.9 | 1.8E-01 |
| Pancreas PDFF | rs75287519 | 3 | 149202565 | T | A | 0.52 | 0.04 | 0.01 | 3.2E-08 | 0 | 4.5E-01 |
| Pancreas PDFF | rs73221948 | 8 | 25464670 | G | T | 0.71 | 0.05 | 0.01 | 1.3E-08 | 0 | 7.8E-01 |
| Pancreas PDFF | rs4733612 | 8 | 129569999 | G | A | 0.27 | 0.06 | 0.01 | 1.5E-11 | 34.3 | 2.2E-01 |
| Pancreas PDFF | rs115478735 | 9 | 136149711 | T | A | 0.18 | 0.07 | 0.01 | 3.9E-12 | 0 | 5.0E-01 |
| Pancreas PDFF | rs2163150 | 10 | 49335932 | C | T | 0.66 | 0.06 | 0.01 | 1.8E-10 | 0 | 5.4E-01 |
| Pancreas PDFF | rs7138126 | 12 | 4133595 | G | A | 0.42 | 0.05 | 0.01 | 1.2E-10 | 0 | 6.6E-01 |
| Pancreas PDFF | rs7405380 | 16 | 88975910 | C | G | 0.38 | 0.05 | 0.01 | 4.9E-10 | 4.7 | 3.1E-01 |
| Pancreas PDFF | rs10422861 | 19 | 33894846 | T | C | 0.66 | 0.08 | 0.01 | 3.7E-25 | 0 | 7.2E-01 |
| Pancreas PDFF | rs8112983 | 19 | 49229525 | C | T | 0.54 | 0.05 | 0.01 | 3.0E-09 | 0 | 5.9E-01 |
| Pancreas PDFF | rs13040225 | 20 | 48830772 | T | A | 0.46 | 0.06 | 0.01 | 1.6E-15 | 0 | 8.4E-01 |
| Pancreas PDFF | rs75159625 | 22 | 46377008 | G | T | 0.32 | 0.05 | 0.01 | 2.5E-09 | 0 | 5.4E-01 |
| Paraspinal AT | rs7649970 | 3 | 12392272 | T | C | 0.12 | 0.10 | 0.01 | 3.7E-16 | 0 | 8.9E-01 |
| Paraspinal AT | rs9835572 | 3 | 24331457 | G | A | 0.27 | 0.05 | 0.01 | 1.6E-08 | 0 | 9.4E-01 |
| Paraspinal AT | rs3777866 | 6 | 133796586 | T | C | 0.32 | 0.06 | 0.01 | 1.2E-11 | 37.7 | 2.1E-01 |
| Paraspinal AT | rs1481758 | 8 | 32131798 | T | C | 0.22 | 0.05 | 0.01 | 2.6E-08 | 0 | 7.3E-01 |
| Paraspinal AT | rs2167330 | 10 | 36463771 | A | G | 0.62 | 0.05 | 0.01 | 3.1E-10 | 48.7 | 1.6E-01 |
| Paraspinal AT | rs504366 | 13 | 22351517 | G | A | 0.34 | 0.08 | 0.01 | 4.5E-23 | 0 | 8.0E-01 |
| Paraspinal AT | rs62033406 | 16 | 53824226 | G | A | 0.41 | 0.05 | 0.01 | 1.9E-09 | 0 | 7.4E-01 |
| Paraspinal AT | rs10405598 | 19 | 33891195 | A | G | 0.27 | 0.08 | 0.01 | 1.1E-18 | 4.3 | 3.1E-01 |
| Pelvic BM fat | rs12410251 | 1 | 22482629 | T | G | 0.20 | 0.05 | 0.01 | 9.0E-10 | 0 | 4.7E-01 |
| Pelvic BM fat | rs10493013 | 1 | 22703035 | T | C | 0.82 | 0.14 | 0.01 | 5.6E-65 | 83.9 | 1.3E-02 |
| Pelvic BM fat | rs6682650 | 1 | 27780127 | G | A | 0.92 | 0.06 | 0.01 | 4.4E-08 | 0 | 6.4E-01 |
| Pelvic BM fat | rs57748040 | 1 | 68660893 | G | A | 0.74 | 0.04 | 0.01 | 2.8E-08 | 0 | 4.1E-01 |
| Pelvic BM fat | rs12044944 | 1 | 240581653 | C | T | 0.81 | 0.06 | 0.01 | 1.8E-11 | 0 | 5.1E-01 |
| Pelvic BM fat | rs17036101 | 3 | 12277845 | A | G | 0.06 | 0.08 | 0.01 | 7.0E-09 | 80.4 | 2.4E-02 |
| Pelvic BM fat | rs2648308 | 3 | 12614107 | T | C | 0.40 | 0.06 | 0.01 | 8.4E-22 | 87.9 | 4.1E-03 |
| Pelvic BM fat | rs7734992 | 5 | 1280128 | T | C | 0.58 | 0.05 | 0.01 | 8.4E-15 | 20.4 | 2.6E-01 |
| Pelvic BM fat | rs41284511 | 6 | 31377754 | G | A | 0.85 | 0.06 | 0.01 | 4.7E-10 | 0 | 3.2E-01 |
| Pelvic BM fat | rs716013 | 6 | 121815687 | A | T | 0.82 | 0.06 | 0.01 | 6.9E-13 | 10.5 | 2.9E-01 |
| Pelvic BM fat | rs72991933 | 6 | 133632199 | A | G | 0.68 | 0.04 | 0.01 | 8.4E-11 | 0 | 5.7E-01 |
| Pelvic BM fat | rs9494167 | 6 | 135489069 | C | T | 0.20 | 0.05 | 0.01 | 1.9E-09 | 0 | 4.7E-01 |
| Pelvic BM fat | rs1856859 | 6 | 151860110 | G | T | 0.10 | 0.08 | 0.01 | 2.4E-15 | 0 | 6.6E-01 |
| Pelvic BM fat | rs4708612 | 6 | 168342453 | A | G | 0.39 | 0.04 | 0.01 | 2.3E-08 | 52.5 | 1.5E-01 |
| Pelvic BM fat | rs10488618 | 7 | 38013198 | T | C | 0.11 | 0.06 | 0.01 | 7.3E-09 | 21.2 | 2.6E-01 |
| Pelvic BM fat | rs4448201 | 7 | 96154912 | G | C | 0.34 | 0.05 | 0.01 | 1.1E-12 | 25.9 | 2.5E-01 |
| Pelvic BM fat | rs4236785 | 8 | 108291877 | A | T | 0.75 | 0.04 | 0.01 | 4.6E-08 | 0 | 5.2E-01 |
| Pelvic BM fat | rs1475718 | 9 | 137118170 | A | G | 0.44 | 0.06 | 0.01 | 6.8E-24 | 0 | 3.6E-01 |
| Pelvic BM fat | rs17476364 | 10 | 71094504 | T | C | 0.89 | 0.08 | 0.01 | 1.5E-15 | 78.1 | 3.3E-02 |
| Pelvic BM fat | rs174565 | 11 | 61591636 | C | G | 0.87 | 0.06 | 0.01 | 3.4E-10 | 82.6 | 1.6E-02 |
| Pelvic BM fat | rs258399 | 12 | 28008199 | G | C | 0.63 | 0.05 | 0.01 | 6.0E-14 | 0 | 8.4E-01 |
| Pelvic BM fat | rs6487672 | 12 | 28423346 | G | T | 0.77 | 0.08 | 0.01 | 1.4E-27 | 0 | 4.0E-01 |
| Pelvic BM fat | rs10774625 | 12 | 111910219 | G | A | 0.51 | 0.06 | 0.01 | 8.8E-19 | 0 | 8.6E-01 |
| Pelvic BM fat | rs76428106 | 13 | 28604007 | T | C | 0.99 | 0.20 | 0.03 | 4.8E-12 | 80 | 2.5E-02 |
| Pelvic BM fat | rs8001611 | 13 | 42965694 | C | T | 0.49 | 0.05 | 0.01 | 8.9E-13 | 0 | 4.9E-01 |
| Pelvic BM fat | rs56030650 | 17 | 38131187 | C | A | 0.52 | 0.05 | 0.01 | 9.0E-17 | 0 | 6.3E-01 |
| Pelvic BM fat | rs12985107 | 19 | 1163596 | A | G | 0.68 | 0.04 | 0.01 | 4.9E-10 | 0 | 3.2E-01 |
| Pelvic BM fat | rs6088801 | 20 | 33942261 | G | A | 0.37 | 0.04 | 0.01 | 2.3E-11 | 50.6 | 1.6E-01 |
| Pelvic BM fat | rs6018158 | 20 | 45538434 | C | T | 0.60 | 0.04 | 0.01 | 1.4E-08 | 0 | 7.1E-01 |
| Thigh BM fat | rs12123076 | 1 | 8838528 | G | A | 0.32 | 0.04 | 0.01 | 3.3E-08 | 0 | 3.5E-01 |
| Thigh BM fat | rs12410251 | 1 | 22482629 | T | G | 0.20 | 0.05 | 0.01 | 2.8E-08 | 68.9 | 7.3E-02 |
| Thigh BM fat | rs7537281 | 1 | 22692078 | A | T | 0.82 | 0.08 | 0.01 | 1.7E-19 | 72.5 | 5.7E-02 |
| Thigh BM fat | rs6696954 | 1 | 66054995 | G | T | 0.66 | 0.04 | 0.01 | 6.5E-09 | 0 | 3.9E-01 |
| Thigh BM fat | rs4684847 | 3 | 12386337 | T | C | 0.12 | 0.08 | 0.01 | 1.3E-13 | 0 | 7.6E-01 |
| Thigh BM fat | rs572076167 | 3 | 132434862 | G | T | 0.14 | 0.05 | 0.01 | 1.3E-08 | 0 | 7.3E-01 |
| Thigh BM fat | rs1874643 | 4 | 55751907 | A | G | 0.23 | 0.05 | 0.01 | 2.3E-11 | 0 | 7.4E-01 |
| Thigh BM fat | rs4425336 | 4 | 89753225 | A | G | 0.79 | 0.05 | 0.01 | 1.2E-08 | 75.2 | 4.5E-02 |
| Thigh BM fat | rs2508702 | 5 | 98898040 | C | G | 0.39 | 0.04 | 0.01 | 2.5E-08 | 0 | 4.9E-01 |
| Thigh BM fat | rs79276873 | 6 | 22842340 | A | G | 0.86 | 0.06 | 0.01 | 3.7E-09 | 65.9 | 8.7E-02 |
| Thigh BM fat | rs4869745 | 6 | 151908076 | T | C | 0.29 | 0.06 | 0.01 | 7.5E-19 | 0 | 7.4E-01 |
| Thigh BM fat | rs3020332 | 6 | 152008924 | C | T | 0.55 | 0.04 | 0.01 | 4.9E-09 | 0 | 4.3E-01 |
| Thigh BM fat | rs10488618 | 7 | 38013198 | T | C | 0.11 | 0.07 | 0.01 | 2.1E-11 | 0 | 4.9E-01 |
| Thigh BM fat | rs4448201 | 7 | 96154912 | G | C | 0.34 | 0.05 | 0.01 | 2.4E-12 | 0 | 8.3E-01 |
| Thigh BM fat | rs45467892 | 7 | 99332997 | A | T | 0.03 | 0.12 | 0.02 | 4.5E-09 | 74.2 | 4.9E-02 |
| Thigh BM fat | rs3779381 | 7 | 120966790 | A | G | 0.74 | 0.07 | 0.01 | 4.9E-19 | 0 | 9.9E-01 |
| Thigh BM fat | rs2808288 | 10 | 27870856 | G | A | 0.23 | 0.04 | 0.01 | 2.6E-08 | 20.6 | 2.6E-01 |
| Thigh BM fat | rs1896995 | 10 | 65365385 | T | C | 0.48 | 0.04 | 0.01 | 2.8E-08 | 0 | 5.3E-01 |
| Thigh BM fat | rs17476364 | 10 | 71094504 | T | C | 0.89 | 0.07 | 0.01 | 2.0E-12 | 65.1 | 9.0E-02 |
| Thigh BM fat | rs11599750 | 10 | 101805442 | C | T | 0.59 | 0.04 | 0.01 | 2.4E-08 | 0 | 5.0E-01 |
| Thigh BM fat | rs4439535 | 11 | 86326044 | A | G | 0.68 | 0.05 | 0.01 | 3.8E-13 | 0 | 9.8E-01 |
| Thigh BM fat | rs76895963 | 12 | 4384844 | G | T | 0.02 | 0.15 | 0.03 | 1.7E-08 | 49.8 | 1.6E-01 |
| Thigh BM fat | rs258399 | 12 | 28008199 | G | C | 0.63 | 0.05 | 0.01 | 2.9E-13 | 0 | 4.2E-01 |
| Thigh BM fat | rs2061759 | 12 | 28463327 | G | A | 0.76 | 0.06 | 0.01 | 1.1E-14 | 15.9 | 2.8E-01 |
| Thigh BM fat | rs8001611 | 13 | 42965694 | C | T | 0.49 | 0.06 | 0.01 | 1.3E-19 | 34.3 | 2.2E-01 |
| Thigh BM fat | rs78667121 | 13 | 43200103 | G | A | 0.97 | 0.11 | 0.02 | 6.0E-09 | 0 | 7.5E-01 |
| Thigh BM fat | rs9521512 | 13 | 110441598 | T | C | 0.59 | 0.04 | 0.01 | 2.2E-08 | 0 | 4.3E-01 |
| Thigh BM fat | rs146939415 | 15 | 51522210 | G | C | 0.99 | 0.19 | 0.03 | 5.8E-09 | 44.7 | 1.8E-01 |
| Thigh BM fat | rs7216991 | 17 | 45052253 | A | G | 0.47 | 0.04 | 0.01 | 1.4E-08 | 0 | 8.2E-01 |
| Thigh BM fat | rs6016547 | 20 | 39962991 | A | C | 0.83 | 0.05 | 0.01 | 1.3E-09 | 55.5 | 1.3E-01 |
| Vertebrae BM fat | rs705963 | 3 | 12524642 | A | C | 0.42 | 0.06 | 0.01 | 5.3E-13 | 0 | 6.6E-01 |
| Vertebrae BM fat | rs218265 | 4 | 55408999 | T | C | 0.85 | 0.07 | 0.01 | 1.4E-08 | 0 | 8.0E-01 |
| Vertebrae BM fat | rs2468532 | 5 | 36482830 | G | A | 0.14 | 0.07 | 0.01 | 7.9E-09 | 0 | 5.3E-01 |
| Vertebrae BM fat | rs201857861 | 6 | 168347869 | C | T | 0.37 | 0.06 | 0.01 | 3.7E-11 | 0 | 6.8E-01 |
| Vertebrae BM fat | rs4807612 | 19 | 1156853 | T | C | 0.68 | 0.05 | 0.01 | 3.9E-08 | 23.2 | 2.5E-01 |

**Supplementary table 7. Strength of genetic instruments for the 10 IDPs used in univariable and multivariable Mendelian randomisation.** The **F-statistics** column represents the strength of instruments in univariable Mendelian randomisation analyses. The **conditional F-statistics** column indicates instrument strength in multivariable analyses, accounting for the strength of each exposure conditional on other exposures, F>10 suggesting robust instrument strength.

| **IDP** | **F-statistics** | **Conditional F-statistics** |
| --- | --- | --- |
| Abdominal subcutaneous AT | 55 | 1.0 |
| Thigh subcutaneous AT | 32 | 1.3 |
| Thigh intermuscular AT | 40 | 1.7 |
| Visceral AT | 27 | 1.3 |
| Liver PDFF | 140 | 9.2 |
| Pancreas PDFF | 38 | 4.0 |
| Paraspinal AT | 48 | 1.6 |
| Pelvic BM fat | 48 | 1.3 |
| Thigh BM fat | 38 | 1.7 |
| Vertebrae BM fat | 35 | 1.4 |

**Supplementary table 8. Univariable and multivariable Mendelian randomisation IVW result.** The univariable (UV) and multivariable (MV) Mendelian randomisation IVW results for the effect of IDPs on 94 biomarkers from publicly available genome wide association studies. SE: standard error, BHP: Benjamini-Hochberg corrected p-value, OR: odds ratio and 95% confidence intervals.

| **Exposure** | **Outcome** | **Model** | **nSNP** | **Beta** | **SE** | **P-value** | **OR** | **OR_lci95** | **OR_uci95** | **BHP** |
| --- | --- | --- | --- | --- | --- | --- | --- | --- | --- | --- |
| Abdominal subcutaneous AT | Alanine transaminase | UV | 2 | 0.064 | 0.017 | 1.55E-04 | 1.07 | 1.03 | 1.1 | 1.38E-02 |
| Abdominal subcutaneous AT | Citrate | UV | 2 | 0.112 | 0.053 | 3.68E-02 | 1.12 | 1.01 | 1.24 | 9.81E-01 |
| Abdominal subcutaneous AT | Fasting insulin | UV | 2 | 0.278 | 0.037 | 3.59E-14 | 1.32 | 1.23 | 1.42 | 3.44E-12 |
| Abdominal subcutaneous AT | HDL cholesterol | UV | 2 | -0.254 | 0.092 | 5.90E-03 | 0.78 | 0.65 | 0.93 | 4.72E-01 |
| Abdominal subcutaneous AT | HOMA-B | UV | 2 | 0.153 | 0.052 | 3.59E-03 | 1.16 | 1.05 | 1.29 | 3.02E-01 |
| Abdominal subcutaneous AT | HOMA-IR | UV | 2 | 0.261 | 0.053 | 8.50E-07 | 1.3 | 1.17 | 1.44 | 7.99E-05 |
| Abdominal subcutaneous AT | IFN_G | UV | 2 | 0.421 | 0.211 | 4.61E-02 | 1.52 | 1.01 | 2.31 | 9.81E-01 |
| Abdominal subcutaneous AT | IL_13 | UV | 2 | 0.854 | 0.309 | 5.73E-03 | 2.35 | 1.28 | 4.31 | 4.64E-01 |
| Abdominal subcutaneous AT | IL_1RA | UV | 2 | 0.868 | 0.305 | 4.44E-03 | 2.38 | 1.31 | 4.33 | 3.64E-01 |
| Abdominal subcutaneous AT | IL_2 | UV | 2 | 0.759 | 0.313 | 1.53E-02 | 2.14 | 1.16 | 3.94 | 9.81E-01 |
| Abdominal subcutaneous AT | IL_2RA | UV | 2 | 0.692 | 0.304 | 2.27E-02 | 2 | 1.1 | 3.62 | 9.81E-01 |
| Abdominal subcutaneous AT | IL_5 | UV | 2 | 0.823 | 0.318 | 9.57E-03 | 2.28 | 1.22 | 4.25 | 7.56E-01 |
| Abdominal subcutaneous AT | IL_6 | UV | 2 | 0.413 | 0.205 | 4.35E-02 | 1.51 | 1.01 | 2.26 | 9.81E-01 |
| Abdominal subcutaneous AT | IL_7 | UV | 2 | 0.898 | 0.315 | 4.33E-03 | 2.45 | 1.32 | 4.55 | 3.59E-01 |
| Abdominal subcutaneous AT | IL_8 | UV | 2 | 0.783 | 0.383 | 4.09E-02 | 2.19 | 1.03 | 4.63 | 9.81E-01 |
| Abdominal subcutaneous AT | Insulin release at 30-min increments | UV | 1 | -0.793 | 0.32 | 1.33E-02 | 0.45 | 0.24 | 0.85 | 9.81E-01 |
| Abdominal subcutaneous AT | Insulin sensitivity index | UV | 2 | -0.374 | 0.172 | 2.99E-02 | 0.69 | 0.49 | 0.96 | 9.81E-01 |
| Abdominal subcutaneous AT | IP_10 | UV | 2 | 0.66 | 0.304 | 2.98E-02 | 1.93 | 1.07 | 3.51 | 9.81E-01 |
| Abdominal subcutaneous AT | Isoleucine | UV | 2 | 0.235 | 0.053 | 8.15E-06 | 1.27 | 1.14 | 1.4 | 7.50E-04 |
| Abdominal subcutaneous AT | Leucine | UV | 2 | 0.159 | 0.052 | 2.02E-03 | 1.17 | 1.06 | 1.3 | 1.76E-01 |
| Abdominal subcutaneous AT | MCP_1_MCAF | UV | 2 | 0.49 | 0.203 | 1.59E-02 | 1.63 | 1.1 | 2.43 | 9.81E-01 |
| Abdominal subcutaneous AT | MIP_1A | UV | 2 | 0.676 | 0.31 | 2.93E-02 | 1.97 | 1.07 | 3.61 | 9.81E-01 |
| Abdominal subcutaneous AT | MIP_1B | UV | 2 | 0.603 | 0.203 | 3.04E-03 | 1.83 | 1.23 | 2.72 | 2.59E-01 |
| Abdominal subcutaneous AT | Non-HDL cholesterol | UV | 2 | 0.179 | 0.059 | 2.51E-03 | 1.2 | 1.07 | 1.34 | 2.16E-01 |
| Abdominal subcutaneous AT | Phenylalanine | UV | 2 | 0.171 | 0.077 | 2.60E-02 | 1.19 | 1.02 | 1.38 | 9.81E-01 |
| Abdominal subcutaneous AT | SHBG-female | UV | 2 | -0.401 | 0.09 | 8.42E-06 | 0.67 | 0.56 | 0.8 | 7.66E-04 |
| Abdominal subcutaneous AT | SHBG-male | UV | 2 | -0.242 | 0.057 | 1.93E-05 | 0.78 | 0.7 | 0.88 | 1.74E-03 |
| Abdominal subcutaneous AT | TNF_B | UV | 2 | -1.031 | 0.464 | 2.61E-02 | 0.36 | 0.14 | 0.88 | 9.81E-01 |
| Abdominal subcutaneous AT | Total triglycerides | UV | 2 | 0.256 | 0.069 | 2.05E-04 | 1.29 | 1.13 | 1.48 | 1.81E-02 |
| Abdominal subcutaneous AT | Tyrosine | UV | 2 | 0.253 | 0.054 | 2.38E-06 | 1.29 | 1.16 | 1.43 | 2.21E-04 |
| Abdominal subcutaneous AT | Valine | UV | 2 | 0.262 | 0.052 | 5.52E-07 | 1.3 | 1.17 | 1.44 | 5.24E-05 |
| Abdominal subcutaneous AT | VEGF | UV | 2 | 0.501 | 0.219 | 2.22E-02 | 1.65 | 1.07 | 2.53 | 9.81E-01 |
| Thigh subcutaneous AT | 3-Hydroxybutyrate | UV | 23 | 0.091 | 0.036 | 1.25E-02 | 1.09 | 1.02 | 1.18 | 8.38E-01 |
| Thigh subcutaneous AT | Acetone | UV | 23 | 0.106 | 0.028 | 1.60E-04 | 1.11 | 1.05 | 1.18 | 1.28E-02 |
| Thigh subcutaneous AT | Adiponectin | UV | 19 | 0.239 | 0.062 | 1.16E-04 | 1.27 | 1.12 | 1.43 | 9.49E-03 |
| Thigh subcutaneous AT | Alanine | UV | 23 | -0.196 | 0.033 | 4.52E-09 | 0.82 | 0.77 | 0.88 | 4.20E-07 |
| Thigh subcutaneous AT | Alanine transaminase | UV | 21 | -0.039 | 0.011 | 5.14E-04 | 0.96 | 0.94 | 0.98 | 3.80E-02 |
| Thigh subcutaneous AT | ApoB/apoA1 ratio | UV | 23 | -0.248 | 0.061 | 4.03E-05 | 0.78 | 0.69 | 0.88 | 3.46E-03 |
| Thigh subcutaneous AT | Apolipoprotein A1 | UV | 23 | 0.192 | 0.071 | 7.04E-03 | 1.21 | 1.05 | 1.39 | 4.93E-01 |
| Thigh subcutaneous AT | Apolipoprotein B | UV | 23 | -0.164 | 0.046 | 3.05E-04 | 0.85 | 0.78 | 0.93 | 2.35E-02 |
| Thigh subcutaneous AT | Degree of unsaturation | UV | 23 | 0.135 | 0.059 | 2.22E-02 | 1.14 | 1.02 | 1.29 | 9.68E-01 |
| Thigh subcutaneous AT | Docosahexaenoic acid | UV | 23 | -0.074 | 0.035 | 3.82E-02 | 0.93 | 0.87 | 1 | 9.68E-01 |
| Thigh subcutaneous AT | Gamma glutamyl Transferase | UV | 22 | -0.046 | 0.009 | 2.97E-07 | 0.96 | 0.94 | 0.97 | 2.71E-05 |
| Thigh subcutaneous AT | Glucose | UV | 23 | -0.094 | 0.031 | 2.63E-03 | 0.91 | 0.86 | 0.97 | 1.89E-01 |
| Thigh subcutaneous AT | Glycine | UV | 23 | 0.141 | 0.056 | 1.20E-02 | 1.15 | 1.03 | 1.29 | 8.17E-01 |
| Thigh subcutaneous AT | Glycoprotein acetyls | UV | 23 | -0.182 | 0.052 | 4.50E-04 | 0.83 | 0.75 | 0.92 | 3.42E-02 |
| Thigh subcutaneous AT | HbA1c | UV | 23 | -0.033 | 0.014 | 2.08E-02 | 0.97 | 0.94 | 0.99 | 9.68E-01 |
| Thigh subcutaneous AT | HDL cholesterol | UV | 23 | 0.327 | 0.085 | 1.11E-04 | 1.39 | 1.18 | 1.64 | 9.19E-03 |
| Thigh subcutaneous AT | Histidine | UV | 23 | -0.078 | 0.035 | 2.71E-02 | 0.92 | 0.86 | 0.99 | 9.68E-01 |
| Thigh subcutaneous AT | Insulin disposition index | UV | 15 | 0.391 | 0.15 | 8.97E-03 | 1.48 | 1.1 | 1.98 | 6.19E-01 |
| Thigh subcutaneous AT | Insulin levels | UV | 18 | -0.33 | 0.138 | 1.67E-02 | 0.72 | 0.55 | 0.94 | 9.68E-01 |
| Thigh subcutaneous AT | Insulin response 30 mins | UV | 15 | -0.362 | 0.167 | 2.96E-02 | 0.7 | 0.5 | 0.96 | 9.68E-01 |
| Thigh subcutaneous AT | Insulin sensitivity index | UV | 20 | 0.255 | 0.08 | 1.48E-03 | 1.29 | 1.1 | 1.51 | 1.08E-01 |
| Thigh subcutaneous AT | Isoleucine | UV | 23 | -0.153 | 0.044 | 4.81E-04 | 0.86 | 0.79 | 0.94 | 3.61E-02 |
| Thigh subcutaneous AT | LDL cholesterol | UV | 23 | -0.136 | 0.036 | 1.95E-04 | 0.87 | 0.81 | 0.94 | 1.54E-02 |
| Thigh subcutaneous AT | Leucine | UV | 23 | -0.175 | 0.046 | 1.36E-04 | 0.84 | 0.77 | 0.92 | 1.10E-02 |
| Thigh subcutaneous AT | Monounsaturated fatty acids | UV | 23 | -0.286 | 0.063 | 6.33E-06 | 0.75 | 0.66 | 0.85 | 5.57E-04 |
| Thigh subcutaneous AT | Non-HDL cholesterol | UV | 23 | -0.286 | 0.045 | 1.59E-10 | 0.75 | 0.69 | 0.82 | 1.51E-08 |
| Thigh subcutaneous AT | Omega-3 fatty acids | UV | 23 | -0.22 | 0.037 | 3.00E-09 | 0.8 | 0.75 | 0.86 | 2.82E-07 |
| Thigh subcutaneous AT | omega-6/omega-3 fatty acids ratio | UV | 23 | 0.207 | 0.031 | 1.42E-11 | 1.23 | 1.16 | 1.31 | 1.36E-09 |
| Thigh subcutaneous AT | Polyunsaturated fatty acids | UV | 23 | -0.138 | 0.049 | 4.97E-03 | 0.87 | 0.79 | 0.96 | 3.53E-01 |
| Thigh subcutaneous AT | Proinsulin levels | UV | 18 | -0.097 | 0.042 | 2.04E-02 | 0.91 | 0.84 | 0.99 | 9.68E-01 |
| Thigh subcutaneous AT | PUFA/MUFA ratio | UV | 23 | 0.286 | 0.074 | 1.06E-04 | 1.33 | 1.15 | 1.54 | 8.91E-03 |
| Thigh subcutaneous AT | Saturated fatty acids | UV | 23 | -0.241 | 0.049 | 9.69E-07 | 0.79 | 0.71 | 0.87 | 8.72E-05 |
| Thigh subcutaneous AT | SHBG-female | UV | 22 | 0.282 | 0.062 | 4.84E-06 | 1.33 | 1.17 | 1.5 | 4.31E-04 |
| Thigh subcutaneous AT | SHBG-male | UV | 22 | 0.23 | 0.062 | 2.08E-04 | 1.26 | 1.11 | 1.42 | 1.62E-02 |
| Thigh subcutaneous AT | Total cholesterol | UV | 23 | -0.151 | 0.038 | 8.34E-05 | 0.86 | 0.8 | 0.93 | 7.09E-03 |
| Thigh subcutaneous AT | Total triglycerides | UV | 23 | -0.382 | 0.068 | 1.56E-08 | 0.68 | 0.6 | 0.78 | 1.44E-06 |
| Thigh subcutaneous AT | Tyrosine | UV | 23 | -0.085 | 0.041 | 3.81E-02 | 0.92 | 0.85 | 1 | 9.68E-01 |
| Thigh subcutaneous AT | Valine | UV | 23 | -0.211 | 0.049 | 1.45E-05 | 0.81 | 0.74 | 0.89 | 1.26E-03 |
| Thigh intermuscular AT | Adiponectin | UV | 13 | 0.271 | 0.034 | 1.46E-15 | 1.31 | 1.23 | 1.4 | 1.41E-13 |
| Thigh intermuscular AT | Alanine | UV | 14 | -0.136 | 0.03 | 4.26E-06 | 0.87 | 0.82 | 0.92 | 3.79E-04 |
| Thigh intermuscular AT | Alanine transaminase | UV | 13 | -0.041 | 0.009 | 2.66E-06 | 0.96 | 0.94 | 0.98 | 2.39E-04 |
| Thigh intermuscular AT | ApoB/apoA1 ratio | UV | 14 | -0.086 | 0.028 | 2.53E-03 | 0.92 | 0.87 | 0.97 | 1.77E-01 |
| Thigh intermuscular AT | Apolipoprotein A1 | UV | 14 | 0.139 | 0.045 | 1.98E-03 | 1.15 | 1.05 | 1.25 | 1.41E-01 |
| Thigh intermuscular AT | Degree of unsaturation | UV | 14 | 0.12 | 0.048 | 1.21E-02 | 1.13 | 1.03 | 1.24 | 7.87E-01 |
| Thigh intermuscular AT | Fasting glucose | UV | 14 | -0.031 | 0.014 | 3.18E-02 | 0.97 | 0.94 | 1 | 9.99E-01 |
| Thigh intermuscular AT | Fasting insulin | UV | 14 | -0.132 | 0.027 | 1.24E-06 | 0.88 | 0.83 | 0.92 | 1.13E-04 |
| Thigh intermuscular AT | G_CSF | UV | 14 | 0.193 | 0.096 | 4.47E-02 | 1.21 | 1 | 1.46 | 9.99E-01 |
| Thigh intermuscular AT | Gamma glutamyl Transferase | UV | 14 | -0.047 | 0.009 | 3.76E-07 | 0.95 | 0.94 | 0.97 | 3.46E-05 |
| Thigh intermuscular AT | Glucose | UV | 14 | -0.088 | 0.026 | 5.39E-04 | 0.92 | 0.87 | 0.96 | 4.26E-02 |
| Thigh intermuscular AT | Glutamine | UV | 14 | 0.133 | 0.036 | 2.60E-04 | 1.14 | 1.06 | 1.23 | 2.16E-02 |
| Thigh intermuscular AT | Glycine | UV | 14 | 0.15 | 0.039 | 1.14E-04 | 1.16 | 1.08 | 1.25 | 9.65E-03 |
| Thigh intermuscular AT | Glycoprotein acetyls | UV | 14 | -0.074 | 0.029 | 1.01E-02 | 0.93 | 0.88 | 0.98 | 6.69E-01 |
| Thigh intermuscular AT | HbA1c | UV | 14 | -0.031 | 0.011 | 3.53E-03 | 0.97 | 0.95 | 0.99 | 2.43E-01 |
| Thigh intermuscular AT | HDL cholesterol | UV | 14 | 0.213 | 0.052 | 4.38E-05 | 1.24 | 1.12 | 1.37 | 3.77E-03 |
| Thigh intermuscular AT | HOMA-B | UV | 13 | -0.077 | 0.02 | 1.18E-04 | 0.93 | 0.89 | 0.96 | 9.91E-03 |
| Thigh intermuscular AT | HOMA-IR | UV | 13 | -0.101 | 0.035 | 4.40E-03 | 0.9 | 0.84 | 0.97 | 2.99E-01 |
| Thigh intermuscular AT | IL_10 | UV | 14 | 0.218 | 0.099 | 2.82E-02 | 1.24 | 1.02 | 1.51 | 9.99E-01 |
| Thigh intermuscular AT | IL_18 | UV | 14 | 0.334 | 0.138 | 1.53E-02 | 1.4 | 1.07 | 1.83 | 9.77E-01 |
| Thigh intermuscular AT | Insulin disposition index | UV | 11 | 0.256 | 0.129 | 4.76E-02 | 1.29 | 1 | 1.67 | 9.99E-01 |
| Thigh intermuscular AT | Insulin levels | UV | 13 | -0.453 | 0.129 | 4.72E-04 | 0.64 | 0.49 | 0.82 | 3.78E-02 |
| Thigh intermuscular AT | Insulin release at 30-min increments | UV | 11 | -0.49 | 0.14 | 4.58E-04 | 0.61 | 0.47 | 0.81 | 3.71E-02 |
| Thigh intermuscular AT | Insulin response 30 mins | UV | 11 | -0.493 | 0.144 | 6.34E-04 | 0.61 | 0.46 | 0.81 | 4.82E-02 |
| Thigh intermuscular AT | Insulin sensitivity index | UV | 14 | 0.273 | 0.08 | 6.18E-04 | 1.31 | 1.12 | 1.54 | 4.76E-02 |
| Thigh intermuscular AT | Isoleucine | UV | 14 | -0.123 | 0.038 | 1.03E-03 | 0.88 | 0.82 | 0.95 | 7.59E-02 |
| Thigh intermuscular AT | Leucine | UV | 14 | -0.163 | 0.038 | 2.09E-05 | 0.85 | 0.79 | 0.92 | 1.82E-03 |
| Thigh intermuscular AT | Monounsaturated fatty acids | UV | 14 | -0.12 | 0.035 | 6.83E-04 | 0.89 | 0.83 | 0.95 | 5.13E-02 |
| Thigh intermuscular AT | Non-HDL cholesterol | UV | 14 | -0.07 | 0.022 | 1.51E-03 | 0.93 | 0.89 | 0.97 | 1.09E-01 |
| Thigh intermuscular AT | Omega-3 fatty acids | UV | 14 | -0.076 | 0.027 | 4.59E-03 | 0.93 | 0.88 | 0.98 | 3.08E-01 |
| Thigh intermuscular AT | omega-6/omega-3 fatty acids ratio | UV | 14 | 0.095 | 0.028 | 5.78E-04 | 1.1 | 1.04 | 1.16 | 4.51E-02 |
| Thigh intermuscular AT | PUFA/MUFA ratio | UV | 14 | 0.166 | 0.051 | 1.27E-03 | 1.18 | 1.07 | 1.31 | 9.26E-02 |
| Thigh intermuscular AT | Saturated fatty acids | UV | 14 | -0.098 | 0.027 | 3.23E-04 | 0.91 | 0.86 | 0.96 | 2.65E-02 |
| Thigh intermuscular AT | SCGF_B | UV | 14 | -0.27 | 0.137 | 4.80E-02 | 0.76 | 0.58 | 1 | 9.99E-01 |
| Thigh intermuscular AT | SHBG-female | UV | 14 | 0.275 | 0.047 | 5.49E-09 | 1.32 | 1.2 | 1.44 | 5.22E-07 |
| Thigh intermuscular AT | SHBG-male | UV | 14 | 0.26 | 0.046 | 1.52E-08 | 1.3 | 1.18 | 1.42 | 1.43E-06 |
| Thigh intermuscular AT | Total triglycerides | UV | 14 | -0.237 | 0.046 | 2.93E-07 | 0.79 | 0.72 | 0.86 | 2.72E-05 |
| Thigh intermuscular AT | Tyrosine | UV | 14 | -0.078 | 0.034 | 2.10E-02 | 0.93 | 0.87 | 0.99 | 9.99E-01 |
| Thigh intermuscular AT | Valine | UV | 14 | -0.178 | 0.04 | 7.81E-06 | 0.84 | 0.77 | 0.9 | 6.87E-04 |
| Visceral AT | Acetoacetate | UV | 7 | 0.103 | 0.051 | 4.23E-02 | 1.11 | 1 | 1.22 | 9.90E-01 |
| Visceral AT | Degree of unsaturation | UV | 7 | 0.198 | 0.068 | 3.68E-03 | 1.22 | 1.07 | 1.39 | 3.50E-01 |
| Visceral AT | IL_5 | UV | 7 | 0.693 | 0.247 | 5.07E-03 | 2 | 1.23 | 3.25 | 4.76E-01 |
| Visceral AT | IL_7 | UV | 7 | 0.716 | 0.246 | 3.57E-03 | 2.05 | 1.26 | 3.31 | 3.42E-01 |
| Visceral AT | MCP_1_MCAF | UV | 7 | 0.412 | 0.176 | 1.93E-02 | 1.51 | 1.07 | 2.13 | 9.90E-01 |
| Liver PDFF | Alanine transaminase | UV | 9 | 0.079 | 0.008 | 1.06E-20 | 1.08 | 1.06 | 1.1 | 1.02E-18 |
| Liver PDFF | Phenylalanine | UV | 9 | 0.049 | 0.021 | 2.14E-02 | 1.05 | 1.01 | 1.09 | 9.77E-01 |
| Liver PDFF | Tyrosine | UV | 9 | 0.145 | 0.054 | 7.71E-03 | 1.16 | 1.04 | 1.29 | 7.32E-01 |
| Pancreas PDFF | 3-Hydroxybutyrate | UV | 13 | 0.063 | 0.022 | 5.32E-03 | 1.06 | 1.02 | 1.11 | 4.94E-01 |
| Pancreas PDFF | Apolipoprotein A1 | UV | 13 | 0.075 | 0.028 | 7.78E-03 | 1.08 | 1.02 | 1.14 | 7.16E-01 |
| Pancreas PDFF | C-Reactive protein level | UV | 6 | 0.138 | 0.056 | 1.32E-02 | 1.15 | 1.03 | 1.28 | 9.71E-01 |
| Pancreas PDFF | Insulin release at 30-min increments | UV | 4 | 0.628 | 0.221 | 4.53E-03 | 1.87 | 1.21 | 2.89 | 4.26E-01 |
| Pancreas PDFF | Insulin response 30 mins | UV | 4 | 0.661 | 0.221 | 2.84E-03 | 1.94 | 1.25 | 2.99 | 2.69E-01 |
| Pancreas PDFF | Leptin | UV | 6 | 0.082 | 0.041 | 4.79E-02 | 1.09 | 1 | 1.18 | 9.71E-01 |
| Pancreas PDFF | MCP_3 | UV | 9 | -0.654 | 0.272 | 1.60E-02 | 0.52 | 0.31 | 0.89 | 9.71E-01 |
| Pancreas PDFF | Omega-3 fatty acids | UV | 13 | -0.054 | 0.022 | 1.49E-02 | 0.95 | 0.91 | 0.99 | 9.71E-01 |
| Pancreas PDFF | omega-6/omega-3 fatty acids ratio | UV | 13 | 0.103 | 0.022 | 3.37E-06 | 1.11 | 1.06 | 1.16 | 3.23E-04 |
| Pancreas PDFF | Proinsulin levels | UV | 5 | -0.125 | 0.056 | 2.42E-02 | 0.88 | 0.79 | 0.98 | 9.71E-01 |
| Pancreas PDFF | Pyruvate | UV | 13 | 0.088 | 0.044 | 4.53E-02 | 1.09 | 1 | 1.19 | 9.71E-01 |
| Paraspinal AT | Adiponectin | UV | 6 | 0.099 | 0.05 | 4.69E-02 | 1.1 | 1 | 1.22 | 9.92E-01 |
| Paraspinal AT | Alanine | UV | 8 | -0.081 | 0.029 | 5.10E-03 | 0.92 | 0.87 | 0.98 | 4.89E-01 |
| Paraspinal AT | Glutamine | UV | 8 | 0.095 | 0.043 | 2.71E-02 | 1.1 | 1.01 | 1.2 | 9.92E-01 |
| Paraspinal AT | IL_7 | UV | 8 | 0.305 | 0.152 | 4.50E-02 | 1.36 | 1.01 | 1.83 | 9.92E-01 |
| Paraspinal AT | SHBG-male | UV | 8 | 0.133 | 0.064 | 3.81E-02 | 1.14 | 1.01 | 1.3 | 9.92E-01 |
| Pelvic bone marrow fat | Apolipoprotein A1 | UV | 29 | 0.075 | 0.035 | 3.38E-02 | 1.08 | 1.01 | 1.15 | 9.79E-01 |
| Pelvic bone marrow fat | Citrate | UV | 29 | 0.045 | 0.02 | 2.29E-02 | 1.05 | 1.01 | 1.09 | 9.79E-01 |
| Pelvic bone marrow fat | Fasting insulin | UV | 25 | -0.036 | 0.015 | 1.87E-02 | 0.97 | 0.94 | 0.99 | 9.79E-01 |
| Pelvic bone marrow fat | Glucose | UV | 29 | 0.077 | 0.027 | 4.64E-03 | 1.08 | 1.02 | 1.14 | 4.40E-01 |
| Pelvic bone marrow fat | HDL cholesterol | UV | 29 | 0.091 | 0.028 | 1.18E-03 | 1.1 | 1.04 | 1.16 | 1.13E-01 |
| Pelvic bone marrow fat | IL_18 | UV | 27 | -0.215 | 0.108 | 4.73E-02 | 0.81 | 0.65 | 1 | 9.79E-01 |
| Pelvic bone marrow fat | Lactate | UV | 29 | -0.087 | 0.035 | 1.20E-02 | 0.92 | 0.86 | 0.98 | 9.79E-01 |
| Pelvic bone marrow fat | LDL cholesterol | UV | 29 | 0.083 | 0.031 | 7.90E-03 | 1.09 | 1.02 | 1.15 | 7.43E-01 |
| Pelvic bone marrow fat | Non-HDL cholesterol | UV | 29 | 0.07 | 0.03 | 1.84E-02 | 1.07 | 1.01 | 1.14 | 9.79E-01 |
| Pelvic bone marrow fat | Pyruvate | UV | 29 | -0.056 | 0.025 | 2.72E-02 | 0.95 | 0.9 | 0.99 | 9.79E-01 |
| Pelvic bone marrow fat | Total cholesterol | UV | 29 | 0.087 | 0.034 | 9.89E-03 | 1.09 | 1.02 | 1.16 | 9.20E-01 |
| Thigh bone marrow fat | Acetone | UV | 30 | 0.05 | 0.024 | 3.41E-02 | 1.05 | 1 | 1.1 | 9.98E-01 |
| Thigh bone marrow fat | Alkaline phosphatase | UV | 30 | 0.021 | 0.008 | 1.14E-02 | 1.02 | 1 | 1.04 | 9.98E-01 |
| Thigh bone marrow fat | Fasting glucose | UV | 25 | -0.051 | 0.021 | 1.33E-02 | 0.95 | 0.91 | 0.99 | 9.98E-01 |
| Thigh bone marrow fat | MCP_1_MCAF | UV | 26 | 0.172 | 0.069 | 1.23E-02 | 1.19 | 1.04 | 1.36 | 9.98E-01 |
| Vertebrae bone marrow fat | 3-Hydroxybutyrate | UV | 5 | -0.071 | 0.034 | 3.59E-02 | 0.93 | 0.87 | 1 | 9.66E-01 |
| Vertebrae bone marrow fat | Apolipoprotein B | UV | 5 | 0.093 | 0.034 | 5.78E-03 | 1.1 | 1.03 | 1.17 | 5.32E-01 |
| Vertebrae bone marrow fat | IL_10 | UV | 4 | 0.344 | 0.174 | 4.83E-02 | 1.41 | 1 | 1.99 | 9.66E-01 |
| Vertebrae bone marrow fat | LDL cholesterol | UV | 5 | 0.135 | 0.028 | 1.87E-06 | 1.14 | 1.08 | 1.21 | 1.80E-04 |
| Vertebrae bone marrow fat | Linoleic acid | UV | 5 | 0.101 | 0.039 | 9.49E-03 | 1.11 | 1.03 | 1.19 | 8.54E-01 |
| Vertebrae bone marrow fat | Monounsaturated fatty acids | UV | 5 | 0.095 | 0.041 | 1.95E-02 | 1.1 | 1.02 | 1.19 | 9.66E-01 |
| Vertebrae bone marrow fat | Non-HDL cholesterol | UV | 5 | 0.168 | 0.044 | 1.53E-04 | 1.18 | 1.08 | 1.29 | 1.45E-02 |
| Vertebrae bone marrow fat | Omega-6 fatty acids | UV | 5 | 0.114 | 0.039 | 3.36E-03 | 1.12 | 1.04 | 1.21 | 3.12E-01 |
| Vertebrae bone marrow fat | Polyunsaturated fatty acids | UV | 5 | 0.111 | 0.041 | 7.33E-03 | 1.12 | 1.03 | 1.21 | 6.67E-01 |
| Vertebrae bone marrow fat | Saturated fatty acids | UV | 5 | 0.085 | 0.034 | 1.11E-02 | 1.09 | 1.02 | 1.16 | 9.66E-01 |
| Vertebrae bone marrow fat | Total cholesterol | UV | 5 | 0.154 | 0.046 | 8.16E-04 | 1.17 | 1.07 | 1.28 | 7.67E-02 |
| Abdominal subcutaneous AT | Fasting insulin | MV | 1 | 0.22 | 0.077 | 4.10E-03 | 1.25 | 1.07 | 1.45 | 3.85E-01 |
| Abdominal subcutaneous AT | Glucose | MV | 1 | 0.205 | 0.093 | 2.71E-02 | 1.23 | 1.02 | 1.47 | 9.88E-01 |
| Abdominal subcutaneous AT | HDL cholesterol | MV | 1 | -0.415 | 0.162 | 1.04E-02 | 0.66 | 0.48 | 0.91 | 9.70E-01 |
| Abdominal subcutaneous AT | Insulin sensitivity index | MV | 1 | -0.498 | 0.126 | 7.46E-05 | 0.61 | 0.48 | 0.78 | 7.16E-03 |
| Abdominal subcutaneous AT | Phenylalanine | MV | 1 | 0.22 | 0.069 | 1.46E-03 | 1.25 | 1.09 | 1.43 | 1.39E-01 |
| Abdominal subcutaneous AT | Tyrosine | MV | 1 | 0.275 | 0.113 | 1.48E-02 | 1.32 | 1.06 | 1.64 | 9.88E-01 |
| Abdominal subcutaneous AT | Valine | MV | 1 | 0.302 | 0.125 | 1.55E-02 | 1.35 | 1.06 | 1.73 | 9.88E-01 |
| Thigh subcutaneous AT | Acetone | MV | 19 | 0.124 | 0.045 | 5.51E-03 | 1.13 | 1.04 | 1.24 | 4.46E-01 |
| Thigh subcutaneous AT | Adiponectin | MV | 16 | 0.22 | 0.074 | 3.00E-03 | 1.25 | 1.08 | 1.44 | 2.58E-01 |
| Thigh subcutaneous AT | Alanine | MV | 19 | -0.248 | 0.067 | 2.19E-04 | 0.78 | 0.68 | 0.89 | 2.01E-02 |
| Thigh subcutaneous AT | ApoB/apoA1 ratio | MV | 19 | -0.359 | 0.133 | 6.95E-03 | 0.7 | 0.54 | 0.91 | 5.35E-01 |
| Thigh subcutaneous AT | Apolipoprotein A1 | MV | 19 | 0.258 | 0.087 | 3.10E-03 | 1.29 | 1.09 | 1.54 | 2.63E-01 |
| Thigh subcutaneous AT | Corrected insulin response | MV | 9 | -0.265 | 0.134 | 4.73E-02 | 0.77 | 0.59 | 1 | 9.93E-01 |
| Thigh subcutaneous AT | Fasting insulin | MV | 16 | -0.144 | 0.042 | 5.72E-04 | 0.87 | 0.8 | 0.94 | 5.15E-02 |
| Thigh subcutaneous AT | Gamma glutamyl Transferase | MV | 19 | -0.042 | 0.017 | 1.27E-02 | 0.96 | 0.93 | 0.99 | 9.43E-01 |
| Thigh subcutaneous AT | Glucose | MV | 19 | -0.136 | 0.053 | 9.71E-03 | 0.87 | 0.79 | 0.97 | 7.38E-01 |
| Thigh subcutaneous AT | Glycine | MV | 19 | 0.206 | 0.075 | 6.00E-03 | 1.23 | 1.06 | 1.42 | 4.72E-01 |
| Thigh subcutaneous AT | Glycoprotein acetyls | MV | 19 | -0.255 | 0.114 | 2.57E-02 | 0.78 | 0.62 | 0.97 | 9.93E-01 |
| Thigh subcutaneous AT | HDL cholesterol | MV | 19 | 0.464 | 0.092 | 3.93E-07 | 1.59 | 1.33 | 1.9 | 3.73E-05 |
| Thigh subcutaneous AT | HOMA-B | MV | 15 | -0.084 | 0.036 | 2.13E-02 | 0.92 | 0.86 | 0.99 | 9.93E-01 |
| Thigh subcutaneous AT | HOMA-IR | MV | 15 | -0.143 | 0.05 | 4.17E-03 | 0.87 | 0.79 | 0.96 | 3.42E-01 |
| Thigh subcutaneous AT | IL_13 | MV | 17 | -0.483 | 0.221 | 2.86E-02 | 0.62 | 0.4 | 0.95 | 9.93E-01 |
| Thigh subcutaneous AT | Insulin levels | MV | 15 | -0.548 | 0.164 | 8.24E-04 | 0.58 | 0.42 | 0.8 | 7.34E-02 |
| Thigh subcutaneous AT | Insulin response 30 mins | MV | 9 | -0.527 | 0.209 | 1.15E-02 | 0.59 | 0.39 | 0.89 | 8.60E-01 |
| Thigh subcutaneous fat | Insulin sensitivity index | MV | 17 | 0.374 | 0.072 | 2.05E-07 | 1.45 | 1.26 | 1.67 | 2.05E-07 |
| Thigh subcutaneous AT | Isoleucine | MV | 19 | -0.179 | 0.061 | 3.46E-03 | 0.84 | 0.74 | 0.94 | 2.91E-01 |
| Thigh subcutaneous AT | Leucine | MV | 19 | -0.2 | 0.065 | 2.13E-03 | 0.82 | 0.72 | 0.93 | 1.85E-01 |
| Thigh subcutaneous AT | MCP_3 | MV | 17 | 0.988 | 0.358 | 5.73E-03 | 2.69 | 1.33 | 5.41 | 4.58E-01 |
| Thigh subcutaneous AT | Monounsaturated fatty acids | MV | 19 | -0.347 | 0.143 | 1.54E-02 | 0.71 | 0.53 | 0.94 | 9.93E-01 |
| Thigh subcutaneous AT | Non-HDL cholesterol | MV | 19 | -0.471 | 0.171 | 6.05E-03 | 0.62 | 0.45 | 0.87 | 4.72E-01 |
| Thigh subcutaneous AT | Phenylalanine | MV | 19 | -0.125 | 0.039 | 1.41E-03 | 0.88 | 0.82 | 0.95 | 1.24E-01 |
| Thigh subcutaneous AT | PUFA/MUFA ratio | MV | 19 | 0.317 | 0.138 | 2.12E-02 | 1.37 | 1.05 | 1.8 | 9.93E-01 |
| Thigh subcutaneous AT | Saturated fatty acids | MV | 19 | -0.293 | 0.139 | 3.49E-02 | 0.75 | 0.57 | 0.98 | 9.93E-01 |
| Thigh subcutaneous AT | SHBG-female | MV | 18 | 0.332 | 0.085 | 9.09E-05 | 1.39 | 1.18 | 1.65 | 8.54E-03 |
| Thigh subcutaneous AT | SHBG-male | MV | 18 | 0.312 | 0.107 | 3.52E-03 | 1.37 | 1.11 | 1.68 | 2.92E-01 |
| Thigh subcutaneous AT | Total triglycerides | MV | 19 | -0.52 | 0.144 | 3.18E-04 | 0.59 | 0.45 | 0.79 | 2.89E-02 |
| Thigh subcutaneous AT | Tyrosine | MV | 19 | -0.137 | 0.064 | 3.27E-02 | 0.87 | 0.77 | 0.99 | 9.93E-01 |
| Thigh subcutaneous AT | Valine | MV | 19 | -0.271 | 0.071 | 1.31E-04 | 0.76 | 0.66 | 0.88 | 1.21E-02 |
| Thigh intermuscular AT | 3-Hydroxybutyrate | MV | 7 | 0.178 | 0.076 | 1.91E-02 | 1.2 | 1.03 | 1.39 | 9.93E-01 |
| Thigh intermuscular AT | Acetoacetate | MV | 7 | 0.119 | 0.057 | 3.76E-02 | 1.13 | 1.01 | 1.26 | 9.93E-01 |
| Thigh intermuscular AT | Adiponectin | MV | 7 | 0.247 | 0.097 | 1.08E-02 | 1.28 | 1.06 | 1.55 | 9.80E-01 |
| Thigh intermuscular AT | Alanine transaminase | MV | 7 | -0.032 | 0.013 | 1.52E-02 | 0.97 | 0.94 | 0.99 | 9.93E-01 |
| Thigh intermuscular AT | Corrected insulin response | MV | 3 | -0.646 | 0.202 | 1.41E-03 | 0.52 | 0.35 | 0.78 | 1.34E-01 |
| Thigh intermuscular AT | Fasting glucose | MV | 7 | -0.137 | 0.059 | 2.04E-02 | 0.87 | 0.78 | 0.98 | 9.93E-01 |
| Thigh intermuscular AT | Fasting insulin | MV | 7 | -0.195 | 0.064 | 2.29E-03 | 0.82 | 0.73 | 0.93 | 2.15E-01 |
| Thigh intermuscular AT | FGF_BASIC | MV | 7 | 0.38 | 0.182 | 3.69E-02 | 1.46 | 1.02 | 2.09 | 9.93E-01 |
| Thigh intermuscular AT | Glucose | MV | 7 | -0.168 | 0.067 | 1.25E-02 | 0.85 | 0.74 | 0.96 | 9.93E-01 |
| Thigh intermuscular AT | HOMA-IR | MV | 7 | -0.144 | 0.066 | 2.82E-02 | 0.87 | 0.76 | 0.98 | 9.93E-01 |
| Thigh intermuscular AT | Insulin levels | MV | 7 | -0.631 | 0.213 | 3.06E-03 | 0.53 | 0.35 | 0.81 | 2.85E-01 |
| Thigh intermuscular AT | Insulin release at 30-min increments | MV | 3 | -0.827 | 0.376 | 2.80E-02 | 0.44 | 0.21 | 0.91 | 9.93E-01 |
| Thigh intermuscular AT | Insulin response 30 mins | MV | 3 | -0.792 | 0.313 | 1.13E-02 | 0.45 | 0.25 | 0.84 | 9.93E-01 |
| Thigh intermuscular fat | Insulin sensitivity index | MV | 7 | 0.32 | 0.09 | 3.45E-04 | 1.38 | 1.16 | 1.64 | 3.45E-04 |
| Thigh intermuscular AT | TNF_B | MV | 7 | -1.118 | 0.402 | 5.46E-03 | 0.33 | 0.15 | 0.72 | 5.03E-01 |
| Visceral AT | Alanine transaminase | MV | 4 | 0.041 | 0.017 | 1.42E-02 | 1.04 | 1.01 | 1.08 | 9.97E-01 |
| Visceral AT | EOTAXIN | MV | 4 | 0.543 | 0.184 | 3.18E-03 | 1.72 | 1.2 | 2.47 | 3.05E-01 |
| Visceral AT | Fasting insulin | MV | 2 | 0.214 | 0.082 | 8.90E-03 | 1.24 | 1.06 | 1.45 | 8.28E-01 |
| Visceral AT | GROA | MV | 4 | 0.717 | 0.284 | 1.17E-02 | 2.05 | 1.17 | 3.58 | 9.97E-01 |
| Visceral AT | IL_1RA | MV | 4 | 0.765 | 0.342 | 2.53E-02 | 2.15 | 1.1 | 4.2 | 9.97E-01 |
| Visceral AT | IP_10 | MV | 4 | 0.817 | 0.298 | 6.09E-03 | 2.26 | 1.26 | 4.06 | 5.79E-01 |
| Visceral AT | Leptin | MV | 3 | -0.322 | 0.121 | 7.93E-03 | 0.72 | 0.57 | 0.92 | 7.45E-01 |
| Liver PDFF | Alanine transaminase | MV | 9 | 0.077 | 0.005 | 5.35E-58 | 1.08 | 1.07 | 1.09 | 5.14E-56 |
| Liver PDFF | ApoB/apoA1 ratio | MV | 9 | -0.131 | 0.062 | 3.44E-02 | 0.88 | 0.78 | 0.99 | 9.75E-01 |
| Liver PDFF | Apolipoprotein B | MV | 9 | -0.135 | 0.066 | 4.14E-02 | 0.87 | 0.77 | 0.99 | 9.75E-01 |
| Liver PDFF | Gamma glutamyl Transferase | MV | 9 | 0.029 | 0.008 | 2.62E-04 | 1.03 | 1.01 | 1.05 | 2.46E-02 |
| Liver PDFF | LDL cholesterol | MV | 9 | -0.179 | 0.065 | 6.17E-03 | 0.84 | 0.74 | 0.95 | 5.56E-01 |
| Liver PDFF | MCP_3 | MV | 8 | 0.364 | 0.158 | 2.14E-02 | 1.44 | 1.06 | 1.96 | 9.75E-01 |
| Liver PDFF | Non-HDL cholesterol | MV | 9 | -0.235 | 0.078 | 2.59E-03 | 0.79 | 0.68 | 0.92 | 2.41E-01 |
| Liver PDFF | Phenylalanine | MV | 9 | 0.05 | 0.018 | 6.08E-03 | 1.05 | 1.01 | 1.09 | 5.53E-01 |
| Liver PDFF | SHBG-male | MV | 9 | 0.119 | 0.049 | 1.54E-02 | 1.13 | 1.02 | 1.24 | 9.75E-01 |
| Liver PDFF | Total cholesterol | MV | 9 | -0.191 | 0.067 | 4.21E-03 | 0.83 | 0.72 | 0.94 | 3.88E-01 |
| Liver PDFF | Tyrosine | MV | 9 | 0.135 | 0.03 | 5.80E-06 | 1.14 | 1.08 | 1.21 | 5.51E-04 |
| Pancreas PDFF | Adiponectin | MV | 5 | 0.141 | 0.069 | 4.07E-02 | 1.15 | 1.01 | 1.32 | 9.98E-01 |
| Pancreas PDFF | Alkaline phosphatase | MV | 12 | -0.052 | 0.02 | 9.96E-03 | 0.95 | 0.91 | 0.99 | 9.46E-01 |
| Pancreas PDFF | Citrate | MV | 12 | 0.135 | 0.046 | 3.26E-03 | 1.14 | 1.05 | 1.25 | 3.13E-01 |
| Pancreas PDFF | Phenylalanine | MV | 12 | 0.07 | 0.03 | 1.95E-02 | 1.07 | 1.01 | 1.14 | 9.98E-01 |
| Paraspinal AT | Corrected insulin response | MV | 2 | 0.624 | 0.244 | 1.07E-02 | 1.87 | 1.16 | 3.01 | 9.90E-01 |
| Paraspinal AT | TNF_B | MV | 6 | 0.745 | 0.368 | 4.31E-02 | 2.11 | 1.02 | 4.34 | 9.90E-01 |
| Pelvic bone marrow fat | Corrected insulin response | MV | 8 | 0.398 | 0.176 | 2.41E-02 | 1.49 | 1.05 | 2.1 | 9.96E-01 |
| Pelvic bone marrow fat | Degree of unsaturation | MV | 25 | 0.526 | 0.246 | 3.26E-02 | 1.69 | 1.04 | 2.74 | 9.96E-01 |
| Pelvic bone marrow fat | PUFA/MUFA ratio | MV | 25 | 0.405 | 0.186 | 2.95E-02 | 1.5 | 1.04 | 2.16 | 9.96E-01 |
| Vertebrae bone marrow fat | MCP_3 | MV | 3 | 1 | 0.436 | 2.19E-02 | 2.72 | 1.16 | 6.4 | 9.91E-01 |
| Vertebrae bone marrow fat | Phenylalanine | MV | 4 | -0.093 | 0.045 | 4.11E-02 | 0.91 | 0.83 | 1 | 9.91E-01 |
| Vertebrae bone marrow fat | SHBG-female | MV | 4 | -0.25 | 0.097 | 9.49E-03 | 0.78 | 0.64 | 0.94 | 9.11E-01 |
| Vertebrae bone marrow fat | SHBG-male | MV | 4 | -0.245 | 0.121 | 4.33E-02 | 0.78 | 0.62 | 0.99 | 9.91E-01 |
| Vertebrae bone marrow fat | Tyrosine | MV | 4 | -0.153 | 0.074 | 3.89E-02 | 0.86 | 0.74 | 0.99 | 9.91E-01 |

**Supplementary table 9. The univariable Mendelian randomisation IVW result.** IVW results for the effect of IDPs on the risk of the 26 disease outcomes meta-analysed using FinnGen and publicly available genome wide association studies. SE: standard error, BHP: Benjamini-Hochberg corrected p-value, OR: odds ratio and 95% confidence intervals.

| **Exposure** | **Outcome** | **Beta** | **SE** | **P-value** | **OR** | **OR_lci95** | **OR_uci95** | **BHP** |
| --- | --- | --- | --- | --- | --- | --- | --- | --- |
| Abdominal subcutaneous AT | Type 2 diabetes | 1.297 | 0.573 | 2.4E-02 | 3.7 | 1.2 | 11.2 | 4.0E-01 |
| Abdominal subcutaneous AT | Polycystic ovary syndrome | 0.531 | 0.264 | 4.4E-02 | 1.7 | 1.0 | 2.9 | 5.7E-01 |
| Abdominal subcutaneous AT | MASLD | 0.931 | 0.217 | 1.8E-05 | 2.5 | 1.7 | 3.9 | 4.2E-04 |
| Abdominal subcutaneous AT | Chronic kidney disease | 0.240 | 0.141 | 8.8E-02 | 1.3 | 1.0 | 1.7 | 9.6E-01 |
| Abdominal subcutaneous AT | Hypertension | 0.009 | 0.030 | 7.7E-01 | 1.0 | 1.0 | 1.1 | 9.6E-01 |
| Abdominal subcutaneous AT | Coronary artery disease | 0.291 | 0.144 | 4.3E-02 | 1.3 | 1.0 | 1.8 | 5.7E-01 |
| Abdominal subcutaneous AT | Stroke | 0.212 | 0.105 | 4.3E-02 | 1.2 | 1.0 | 1.5 | 5.7E-01 |
| Abdominal subcutaneous AT | Myocardial infarction | -0.043 | 0.202 | 8.3E-01 | 1.0 | 0.6 | 1.4 | 9.6E-01 |
| Abdominal subcutaneous AT | Aortic aneurysm | -0.046 | 0.174 | 7.9E-01 | 1.0 | 0.7 | 1.3 | 9.6E-01 |
| Abdominal subcutaneous AT | Heart failure | 0.477 | 0.109 | 1.3E-05 | 1.6 | 1.3 | 2.0 | 3.0E-04 |
| Abdominal subcutaneous AT | Atrial fibrillation | 0.429 | 0.190 | 2.4E-02 | 1.5 | 1.1 | 2.2 | 4.0E-01 |
| Abdominal subcutaneous AT | Peripheral artery disease | 0.236 | 0.222 | 2.9E-01 | 1.3 | 0.8 | 2.0 | 9.6E-01 |
| Abdominal subcutaneous AT | Deep vein thrombosis | 0.002 | 0.004 | 6.5E-01 | 1.0 | 1.0 | 1.0 | 9.6E-01 |
| Abdominal subcutaneous AT | Pulmonary embolism | 0.192 | 0.162 | 2.3E-01 | 1.2 | 0.9 | 1.7 | 9.6E-01 |
| Abdominal subcutaneous AT | Gout | 0.622 | 0.224 | 5.6E-03 | 1.9 | 1.2 | 2.9 | 1.1E-01 |
| Abdominal subcutaneous AT | Knee osteoarthritis | 0.620 | 0.128 | 1.3E-06 | 1.9 | 1.4 | 2.4 | 3.4E-05 |
| Abdominal subcutaneous AT | Hip osteoarthritis | 0.734 | 0.197 | 2.0E-04 | 2.1 | 1.4 | 3.1 | 4.2E-03 |
| Abdominal subcutaneous AT | Rheumatoid arthritis | -0.312 | 0.409 | 4.5E-01 | 0.7 | 0.3 | 1.6 | 9.6E-01 |
| Abdominal subcutaneous AT | Osteoporosis | -0.005 | 0.003 | 1.8E-01 | 1.0 | 1.0 | 1.0 | 9.6E-01 |
| Abdominal subcutaneous AT | Asthma | 0.251 | 0.066 | 1.4E-04 | 1.3 | 1.1 | 1.5 | 3.0E-03 |
| Abdominal subcutaneous AT | Psoriasis | -0.022 | 0.180 | 9.0E-01 | 1.0 | 0.7 | 1.4 | 9.6E-01 |
| Abdominal subcutaneous AT | Depression | 0.082 | 0.151 | 5.9E-01 | 1.1 | 0.8 | 1.5 | 9.6E-01 |
| Abdominal subcutaneous AT | Parkinson's disease | 0.011 | 0.226 | 9.6E-01 | 1.0 | 0.6 | 1.6 | 9.6E-01 |
| Abdominal subcutaneous AT | Alzheimer’s disease | -0.298 | 0.135 | 2.8E-02 | 0.7 | 0.6 | 1.0 | 4.4E-01 |
| Abdominal subcutaneous AT | Cholelithiasis | 0.574 | 0.245 | 1.9E-02 | 1.8 | 1.1 | 2.9 | 3.6E-01 |
| Abdominal subcutaneous AT | Gastroesophageal reflux disease | 0.447 | 0.059 | 5.5E-14 | 1.6 | 1.4 | 1.8 | 1.4E-12 |
| Thigh subcutaneous AT | Type 2 diabetes | -0.468 | 0.146 | 1.3E-03 | 0.6 | 0.5 | 0.8 | 2.8E-02 |
| Thigh subcutaneous AT | Polycystic ovary syndrome | -0.245 | 0.115 | 3.3E-02 | 0.8 | 0.6 | 1.0 | 5.6E-01 |
| Thigh subcutaneous AT | MASLD | -0.245 | 0.123 | 4.7E-02 | 0.8 | 0.6 | 1.0 | 7.6E-01 |
| Thigh subcutaneous AT | Chronic kidney disease | -0.071 | 0.060 | 2.3E-01 | 0.9 | 0.8 | 1.0 | 9.8E-01 |
| Thigh subcutaneous AT | Hypertension | -0.075 | 0.013 | 2.1E-08 | 0.9 | 0.9 | 1.0 | 5.5E-07 |
| Thigh subcutaneous AT | Coronary artery disease | -0.271 | 0.066 | 4.6E-05 | 0.8 | 0.7 | 0.9 | 1.1E-03 |
| Thigh subcutaneous AT | Stroke | -0.080 | 0.059 | 1.7E-01 | 0.9 | 0.8 | 1.0 | 9.8E-01 |
| Thigh subcutaneous AT | Myocardial infarction | -0.303 | 0.065 | 2.8E-06 | 0.7 | 0.7 | 0.8 | 7.1E-05 |
| Thigh subcutaneous AT | Aortic aneurysm | -0.341 | 0.091 | 1.8E-04 | 0.7 | 0.6 | 0.8 | 4.0E-03 |
| Thigh subcutaneous AT | Heart failure | 0.054 | 0.056 | 3.3E-01 | 1.1 | 0.9 | 1.2 | 9.8E-01 |
| Thigh subcutaneous AT | Atrial fibrillation | 0.081 | 0.067 | 2.2E-01 | 1.1 | 1.0 | 1.2 | 9.8E-01 |
| Thigh subcutaneous AT | Peripheral artery disease | -0.214 | 0.068 | 1.6E-03 | 0.8 | 0.7 | 0.9 | 3.2E-02 |
| Thigh subcutaneous AT | Deep vein thrombosis | 0.001 | 0.002 | 6.7E-01 | 1.0 | 1.0 | 1.0 | 9.8E-01 |
| Thigh subcutaneous AT | Pulmonary embolism | 0.232 | 0.088 | 8.6E-03 | 1.3 | 1.1 | 1.5 | 1.6E-01 |
| Thigh subcutaneous AT | Gout | -0.225 | 0.122 | 6.6E-02 | 0.8 | 0.6 | 1.0 | 9.8E-01 |
| Thigh subcutaneous AT | Knee osteoarthritis | 0.285 | 0.066 | 1.4E-05 | 1.3 | 1.2 | 1.5 | 3.4E-04 |
| Thigh subcutaneous AT | Hip osteoarthritis | -0.002 | 0.078 | 9.8E-01 | 1.0 | 0.9 | 1.2 | 9.8E-01 |
| Thigh subcutaneous AT | Rheumatoid arthritis | 0.071 | 0.078 | 3.6E-01 | 1.1 | 0.9 | 1.2 | 9.8E-01 |
| Thigh subcutaneous AT | Osteoporosis | 0.006 | 0.002 | 2.5E-03 | 1.0 | 1.0 | 1.0 | 4.8E-02 |
| Thigh subcutaneous AT | Asthma | 0.063 | 0.043 | 1.5E-01 | 1.1 | 1.0 | 1.2 | 9.8E-01 |
| Thigh subcutaneous AT | Psoriasis | -0.076 | 0.072 | 2.9E-01 | 0.9 | 0.8 | 1.1 | 9.8E-01 |
| Thigh subcutaneous AT | Depression | 0.065 | 0.036 | 7.2E-02 | 1.1 | 1.0 | 1.1 | 9.8E-01 |
| Thigh subcutaneous AT | Parkinson's disease | -0.009 | 0.095 | 9.2E-01 | 1.0 | 0.8 | 1.2 | 9.8E-01 |
| Thigh subcutaneous AT | Alzheimer’s disease | -0.006 | 0.040 | 8.9E-01 | 1.0 | 0.9 | 1.1 | 9.8E-01 |
| Thigh subcutaneous AT | Cholelithiasis | 0.043 | 0.053 | 4.2E-01 | 1.0 | 0.9 | 1.2 | 9.8E-01 |
| Thigh subcutaneous AT | Gastroesophageal reflux disease | 0.073 | 0.049 | 1.4E-01 | 1.1 | 1.0 | 1.2 | 9.8E-01 |
| Thigh intermuscular AT | Type 2 diabetes | -0.619 | 0.093 | 2.7E-11 | 0.5 | 0.4 | 0.6 | 7.1E-10 |
| Thigh intermuscular AT | Polycystic ovary syndrome | -0.501 | 0.147 | 6.7E-04 | 0.6 | 0.5 | 0.8 | 1.4E-02 |
| Thigh intermuscular AT | MASLD | -0.465 | 0.084 | 2.8E-08 | 0.6 | 0.5 | 0.7 | 6.5E-07 |
| Thigh intermuscular AT | Chronic kidney disease | -0.091 | 0.064 | 1.5E-01 | 0.9 | 0.8 | 1.0 | 9.8E-01 |
| Thigh intermuscular AT | Hypertension | -0.065 | 0.011 | 3.7E-09 | 0.9 | 0.9 | 1.0 | 9.1E-08 |
| Thigh intermuscular AT | Coronary artery disease | -0.284 | 0.050 | 1.1E-08 | 0.8 | 0.7 | 0.8 | 2.7E-07 |
| Thigh intermuscular AT | Stroke | -0.119 | 0.062 | 5.4E-02 | 0.9 | 0.8 | 1.0 | 9.6E-01 |
| Thigh intermuscular AT | Myocardial infarction | -0.215 | 0.055 | 1.0E-04 | 0.8 | 0.7 | 0.9 | 2.2E-03 |
| Thigh intermuscular AT | Aortic aneurysm | -0.138 | 0.087 | 1.1E-01 | 0.9 | 0.7 | 1.0 | 9.8E-01 |
| Thigh intermuscular AT | Heart failure | -0.055 | 0.036 | 1.3E-01 | 0.9 | 0.9 | 1.0 | 9.8E-01 |
| Thigh intermuscular AT | Atrial fibrillation | -0.011 | 0.049 | 8.2E-01 | 1.0 | 0.9 | 1.1 | 9.8E-01 |
| Thigh intermuscular AT | Peripheral artery disease | -0.181 | 0.076 | 1.8E-02 | 0.8 | 0.7 | 1.0 | 3.6E-01 |
| Thigh intermuscular AT | Deep vein thrombosis | 0.000 | 0.002 | 9.3E-01 | 1.0 | 1.0 | 1.0 | 9.8E-01 |
| Thigh intermuscular AT | Pulmonary embolism | 0.164 | 0.087 | 6.0E-02 | 1.2 | 1.0 | 1.4 | 9.8E-01 |
| Thigh intermuscular AT | Gout | -0.157 | 0.153 | 3.0E-01 | 0.9 | 0.6 | 1.2 | 9.8E-01 |
| Thigh intermuscular AT | Knee osteoarthritis | 0.030 | 0.059 | 6.1E-01 | 1.0 | 0.9 | 1.2 | 9.8E-01 |
| Thigh intermuscular AT | Hip osteoarthritis | -0.065 | 0.058 | 2.6E-01 | 0.9 | 0.8 | 1.1 | 9.8E-01 |
| Thigh intermuscular AT | Rheumatoid arthritis | 0.057 | 0.075 | 4.5E-01 | 1.1 | 0.9 | 1.2 | 9.8E-01 |
| Thigh intermuscular AT | Osteoporosis | 0.003 | 0.002 | 2.0E-01 | 1.0 | 1.0 | 1.0 | 9.8E-01 |
| Thigh intermuscular AT | Asthma | 0.083 | 0.036 | 2.0E-02 | 1.1 | 1.0 | 1.2 | 3.8E-01 |
| Thigh intermuscular AT | Psoriasis | 0.025 | 0.069 | 7.1E-01 | 1.0 | 0.9 | 1.2 | 9.8E-01 |
| Thigh intermuscular AT | Depression | -0.004 | 0.038 | 9.1E-01 | 1.0 | 0.9 | 1.1 | 9.8E-01 |
| Thigh intermuscular AT | Parkinson's disease | -0.054 | 0.088 | 5.4E-01 | 0.9 | 0.8 | 1.1 | 9.8E-01 |
| Thigh intermuscular AT | Alzheimer’s disease | -0.051 | 0.046 | 2.7E-01 | 1.0 | 0.9 | 1.0 | 9.8E-01 |
| Thigh intermuscular AT | Cholelithiasis | 0.001 | 0.044 | 9.8E-01 | 1.0 | 0.9 | 1.1 | 9.8E-01 |
| Thigh intermuscular AT | Gastroesophageal reflux disease | -0.015 | 0.040 | 7.1E-01 | 1.0 | 0.9 | 1.1 | 9.8E-01 |
| Visceral AT | Type 2 diabetes | 0.229 | 0.394 | 5.6E-01 | 1.3 | 0.6 | 2.7 | 1.0E+00 |
| Visceral AT | Polycystic ovary syndrome | 0.217 | 0.233 | 3.5E-01 | 1.2 | 0.8 | 2.0 | 1.0E+00 |
| Visceral AT | MASLD | 0.137 | 0.283 | 6.3E-01 | 1.1 | 0.7 | 2.0 | 1.0E+00 |
| Visceral AT | Chronic kidney disease | -0.022 | 0.139 | 8.8E-01 | 1.0 | 0.7 | 1.3 | 1.0E+00 |
| Visceral AT | Hypertension | 0.005 | 0.035 | 8.9E-01 | 1.0 | 0.9 | 1.1 | 1.0E+00 |
| Visceral AT | Coronary artery disease | -0.039 | 0.152 | 8.0E-01 | 1.0 | 0.7 | 1.3 | 1.0E+00 |
| Visceral AT | Stroke | 0.117 | 0.110 | 2.9E-01 | 1.1 | 0.9 | 1.4 | 1.0E+00 |
| Visceral AT | Myocardial infarction | -0.153 | 0.127 | 2.3E-01 | 0.9 | 0.7 | 1.1 | 1.0E+00 |
| Visceral AT | Aortic aneurysm | -0.136 | 0.160 | 3.9E-01 | 0.9 | 0.6 | 1.2 | 1.0E+00 |
| Visceral AT | Heart failure | 0.153 | 0.118 | 2.0E-01 | 1.2 | 0.9 | 1.5 | 1.0E+00 |
| Visceral AT | Atrial fibrillation | 0.000 | 0.139 | 1.0E+00 | 1.0 | 0.8 | 1.3 | 1.0E+00 |
| Visceral AT | Peripheral artery disease | 0.094 | 0.136 | 4.9E-01 | 1.1 | 0.8 | 1.4 | 1.0E+00 |
| Visceral AT | Deep vein thrombosis | 0.005 | 0.003 | 5.8E-02 | 1.0 | 1.0 | 1.0 | 1.0E+00 |
| Visceral AT | Pulmonary embolism | 0.292 | 0.171 | 8.7E-02 | 1.3 | 1.0 | 1.9 | 1.0E+00 |
| Visceral AT | Gout | -0.002 | 0.020 | 9.1E-01 | 1.0 | 1.0 | 1.0 | 1.0E+00 |
| Visceral AT | Knee osteoarthritis | 0.328 | 0.164 | 4.6E-02 | 1.4 | 1.0 | 1.9 | 1.0E+00 |
| Visceral AT | Hip osteoarthritis | 0.305 | 0.173 | 7.7E-02 | 1.4 | 1.0 | 1.9 | 1.0E+00 |
| Visceral AT | Rheumatoid arthritis | -0.107 | 0.205 | 6.0E-01 | 0.9 | 0.6 | 1.3 | 1.0E+00 |
| Visceral AT | Osteoporosis | -0.001 | 0.004 | 7.3E-01 | 1.0 | 1.0 | 1.0 | 1.0E+00 |
| Visceral AT | Asthma | 0.267 | 0.055 | 1.5E-06 | 1.3 | 1.2 | 1.5 | 3.8E-05 |
| Visceral AT | Psoriasis | 0.143 | 0.139 | 3.0E-01 | 1.2 | 0.9 | 1.5 | 1.0E+00 |
| Visceral AT | Depression | 0.152 | 0.102 | 1.4E-01 | 1.2 | 1.0 | 1.4 | 1.0E+00 |
| Visceral AT | Parkinson's disease | -0.004 | 0.165 | 9.8E-01 | 1.0 | 0.7 | 1.4 | 1.0E+00 |
| Visceral AT | Alzheimer’s disease | -0.131 | 0.109 | 2.3E-01 | 0.9 | 0.7 | 1.1 | 1.0E+00 |
| Visceral AT | Cholelithiasis | 0.401 | 0.119 | 7.3E-04 | 1.5 | 1.2 | 1.9 | 1.8E-02 |
| Visceral AT | Gastroesophageal reflux disease | 0.186 | 0.095 | 5.1E-02 | 1.2 | 1.0 | 1.5 | 1.0E+00 |
| Liver PDFF | Type 2 diabetes | 0.194 | 0.077 | 1.2E-02 | 1.2 | 1.0 | 1.4 | 2.6E-01 |
| Liver PDFF | Polycystic ovary syndrome | 0.047 | 0.076 | 5.4E-01 | 1.0 | 0.9 | 1.2 | 9.5E-01 |
| Liver PDFF | MASLD | 1.334 | 0.077 | 8.9E-67 | 3.8 | 3.3 | 4.4 | 2.3E-65 |
| Liver PDFF | Chronic kidney disease | -0.024 | 0.063 | 7.1E-01 | 1.0 | 0.9 | 1.1 | 9.5E-01 |
| Liver PDFF | Hypertension | 0.012 | 0.009 | 1.8E-01 | 1.0 | 1.0 | 1.0 | 9.5E-01 |
| Liver PDFF | Coronary artery disease | -0.093 | 0.071 | 1.9E-01 | 0.9 | 0.8 | 1.0 | 9.5E-01 |
| Liver PDFF | Stroke | -0.042 | 0.029 | 1.4E-01 | 1.0 | 0.9 | 1.0 | 9.5E-01 |
| Liver PDFF | Myocardial infarction | -0.205 | 0.073 | 5.1E-03 | 0.8 | 0.7 | 0.9 | 1.2E-01 |
| Liver PDFF | Aortic aneurysm | -0.035 | 0.071 | 6.2E-01 | 1.0 | 0.8 | 1.1 | 9.5E-01 |
| Liver PDFF | Heart failure | -0.014 | 0.039 | 7.3E-01 | 1.0 | 0.9 | 1.1 | 9.5E-01 |
| Liver PDFF | Atrial fibrillation | -0.038 | 0.022 | 8.1E-02 | 1.0 | 0.9 | 1.0 | 9.5E-01 |
| Liver PDFF | Peripheral artery disease | -0.144 | 0.044 | 1.0E-03 | 0.9 | 0.8 | 0.9 | 2.5E-02 |
| Liver PDFF | Deep vein thrombosis | -0.002 | 0.001 | 6.8E-02 | 1.0 | 1.0 | 1.0 | 9.5E-01 |
| Liver PDFF | Pulmonary embolism | -0.066 | 0.073 | 3.7E-01 | 0.9 | 0.8 | 1.1 | 9.5E-01 |
| Liver PDFF | Gout | -0.109 | 0.190 | 5.7E-01 | 0.9 | 0.6 | 1.3 | 9.5E-01 |
| Liver PDFF | Knee osteoarthritis | 0.020 | 0.037 | 5.9E-01 | 1.0 | 0.9 | 1.1 | 9.5E-01 |
| Liver PDFF | Hip osteoarthritis | 0.003 | 0.042 | 9.5E-01 | 1.0 | 0.9 | 1.1 | 9.5E-01 |
| Liver PDFF | Rheumatoid arthritis | 0.013 | 0.032 | 6.8E-01 | 1.0 | 1.0 | 1.1 | 9.5E-01 |
| Liver PDFF | Osteoporosis | 0.003 | 0.001 | 2.7E-03 | 1.0 | 1.0 | 1.0 | 6.4E-02 |
| Liver PDFF | Asthma | 0.004 | 0.033 | 9.1E-01 | 1.0 | 0.9 | 1.1 | 9.5E-01 |
| Liver PDFF | Psoriasis | 0.117 | 0.048 | 1.5E-02 | 1.1 | 1.0 | 1.2 | 3.2E-01 |
| Liver PDFF | Depression | -0.025 | 0.027 | 3.7E-01 | 1.0 | 0.9 | 1.0 | 9.5E-01 |
| Liver PDFF | Parkinson's disease | -0.120 | 0.060 | 4.6E-02 | 0.9 | 0.8 | 1.0 | 9.2E-01 |
| Liver PDFF | Alzheimer’s disease | 0.010 | 0.049 | 8.3E-01 | 1.0 | 0.9 | 1.1 | 9.5E-01 |
| Liver PDFF | Cholelithiasis | -0.146 | 0.081 | 7.3E-02 | 0.9 | 0.7 | 1.0 | 9.5E-01 |
| Liver PDFF | Gastroesophageal reflux disease | -0.017 | 0.022 | 4.2E-01 | 1.0 | 0.9 | 1.0 | 9.5E-01 |
| Pancreas PDFF | Type 2 diabetes | -0.116 | 0.100 | 2.5E-01 | 0.9 | 0.7 | 1.1 | 9.4E-01 |
| Pancreas PDFF | Polycystic ovary syndrome | 0.011 | 0.129 | 9.3E-01 | 1.0 | 0.8 | 1.3 | 9.4E-01 |
| Pancreas PDFF | MASLD | 0.010 | 0.098 | 9.2E-01 | 1.0 | 0.8 | 1.2 | 9.4E-01 |
| Pancreas PDFF | Chronic kidney disease | -0.084 | 0.059 | 1.6E-01 | 0.9 | 0.8 | 1.0 | 9.4E-01 |
| Pancreas PDFF | Hypertension | -0.011 | 0.016 | 4.7E-01 | 1.0 | 1.0 | 1.0 | 9.4E-01 |
| Pancreas PDFF | Coronary artery disease | -0.088 | 0.062 | 1.6E-01 | 0.9 | 0.8 | 1.0 | 9.4E-01 |
| Pancreas PDFF | Stroke | 0.079 | 0.052 | 1.3E-01 | 1.1 | 1.0 | 1.2 | 9.4E-01 |
| Pancreas PDFF | Myocardial infarction | 0.053 | 0.074 | 4.7E-01 | 1.1 | 0.9 | 1.2 | 9.4E-01 |
| Pancreas PDFF | Aortic aneurysm | 0.054 | 0.074 | 4.7E-01 | 1.1 | 0.9 | 1.2 | 9.4E-01 |
| Pancreas PDFF | Heart failure | 0.076 | 0.063 | 2.3E-01 | 1.1 | 1.0 | 1.2 | 9.4E-01 |
| Pancreas PDFF | Atrial fibrillation | 0.119 | 0.050 | 1.8E-02 | 1.1 | 1.0 | 1.2 | 4.6E-01 |
| Pancreas PDFF | Peripheral artery disease | -0.055 | 0.083 | 5.1E-01 | 0.9 | 0.8 | 1.1 | 9.4E-01 |
| Pancreas PDFF | Deep vein thrombosis | 0.011 | 0.008 | 1.6E-01 | 1.0 | 1.0 | 1.0 | 9.4E-01 |
| Pancreas PDFF | Pulmonary embolism | 0.577 | 0.256 | 2.4E-02 | 1.8 | 1.1 | 2.9 | 6.0E-01 |
| Pancreas PDFF | Gout | -0.215 | 0.122 | 7.8E-02 | 0.8 | 0.6 | 1.0 | 9.4E-01 |
| Pancreas PDFF | Knee osteoarthritis | 0.060 | 0.044 | 1.8E-01 | 1.1 | 1.0 | 1.2 | 9.4E-01 |
| Pancreas PDFF | Hip osteoarthritis | -0.087 | 0.054 | 1.1E-01 | 0.9 | 0.8 | 1.0 | 9.4E-01 |
| Pancreas PDFF | Rheumatoid arthritis | -0.061 | 0.086 | 4.8E-01 | 0.9 | 0.8 | 1.1 | 9.4E-01 |
| Pancreas PDFF | Osteoporosis | -0.001 | 0.001 | 5.2E-01 | 1.0 | 1.0 | 1.0 | 9.4E-01 |
| Pancreas PDFF | Asthma | 0.068 | 0.050 | 1.8E-01 | 1.1 | 1.0 | 1.2 | 9.4E-01 |
| Pancreas PDFF | Psoriasis | 0.013 | 0.104 | 9.0E-01 | 1.0 | 0.8 | 1.2 | 9.4E-01 |
| Pancreas PDFF | Depression | -0.023 | 0.044 | 6.0E-01 | 1.0 | 0.9 | 1.1 | 9.4E-01 |
| Pancreas PDFF | Parkinson's disease | -0.181 | 0.091 | 4.7E-02 | 0.8 | 0.7 | 1.0 | 9.4E-01 |
| Pancreas PDFF | Alzheimer’s disease | -0.003 | 0.047 | 9.4E-01 | 1.0 | 0.9 | 1.1 | 9.4E-01 |
| Pancreas PDFF | Cholelithiasis | 0.184 | 0.091 | 4.3E-02 | 1.2 | 1.0 | 1.4 | 9.4E-01 |
| Pancreas PDFF | Gastroesophageal reflux disease | 0.007 | 0.041 | 8.7E-01 | 1.0 | 0.9 | 1.1 | 9.4E-01 |
| Paraspinal AT | Type 2 diabetes | -0.094 | 0.227 | 6.8E-01 | 0.9 | 0.6 | 1.4 | 9.6E-01 |
| Paraspinal AT | Polycystic ovary syndrome | -0.278 | 0.184 | 1.3E-01 | 0.8 | 0.5 | 1.1 | 9.6E-01 |
| Paraspinal AT | MASLD | -0.119 | 0.163 | 4.7E-01 | 0.9 | 0.6 | 1.2 | 9.6E-01 |
| Paraspinal AT | Chronic kidney disease | -0.034 | 0.073 | 6.4E-01 | 1.0 | 0.8 | 1.1 | 9.6E-01 |
| Paraspinal AT | Hypertension | -0.011 | 0.014 | 4.3E-01 | 1.0 | 1.0 | 1.0 | 9.6E-01 |
| Paraspinal AT | Coronary artery disease | -0.118 | 0.070 | 9.2E-02 | 0.9 | 0.8 | 1.0 | 9.6E-01 |
| Paraspinal AT | Stroke | -0.007 | 0.065 | 9.2E-01 | 1.0 | 0.9 | 1.1 | 9.6E-01 |
| Paraspinal AT | Myocardial infarction | -0.131 | 0.049 | 7.3E-03 | 0.9 | 0.8 | 1.0 | 1.9E-01 |
| Paraspinal AT | Aortic aneurysm | -0.010 | 0.087 | 9.1E-01 | 1.0 | 0.8 | 1.2 | 9.6E-01 |
| Paraspinal AT | Heart failure | -0.039 | 0.071 | 5.8E-01 | 1.0 | 0.8 | 1.1 | 9.6E-01 |
| Paraspinal AT | Atrial fibrillation | 0.155 | 0.064 | 1.6E-02 | 1.2 | 1.0 | 1.3 | 4.0E-01 |
| Paraspinal AT | Peripheral artery disease | -0.089 | 0.094 | 3.4E-01 | 0.9 | 0.8 | 1.1 | 9.6E-01 |
| Paraspinal AT | Deep vein thrombosis | 0.002 | 0.003 | 5.7E-01 | 1.0 | 1.0 | 1.0 | 9.6E-01 |
| Paraspinal AT | Pulmonary embolism | 0.167 | 0.078 | 3.2E-02 | 1.2 | 1.0 | 1.4 | 7.8E-01 |
| Paraspinal AT | Gout | -0.141 | 0.163 | 3.9E-01 | 0.9 | 0.6 | 1.2 | 9.6E-01 |
| Paraspinal AT | Knee osteoarthritis | 0.093 | 0.091 | 3.1E-01 | 1.1 | 0.9 | 1.3 | 9.6E-01 |
| Paraspinal AT | Hip osteoarthritis | 0.175 | 0.094 | 6.5E-02 | 1.2 | 1.0 | 1.4 | 9.6E-01 |
| Paraspinal AT | Rheumatoid arthritis | -0.030 | 0.066 | 6.5E-01 | 1.0 | 0.9 | 1.1 | 9.6E-01 |
| Paraspinal AT | Osteoporosis | 0.000 | 0.002 | 9.6E-01 | 1.0 | 1.0 | 1.0 | 9.6E-01 |
| Paraspinal AT | Asthma | 0.013 | 0.038 | 7.4E-01 | 1.0 | 0.9 | 1.1 | 9.6E-01 |
| Paraspinal AT | Psoriasis | 0.004 | 0.081 | 9.6E-01 | 1.0 | 0.9 | 1.2 | 9.6E-01 |
| Paraspinal AT | Depression | -0.004 | 0.045 | 9.3E-01 | 1.0 | 0.9 | 1.1 | 9.6E-01 |
| Paraspinal AT | Parkinson's disease | -0.017 | 0.091 | 8.6E-01 | 1.0 | 0.8 | 1.2 | 9.6E-01 |
| Paraspinal AT | Alzheimer’s disease | -0.074 | 0.049 | 1.4E-01 | 0.9 | 0.8 | 1.0 | 9.6E-01 |
| Paraspinal AT | Cholelithiasis | 0.081 | 0.075 | 2.8E-01 | 1.1 | 0.9 | 1.3 | 9.6E-01 |
| Paraspinal AT | Gastroesophageal reflux disease | 0.035 | 0.058 | 5.4E-01 | 1.0 | 0.9 | 1.2 | 9.6E-01 |
| Pelvic bone marrow fat | Type 2 diabetes | -0.026 | 0.043 | 5.5E-01 | 1.0 | 0.9 | 1.1 | 9.5E-01 |
| Pelvic bone marrow fat | Polycystic ovary syndrome | 0.120 | 0.098 | 2.2E-01 | 1.1 | 0.9 | 1.4 | 9.5E-01 |
| Pelvic bone marrow fat | MASLD | -0.067 | 0.058 | 2.5E-01 | 0.9 | 0.8 | 1.0 | 9.5E-01 |
| Pelvic bone marrow fat | Chronic kidney disease | -0.047 | 0.038 | 2.2E-01 | 1.0 | 0.9 | 1.0 | 9.5E-01 |
| Pelvic bone marrow fat | Hypertension | -0.006 | 0.011 | 5.6E-01 | 1.0 | 1.0 | 1.0 | 9.5E-01 |
| Pelvic bone marrow fat | Coronary artery disease | -0.049 | 0.050 | 3.3E-01 | 1.0 | 0.9 | 1.0 | 9.5E-01 |
| Pelvic bone marrow fat | Stroke | -0.050 | 0.044 | 2.5E-01 | 1.0 | 0.9 | 1.0 | 9.5E-01 |
| Pelvic bone marrow fat | Myocardial infarction | -0.021 | 0.054 | 7.0E-01 | 1.0 | 0.9 | 1.1 | 9.5E-01 |
| Pelvic bone marrow fat | Aortic aneurysm | -0.039 | 0.060 | 5.1E-01 | 1.0 | 0.9 | 1.1 | 9.5E-01 |
| Pelvic bone marrow fat | Heart failure | -0.067 | 0.034 | 4.6E-02 | 0.9 | 0.9 | 1.0 | 9.2E-01 |
| Pelvic bone marrow fat | Atrial fibrillation | -0.028 | 0.040 | 4.8E-01 | 1.0 | 0.9 | 1.1 | 9.5E-01 |
| Pelvic bone marrow fat | Peripheral artery disease | 0.016 | 0.054 | 7.6E-01 | 1.0 | 0.9 | 1.1 | 9.5E-01 |
| Pelvic bone marrow fat | Deep vein thrombosis | -0.001 | 0.002 | 5.1E-01 | 1.0 | 1.0 | 1.0 | 9.5E-01 |
| Pelvic bone marrow fat | Pulmonary embolism | 0.035 | 0.059 | 5.5E-01 | 1.0 | 0.9 | 1.2 | 9.5E-01 |
| Pelvic bone marrow fat | Gout | -0.203 | 0.073 | 5.2E-03 | 0.8 | 0.7 | 0.9 | 1.3E-01 |
| Pelvic bone marrow fat | Knee osteoarthritis | -0.139 | 0.049 | 4.6E-03 | 0.9 | 0.8 | 1.0 | 1.2E-01 |
| Pelvic bone marrow fat | Hip osteoarthritis | -0.111 | 0.046 | 1.5E-02 | 0.9 | 0.8 | 1.0 | 3.3E-01 |
| Pelvic bone marrow fat | Rheumatoid arthritis | -0.170 | 0.079 | 3.1E-02 | 0.8 | 0.7 | 1.0 | 6.5E-01 |
| Pelvic bone marrow fat | Osteoporosis | 0.007 | 0.003 | 9.7E-03 | 1.0 | 1.0 | 1.0 | 2.2E-01 |
| Pelvic bone marrow fat | Asthma | -0.036 | 0.041 | 3.8E-01 | 1.0 | 0.9 | 1.0 | 9.5E-01 |
| Pelvic bone marrow fat | Psoriasis | -0.105 | 0.091 | 2.5E-01 | 0.9 | 0.8 | 1.1 | 9.5E-01 |
| Pelvic bone marrow fat | Depression | -0.046 | 0.029 | 1.2E-01 | 1.0 | 0.9 | 1.0 | 9.5E-01 |
| Pelvic bone marrow fat | Parkinson's disease | -0.063 | 0.054 | 2.4E-01 | 0.9 | 0.8 | 1.0 | 9.5E-01 |
| Pelvic bone marrow fat | Alzheimer’s disease | 0.051 | 0.037 | 1.6E-01 | 1.1 | 1.0 | 1.1 | 9.5E-01 |
| Pelvic bone marrow fat | Cholelithiasis | -0.094 | 0.035 | 7.5E-03 | 0.9 | 0.8 | 1.0 | 1.8E-01 |
| Pelvic bone marrow fat | Gastroesophageal reflux disease | -0.002 | 0.027 | 9.5E-01 | 1.0 | 0.9 | 1.1 | 9.5E-01 |
| Thigh bone marrow fat | Type 2 diabetes | -0.159 | 0.088 | 7.0E-02 | 0.9 | 0.7 | 1.0 | 9.5E-01 |
| Thigh bone marrow fat | Polycystic ovary syndrome | -0.197 | 0.103 | 5.5E-02 | 0.8 | 0.7 | 1.0 | 9.5E-01 |
| Thigh bone marrow fat | MASLD | -0.103 | 0.077 | 1.8E-01 | 0.9 | 0.8 | 1.0 | 9.5E-01 |
| Thigh bone marrow fat | Chronic kidney disease | 0.054 | 0.037 | 1.5E-01 | 1.1 | 1.0 | 1.1 | 9.5E-01 |
| Thigh bone marrow fat | Hypertension | 0.005 | 0.009 | 5.6E-01 | 1.0 | 1.0 | 1.0 | 9.5E-01 |
| Thigh bone marrow fat | Coronary artery disease | -0.012 | 0.041 | 7.7E-01 | 1.0 | 0.9 | 1.1 | 9.5E-01 |
| Thigh bone marrow fat | Stroke | -0.004 | 0.035 | 9.2E-01 | 1.0 | 0.9 | 1.1 | 9.5E-01 |
| Thigh bone marrow fat | Myocardial infarction | -0.003 | 0.038 | 9.5E-01 | 1.0 | 0.9 | 1.1 | 9.5E-01 |
| Thigh bone marrow fat | Aortic aneurysm | -0.023 | 0.061 | 7.1E-01 | 1.0 | 0.9 | 1.1 | 9.5E-01 |
| Thigh bone marrow fat | Heart failure | 0.033 | 0.031 | 2.9E-01 | 1.0 | 1.0 | 1.1 | 9.5E-01 |
| Thigh bone marrow fat | Atrial fibrillation | 0.102 | 0.047 | 3.1E-02 | 1.1 | 1.0 | 1.2 | 7.2E-01 |
| Thigh bone marrow fat | Peripheral artery disease | -0.041 | 0.052 | 4.3E-01 | 1.0 | 0.9 | 1.1 | 9.5E-01 |
| Thigh bone marrow fat | Deep vein thrombosis | 0.002 | 0.002 | 2.6E-01 | 1.0 | 1.0 | 1.0 | 9.5E-01 |
| Thigh bone marrow fat | Pulmonary embolism | 0.084 | 0.063 | 1.8E-01 | 1.1 | 1.0 | 1.2 | 9.5E-01 |
| Thigh bone marrow fat | Gout | -0.081 | 0.091 | 3.7E-01 | 0.9 | 0.8 | 1.1 | 9.5E-01 |
| Thigh bone marrow fat | Knee osteoarthritis | -0.206 | 0.041 | 5.4E-07 | 0.8 | 0.8 | 0.9 | 1.4E-05 |
| Thigh bone marrow fat | Hip osteoarthritis | -0.133 | 0.053 | 1.2E-02 | 0.9 | 0.8 | 1.0 | 2.9E-01 |
| Thigh bone marrow fat | Rheumatoid arthritis | -0.011 | 0.044 | 8.1E-01 | 1.0 | 0.9 | 1.1 | 9.5E-01 |
| Thigh bone marrow fat | Osteoporosis | 0.011 | 0.003 | 5.3E-05 | 1.0 | 1.0 | 1.0 | 1.3E-03 |
| Thigh bone marrow fat | Asthma | 0.052 | 0.028 | 6.5E-02 | 1.1 | 1.0 | 1.1 | 9.5E-01 |
| Thigh bone marrow fat | Psoriasis | -0.028 | 0.054 | 6.0E-01 | 1.0 | 0.9 | 1.1 | 9.5E-01 |
| Thigh bone marrow fat | Depression | -0.039 | 0.035 | 2.7E-01 | 1.0 | 0.9 | 1.0 | 9.5E-01 |
| Thigh bone marrow fat | Parkinson's disease | -0.087 | 0.061 | 1.6E-01 | 0.9 | 0.8 | 1.0 | 9.5E-01 |
| Thigh bone marrow fat | Alzheimer’s disease | -0.036 | 0.038 | 3.5E-01 | 1.0 | 0.9 | 1.0 | 9.5E-01 |
| Thigh bone marrow fat | Cholelithiasis | -0.019 | 0.048 | 7.0E-01 | 1.0 | 0.9 | 1.1 | 9.5E-01 |
| Thigh bone marrow fat | Gastroesophageal reflux disease | 0.023 | 0.022 | 3.1E-01 | 1.0 | 1.0 | 1.1 | 9.5E-01 |
| Vertebrae bone marrow fat | Type 2 diabetes | 0.101 | 0.083 | 2.2E-01 | 1.1 | 0.9 | 1.3 | 9.9E-01 |
| Vertebrae bone marrow fat | Polycystic ovary syndrome | 0.101 | 0.205 | 6.2E-01 | 1.1 | 0.7 | 1.7 | 9.9E-01 |
| Vertebrae bone marrow fat | MASLD | 0.095 | 0.111 | 3.9E-01 | 1.1 | 0.9 | 1.4 | 9.9E-01 |
| Vertebrae bone marrow fat | Chronic kidney disease | 0.078 | 0.070 | 2.7E-01 | 1.1 | 0.9 | 1.2 | 9.9E-01 |
| Vertebrae bone marrow fat | Hypertension | 0.006 | 0.007 | 3.6E-01 | 1.0 | 1.0 | 1.0 | 9.9E-01 |
| Vertebrae bone marrow fat | Coronary artery disease | 0.061 | 0.059 | 3.0E-01 | 1.1 | 0.9 | 1.2 | 9.9E-01 |
| Vertebrae bone marrow fat | Stroke | -0.036 | 0.073 | 6.2E-01 | 1.0 | 0.8 | 1.1 | 9.9E-01 |
| Vertebrae bone marrow fat | Myocardial infarction | 0.078 | 0.059 | 1.9E-01 | 1.1 | 1.0 | 1.2 | 9.9E-01 |
| Vertebrae bone marrow fat | Aortic aneurysm | -0.103 | 0.118 | 3.8E-01 | 0.9 | 0.7 | 1.1 | 9.9E-01 |
| Vertebrae bone marrow fat | Heart failure | -0.065 | 0.052 | 2.1E-01 | 0.9 | 0.8 | 1.0 | 9.9E-01 |
| Vertebrae bone marrow fat | Atrial fibrillation | -0.041 | 0.066 | 5.4E-01 | 1.0 | 0.8 | 1.1 | 9.9E-01 |
| Vertebrae bone marrow fat | Peripheral artery disease | 0.113 | 0.096 | 2.4E-01 | 1.1 | 0.9 | 1.4 | 9.9E-01 |
| Vertebrae bone marrow fat | Deep vein thrombosis | 0.000 | 0.003 | 9.3E-01 | 1.0 | 1.0 | 1.0 | 9.9E-01 |
| Vertebrae bone marrow fat | Pulmonary embolism | -0.032 | 0.167 | 8.5E-01 | 1.0 | 0.7 | 1.3 | 9.9E-01 |
| Vertebrae bone marrow fat | Gout | 0.036 | 0.167 | 8.3E-01 | 1.0 | 0.7 | 1.4 | 9.9E-01 |
| Vertebrae bone marrow fat | Knee osteoarthritis | 0.033 | 0.053 | 5.4E-01 | 1.0 | 0.9 | 1.1 | 9.9E-01 |
| Vertebrae bone marrow fat | Hip osteoarthritis | 0.227 | 0.075 | 2.4E-03 | 1.3 | 1.1 | 1.5 | 6.2E-02 |
| Vertebrae bone marrow fat | Rheumatoid arthritis | -0.161 | 0.131 | 2.2E-01 | 0.9 | 0.7 | 1.1 | 9.9E-01 |
| Vertebrae bone marrow fat | Osteoporosis | -0.005 | 0.003 | 1.1E-01 | 1.0 | 1.0 | 1.0 | 9.9E-01 |
| Vertebrae bone marrow fat | Asthma | -0.119 | 0.067 | 7.3E-02 | 0.9 | 0.8 | 1.0 | 9.9E-01 |
| Vertebrae bone marrow fat | Psoriasis | -0.110 | 0.086 | 2.0E-01 | 0.9 | 0.8 | 1.1 | 9.9E-01 |
| Vertebrae bone marrow fat | Depression | 0.000 | 0.064 | 9.9E-01 | 1.0 | 0.9 | 1.1 | 9.9E-01 |
| Vertebrae bone marrow fat | Parkinson's disease | 0.026 | 0.129 | 8.4E-01 | 1.0 | 0.8 | 1.3 | 9.9E-01 |
| Vertebrae bone marrow fat | Alzheimer’s disease | 0.033 | 0.063 | 5.9E-01 | 1.0 | 0.9 | 1.2 | 9.9E-01 |
| Vertebrae bone marrow fat | Cholelithiasis | -0.058 | 0.046 | 2.1E-01 | 0.9 | 0.9 | 1.0 | 9.9E-01 |
| Vertebrae bone marrow fat | Gastroesophageal reflux disease | 0.075 | 0.041 | 6.4E-02 | 1.1 | 1.0 | 1.2 | 9.9E-01 |

**Supplementary table.10. The multivariable Mendelian randomisation IVW result.** Outcome: the meta-analysed disease outcomes from FinnGen and PubGwas; Beta: the effect estimate assuming no overlap between IDPs; SE: the standard error of beta; P-value: the statistical significance; OR, OR_lci95 and OR_uci95: the odds ratio and 95% CI; BHP: Benjamini Hochberg corrected pvalue; Robust Est: the effect size after adjusting for the phenotypic correlation between the IDPs; QStat: Cochran’s Q for level of heterogeneity in the genetic instrument; Q_pval: the statistical significance for Statistics.

| **Exposure** | **Outcome** | **Beta** | **SE** | **P-value** | **OR** | **OR_lci95** | **OR_uci95** | **BHP** | **Robust Est** | **QStat** | **Q_pval** |
| --- | --- | --- | --- | --- | --- | --- | --- | --- | --- | --- | --- |
| Abdominal subcutaneous AT | Type 2 diabetes | 0.806 | 0.216 | 1.94E-04 | 2.24 | 1.47 | 3.42 | 0.01 | 0.43 | 2.71 | 1 |
| Abdominal subcutaneous AT | Polycystic ovary syndrome | 0.884 | 0.485 | 6.82E-02 | 2.42 | 0.94 | 6.25 | 0.96 | 1.01 | 6.52 | 1 |
| Abdominal subcutaneous AT | MASLD | -0.022 | 0.454 | 9.61E-01 | 0.98 | 0.40 | 2.38 | 0.96 | -0.14 | 0.65 | 1 |
| Abdominal subcutaneous AT | Chronic kidney disease | -0.008 | 0.147 | 9.55E-01 | 0.99 | 0.74 | 1.32 | 0.96 | -0.08 | 1.50 | 1 |
| Abdominal subcutaneous AT | Hypertension | 0.069 | 0.032 | 3.11E-02 | 1.07 | 1.01 | 1.14 | 0.69 | 0.16 | 3.15 | 1 |
| Abdominal subcutaneous AT | Coronary artery disease | 0.233 | 0.167 | 1.63E-01 | 1.26 | 0.91 | 1.75 | 0.96 | 0.20 | 1.51 | 1 |
| Abdominal subcutaneous AT | Stroke | 0.467 | 0.214 | 2.94E-02 | 1.60 | 1.05 | 2.43 | 0.68 | 0.46 | 14.01 | 1 |
| Abdominal subcutaneous AT | Myocardial infarction | 0.150 | 0.183 | 4.13E-01 | 1.16 | 0.81 | 1.66 | 0.96 | 0.07 | 2.20 | 1 |
| Abdominal subcutaneous AT | Aortic aneurysm | -0.075 | 0.200 | 7.09E-01 | 0.93 | 0.63 | 1.37 | 0.96 | -0.63 | 5.43 | 1 |
| Abdominal subcutaneous AT | Heart failure | 0.319 | 0.123 | 9.73E-03 | 1.38 | 1.08 | 1.75 | 0.23 | 0.31 | 1.29 | 1 |
| Abdominal subcutaneous AT | Atrial fibrillation | 0.400 | 0.139 | 4.04E-03 | 1.49 | 1.14 | 1.96 | 0.10 | 0.35 | 39.20 | 1 |
| Abdominal subcutaneous AT | Peripheral artery disease | 0.199 | 0.178 | 2.64E-01 | 1.22 | 0.86 | 1.73 | 0.96 | -0.01 | 2.94 | 1 |
| Abdominal subcutaneous AT | Deep vein thrombosis | -0.022 | 0.491 | 9.65E-01 | 0.98 | 0.37 | 2.56 | 0.96 | 0.01 | 2.24 | 1 |
| Abdominal subcutaneous AT | Pulmonary embolism | 0.159 | 0.390 | 6.84E-01 | 1.17 | 0.55 | 2.52 | 0.96 | -0.29 | 5.22 | 1 |
| Abdominal subcutaneous AT | Gout | -0.174 | 0.331 | 5.99E-01 | 0.84 | 0.44 | 1.61 | 0.96 | -0.33 | 9.03 | 1 |
| Abdominal subcutaneous AT | Knee osteoarthritis | 0.225 | 0.144 | 1.19E-01 | 1.25 | 0.94 | 1.66 | 0.96 | 0.04 | 4.09 | 1 |
| Abdominal subcutaneous AT | Hip osteoarthritis | 0.387 | 0.207 | 6.15E-02 | 1.47 | 0.98 | 2.21 | 0.96 | 0.06 | 4.53 | 1 |
| Abdominal subcutaneous AT | Rheumatoid arthritis | -0.371 | 0.224 | 9.87E-02 | 0.69 | 0.44 | 1.07 | 0.96 | -0.34 | 21.99 | 1 |
| Abdominal subcutaneous AT | Osteoporosis | -0.002 | 0.007 | 7.75E-01 | 1.00 | 0.99 | 1.01 | 0.96 | -0.10 | 35.61 | 1 |
| Abdominal subcutaneous AT | Asthma | 0.008 | 0.122 | 9.47E-01 | 1.01 | 0.79 | 1.28 | 0.96 | 0.04 | 2.38 | 1 |
| Abdominal subcutaneous AT | Psoriasis | -0.236 | 0.244 | 3.33E-01 | 0.79 | 0.49 | 1.27 | 0.96 | -0.04 | 6.22 | 1 |
| Abdominal subcutaneous AT | Depression | 0.068 | 0.105 | 5.14E-01 | 1.07 | 0.87 | 1.31 | 0.96 | 0.13 | 19.63 | 1 |
| Abdominal subcutaneous AT | Parkinson's disease | 0.113 | 0.318 | 7.22E-01 | 1.12 | 0.60 | 2.09 | 0.96 | -0.06 | 2.53 | 1 |
| Abdominal subcutaneous AT | Alzheimer’s disease | -0.159 | 1.189 | 8.94E-01 | 0.85 | 0.08 | 8.77 | 0.96 | -0.11 | 3.21 | 1 |
| Abdominal subcutaneous AT | Cholelithiasis | 0.195 | 0.175 | 2.66E-01 | 1.21 | 0.86 | 1.71 | 0.96 | 0.18 | 7.00 | 1 |
| Abdominal subcutaneous AT | Gastroesophageal reflux disease | 0.060 | 0.100 | 5.52E-01 | 1.06 | 0.87 | 1.29 | 0.96 | 0.07 | 1.89 | 1 |
| Thigh subcutaneous AT | Type 2 diabetes | -0.593 | 0.125 | 1.94E-06 | 0.55 | 0.43 | 0.71 | 0.00 | -0.31 | 2.71 | 1 |
| Thigh subcutaneous AT | Polycystic ovary syndrome | -0.733 | 0.280 | 8.71E-03 | 0.48 | 0.28 | 0.83 | 0.17 | -0.55 | 6.52 | 1 |
| Thigh subcutaneous AT | MASLD | -0.165 | 0.262 | 5.29E-01 | 0.85 | 0.51 | 1.42 | 0.96 | 0.02 | 0.65 | 1 |
| Thigh subcutaneous AT | Chronic kidney disease | -0.005 | 0.084 | 9.56E-01 | 1.00 | 0.84 | 1.17 | 0.96 | 0.03 | 1.50 | 1 |
| Thigh subcutaneous AT | Hypertension | -0.098 | 0.018 | 6.22E-08 | 0.91 | 0.88 | 0.94 | 0.00 | -0.21 | 3.15 | 1 |
| Thigh subcutaneous AT | Coronary artery disease | -0.361 | 0.095 | 1.43E-04 | 0.70 | 0.58 | 0.84 | 0.00 | -0.27 | 1.51 | 1 |
| Thigh subcutaneous AT | Stroke | -0.194 | 0.124 | 1.16E-01 | 0.82 | 0.65 | 1.05 | 0.96 | -0.18 | 14.01 | 1 |
| Thigh subcutaneous AT | Myocardial infarction | -0.399 | 0.105 | 1.44E-04 | 0.67 | 0.55 | 0.82 | 0.00 | -0.34 | 2.20 | 1 |
| Thigh subcutaneous AT | Aortic aneurysm | -0.365 | 0.114 | 1.37E-03 | 0.69 | 0.56 | 0.87 | 0.03 | -0.28 | 5.43 | 1 |
| Thigh subcutaneous AT | Heart failure | -0.102 | 0.071 | 1.52E-01 | 0.90 | 0.79 | 1.04 | 0.96 | -0.05 | 1.29 | 1 |
| Thigh subcutaneous AT | Atrial fibrillation | -0.060 | 0.080 | 4.55E-01 | 0.94 | 0.81 | 1.10 | 0.96 | -0.08 | 39.20 | 1 |
| Thigh subcutaneous AT | Peripheral artery disease | -0.279 | 0.100 | 5.16E-03 | 0.76 | 0.62 | 0.92 | 0.11 | -0.08 | 2.94 | 1 |
| Thigh subcutaneous AT | Deep vein thrombosis | 0.212 | 0.284 | 4.55E-01 | 1.24 | 0.71 | 2.15 | 0.96 | 0.05 | 2.24 | 1 |
| Thigh subcutaneous AT | Pulmonary embolism | 0.192 | 0.225 | 3.95E-01 | 1.21 | 0.78 | 1.88 | 0.96 | 0.28 | 5.22 | 1 |
| Thigh subcutaneous AT | Gout | -0.109 | 0.191 | 5.70E-01 | 0.90 | 0.62 | 1.31 | 0.96 | -0.22 | 9.03 | 1 |
| Thigh subcutaneous AT | Knee osteoarthritis | 0.136 | 0.083 | 1.00E-01 | 1.15 | 0.97 | 1.35 | 0.96 | 0.13 | 4.09 | 1 |
| Thigh subcutaneous AT | Hip osteoarthritis | -0.180 | 0.119 | 1.31E-01 | 0.83 | 0.66 | 1.06 | 0.96 | -0.11 | 4.53 | 1 |
| Thigh subcutaneous AT | Rheumatoid arthritis | 0.145 | 0.125 | 2.44E-01 | 1.16 | 0.91 | 1.48 | 0.96 | 0.03 | 21.99 | 1 |
| Thigh subcutaneous AT | Osteoporosis | 0.008 | 0.004 | 3.92E-02 | 1.01 | 1.00 | 1.02 | 0.74 | 0.27 | 35.61 | 1 |
| Thigh subcutaneous AT | Asthma | 0.074 | 0.070 | 2.86E-01 | 1.08 | 0.94 | 1.24 | 0.96 | 0.12 | 2.38 | 1 |
| Thigh subcutaneous AT | Psoriasis | -0.024 | 0.140 | 8.66E-01 | 0.98 | 0.74 | 1.29 | 0.96 | 0.11 | 6.22 | 1 |
| Thigh subcutaneous AT | Depression | 0.031 | 0.060 | 6.03E-01 | 1.03 | 0.92 | 1.16 | 0.96 | 0.00 | 19.63 | 1 |
| Thigh subcutaneous AT | Parkinson's disease | -0.147 | 0.184 | 4.24E-01 | 0.86 | 0.60 | 1.24 | 0.96 | -0.07 | 2.53 | 1 |
| Thigh subcutaneous AT | Alzheimer’s disease | -0.802 | 0.687 | 2.43E-01 | 0.45 | 0.12 | 1.72 | 0.96 | -0.18 | 3.21 | 1 |
| Thigh subcutaneous AT | Cholelithiasis | 0.005 | 0.099 | 9.57E-01 | 1.01 | 0.83 | 1.22 | 0.96 | -0.09 | 7.00 | 1 |
| Thigh subcutaneous AT | Gastroesophageal reflux disease | 0.037 | 0.057 | 5.14E-01 | 1.04 | 0.93 | 1.16 | 0.96 | 0.08 | 1.89 | 1 |
| Thigh intermuscular AT | Type 2 diabetes | -0.475 | 0.153 | 1.94E-03 | 0.62 | 0.46 | 0.84 | 0.05 | -0.47 | 2.71 | 1 |
| Thigh intermuscular AT | Polycystic ovary syndrome | -0.244 | 0.343 | 4.76E-01 | 0.78 | 0.40 | 1.53 | 0.99 | -0.66 | 6.52 | 1 |
| Thigh intermuscular AT | MASLD | -0.024 | 0.321 | 9.41E-01 | 0.98 | 0.52 | 1.83 | 0.99 | 0.31 | 0.65 | 1 |
| Thigh intermuscular AT | Chronic kidney disease | -0.122 | 0.102 | 2.34E-01 | 0.89 | 0.72 | 1.08 | 0.99 | -0.13 | 1.50 | 1 |
| Thigh intermuscular AT | Hypertension | -0.039 | 0.022 | 7.27E-02 | 0.96 | 0.92 | 1.00 | 0.99 | -0.03 | 3.15 | 1 |
| Thigh intermuscular AT | Coronary artery disease | -0.204 | 0.115 | 7.75E-02 | 0.82 | 0.65 | 1.02 | 0.99 | -0.11 | 1.51 | 1 |
| Thigh intermuscular AT | Stroke | -0.182 | 0.153 | 2.34E-01 | 0.83 | 0.62 | 1.12 | 0.99 | 0.07 | 14.01 | 1 |
| Thigh intermuscular AT | Myocardial infarction | -0.098 | 0.128 | 4.46E-01 | 0.91 | 0.71 | 1.17 | 0.99 | 0.19 | 2.20 | 1 |
| Thigh intermuscular AT | Aortic aneurysm | -0.238 | 0.140 | 8.80E-02 | 0.79 | 0.60 | 1.04 | 0.99 | -0.01 | 5.43 | 1 |
| Thigh intermuscular AT | Heart failure | 0.057 | 0.086 | 5.08E-01 | 1.06 | 0.89 | 1.25 | 0.99 | 0.14 | 1.29 | 1 |
| Thigh intermuscular AT | Atrial fibrillation | -0.046 | 0.098 | 6.41E-01 | 0.96 | 0.79 | 1.16 | 0.99 | 0.08 | 39.20 | 1 |
| Thigh intermuscular AT | Peripheral artery disease | -0.413 | 0.123 | 8.06E-04 | 0.66 | 0.52 | 0.84 | 0.02 | -0.12 | 2.94 | 1 |
| Thigh intermuscular AT | Deep vein thrombosis | 0.210 | 0.348 | 5.46E-01 | 1.23 | 0.62 | 2.44 | 0.99 | 0.09 | 2.24 | 1 |
| Thigh intermuscular AT | Pulmonary embolism | 0.097 | 0.275 | 7.25E-01 | 1.10 | 0.64 | 1.89 | 0.99 | 0.36 | 5.22 | 1 |
| Thigh intermuscular AT | Gout | -0.030 | 0.241 | 9.01E-01 | 0.97 | 0.60 | 1.56 | 0.99 | -0.15 | 9.03 | 1 |
| Thigh intermuscular AT | Knee osteoarthritis | -0.001 | 0.101 | 9.95E-01 | 1.00 | 0.82 | 1.22 | 0.99 | 0.21 | 4.09 | 1 |
| Thigh intermuscular AT | Hip osteoarthritis | -0.109 | 0.146 | 4.56E-01 | 0.90 | 0.67 | 1.19 | 0.99 | 0.13 | 4.53 | 1 |
| Thigh intermuscular AT | Rheumatoid arthritis | 0.115 | 0.155 | 4.57E-01 | 1.12 | 0.83 | 1.52 | 0.99 | 0.30 | 21.99 | 1 |
| Thigh intermuscular AT | Osteoporosis | 0.008 | 0.005 | 6.70E-02 | 1.01 | 1.00 | 1.02 | 0.99 | 0.44 | 35.61 | 1 |
| Thigh intermuscular AT | Asthma | 0.160 | 0.085 | 6.06E-02 | 1.17 | 0.99 | 1.39 | 0.99 | 0.04 | 2.38 | 1 |
| Thigh intermuscular AT | Psoriasis | 0.010 | 0.172 | 9.51E-01 | 1.01 | 0.72 | 1.42 | 0.99 | -0.16 | 6.22 | 1 |
| Thigh intermuscular AT | Depression | 0.039 | 0.074 | 6.01E-01 | 1.04 | 0.90 | 1.20 | 0.99 | -0.21 | 19.63 | 1 |
| Thigh intermuscular AT | Parkinson's disease | 0.206 | 0.225 | 3.60E-01 | 1.23 | 0.79 | 1.91 | 0.99 | -0.07 | 2.53 | 1 |
| Thigh intermuscular AT | Alzheimer’s disease | -0.166 | 0.841 | 8.43E-01 | 0.85 | 0.16 | 4.40 | 0.99 | -0.11 | 3.21 | 1 |
| Thigh intermuscular AT | Cholelithiasis | -0.076 | 0.121 | 5.30E-01 | 0.93 | 0.73 | 1.18 | 0.99 | -0.08 | 7.00 | 1 |
| Thigh intermuscular AT | Gastroesophageal reflux disease | 0.047 | 0.070 | 5.06E-01 | 1.05 | 0.91 | 1.20 | 0.99 | 0.06 | 1.89 | 1 |
| Visceral AT | Type 2 diabetes | 0.340 | 0.199 | 8.73E-02 | 1.40 | 0.95 | 2.07 | 0.97 | 0.37 | 2.71 | 1 |
| Visceral AT | Polycystic ovary syndrome | 0.261 | 0.443 | 5.55E-01 | 1.30 | 0.54 | 3.10 | 0.97 | 0.58 | 6.52 | 1 |
| Visceral AT | MASLD | 0.277 | 0.417 | 5.06E-01 | 1.32 | 0.58 | 2.98 | 0.97 | -0.03 | 0.65 | 1 |
| Visceral AT | Chronic kidney disease | 0.307 | 0.137 | 2.56E-02 | 1.36 | 1.04 | 1.78 | 0.61 | 0.18 | 1.50 | 1 |
| Visceral AT | Hypertension | 0.005 | 0.029 | 8.68E-01 | 1.00 | 0.95 | 1.06 | 0.97 | 0.04 | 3.15 | 1 |
| Visceral AT | Coronary artery disease | 0.128 | 0.158 | 4.17E-01 | 1.14 | 0.83 | 1.55 | 0.97 | 0.03 | 1.51 | 1 |
| Visceral AT | Stroke | -0.074 | 0.197 | 7.08E-01 | 0.93 | 0.63 | 1.37 | 0.97 | -0.20 | 14.01 | 1 |
| Visceral AT | Myocardial infarction | 0.017 | 0.167 | 9.21E-01 | 1.02 | 0.73 | 1.41 | 0.97 | -0.07 | 2.20 | 1 |
| Visceral AT | Aortic aneurysm | 0.416 | 0.184 | 2.36E-02 | 1.52 | 1.06 | 2.17 | 0.59 | 0.56 | 5.43 | 1 |
| Visceral AT | Heart failure | -0.030 | 0.114 | 7.90E-01 | 0.97 | 0.78 | 1.21 | 0.97 | -0.04 | 1.29 | 1 |
| Visceral AT | Atrial fibrillation | -0.284 | 0.129 | 2.76E-02 | 0.75 | 0.58 | 0.97 | 0.64 | -0.20 | 39.20 | 1 |
| Visceral AT | Peripheral artery disease | 0.273 | 0.164 | 9.67E-02 | 1.31 | 0.95 | 1.81 | 0.97 | 0.33 | 2.94 | 1 |
| Visceral AT | Deep vein thrombosis | 0.170 | 0.449 | 7.05E-01 | 1.18 | 0.49 | 2.86 | 0.97 | 0.13 | 2.24 | 1 |
| Visceral AT | Pulmonary embolism | -0.228 | 0.357 | 5.23E-01 | 0.80 | 0.40 | 1.60 | 0.97 | 0.06 | 5.22 | 1 |
| Visceral AT | Gout | 0.582 | 0.320 | 6.90E-02 | 1.79 | 0.96 | 3.35 | 0.97 | 0.49 | 9.03 | 1 |
| Visceral AT | Knee osteoarthritis | -0.084 | 0.131 | 5.21E-01 | 0.92 | 0.71 | 1.19 | 0.97 | -0.21 | 4.09 | 1 |
| Visceral AT | Hip osteoarthritis | 0.304 | 0.189 | 1.09E-01 | 1.35 | 0.93 | 1.96 | 0.97 | 0.03 | 4.53 | 1 |
| Visceral AT | Rheumatoid arthritis | 0.136 | 0.214 | 5.26E-01 | 1.15 | 0.75 | 1.74 | 0.97 | 0.00 | 21.99 | 1 |
| Visceral AT | Osteoporosis | -0.004 | 0.006 | 5.09E-01 | 1.00 | 0.98 | 1.01 | 0.97 | 0.07 | 35.61 | 1 |
| Visceral AT | Asthma | 0.026 | 0.111 | 8.12E-01 | 1.03 | 0.83 | 1.28 | 0.97 | 0.09 | 2.38 | 1 |
| Visceral AT | Psoriasis | 0.275 | 0.224 | 2.20E-01 | 1.32 | 0.85 | 2.04 | 0.97 | 0.47 | 6.22 | 1 |
| Visceral AT | Depression | 0.004 | 0.096 | 9.65E-01 | 1.00 | 0.83 | 1.21 | 0.97 | 0.10 | 19.63 | 1 |
| Visceral AT | Parkinson's disease | -0.079 | 0.291 | 7.86E-01 | 0.92 | 0.52 | 1.64 | 0.97 | -0.07 | 2.53 | 1 |
| Visceral AT | Alzheimer’s disease | -1.876 | 1.083 | 8.31E-02 | 0.15 | 0.02 | 1.28 | 0.97 | -0.45 | 3.21 | 1 |
| Visceral AT | Cholelithiasis | 0.423 | 0.160 | 8.23E-03 | 1.53 | 1.12 | 2.09 | 0.21 | 0.41 | 7.00 | 1 |
| Visceral AT | Gastroesophageal reflux disease | 0.161 | 0.097 | 9.83E-02 | 1.17 | 0.97 | 1.42 | 0.97 | 0.09 | 1.89 | 1 |
| Liver PDFF | Type 2 diabetes | 0.201 | 0.055 | 2.71E-04 | 1.22 | 1.10 | 1.36 | 0.01 | 0.32 | 2.71 | 1 |
| Liver PDFF | Polycystic ovary syndrome | 0.016 | 0.122 | 8.97E-01 | 1.02 | 0.80 | 1.29 | 0.93 | 0.08 | 6.52 | 1 |
| Liver PDFF | MASLD | 1.705 | 0.106 | 1.69E-58 | 5.50 | 4.47 | 6.77 | 0.00 | 1.33 | 0.65 | 1 |
| Liver PDFF | Chronic kidney disease | -0.017 | 0.038 | 6.58E-01 | 0.98 | 0.91 | 1.06 | 0.93 | -0.01 | 1.50 | 1 |
| Liver PDFF | Hypertension | 0.004 | 0.008 | 6.75E-01 | 1.00 | 0.99 | 1.02 | 0.93 | 0.05 | 3.15 | 1 |
| Liver PDFF | Coronary artery disease | -0.085 | 0.043 | 4.93E-02 | 0.92 | 0.84 | 1.00 | 0.84 | -0.11 | 1.51 | 1 |
| Liver PDFF | Stroke | -0.047 | 0.054 | 3.87E-01 | 0.95 | 0.86 | 1.06 | 0.93 | -0.01 | 14.01 | 1 |
| Liver PDFF | Myocardial infarction | -0.160 | 0.047 | 7.15E-04 | 0.85 | 0.78 | 0.93 | 0.02 | -0.16 | 2.20 | 1 |
| Liver PDFF | Aortic aneurysm | -0.104 | 0.050 | 3.84E-02 | 0.90 | 0.82 | 0.99 | 0.73 | -0.13 | 5.43 | 1 |
| Liver PDFF | Heart failure | -0.004 | 0.032 | 9.10E-01 | 1.00 | 0.94 | 1.06 | 0.93 | 0.00 | 1.29 | 1 |
| Liver PDFF | Atrial fibrillation | -0.017 | 0.036 | 6.31E-01 | 0.98 | 0.92 | 1.05 | 0.93 | -0.03 | 39.20 | 1 |
| Liver PDFF | Peripheral artery disease | -0.118 | 0.044 | 6.90E-03 | 0.89 | 0.82 | 0.97 | 0.14 | -0.13 | 2.94 | 1 |
| Liver PDFF | Deep vein thrombosis | -0.037 | 0.124 | 7.65E-01 | 0.96 | 0.76 | 1.23 | 0.93 | -0.01 | 2.24 | 1 |
| Liver PDFF | Pulmonary embolism | -0.041 | 0.099 | 6.82E-01 | 0.96 | 0.79 | 1.17 | 0.93 | 0.07 | 5.22 | 1 |
| Liver PDFF | Gout | -0.095 | 0.088 | 2.78E-01 | 0.91 | 0.77 | 1.08 | 0.93 | -0.15 | 9.03 | 1 |
| Liver PDFF | Knee osteoarthritis | 0.057 | 0.037 | 1.26E-01 | 1.06 | 0.98 | 1.14 | 0.93 | 0.04 | 4.09 | 1 |
| Liver PDFF | Hip osteoarthritis | 0.011 | 0.052 | 8.31E-01 | 1.01 | 0.91 | 1.12 | 0.93 | 0.05 | 4.53 | 1 |
| Liver PDFF | Rheumatoid arthritis | 0.015 | 0.054 | 7.86E-01 | 1.01 | 0.91 | 1.13 | 0.93 | 0.00 | 21.99 | 1 |
| Liver PDFF | Osteoporosis | 0.004 | 0.002 | 2.60E-02 | 1.00 | 1.00 | 1.01 | 0.52 | 0.16 | 35.61 | 1 |
| Liver PDFF | Asthma | 0.018 | 0.032 | 5.59E-01 | 1.02 | 0.96 | 1.08 | 0.93 | 0.00 | 2.38 | 1 |
| Liver PDFF | Psoriasis | 0.123 | 0.061 | 4.52E-02 | 1.13 | 1.00 | 1.28 | 0.81 | 0.10 | 6.22 | 1 |
| Liver PDFF | Depression | -0.018 | 0.026 | 5.02E-01 | 0.98 | 0.93 | 1.03 | 0.93 | 0.00 | 19.63 | 1 |
| Liver PDFF | Parkinson's disease | -0.130 | 0.081 | 1.07E-01 | 0.88 | 0.75 | 1.03 | 0.93 | 0.00 | 2.53 | 1 |
| Liver PDFF | Alzheimer’s disease | -0.907 | 0.297 | 2.26E-03 | 0.40 | 0.23 | 0.72 | 0.05 | -0.23 | 3.21 | 1 |
| Liver PDFF | Cholelithiasis | -0.123 | 0.044 | 4.88E-03 | 0.88 | 0.81 | 0.96 | 0.11 | -0.09 | 7.00 | 1 |
| Liver PDFF | Gastroesophageal reflux disease | 0.002 | 0.025 | 9.29E-01 | 1.00 | 0.95 | 1.05 | 0.93 | 0.02 | 1.89 | 1 |
| Pancreas PDFF | Type 2 diabetes | -0.005 | 0.100 | 9.62E-01 | 1.00 | 0.82 | 1.21 | 0.96 | -0.04 | 2.71 | 1 |
| Pancreas PDFF | Polycystic ovary syndrome | 0.106 | 0.216 | 6.24E-01 | 1.11 | 0.73 | 1.70 | 0.96 | -0.05 | 6.52 | 1 |
| Pancreas PDFF | MASLD | -0.246 | 0.203 | 2.25E-01 | 0.78 | 0.53 | 1.16 | 0.96 | -0.31 | 0.65 | 1 |
| Pancreas PDFF | Chronic kidney disease | -0.096 | 0.069 | 1.67E-01 | 0.91 | 0.79 | 1.04 | 0.96 | -0.20 | 1.50 | 1 |
| Pancreas PDFF | Hypertension | -0.004 | 0.014 | 7.74E-01 | 1.00 | 0.97 | 1.02 | 0.96 | 0.05 | 3.15 | 1 |
| Pancreas PDFF | Coronary artery disease | 0.036 | 0.077 | 6.42E-01 | 1.04 | 0.89 | 1.21 | 0.96 | 0.02 | 1.51 | 1 |
| Pancreas PDFF | Stroke | 0.013 | 0.098 | 8.95E-01 | 1.01 | 0.84 | 1.23 | 0.96 | 0.12 | 14.01 | 1 |
| Pancreas PDFF | Myocardial infarction | 0.156 | 0.081 | 5.51E-02 | 1.17 | 1.00 | 1.37 | 0.96 | 0.23 | 2.20 | 1 |
| Pancreas PDFF | Aortic aneurysm | -0.018 | 0.090 | 8.38E-01 | 0.98 | 0.82 | 1.17 | 0.96 | 0.01 | 5.43 | 1 |
| Pancreas PDFF | Heart failure | 0.053 | 0.054 | 3.27E-01 | 1.05 | 0.95 | 1.17 | 0.96 | 0.00 | 1.29 | 1 |
| Pancreas PDFF | Atrial fibrillation | 0.076 | 0.064 | 2.38E-01 | 1.08 | 0.95 | 1.22 | 0.96 | 0.17 | 39.20 | 1 |
| Pancreas PDFF | Peripheral artery disease | 0.062 | 0.082 | 4.49E-01 | 1.06 | 0.91 | 1.25 | 0.96 | 0.05 | 2.94 | 1 |
| Pancreas PDFF | Deep vein thrombosis | 0.747 | 0.218 | 6.00E-04 | 2.11 | 1.38 | 3.24 | 0.02 | 0.38 | 2.24 | 1 |
| Pancreas PDFF | Pulmonary embolism | 0.397 | 0.173 | 2.17E-02 | 1.49 | 1.06 | 2.09 | 0.54 | 0.40 | 5.22 | 1 |
| Pancreas PDFF | Gout | -0.276 | 0.160 | 8.48E-02 | 0.76 | 0.55 | 1.04 | 0.96 | -0.44 | 9.03 | 1 |
| Pancreas PDFF | Knee osteoarthritis | 0.061 | 0.063 | 3.36E-01 | 1.06 | 0.94 | 1.20 | 0.96 | 0.08 | 4.09 | 1 |
| Pancreas PDFF | Hip osteoarthritis | -0.093 | 0.092 | 3.14E-01 | 0.91 | 0.76 | 1.09 | 0.96 | -0.09 | 4.53 | 1 |
| Pancreas PDFF | Rheumatoid arthritis | -0.129 | 0.102 | 2.04E-01 | 0.88 | 0.72 | 1.07 | 0.96 | -0.09 | 21.99 | 1 |
| Pancreas PDFF | Osteoporosis | -0.001 | 0.003 | 6.35E-01 | 1.00 | 0.99 | 1.00 | 0.96 | 0.20 | 35.61 | 1 |
| Pancreas PDFF | Asthma | 0.027 | 0.053 | 6.11E-01 | 1.03 | 0.93 | 1.14 | 0.96 | -0.11 | 2.38 | 1 |
| Pancreas PDFF | Psoriasis | -0.181 | 0.109 | 9.70E-02 | 0.83 | 0.67 | 1.03 | 0.96 | -0.12 | 6.22 | 1 |
| Pancreas PDFF | Depression | -0.034 | 0.047 | 4.76E-01 | 0.97 | 0.88 | 1.06 | 0.96 | -0.01 | 19.63 | 1 |
| Pancreas PDFF | Parkinson's disease | -0.281 | 0.142 | 4.85E-02 | 0.76 | 0.57 | 1.00 | 0.96 | -0.07 | 2.53 | 1 |
| Pancreas PDFF | Alzheimer’s disease | 0.562 | 0.529 | 2.87E-01 | 1.76 | 0.62 | 4.95 | 0.96 | 0.32 | 3.21 | 1 |
| Pancreas PDFF | Cholelithiasis | 0.079 | 0.079 | 3.19E-01 | 1.08 | 0.93 | 1.26 | 0.96 | 0.01 | 7.00 | 1 |
| Pancreas PDFF | Gastroesophageal reflux disease | -0.002 | 0.048 | 9.60E-01 | 1.00 | 0.91 | 1.10 | 0.96 | -0.01 | 1.89 | 1 |
| Paraspinal AT | Type 2 diabetes | -0.026 | 0.152 | 8.64E-01 | 0.97 | 0.72 | 1.31 | 0.99 | -0.01 | 2.71 | 1 |
| Paraspinal AT | Polycystic ovary syndrome | -0.089 | 0.342 | 7.94E-01 | 0.91 | 0.47 | 1.79 | 0.99 | 0.10 | 6.52 | 1 |
| Paraspinal AT | MASLD | -0.322 | 0.321 | 3.15E-01 | 0.72 | 0.39 | 1.36 | 0.99 | -0.35 | 0.65 | 1 |
| Paraspinal AT | Chronic kidney disease | 0.082 | 0.102 | 4.26E-01 | 1.08 | 0.89 | 1.33 | 0.99 | 0.19 | 1.50 | 1 |
| Paraspinal AT | Hypertension | -0.005 | 0.022 | 8.17E-01 | 0.99 | 0.95 | 1.04 | 0.99 | -0.07 | 3.15 | 1 |
| Paraspinal AT | Coronary artery disease | -0.002 | 0.115 | 9.89E-01 | 1.00 | 0.80 | 1.25 | 0.99 | -0.08 | 1.51 | 1 |
| Paraspinal AT | Stroke | -0.047 | 0.152 | 7.55E-01 | 0.95 | 0.71 | 1.28 | 0.99 | -0.15 | 14.01 | 1 |
| Paraspinal AT | Myocardial infarction | -0.091 | 0.127 | 4.77E-01 | 0.91 | 0.71 | 1.17 | 0.99 | -0.31 | 2.20 | 1 |
| Paraspinal AT | Aortic aneurysm | 0.086 | 0.139 | 5.37E-01 | 1.09 | 0.83 | 1.43 | 0.99 | -0.23 | 5.43 | 1 |
| Paraspinal AT | Heart failure | -0.089 | 0.086 | 3.01E-01 | 0.92 | 0.77 | 1.08 | 0.99 | -0.08 | 1.29 | 1 |
| Paraspinal AT | Atrial fibrillation | 0.051 | 0.096 | 5.94E-01 | 1.05 | 0.87 | 1.27 | 0.99 | -0.08 | 39.20 | 1 |
| Paraspinal AT | Peripheral artery disease | 0.063 | 0.122 | 6.05E-01 | 1.07 | 0.84 | 1.35 | 0.99 | -0.13 | 2.94 | 1 |
| Paraspinal AT | Deep vein thrombosis | -0.472 | 0.346 | 1.73E-01 | 0.62 | 0.32 | 1.23 | 0.99 | -0.22 | 2.24 | 1 |
| Paraspinal AT | Pulmonary embolism | -0.240 | 0.275 | 3.81E-01 | 0.79 | 0.46 | 1.35 | 0.99 | -0.48 | 5.22 | 1 |
| Paraspinal AT | Gout | 0.284 | 0.255 | 2.65E-01 | 1.33 | 0.81 | 2.19 | 0.99 | 0.36 | 9.03 | 1 |
| Paraspinal AT | Knee osteoarthritis | 0.063 | 0.100 | 5.26E-01 | 1.07 | 0.88 | 1.30 | 0.99 | -0.09 | 4.09 | 1 |
| Paraspinal AT | Hip osteoarthritis | 0.183 | 0.146 | 2.09E-01 | 1.20 | 0.90 | 1.60 | 0.99 | 0.09 | 4.53 | 1 |
| Paraspinal AT | Rheumatoid arthritis | 0.020 | 0.153 | 8.96E-01 | 1.02 | 0.76 | 1.38 | 0.99 | -0.26 | 21.99 | 1 |
| Paraspinal AT | Osteoporosis | -0.006 | 0.005 | 1.57E-01 | 0.99 | 0.98 | 1.00 | 0.99 | -0.25 | 35.61 | 1 |
| Paraspinal AT | Asthma | -0.021 | 0.084 | 7.99E-01 | 0.98 | 0.83 | 1.15 | 0.99 | 0.10 | 2.38 | 1 |
| Paraspinal AT | Psoriasis | -0.084 | 0.172 | 6.26E-01 | 0.92 | 0.66 | 1.29 | 0.99 | -0.06 | 6.22 | 1 |
| Paraspinal AT | Depression | -0.040 | 0.074 | 5.87E-01 | 0.96 | 0.83 | 1.11 | 0.99 | 0.20 | 19.63 | 1 |
| Paraspinal AT | Parkinson's disease | -0.169 | 0.225 | 4.52E-01 | 0.84 | 0.54 | 1.31 | 0.99 | 0.06 | 2.53 | 1 |
| Paraspinal AT | Alzheimer’s disease | -0.433 | 0.839 | 6.06E-01 | 0.65 | 0.13 | 3.36 | 0.99 | -0.14 | 3.21 | 1 |
| Paraspinal AT | Cholelithiasis | -0.084 | 0.120 | 4.85E-01 | 0.92 | 0.73 | 1.16 | 0.99 | -0.09 | 7.00 | 1 |
| Paraspinal AT | Gastroesophageal reflux disease | 0.038 | 0.076 | 6.21E-01 | 1.04 | 0.89 | 1.21 | 0.99 | 0.10 | 1.89 | 1 |
| Pelvic bone marrow fat | Type 2 diabetes | -0.093 | 0.172 | 5.90E-01 | 0.91 | 0.65 | 1.28 | 1.00 | -0.17 | 2.71 | 1 |
| Pelvic bone marrow fat | Polycystic ovary syndrome | -0.060 | 0.393 | 8.78E-01 | 0.94 | 0.44 | 2.04 | 1.00 | -0.59 | 6.52 | 1 |
| Pelvic bone marrow fat | MASLD | 0.200 | 0.369 | 5.88E-01 | 1.22 | 0.59 | 2.52 | 1.00 | 0.40 | 0.65 | 1 |
| Pelvic bone marrow fat | Chronic kidney disease | -0.196 | 0.114 | 8.56E-02 | 0.82 | 0.66 | 1.03 | 1.00 | 0.00 | 1.50 | 1 |
| Pelvic bone marrow fat | Hypertension | -0.035 | 0.024 | 1.46E-01 | 0.97 | 0.92 | 1.01 | 1.00 | -0.16 | 3.15 | 1 |
| Pelvic bone marrow fat | Coronary artery disease | -0.286 | 0.125 | 2.23E-02 | 0.75 | 0.59 | 0.96 | 0.56 | -0.17 | 1.51 | 1 |
| Pelvic bone marrow fat | Stroke | -0.001 | 0.175 | 9.96E-01 | 1.00 | 0.71 | 1.41 | 1.00 | -0.05 | 14.01 | 1 |
| Pelvic bone marrow fat | Myocardial infarction | -0.219 | 0.145 | 1.30E-01 | 0.80 | 0.60 | 1.07 | 1.00 | -0.15 | 2.20 | 1 |
| Pelvic bone marrow fat | Aortic aneurysm | -0.066 | 0.156 | 6.74E-01 | 0.94 | 0.69 | 1.27 | 1.00 | 0.16 | 5.43 | 1 |
| Pelvic bone marrow fat | Heart failure | -0.153 | 0.098 | 1.17E-01 | 0.86 | 0.71 | 1.04 | 1.00 | -0.15 | 1.29 | 1 |
| Pelvic bone marrow fat | Atrial fibrillation | -0.036 | 0.108 | 7.42E-01 | 0.97 | 0.78 | 1.19 | 1.00 | -0.20 | 39.20 | 1 |
| Pelvic bone marrow fat | Peripheral artery disease | -0.052 | 0.135 | 7.01E-01 | 0.95 | 0.73 | 1.24 | 1.00 | 0.02 | 2.94 | 1 |
| Pelvic bone marrow fat | Deep vein thrombosis | -0.387 | 0.399 | 3.33E-01 | 0.68 | 0.31 | 1.49 | 1.00 | -0.20 | 2.24 | 1 |
| Pelvic bone marrow fat | Pulmonary embolism | -0.239 | 0.317 | 4.50E-01 | 0.79 | 0.42 | 1.46 | 1.00 | -0.22 | 5.22 | 1 |
| Pelvic bone marrow fat | Gout | -0.817 | 0.269 | 2.40E-03 | 0.44 | 0.26 | 0.75 | 0.06 | -0.11 | 9.03 | 1 |
| Pelvic bone marrow fat | Knee osteoarthritis | -0.007 | 0.114 | 9.51E-01 | 0.99 | 0.79 | 1.24 | 1.00 | 0.42 | 4.09 | 1 |
| Pelvic bone marrow fat | Hip osteoarthritis | -0.066 | 0.168 | 6.94E-01 | 0.94 | 0.67 | 1.30 | 1.00 | 0.31 | 4.53 | 1 |
| Pelvic bone marrow fat | Rheumatoid arthritis | -0.031 | 0.165 | 8.54E-01 | 0.97 | 0.70 | 1.34 | 1.00 | -0.01 | 21.99 | 1 |
| Pelvic bone marrow fat | Osteoporosis | 0.004 | 0.005 | 4.03E-01 | 1.00 | 0.99 | 1.01 | 1.00 | -0.71 | 35.61 | 1 |
| Pelvic bone marrow fat | Asthma | -0.137 | 0.096 | 1.54E-01 | 0.87 | 0.72 | 1.05 | 1.00 | -0.04 | 2.38 | 1 |
| Pelvic bone marrow fat | Psoriasis | 0.021 | 0.195 | 9.14E-01 | 1.02 | 0.70 | 1.50 | 1.00 | -0.01 | 6.22 | 1 |
| Pelvic bone marrow fat | Depression | -0.122 | 0.085 | 1.48E-01 | 0.88 | 0.75 | 1.04 | 1.00 | -0.33 | 19.63 | 1 |
| Pelvic bone marrow fat | Parkinson's disease | 0.011 | 0.259 | 9.67E-01 | 1.01 | 0.61 | 1.68 | 1.00 | -0.03 | 2.53 | 1 |
| Pelvic bone marrow fat | Alzheimer’s disease | 0.497 | 0.966 | 6.07E-01 | 1.64 | 0.25 | 10.92 | 1.00 | -0.32 | 3.21 | 1 |
| Pelvic bone marrow fat | Cholelithiasis | -0.208 | 0.134 | 1.19E-01 | 0.81 | 0.62 | 1.06 | 1.00 | 0.06 | 7.00 | 1 |
| Pelvic bone marrow fat | Gastroesophageal reflux disease | -0.163 | 0.078 | 3.59E-02 | 0.85 | 0.73 | 0.99 | 0.86 | -0.19 | 1.89 | 1 |
| Thigh bone marrow fat | Type 2 diabetes | 0.016 | 0.154 | 9.15E-01 | 1.02 | 0.75 | 1.37 | 0.99 | 0.11 | 2.71 | 1 |
| Thigh bone marrow fat | Polycystic ovary syndrome | -0.401 | 0.350 | 2.51E-01 | 0.67 | 0.34 | 1.33 | 0.99 | 0.50 | 6.52 | 1 |
| Thigh bone marrow fat | MASLD | -0.020 | 0.328 | 9.52E-01 | 0.98 | 0.52 | 1.86 | 0.99 | -0.37 | 0.65 | 1 |
| Thigh bone marrow fat | Chronic kidney disease | 0.232 | 0.103 | 2.44E-02 | 1.26 | 1.03 | 1.54 | 0.63 | 0.10 | 1.50 | 1 |
| Thigh bone marrow fat | Hypertension | 0.025 | 0.022 | 2.50E-01 | 1.03 | 0.98 | 1.07 | 0.99 | 0.09 | 3.15 | 1 |
| Thigh bone marrow fat | Coronary artery disease | 0.231 | 0.114 | 4.29E-02 | 1.26 | 1.01 | 1.58 | 0.99 | 0.13 | 1.51 | 1 |
| Thigh bone marrow fat | Stroke | 0.026 | 0.154 | 8.65E-01 | 1.03 | 0.76 | 1.39 | 0.99 | -0.05 | 14.01 | 1 |
| Thigh bone marrow fat | Myocardial infarction | 0.115 | 0.129 | 3.76E-01 | 1.12 | 0.87 | 1.45 | 0.99 | -0.05 | 2.20 | 1 |
| Thigh bone marrow fat | Aortic aneurysm | -0.029 | 0.141 | 8.35E-01 | 0.97 | 0.74 | 1.28 | 0.99 | -0.43 | 5.43 | 1 |
| Thigh bone marrow fat | Heart failure | 0.156 | 0.088 | 7.48E-02 | 1.17 | 0.98 | 1.39 | 0.99 | 0.13 | 1.29 | 1 |
| Thigh bone marrow fat | Atrial fibrillation | 0.103 | 0.098 | 2.90E-01 | 1.11 | 0.92 | 1.34 | 0.99 | 0.25 | 39.20 | 1 |
| Thigh bone marrow fat | Peripheral artery disease | 0.017 | 0.123 | 8.87E-01 | 1.02 | 0.80 | 1.29 | 0.99 | -0.10 | 2.94 | 1 |
| Thigh bone marrow fat | Deep vein thrombosis | 0.342 | 0.355 | 3.35E-01 | 1.41 | 0.70 | 2.82 | 0.99 | 0.17 | 2.24 | 1 |
| Thigh bone marrow fat | Pulmonary embolism | 0.150 | 0.281 | 5.93E-01 | 1.16 | 0.67 | 2.02 | 0.99 | 0.22 | 5.22 | 1 |
| Thigh bone marrow fat | Gout | 0.423 | 0.244 | 8.30E-02 | 1.53 | 0.95 | 2.46 | 0.99 | -0.16 | 9.03 | 1 |
| Thigh bone marrow fat | Knee osteoarthritis | -0.170 | 0.102 | 9.64E-02 | 0.84 | 0.69 | 1.03 | 0.99 | -0.64 | 4.09 | 1 |
| Thigh bone marrow fat | Hip osteoarthritis | -0.056 | 0.150 | 7.06E-01 | 0.95 | 0.71 | 1.27 | 0.99 | -0.56 | 4.53 | 1 |
| Thigh bone marrow fat | Rheumatoid arthritis | -0.001 | 0.153 | 9.94E-01 | 1.00 | 0.74 | 1.35 | 0.99 | -0.03 | 21.99 | 1 |
| Thigh bone marrow fat | Osteoporosis | 0.008 | 0.005 | 1.03E-01 | 1.01 | 1.00 | 1.02 | 0.99 | 0.71 | 35.61 | 1 |
| Thigh bone marrow fat | Asthma | 0.170 | 0.086 | 4.82E-02 | 1.19 | 1.00 | 1.40 | 0.99 | 0.13 | 2.38 | 1 |
| Thigh bone marrow fat | Psoriasis | -0.098 | 0.174 | 5.74E-01 | 0.91 | 0.64 | 1.28 | 0.99 | -0.03 | 6.22 | 1 |
| Thigh bone marrow fat | Depression | 0.094 | 0.075 | 2.09E-01 | 1.10 | 0.95 | 1.27 | 0.99 | 0.36 | 19.63 | 1 |
| Thigh bone marrow fat | Parkinson's disease | -0.253 | 0.230 | 2.71E-01 | 0.78 | 0.49 | 1.22 | 0.99 | 0.03 | 2.53 | 1 |
| Thigh bone marrow fat | Alzheimer’s disease | -0.552 | 0.858 | 5.20E-01 | 0.58 | 0.11 | 3.10 | 0.99 | 0.10 | 3.21 | 1 |
| Thigh bone marrow fat | Cholelithiasis | 0.214 | 0.121 | 7.83E-02 | 1.24 | 0.98 | 1.57 | 0.99 | -0.08 | 7.00 | 1 |
| Thigh bone marrow fat | Gastroesophageal reflux disease | 0.139 | 0.070 | 4.79E-02 | 1.15 | 1.00 | 1.32 | 0.99 | 0.18 | 1.89 | 1 |
| Vertebrae bone marrow fat | Type 2 diabetes | 0.113 | 0.146 | 4.39E-01 | 1.12 | 0.84 | 1.49 | 0.93 | 0.24 | 2.71 | 1 |
| Vertebrae bone marrow fat | Polycystic ovary syndrome | 0.536 | 0.317 | 9.12E-02 | 1.71 | 0.92 | 3.18 | 0.93 | 0.51 | 6.52 | 1 |
| Vertebrae bone marrow fat | MASLD | -0.203 | 0.297 | 4.96E-01 | 0.82 | 0.46 | 1.46 | 0.93 | -0.09 | 0.65 | 1 |
| Vertebrae bone marrow fat | Chronic kidney disease | 0.076 | 0.098 | 4.40E-01 | 1.08 | 0.89 | 1.31 | 0.93 | 0.00 | 1.50 | 1 |
| Vertebrae bone marrow fat | Hypertension | 0.016 | 0.021 | 4.37E-01 | 1.02 | 0.98 | 1.06 | 0.93 | 0.10 | 3.15 | 1 |
| Vertebrae bone marrow fat | Coronary artery disease | 0.158 | 0.105 | 1.35E-01 | 1.17 | 0.95 | 1.44 | 0.93 | 0.15 | 1.51 | 1 |
| Vertebrae bone marrow fat | Stroke | -0.146 | 0.149 | 3.27E-01 | 0.86 | 0.65 | 1.16 | 0.93 | 0.02 | 14.01 | 1 |
| Vertebrae bone marrow fat | Myocardial infarction | 0.172 | 0.120 | 1.51E-01 | 1.19 | 0.94 | 1.50 | 0.93 | 0.17 | 2.20 | 1 |
| Vertebrae bone marrow fat | Aortic aneurysm | 0.037 | 0.132 | 7.77E-01 | 1.04 | 0.80 | 1.34 | 0.93 | -0.05 | 5.43 | 1 |
| Vertebrae bone marrow fat | Heart failure | -0.037 | 0.081 | 6.44E-01 | 0.96 | 0.82 | 1.13 | 0.93 | 0.02 | 1.29 | 1 |
| Vertebrae bone marrow fat | Atrial fibrillation | -0.085 | 0.092 | 3.59E-01 | 0.92 | 0.77 | 1.10 | 0.93 | 0.02 | 39.20 | 1 |
| Vertebrae bone marrow fat | Peripheral artery disease | 0.100 | 0.120 | 4.06E-01 | 1.10 | 0.87 | 1.40 | 0.93 | 0.19 | 2.94 | 1 |
| Vertebrae bone marrow fat | Deep vein thrombosis | 0.236 | 0.322 | 4.63E-01 | 1.27 | 0.67 | 2.38 | 0.93 | 0.10 | 2.24 | 1 |
| Vertebrae bone marrow fat | Pulmonary embolism | 0.184 | 0.256 | 4.72E-01 | 1.20 | 0.73 | 1.98 | 0.93 | -0.06 | 5.22 | 1 |
| Vertebrae bone marrow fat | Gout | 0.683 | 0.220 | 1.93E-03 | 1.98 | 1.29 | 3.05 | 0.05 | 0.32 | 9.03 | 1 |
| Vertebrae bone marrow fat | Knee osteoarthritis | -0.015 | 0.094 | 8.73E-01 | 0.99 | 0.82 | 1.19 | 0.93 | -0.24 | 4.09 | 1 |
| Vertebrae bone marrow fat | Hip osteoarthritis | 0.143 | 0.136 | 2.92E-01 | 1.15 | 0.88 | 1.50 | 0.93 | 0.00 | 4.53 | 1 |
| Vertebrae bone marrow fat | Rheumatoid arthritis | -0.104 | 0.143 | 4.68E-01 | 0.90 | 0.68 | 1.19 | 0.93 | -0.17 | 21.99 | 1 |
| Vertebrae bone marrow fat | Osteoporosis | -0.006 | 0.004 | 1.56E-01 | 0.99 | 0.99 | 1.00 | 0.93 | 0.34 | 35.61 | 1 |
| Vertebrae bone marrow fat | Asthma | -0.028 | 0.080 | 7.25E-01 | 0.97 | 0.83 | 1.14 | 0.93 | -0.04 | 2.38 | 1 |
| Vertebrae bone marrow fat | Psoriasis | -0.052 | 0.159 | 7.45E-01 | 0.95 | 0.70 | 1.30 | 0.93 | -0.08 | 6.22 | 1 |
| Vertebrae bone marrow fat | Depression | 0.006 | 0.069 | 9.35E-01 | 1.01 | 0.88 | 1.15 | 0.93 | 0.03 | 19.63 | 1 |
| Vertebrae bone marrow fat | Parkinson's disease | 0.246 | 0.209 | 2.39E-01 | 1.28 | 0.85 | 1.93 | 0.93 | -0.05 | 2.53 | 1 |
| Vertebrae bone marrow fat | Alzheimer’s disease | -0.416 | 0.778 | 5.92E-01 | 0.66 | 0.14 | 3.03 | 0.93 | 0.25 | 3.21 | 1 |
| Vertebrae bone marrow fat | Cholelithiasis | -0.080 | 0.115 | 4.84E-01 | 0.92 | 0.74 | 1.16 | 0.93 | -0.22 | 7.00 | 1 |
| Vertebrae bone marrow fat | Gastroesophageal reflux disease | 0.130 | 0.066 | 4.87E-02 | 1.14 | 1.00 | 1.30 | 0.93 | 0.18 | 1.89 | 1 |

**Supplementary table. 11. Sensitivity results from different Mendelian randomization tests for the effect of IDPs on disease outcomes.** nSNP: number of SNPs used for the exposure; OR: odds ratio; 95% LCI: the lower confidence interval of the odds ratio; 95% UCI: the upper confidence interval of the odds ratio.

| Outcome | Exposure | nSNP | Method | Beta | SE | P.value | OR | OR_lci95 | OR_uci95 |
| --- | --- | --- | --- | --- | --- | --- | --- | --- | --- |
| Type 2 diabetes | Thigh subcutaneous AT | 21 | Inverse variance weighted | -0.468 | 0.146 | 1E-03 | 0.6 | 0.5 | 0.8 |
| Type 2 diabetes | Thigh subcutaneous AT | 21 | MR Egger | -0.788 | 0.474 | 1E-01 | 0.5 | 0.2 | 1.2 |
| Type 2 diabetes | Thigh subcutaneous AT | 21 | Weighted median | -0.507 | 0.051 | 2E-23 | 0.6 | 0.5 | 0.7 |
| Type 2 diabetes | Thigh subcutaneous AT | 21 | Simple mode | -0.64 | 0.088 | 4E-13 | 0.5 | 0.4 | 0.6 |
| Type 2 diabetes | Thigh subcutaneous AT | 21 | Weighted mode | -0.5 | 0.061 | 3E-16 | 0.6 | 0.5 | 0.7 |
| Polycystic ovary syndrome | Thigh subcutaneous AT | 21 | Inverse variance weighted | -0.245 | 0.115 | 3E-02 | 0.8 | 0.6 | 1 |
| Polycystic ovary syndrome | Thigh subcutaneous AT | 21 | MR Egger | -1.039 | 0.349 | 3E-03 | 0.4 | 0.2 | 0.7 |
| Polycystic ovary syndrome | Thigh subcutaneous AT | 21 | Weighted median | -0.181 | 0.158 | 3E-01 | 0.8 | 0.6 | 1.1 |
| Polycystic ovary syndrome | Thigh subcutaneous AT | 21 | Simple mode | -0.106 | 0.311 | 7E-01 | 0.9 | 0.5 | 1.7 |
| Polycystic ovary syndrome | Thigh subcutaneous AT | 21 | Weighted mode | -0.159 | 0.291 | 6E-01 | 0.9 | 0.5 | 1.5 |
| MASLD | Thigh subcutaneous AT | 21 | Inverse variance weighted | -0.244 | 0.123 | 5E-02 | 0.8 | 0.6 | 1 |
| MASLD | Thigh subcutaneous AT | 21 | MR Egger | -1.006 | 0.4 | 1E-02 | 0.4 | 0.2 | 0.8 |
| MASLD | Thigh subcutaneous AT | 21 | Weighted median | -0.359 | 0.112 | 1E-03 | 0.7 | 0.6 | 0.9 |
| MASLD | Thigh subcutaneous AT | 21 | Simple mode | -0.275 | 0.189 | 1E-01 | 0.8 | 0.5 | 1.1 |
| MASLD | Thigh subcutaneous AT | 21 | Weighted mode | -0.383 | 0.142 | 7E-03 | 0.7 | 0.5 | 0.9 |
| Hypertension | Thigh subcutaneous AT | 21 | Inverse variance weighted | -0.074 | 0.013 | 9E-09 | 0.9 | 0.9 | 1 |
| Hypertension | Thigh subcutaneous AT | 21 | MR Egger | -0.128 | 0.046 | 5E-03 | 0.9 | 0.8 | 1 |
| Hypertension | Thigh subcutaneous AT | 21 | Weighted median | -0.069 | 0.009 | 1E-14 | 0.9 | 0.9 | 1 |
| Hypertension | Thigh subcutaneous AT | 21 | Simple mode | -0.079 | 0.016 | 6E-07 | 0.9 | 0.9 | 1 |
| Hypertension | Thigh subcutaneous AT | 21 | Weighted mode | -0.075 | 0.016 | 2E-06 | 0.9 | 0.9 | 1 |
| Coronary artery disease | Thigh subcutaneous AT | 21 | Inverse variance weighted | -0.272 | 0.066 | 4E-05 | 0.8 | 0.7 | 0.9 |
| Coronary artery disease | Thigh subcutaneous AT | 21 | MR Egger | -0.486 | 0.225 | 3E-02 | 0.6 | 0.4 | 1 |
| Coronary artery disease | Thigh subcutaneous AT | 21 | Weighted median | -0.297 | 0.067 | 9E-06 | 0.7 | 0.7 | 0.8 |
| Coronary artery disease | Thigh subcutaneous AT | 21 | Simple mode | -0.261 | 0.121 | 3E-02 | 0.8 | 0.6 | 1 |
| Coronary artery disease | Thigh subcutaneous AT | 21 | Weighted mode | -0.299 | 0.109 | 6E-03 | 0.7 | 0.6 | 0.9 |
| Myocardial infarction | Thigh subcutaneous AT | 21 | Inverse variance weighted | -0.302 | 0.065 | 3E-06 | 0.7 | 0.7 | 0.8 |
| Myocardial infarction | Thigh subcutaneous AT | 21 | MR Egger | -0.379 | 0.218 | 8E-02 | 0.7 | 0.4 | 1 |
| Myocardial infarction | Thigh subcutaneous AT | 21 | Weighted median | -0.267 | 0.06 | 8E-06 | 0.8 | 0.7 | 0.9 |
| Myocardial infarction | Thigh subcutaneous AT | 21 | Simple mode | -0.274 | 0.11 | 1E-02 | 0.8 | 0.6 | 0.9 |
| Myocardial infarction | Thigh subcutaneous AT | 21 | Weighted mode | -0.283 | 0.089 | 1E-03 | 0.8 | 0.6 | 0.9 |
| Aortic aneurysm | Thigh subcutaneous AT | 21 | Inverse variance weighted | -0.341 | 0.091 | 2E-04 | 0.7 | 0.6 | 0.8 |
| Aortic aneurysm | Thigh subcutaneous AT | 21 | MR Egger | -0.788 | 0.282 | 5E-03 | 0.5 | 0.3 | 0.8 |
| Aortic aneurysm | Thigh subcutaneous AT | 21 | Weighted median | -0.323 | 0.109 | 3E-03 | 0.7 | 0.6 | 0.9 |
| Aortic aneurysm | Thigh subcutaneous AT | 21 | Simple mode | -0.104 | 0.216 | 6E-01 | 0.9 | 0.6 | 1.4 |
| Aortic aneurysm | Thigh subcutaneous AT | 21 | Weighted mode | -0.197 | 0.198 | 3E-01 | 0.8 | 0.6 | 1.2 |
| Peripheral artery disease | Thigh subcutaneous AT | 21 | Inverse variance weighted | -0.214 | 0.068 | 2E-03 | 0.8 | 0.7 | 0.9 |
| Peripheral artery disease | Thigh subcutaneous AT | 21 | MR Egger | -0.564 | 0.22 | 1E-02 | 0.6 | 0.4 | 0.9 |
| Peripheral artery disease | Thigh subcutaneous AT | 21 | Weighted median | -0.222 | 0.082 | 7E-03 | 0.8 | 0.7 | 0.9 |
| Peripheral artery disease | Thigh subcutaneous AT | 21 | Simple mode | -0.222 | 0.145 | 1E-01 | 0.8 | 0.6 | 1.1 |
| Peripheral artery disease | Thigh subcutaneous AT | 21 | Weighted mode | -0.254 | 0.119 | 3E-02 | 0.8 | 0.6 | 1 |
| Pulmonary embolism | Thigh subcutaneous AT | 21 | Inverse variance weighted | 0.232 | 0.089 | 9E-03 | 1.3 | 1.1 | 1.5 |
| Pulmonary embolism | Thigh subcutaneous AT | 21 | MR Egger | 0.105 | 0.291 | 7E-01 | 1.1 | 0.6 | 2 |
| Pulmonary embolism | Thigh subcutaneous AT | 21 | Weighted median | 0.259 | 0.097 | 7E-03 | 1.3 | 1.1 | 1.6 |
| Pulmonary embolism | Thigh subcutaneous AT | 21 | Simple mode | 0.107 | 0.186 | 6E-01 | 1.1 | 0.8 | 1.6 |
| Pulmonary embolism | Thigh subcutaneous AT | 21 | Weighted mode | 0.164 | 0.164 | 3E-01 | 1.2 | 0.9 | 1.6 |
| Knee osteoarthritis | Thigh subcutaneous AT | 21 | Inverse variance weighted | 0.285 | 0.066 | 1E-05 | 1.3 | 1.2 | 1.5 |
| Knee osteoarthritis | Thigh subcutaneous AT | 21 | MR Egger | 0.018 | 0.215 | 9E-01 | 1 | 0.7 | 1.6 |
| Knee osteoarthritis | Thigh subcutaneous AT | 21 | Weighted median | 0.165 | 0.052 | 1E-03 | 1.2 | 1.1 | 1.3 |
| Knee osteoarthritis | Thigh subcutaneous AT | 21 | Simple mode | 0.16 | 0.094 | 9E-02 | 1.2 | 1 | 1.4 |
| Knee osteoarthritis | Thigh subcutaneous AT | 21 | Weighted mode | 0.131 | 0.086 | 1E-01 | 1.1 | 1 | 1.4 |
| Osteoporosis | Thigh subcutaneous AT | 21 | Inverse variance weighted | 0.006 | 0.002 | 2E-03 | 1 | 1 | 1 |
| Osteoporosis | Thigh subcutaneous AT | 21 | MR Egger | 0.025 | 0.006 | 2E-05 | 1 | 1 | 1 |
| Osteoporosis | Thigh subcutaneous AT | 21 | Weighted median | 0.007 | 0.002 | 4E-04 | 1 | 1 | 1 |
| Osteoporosis | Thigh subcutaneous AT | 21 | Simple mode | 0.014 | 0.006 | 2E-02 | 1 | 1 | 1 |
| Osteoporosis | Thigh subcutaneous AT | 21 | Weighted mode | 0.013 | 0.005 | 9E-03 | 1 | 1 | 1 |
| Type 2 diabetes | Thigh intermuscular AT | 13 | Inverse variance weighted | -0.619 | 0.093 | 3E-11 | 0.5 | 0.4 | 0.6 |
| Type 2 diabetes | Thigh intermuscular AT | 13 | MR Egger | -1.187 | 0.448 | 8E-03 | 0.3 | 0.1 | 0.7 |
| Type 2 diabetes | Thigh intermuscular AT | 13 | Weighted median | -0.413 | 0.053 | 4E-15 | 0.7 | 0.6 | 0.7 |
| Type 2 diabetes | Thigh intermuscular AT | 13 | Simple mode | -0.5 | 0.076 | 6E-11 | 0.6 | 0.5 | 0.7 |
| Type 2 diabetes | Thigh intermuscular AT | 13 | Weighted mode | -0.401 | 0.065 | 6E-10 | 0.7 | 0.6 | 0.8 |
| Polycystic ovary syndrome | Thigh intermuscular AT | 13 | Inverse variance weighted | -0.5 | 0.147 | 7E-04 | 0.6 | 0.5 | 0.8 |
| Polycystic ovary syndrome | Thigh intermuscular AT | 13 | MR Egger | -1.06 | 0.711 | 1E-01 | 0.3 | 0.1 | 1.4 |
| Polycystic ovary syndrome | Thigh intermuscular AT | 13 | Weighted median | -0.565 | 0.183 | 2E-03 | 0.6 | 0.4 | 0.8 |
| Polycystic ovary syndrome | Thigh intermuscular AT | 13 | Simple mode | -0.743 | 0.361 | 4E-02 | 0.5 | 0.2 | 1 |
| Polycystic ovary syndrome | Thigh intermuscular AT | 13 | Weighted mode | -0.783 | 0.356 | 3E-02 | 0.5 | 0.2 | 0.9 |
| MASLD | Thigh intermuscular AT | 13 | Inverse variance weighted | -0.466 | 0.084 | 3E-08 | 0.6 | 0.5 | 0.7 |
| MASLD | Thigh intermuscular AT | 13 | MR Egger | -0.718 | 0.405 | 8E-02 | 0.5 | 0.2 | 1.1 |
| MASLD | Thigh intermuscular AT | 13 | Weighted median | -0.497 | 0.11 | 6E-06 | 0.6 | 0.5 | 0.8 |
| MASLD | Thigh intermuscular AT | 13 | Simple mode | -0.472 | 0.19 | 1E-02 | 0.6 | 0.4 | 0.9 |
| MASLD | Thigh intermuscular AT | 13 | Weighted mode | -0.468 | 0.186 | 1E-02 | 0.6 | 0.4 | 0.9 |
| Hypertension | Thigh intermuscular AT | 13 | Inverse variance weighted | -0.065 | 0.011 | 2E-09 | 0.9 | 0.9 | 1 |
| Hypertension | Thigh intermuscular AT | 13 | MR Egger | -0.089 | 0.054 | 1E-01 | 0.9 | 0.8 | 1 |
| Hypertension | Thigh intermuscular AT | 13 | Weighted median | -0.057 | 0.009 | 1E-10 | 0.9 | 0.9 | 1 |
| Hypertension | Thigh intermuscular AT | 13 | Simple mode | -0.067 | 0.016 | 2E-05 | 0.9 | 0.9 | 1 |
| Hypertension | Thigh intermuscular AT | 13 | Weighted mode | -0.066 | 0.014 | 2E-06 | 0.9 | 0.9 | 1 |
| Coronary artery disease | Thigh intermuscular AT | 13 | Inverse variance weighted | -0.284 | 0.05 | 1E-08 | 0.8 | 0.7 | 0.8 |
| Coronary artery disease | Thigh intermuscular AT | 13 | MR Egger | -0.31 | 0.244 | 2E-01 | 0.7 | 0.5 | 1.2 |
| Coronary artery disease | Thigh intermuscular AT | 13 | Weighted median | -0.218 | 0.065 | 8E-04 | 0.8 | 0.7 | 0.9 |
| Coronary artery disease | Thigh intermuscular AT | 13 | Simple mode | -0.203 | 0.122 | 1E-01 | 0.8 | 0.6 | 1 |
| Coronary artery disease | Thigh intermuscular AT | 13 | Weighted mode | -0.193 | 0.12 | 1E-01 | 0.8 | 0.7 | 1 |
| Myocardial infarction | Thigh intermuscular AT | 13 | Inverse variance weighted | -0.215 | 0.055 | 1E-04 | 0.8 | 0.7 | 0.9 |
| Myocardial infarction | Thigh intermuscular AT | 13 | MR Egger | 0.194 | 0.253 | 4E-01 | 1.2 | 0.7 | 2 |
| Myocardial infarction | Thigh intermuscular AT | 13 | Weighted median | -0.097 | 0.063 | 1E-01 | 0.9 | 0.8 | 1 |
| Myocardial infarction | Thigh intermuscular AT | 13 | Simple mode | -0.168 | 0.108 | 1E-01 | 0.8 | 0.7 | 1 |
| Myocardial infarction | Thigh intermuscular AT | 13 | Weighted mode | -0.057 | 0.098 | 6E-01 | 0.9 | 0.8 | 1.1 |
| Peripheral artery disease | Thigh intermuscular AT | 14 | Inverse variance weighted | -0.181 | 0.076 | 2E-02 | 0.8 | 0.7 | 1 |
| Peripheral artery disease | Thigh intermuscular AT | 14 | MR Egger | -0.49 | 0.378 | 2E-01 | 0.6 | 0.3 | 1.3 |
| Peripheral artery disease | Thigh intermuscular AT | 14 | Weighted median | -0.249 | 0.085 | 3E-03 | 0.8 | 0.7 | 0.9 |
| Peripheral artery disease | Thigh intermuscular AT | 14 | Simple mode | -0.249 | 0.138 | 7E-02 | 0.8 | 0.6 | 1 |
| Peripheral artery disease | Thigh intermuscular AT | 14 | Weighted mode | -0.278 | 0.131 | 3E-02 | 0.8 | 0.6 | 1 |
| Asthma | Thigh intermuscular AT | 13 | Inverse variance weighted | 0.083 | 0.036 | 2E-02 | 1.1 | 1 | 1.2 |
| Asthma | Thigh intermuscular AT | 13 | MR Egger | 0.15 | 0.172 | 4E-01 | 1.2 | 0.8 | 1.6 |
| Asthma | Thigh intermuscular AT | 13 | Weighted median | 0.087 | 0.045 | 5E-02 | 1.1 | 1 | 1.2 |
| Asthma | Thigh intermuscular AT | 13 | Simple mode | 0.102 | 0.077 | 2E-01 | 1.1 | 1 | 1.3 |
| Asthma | Thigh intermuscular AT | 13 | Weighted mode | 0.1 | 0.072 | 2E-01 | 1.1 | 1 | 1.3 |
| Knee osteoarthritis | Visceral AT | 6 | Inverse variance weighted | 0.328 | 0.164 | 5E-02 | 1.4 | 1 | 1.9 |
| Knee osteoarthritis | Visceral AT | 6 | MR Egger | -3.044 | 0.831 | 2E-04 | 0 | 0 | 0.2 |
| Knee osteoarthritis | Visceral AT | 6 | Weighted median | 0.125 | 0.091 | 2E-01 | 1.1 | 0.9 | 1.4 |
| Knee osteoarthritis | Visceral AT | 6 | Simple mode | 0.042 | 0.113 | 7E-01 | 1 | 0.8 | 1.3 |
| Knee osteoarthritis | Visceral AT | 6 | Weighted mode | 0.039 | 0.097 | 7E-01 | 1 | 0.9 | 1.3 |
| Asthma | Visceral AT | 6 | Inverse variance weighted | 0.266 | 0.055 | 2E-06 | 1.3 | 1.2 | 1.5 |
| Asthma | Visceral AT | 6 | MR Egger | 0.256 | 0.455 | 6E-01 | 1.3 | 0.5 | 3.2 |
| Asthma | Visceral AT | 6 | Weighted median | 0.224 | 0.072 | 2E-03 | 1.3 | 1.1 | 1.4 |
| Asthma | Visceral AT | 6 | Simple mode | 0.149 | 0.119 | 2E-01 | 1.2 | 0.9 | 1.5 |
| Asthma | Visceral AT | 6 | Weighted mode | 0.147 | 0.115 | 2E-01 | 1.2 | 0.9 | 1.4 |
| Cholelithiasis | Visceral AT | 6 | Inverse variance weighted | 0.4 | 0.118 | 7E-04 | 1.5 | 1.2 | 1.9 |
| Cholelithiasis | Visceral AT | 6 | MR Egger | -0.023 | 1.038 | 1E+00 | 1 | 0.1 | 7.5 |
| Cholelithiasis | Visceral AT | 6 | Weighted median | 0.271 | 0.085 | 1E-03 | 1.3 | 1.1 | 1.5 |
| Cholelithiasis | Visceral AT | 6 | Simple mode | 0.29 | 0.121 | 2E-02 | 1.3 | 1.1 | 1.7 |
| Cholelithiasis | Visceral AT | 6 | Weighted mode | 0.269 | 0.108 | 1E-02 | 1.3 | 1.1 | 1.6 |
| Type 2 diabetes | Liver PDFF | 9 | Inverse variance weighted | 0.194 | 0.077 | 1E-02 | 1.2 | 1 | 1.4 |
| Type 2 diabetes | Liver PDFF | 9 | MR Egger | 0.414 | 0.117 | 4E-04 | 1.5 | 1.2 | 1.9 |
| Type 2 diabetes | Liver PDFF | 9 | Weighted median | 0.204 | 0.024 | 9E-18 | 1.2 | 1.2 | 1.3 |
| Type 2 diabetes | Liver PDFF | 9 | Simple mode | 0.304 | 0.055 | 3E-08 | 1.4 | 1.2 | 1.5 |
| Type 2 diabetes | Liver PDFF | 9 | Weighted mode | 0.184 | 0.02 | 2E-19 | 1.2 | 1.2 | 1.3 |
| MASLD | Liver PDFF | 9 | Inverse variance weighted | 1.335 | 0.077 | 1E-66 | 3.8 | 3.3 | 4.4 |
| MASLD | Liver PDFF | 9 | MR Egger | 1.233 | 0.134 | 4E-20 | 3.4 | 2.6 | 4.5 |
| MASLD | Liver PDFF | 9 | Weighted median | 1.386 | 0.077 | 1E-72 | 4 | 3.4 | 4.6 |
| MASLD | Liver PDFF | 9 | Simple mode | 1.315 | 0.137 | 9E-22 | 3.7 | 2.8 | 4.9 |
| MASLD | Liver PDFF | 9 | Weighted mode | 1.327 | 0.079 | 1E-63 | 3.8 | 3.2 | 4.4 |
| Myocardial infarction | Liver PDFF | 9 | Inverse variance weighted | -0.206 | 0.073 | 5E-03 | 0.8 | 0.7 | 0.9 |
| Myocardial infarction | Liver PDFF | 9 | MR Egger | -0.431 | 0.103 | 3E-05 | 0.7 | 0.5 | 0.8 |
| Myocardial infarction | Liver PDFF | 9 | Weighted median | -0.199 | 0.032 | 7E-10 | 0.8 | 0.8 | 0.9 |
| Myocardial infarction | Liver PDFF | 9 | Simple mode | -0.121 | 0.077 | 1E-01 | 0.9 | 0.8 | 1 |
| Myocardial infarction | Liver PDFF | 9 | Weighted mode | -0.198 | 0.031 | 2E-10 | 0.8 | 0.8 | 0.9 |
| Peripheral artery disease | Liver PDFF | 9 | Inverse variance weighted | -0.144 | 0.044 | 1E-03 | 0.9 | 0.8 | 0.9 |
| Peripheral artery disease | Liver PDFF | 9 | MR Egger | -0.292 | 0.063 | 3E-06 | 0.7 | 0.7 | 0.8 |
| Peripheral artery disease | Liver PDFF | 9 | Weighted median | -0.186 | 0.041 | 6E-06 | 0.8 | 0.8 | 0.9 |
| Peripheral artery disease | Liver PDFF | 9 | Simple mode | -0.107 | 0.082 | 2E-01 | 0.9 | 0.8 | 1.1 |
| Peripheral artery disease | Liver PDFF | 9 | Weighted mode | -0.182 | 0.041 | 8E-06 | 0.8 | 0.8 | 0.9 |
| Osteoporosis | Liver PDFF | 9 | Inverse variance weighted | 0.003 | 0.001 | 3E-03 | 1 | 1 | 1 |
| Osteoporosis | Liver PDFF | 9 | MR Egger | 0.002 | 0.002 | 3E-01 | 1 | 1 | 1 |
| Osteoporosis | Liver PDFF | 9 | Weighted median | 0.003 | 0.001 | 3E-03 | 1 | 1 | 1 |
| Osteoporosis | Liver PDFF | 9 | Simple mode | 0.003 | 0.002 | 1E-01 | 1 | 1 | 1 |
| Osteoporosis | Liver PDFF | 9 | Weighted mode | 0.003 | 0.001 | 3E-03 | 1 | 1 | 1 |
| Psoriasis | Liver PDFF | 9 | Inverse variance weighted | 0.117 | 0.048 | 2E-02 | 1.1 | 1 | 1.2 |
| Psoriasis | Liver PDFF | 9 | MR Egger | 0.095 | 0.082 | 2E-01 | 1.1 | 0.9 | 1.3 |
| Psoriasis | Liver PDFF | 9 | Weighted median | 0.137 | 0.047 | 3E-03 | 1.1 | 1 | 1.3 |
| Psoriasis | Liver PDFF | 9 | Simple mode | 0.099 | 0.075 | 2E-01 | 1.1 | 1 | 1.3 |
| Psoriasis | Liver PDFF | 9 | Weighted mode | 0.138 | 0.045 | 2E-03 | 1.1 | 1.1 | 1.3 |
| Parkinson's disease | Liver PDFF | 9 | Inverse variance weighted | -0.12 | 0.06 | 5E-02 | 0.9 | 0.8 | 1 |
| Parkinson's disease | Liver PDFF | 9 | MR Egger | 0.09 | 0.093 | 3E-01 | 1.1 | 0.9 | 1.3 |
| Parkinson's disease | Liver PDFF | 9 | Weighted median | -0.057 | 0.057 | 3E-01 | 0.9 | 0.8 | 1.1 |
| Parkinson's disease | Liver PDFF | 9 | Simple mode | -0.196 | 0.12 | 1E-01 | 0.8 | 0.6 | 1 |
| Parkinson's disease | Liver PDFF | 9 | Weighted mode | -0.046 | 0.056 | 4E-01 | 1 | 0.9 | 1.1 |
| Atrial fibrillation | Pancreas PDFF | 13 | Inverse variance weighted | 0.119 | 0.05 | 2E-02 | 1.1 | 1 | 1.2 |
| Atrial fibrillation | Pancreas PDFF | 13 | MR Egger | 0.138 | 0.216 | 5E-01 | 1.1 | 0.8 | 1.8 |
| Atrial fibrillation | Pancreas PDFF | 13 | Weighted median | 0.102 | 0.046 | 3E-02 | 1.1 | 1 | 1.2 |
| Atrial fibrillation | Pancreas PDFF | 13 | Simple mode | 0.106 | 0.069 | 1E-01 | 1.1 | 1 | 1.3 |
| Atrial fibrillation | Pancreas PDFF | 13 | Weighted mode | 0.084 | 0.056 | 1E-01 | 1.1 | 1 | 1.2 |
| Pulmonary embolism | Pancreas PDFF | 13 | Inverse variance weighted | 0.577 | 0.256 | 2E-02 | 1.8 | 1.1 | 2.9 |
| Pulmonary embolism | Pancreas PDFF | 13 | MR Egger | 1.147 | 0.989 | 2E-01 | 3.2 | 0.5 | 21.9 |
| Pulmonary embolism | Pancreas PDFF | 13 | Weighted median | 0.247 | 0.106 | 2E-02 | 1.3 | 1 | 1.6 |
| Pulmonary embolism | Pancreas PDFF | 13 | Simple mode | 0.218 | 0.147 | 1E-01 | 1.2 | 0.9 | 1.7 |
| Pulmonary embolism | Pancreas PDFF | 13 | Weighted mode | 0.178 | 0.12 | 1E-01 | 1.2 | 0.9 | 1.5 |
| Parkinson's disease | Pancreas PDFF | 13 | Inverse variance weighted | -0.181 | 0.091 | 5E-02 | 0.8 | 0.7 | 1 |
| Parkinson's disease | Pancreas PDFF | 13 | MR Egger | 0.286 | 0.396 | 5E-01 | 1.3 | 0.6 | 2.9 |
| Parkinson's disease | Pancreas PDFF | 13 | Weighted median | -0.089 | 0.115 | 4E-01 | 0.9 | 0.7 | 1.1 |
| Parkinson's disease | Pancreas PDFF | 13 | Simple mode | -0.268 | 0.191 | 2E-01 | 0.8 | 0.5 | 1.1 |
| Parkinson's disease | Pancreas PDFF | 13 | Weighted mode | -0.016 | 0.145 | 9E-01 | 1 | 0.7 | 1.3 |
| Cholelithiasis | Pancreas PDFF | 13 | Inverse variance weighted | 0.184 | 0.091 | 4E-02 | 1.2 | 1 | 1.4 |
| Cholelithiasis | Pancreas PDFF | 13 | MR Egger | 0.124 | 0.374 | 7E-01 | 1.1 | 0.5 | 2.4 |
| Cholelithiasis | Pancreas PDFF | 13 | Weighted median | 0.057 | 0.049 | 2E-01 | 1.1 | 1 | 1.2 |
| Cholelithiasis | Pancreas PDFF | 13 | Simple mode | -0.047 | 0.064 | 5E-01 | 1 | 0.8 | 1.1 |
| Cholelithiasis | Pancreas PDFF | 13 | Weighted mode | 0.028 | 0.052 | 6E-01 | 1 | 0.9 | 1.1 |
| Myocardial infarction | Paraspinal AT | 8 | Inverse variance weighted | -0.131 | 0.048 | 7E-03 | 0.9 | 0.8 | 1 |
| Myocardial infarction | Paraspinal AT | 8 | MR Egger | -0.075 | 0.204 | 7E-01 | 0.9 | 0.6 | 1.4 |
| Myocardial infarction | Paraspinal AT | 8 | Weighted median | -0.091 | 0.064 | 2E-01 | 0.9 | 0.8 | 1 |
| Myocardial infarction | Paraspinal AT | 8 | Simple mode | -0.174 | 0.104 | 9E-02 | 0.8 | 0.7 | 1 |
| Myocardial infarction | Paraspinal AT | 8 | Weighted mode | -0.081 | 0.093 | 4E-01 | 0.9 | 0.8 | 1.1 |
| Atrial fibrillation | Paraspinal AT | 8 | Inverse variance weighted | 0.155 | 0.064 | 2E-02 | 1.2 | 1 | 1.3 |
| Atrial fibrillation | Paraspinal AT | 8 | MR Egger | -0.208 | 0.254 | 4E-01 | 0.8 | 0.5 | 1.3 |
| Atrial fibrillation | Paraspinal AT | 8 | Weighted median | 0.04 | 0.051 | 4E-01 | 1 | 0.9 | 1.1 |
| Atrial fibrillation | Paraspinal AT | 8 | Simple mode | 0.035 | 0.075 | 6E-01 | 1 | 0.9 | 1.2 |
| Atrial fibrillation | Paraspinal AT | 8 | Weighted mode | 0.017 | 0.055 | 8E-01 | 1 | 0.9 | 1.1 |
| Pulmonary embolism | Paraspinal AT | 8 | Inverse variance weighted | 0.167 | 0.078 | 3E-02 | 1.2 | 1 | 1.4 |
| Pulmonary embolism | Paraspinal AT | 8 | MR Egger | 0.61 | 0.308 | 5E-02 | 1.8 | 1 | 3.4 |
| Pulmonary embolism | Paraspinal AT | 8 | Weighted median | 0.114 | 0.1 | 3E-01 | 1.1 | 0.9 | 1.4 |
| Pulmonary embolism | Paraspinal AT | 8 | Simple mode | 0.147 | 0.167 | 4E-01 | 1.2 | 0.8 | 1.6 |
| Pulmonary embolism | Paraspinal AT | 8 | Weighted mode | 0.154 | 0.151 | 3E-01 | 1.2 | 0.9 | 1.6 |
| Heart failure | Pelvic bone marrow fat | 27 | Inverse variance weighted | -0.066 | 0.034 | 5E-02 | 0.9 | 0.9 | 1 |
| Heart failure | Pelvic bone marrow fat | 27 | MR Egger | 0.069 | 0.1 | 5E-01 | 1.1 | 0.9 | 1.3 |
| Heart failure | Pelvic bone marrow fat | 27 | Weighted median | -0.057 | 0.037 | 1E-01 | 0.9 | 0.9 | 1 |
| Heart failure | Pelvic bone marrow fat | 27 | Simple mode | -0.149 | 0.078 | 6E-02 | 0.9 | 0.7 | 1 |
| Heart failure | Pelvic bone marrow fat | 27 | Weighted mode | -0.071 | 0.067 | 3E-01 | 0.9 | 0.8 | 1.1 |
| Gout | Pelvic bone marrow fat | 27 | Inverse variance weighted | -0.203 | 0.073 | 5E-03 | 0.8 | 0.7 | 0.9 |
| Gout | Pelvic bone marrow fat | 27 | MR Egger | -0.317 | 0.232 | 2E-01 | 0.7 | 0.5 | 1.1 |
| Gout | Pelvic bone marrow fat | 27 | Weighted median | -0.226 | 0.096 | 2E-02 | 0.8 | 0.7 | 1 |
| Gout | Pelvic bone marrow fat | 27 | Simple mode | -0.228 | 0.171 | 2E-01 | 0.8 | 0.6 | 1.1 |
| Gout | Pelvic bone marrow fat | 27 | Weighted mode | -0.236 | 0.13 | 7E-02 | 0.8 | 0.6 | 1 |
| Knee osteoarthritis | Pelvic bone marrow fat | 27 | Inverse variance weighted | -0.139 | 0.049 | 5E-03 | 0.9 | 0.8 | 1 |
| Knee osteoarthritis | Pelvic bone marrow fat | 27 | MR Egger | 0.05 | 0.153 | 7E-01 | 1.1 | 0.8 | 1.4 |
| Knee osteoarthritis | Pelvic bone marrow fat | 27 | Weighted median | -0.101 | 0.036 | 5E-03 | 0.9 | 0.8 | 1 |
| Knee osteoarthritis | Pelvic bone marrow fat | 27 | Simple mode | -0.058 | 0.065 | 4E-01 | 0.9 | 0.8 | 1.1 |
| Knee osteoarthritis | Pelvic bone marrow fat | 27 | Weighted mode | -0.108 | 0.049 | 3E-02 | 0.9 | 0.8 | 1 |
| Hip osteoarthritis | Pelvic bone marrow fat | 27 | Inverse variance weighted | -0.111 | 0.046 | 2E-02 | 0.9 | 0.8 | 1 |
| Hip osteoarthritis | Pelvic bone marrow fat | 27 | MR Egger | 0.177 | 0.137 | 2E-01 | 1.2 | 0.9 | 1.6 |
| Hip osteoarthritis | Pelvic bone marrow fat | 27 | Weighted median | -0.058 | 0.048 | 2E-01 | 0.9 | 0.9 | 1 |
| Hip osteoarthritis | Pelvic bone marrow fat | 27 | Simple mode | -0.121 | 0.102 | 2E-01 | 0.9 | 0.7 | 1.1 |
| Hip osteoarthritis | Pelvic bone marrow fat | 27 | Weighted mode | -0.002 | 0.067 | 1E+00 | 1 | 0.9 | 1.1 |
| Rheumatoid arthritis | Pelvic bone marrow fat | 27 | Inverse variance weighted | -0.17 | 0.079 | 3E-02 | 0.8 | 0.7 | 1 |
| Rheumatoid arthritis | Pelvic bone marrow fat | 27 | MR Egger | -0.137 | 0.251 | 6E-01 | 0.9 | 0.5 | 1.4 |
| Rheumatoid arthritis | Pelvic bone marrow fat | 27 | Weighted median | -0.088 | 0.053 | 1E-01 | 0.9 | 0.8 | 1 |
| Rheumatoid arthritis | Pelvic bone marrow fat | 27 | Simple mode | -0.115 | 0.09 | 2E-01 | 0.9 | 0.7 | 1.1 |
| Rheumatoid arthritis | Pelvic bone marrow fat | 27 | Weighted mode | -0.033 | 0.063 | 6E-01 | 1 | 0.9 | 1.1 |
| Osteoporosis | Pelvic bone marrow fat | 27 | Inverse variance weighted | 0.007 | 0.003 | 2E-02 | 1 | 1 | 1 |
| Osteoporosis | Pelvic bone marrow fat | 27 | MR Egger | 0.01 | 0.008 | 2E-01 | 1 | 1 | 1 |
| Osteoporosis | Pelvic bone marrow fat | 27 | Weighted median | 0.002 | 0.002 | 3E-01 | 1 | 1 | 1 |
| Osteoporosis | Pelvic bone marrow fat | 27 | Simple mode | -0.003 | 0.002 | 1E-01 | 1 | 1 | 1 |
| Osteoporosis | Pelvic bone marrow fat | 27 | Weighted mode | -0.002 | 0.003 | 5E-01 | 1 | 1 | 1 |
| Cholelithiasis | Pelvic bone marrow fat | 27 | Inverse variance weighted | -0.094 | 0.035 | 7E-03 | 0.9 | 0.8 | 1 |
| Cholelithiasis | Pelvic bone marrow fat | 27 | MR Egger | 0.014 | 0.108 | 9E-01 | 1 | 0.8 | 1.3 |
| Cholelithiasis | Pelvic bone marrow fat | 27 | Weighted median | -0.003 | 0.034 | 9E-01 | 1 | 0.9 | 1.1 |
| Cholelithiasis | Pelvic bone marrow fat | 27 | Simple mode | 0.026 | 0.059 | 7E-01 | 1 | 0.9 | 1.2 |
| Cholelithiasis | Pelvic bone marrow fat | 27 | Weighted mode | 0.048 | 0.047 | 3E-01 | 1 | 1 | 1.1 |
| Atrial fibrillation | Thigh bone marrow fat | 29 | Inverse variance weighted | 0.102 | 0.047 | 3E-02 | 1.1 | 1 | 1.2 |
| Atrial fibrillation | Thigh bone marrow fat | 29 | MR Egger | 0.234 | 0.163 | 2E-01 | 1.3 | 0.9 | 1.7 |
| Atrial fibrillation | Thigh bone marrow fat | 29 | Weighted median | 0.001 | 0.041 | 1E+00 | 1 | 0.9 | 1.1 |
| Atrial fibrillation | Thigh bone marrow fat | 29 | Simple mode | -0.082 | 0.095 | 4E-01 | 0.9 | 0.8 | 1.1 |
| Atrial fibrillation | Thigh bone marrow fat | 29 | Weighted mode | -0.087 | 0.072 | 2E-01 | 0.9 | 0.8 | 1.1 |
| Knee osteoarthritis | Thigh bone marrow fat | 29 | Inverse variance weighted | -0.206 | 0.041 | 6E-07 | 0.8 | 0.8 | 0.9 |
| Knee osteoarthritis | Thigh bone marrow fat | 29 | MR Egger | -0.325 | 0.135 | 2E-02 | 0.7 | 0.6 | 0.9 |
| Knee osteoarthritis | Thigh bone marrow fat | 29 | Weighted median | -0.179 | 0.038 | 3E-06 | 0.8 | 0.8 | 0.9 |
| Knee osteoarthritis | Thigh bone marrow fat | 29 | Simple mode | -0.185 | 0.075 | 1E-02 | 0.8 | 0.7 | 1 |
| Knee osteoarthritis | Thigh bone marrow fat | 29 | Weighted mode | -0.186 | 0.065 | 4E-03 | 0.8 | 0.7 | 0.9 |
| Hip osteoarthritis | Thigh bone marrow fat | 29 | Inverse variance weighted | -0.133 | 0.053 | 1E-02 | 0.9 | 0.8 | 1 |
| Hip osteoarthritis | Thigh bone marrow fat | 29 | MR Egger | -0.177 | 0.175 | 3E-01 | 0.8 | 0.6 | 1.2 |
| Hip osteoarthritis | Thigh bone marrow fat | 29 | Weighted median | -0.163 | 0.05 | 1E-03 | 0.8 | 0.8 | 0.9 |
| Hip osteoarthritis | Thigh bone marrow fat | 29 | Simple mode | -0.155 | 0.088 | 8E-02 | 0.9 | 0.7 | 1 |
| Hip osteoarthritis | Thigh bone marrow fat | 29 | Weighted mode | -0.159 | 0.082 | 5E-02 | 0.9 | 0.7 | 1 |
| Osteoporosis | Thigh bone marrow fat | 29 | Inverse variance weighted | 0.011 | 0.003 | 2E-04 | 1 | 1 | 1 |
| Osteoporosis | Thigh bone marrow fat | 29 | MR Egger | 0.022 | 0.009 | 1E-02 | 1 | 1 | 1 |
| Osteoporosis | Thigh bone marrow fat | 29 | Weighted median | 0.006 | 0.002 | 2E-03 | 1 | 1 | 1 |
| Osteoporosis | Thigh bone marrow fat | 29 | Simple mode | 0 | 0.004 | 1E+00 | 1 | 1 | 1 |
| Osteoporosis | Thigh bone marrow fat | 29 | Weighted mode | -0.001 | 0.003 | 7E-01 | 1 | 1 | 1 |
| Hip osteoarthritis | Vertebrae bone marrow fat | 5 | Inverse variance weighted | 0.227 | 0.075 | 2E-03 | 1.3 | 1.1 | 1.5 |
| Hip osteoarthritis | Vertebrae bone marrow fat | 5 | MR Egger | -0.146 | 0.794 | 9E-01 | 0.9 | 0.2 | 4.1 |
| Hip osteoarthritis | Vertebrae bone marrow fat | 5 | Weighted median | 0.195 | 0.081 | 2E-02 | 1.2 | 1 | 1.4 |
| Hip osteoarthritis | Vertebrae bone marrow fat | 5 | Simple mode | 0.186 | 0.139 | 2E-01 | 1.2 | 0.9 | 1.6 |
| Hip osteoarthritis | Vertebrae bone marrow fat | 5 | Weighted mode | 0.034 | 0.14 | 8E-01 | 1 | 0.8 | 1.4 |

**Supplementary table 12. Pleiotropy and Heterogeneity test results.** The horizontal pleiotropy and heterogeneity tests for the effect of ten IDPs on the risk of 26 disease outcomes from FinnGen and published GWAS. The table shows Cochran's Q, Q pvalue, MR-Egger intercept, and the Egger-intercept pvalue.

| **Source** | **Exposure** | **Outcome** | **Method** | **Q** | **Q_df** | **Q_pval** | **Egger_intercept** | **P-value** |
| --- | --- | --- | --- | --- | --- | --- | --- | --- |
| FinnGen | Abdominal subcutaneous AT | Alzheimer's disease | Inverse variance weighted | 0.50 | 1 | 4.80E-01 |  |  |
| FinnGen | Abdominal subcutaneous AT | Aortic aneurysm | Inverse variance weighted | 0.15 | 1 | 7.00E-01 |  |  |
| FinnGen | Abdominal subcutaneous AT | Asthma | Inverse variance weighted | 0.09 | 1 | 7.60E-01 |  |  |
| FinnGen | Abdominal subcutaneous AT | Atrial fibrillation | Inverse variance weighted | 7.63 | 1 | 5.70E-03 |  |  |
| FinnGen | Abdominal subcutaneous AT | Cholelithiasis | Inverse variance weighted | 17.05 | 1 | 3.70E-05 |  |  |
| FinnGen | Abdominal subcutaneous AT | Chronic kidney disease | Inverse variance weighted | 1.28 | 1 | 2.60E-01 |  |  |
| FinnGen | Abdominal subcutaneous AT | Coronary artery disease | Inverse variance weighted | 0.77 | 1 | 3.80E-01 |  |  |
| FinnGen | Abdominal subcutaneous AT | Deep vein thrombosis | Inverse variance weighted | 3.36 | 1 | 6.70E-02 |  |  |
| FinnGen | Abdominal subcutaneous AT | Depression | Inverse variance weighted | 9.27 | 1 | 2.30E-03 |  |  |
| FinnGen | Abdominal subcutaneous AT | Gastroesophageal reflux disease | Inverse variance weighted | 2.24 | 1 | 1.30E-01 |  |  |
| FinnGen | Abdominal subcutaneous AT | Gout | Inverse variance weighted | 0.01 | 1 | 9.40E-01 |  |  |
| FinnGen | Abdominal subcutaneous AT | Heart failure | Inverse variance weighted | 0.81 | 1 | 3.70E-01 |  |  |
| FinnGen | Abdominal subcutaneous AT | Hip osteoarthritis | Inverse variance weighted | 8.46 | 1 | 3.60E-03 |  |  |
| FinnGen | Abdominal subcutaneous AT | Hypertension | Inverse variance weighted | 21.24 | 1 | 4.10E-06 |  |  |
| FinnGen | Abdominal subcutaneous AT | Knee osteoarthritis | Inverse variance weighted | 12.93 | 1 | 3.20E-04 |  |  |
| FinnGen | Abdominal subcutaneous AT | MASLD | Inverse variance weighted | 1.46 | 1 | 2.30E-01 |  |  |
| FinnGen | Abdominal subcutaneous AT | Myocardial infarction | Inverse variance weighted | 4.63 | 1 | 3.10E-02 |  |  |
| FinnGen | Abdominal subcutaneous AT | Osteoporosis | Inverse variance weighted | 1.18 | 1 | 2.80E-01 |  |  |
| FinnGen | Abdominal subcutaneous AT | Parkinson's disease | Inverse variance weighted | 0.06 | 1 | 8.10E-01 |  |  |
| FinnGen | Abdominal subcutaneous AT | Peripheral artery disease | Inverse variance weighted | 1.80 | 1 | 1.80E-01 |  |  |
| FinnGen | Abdominal subcutaneous AT | Polycystic ovary syndrome | Inverse variance weighted | 0.35 | 1 | 5.60E-01 |  |  |
| FinnGen | Abdominal subcutaneous AT | Psoriasis | Inverse variance weighted | 0.31 | 1 | 5.80E-01 |  |  |
| FinnGen | Abdominal subcutaneous AT | Pulmonary embolism | Inverse variance weighted | 0.49 | 1 | 4.80E-01 |  |  |
| FinnGen | Abdominal subcutaneous AT | Rheumatoid arthritis | Inverse variance weighted | 20.30 | 1 | 6.60E-06 |  |  |
| FinnGen | Abdominal subcutaneous AT | Stroke | Inverse variance weighted | 0.13 | 1 | 7.20E-01 |  |  |
| FinnGen | Abdominal subcutaneous AT | Type 2 diabetes | Inverse variance weighted | 88.12 | 1 | 6.20E-21 |  |  |
| FinnGen | Thigh subcutaneous AT | Alzheimer's disease | Inverse variance weighted | 19.43 | 20 | 4.90E-01 |  |  |
| FinnGen | Thigh subcutaneous AT | Alzheimer's disease | MR Egger | 19.41 | 19 | 4.30E-01 | -0.002 | 8.70E-01 |
| FinnGen | Thigh subcutaneous AT | Aortic aneurysm | Inverse variance weighted | 33.88 | 20 | 2.70E-02 |  |  |
| FinnGen | Thigh subcutaneous AT | Aortic aneurysm | MR Egger | 27.67 | 19 | 9.00E-02 | 0.033 | 5.30E-02 |
| FinnGen | Thigh subcutaneous AT | Asthma | Inverse variance weighted | 44.53 | 20 | 1.30E-03 |  |  |
| FinnGen | Thigh subcutaneous AT | Asthma | MR Egger | 43.94 | 19 | 9.60E-04 | 0.005 | 6.20E-01 |
| FinnGen | Thigh subcutaneous AT | Atrial fibrillation | Inverse variance weighted | 107.41 | 20 | 5.80E-14 |  |  |
| FinnGen | Thigh subcutaneous AT | Atrial fibrillation | MR Egger | 106.44 | 19 | 3.60E-14 | 0.008 | 6.80E-01 |
| FinnGen | Thigh subcutaneous AT | Cholelithiasis | Inverse variance weighted | 71.49 | 20 | 1.00E-07 |  |  |
| FinnGen | Thigh subcutaneous AT | Cholelithiasis | MR Egger | 63.82 | 19 | 9.50E-07 | 0.018 | 1.50E-01 |
| FinnGen | Thigh subcutaneous AT | Chronic kidney disease | Inverse variance weighted | 30.32 | 20 | 6.50E-02 |  |  |
| FinnGen | Thigh subcutaneous AT | Chronic kidney disease | MR Egger | 29.07 | 19 | 6.50E-02 | 0.013 | 3.80E-01 |
| FinnGen | Thigh subcutaneous AT | Coronary artery disease | Inverse variance weighted | 44.13 | 20 | 1.50E-03 |  |  |
| FinnGen | Thigh subcutaneous AT | Coronary artery disease | MR Egger | 41.72 | 19 | 1.90E-03 | 0.015 | 3.10E-01 |
| FinnGen | Thigh subcutaneous AT | Deep vein thrombosis | Inverse variance weighted | 43.48 | 20 | 1.80E-03 |  |  |
| FinnGen | Thigh subcutaneous AT | Deep vein thrombosis | MR Egger | 42.11 | 19 | 1.70E-03 | 0.017 | 4.40E-01 |
| FinnGen | Thigh subcutaneous AT | Depression | Inverse variance weighted | 24.51 | 20 | 2.20E-01 |  |  |
| FinnGen | Thigh subcutaneous AT | Depression | MR Egger | 24.45 | 19 | 1.80E-01 | 0.001 | 8.40E-01 |
| FinnGen | Thigh subcutaneous AT | Gastroesophageal reflux disease | Inverse variance weighted | 40.87 | 20 | 3.90E-03 |  |  |
| FinnGen | Thigh subcutaneous AT | Gastroesophageal reflux disease | MR Egger | 40.07 | 19 | 3.20E-03 | 0.006 | 5.50E-01 |
| FinnGen | Thigh subcutaneous AT | Gout | Inverse variance weighted | 43.94 | 20 | 1.50E-03 |  |  |
| FinnGen | Thigh subcutaneous AT | Gout | MR Egger | 40.65 | 19 | 2.70E-03 | 0.030 | 2.30E-01 |
| FinnGen | Thigh subcutaneous AT | Heart failure | Inverse variance weighted | 43.45 | 20 | 1.80E-03 |  |  |
| FinnGen | Thigh subcutaneous AT | Heart failure | MR Egger | 39.56 | 19 | 3.70E-03 | 0.015 | 1.90E-01 |
| FinnGen | Thigh subcutaneous AT | Hip osteoarthritis | Inverse variance weighted | 91.91 | 20 | 3.40E-11 |  |  |
| FinnGen | Thigh subcutaneous AT | Hip osteoarthritis | MR Egger | 71.61 | 19 | 5.00E-08 | 0.037 | 3.20E-02 |
| FinnGen | Thigh subcutaneous AT | Hypertension | Inverse variance weighted | 258.99 | 20 | 1.70E-43 |  |  |
| FinnGen | Thigh subcutaneous AT | Hypertension | MR Egger | 244.59 | 19 | 3.90E-41 | 0.017 | 3.00E-01 |
| FinnGen | Thigh subcutaneous AT | Knee osteoarthritis | Inverse variance weighted | 142.86 | 20 | 1.50E-20 |  |  |
| FinnGen | Thigh subcutaneous AT | Knee osteoarthritis | MR Egger | 123.87 | 19 | 2.10E-17 | 0.027 | 1.00E-01 |
| FinnGen | Thigh subcutaneous AT | MASLD | Inverse variance weighted | 40.79 | 20 | 4.00E-03 |  |  |
| FinnGen | Thigh subcutaneous AT | MASLD | MR Egger | 38.66 | 19 | 4.90E-03 | 0.034 | 3.20E-01 |
| FinnGen | Thigh subcutaneous AT | Myocardial infarction | Inverse variance weighted | 55.95 | 20 | 3.00E-05 |  |  |
| FinnGen | Thigh subcutaneous AT | Myocardial infarction | MR Egger | 53.73 | 19 | 3.60E-05 | 0.012 | 3.90E-01 |
| FinnGen | Thigh subcutaneous AT | Osteoporosis | Inverse variance weighted | 29.05 | 20 | 8.70E-02 |  |  |
| FinnGen | Thigh subcutaneous AT | Osteoporosis | MR Egger | 22.13 | 19 | 2.80E-01 | -0.036 | 2.50E-02 |
| FinnGen | Thigh subcutaneous AT | Parkinson's disease | Inverse variance weighted | 35.00 | 20 | 2.00E-02 |  |  |
| FinnGen | Thigh subcutaneous AT | Parkinson's disease | MR Egger | 34.38 | 19 | 1.70E-02 | -0.014 | 5.70E-01 |
| FinnGen | Thigh subcutaneous AT | Peripheral artery disease | Inverse variance weighted | 38.56 | 20 | 7.60E-03 |  |  |
| FinnGen | Thigh subcutaneous AT | Peripheral artery disease | MR Egger | 36.13 | 19 | 1.00E-02 | 0.018 | 2.70E-01 |
| FinnGen | Thigh subcutaneous AT | Polycystic ovary syndrome | Inverse variance weighted | 20.03 | 20 | 4.60E-01 |  |  |
| FinnGen | Thigh subcutaneous AT | Polycystic ovary syndrome | MR Egger | 19.16 | 19 | 4.50E-01 | 0.027 | 3.60E-01 |
| FinnGen | Thigh subcutaneous AT | Psoriasis | Inverse variance weighted | 26.23 | 20 | 1.60E-01 |  |  |
| FinnGen | Thigh subcutaneous AT | Psoriasis | MR Egger | 26.20 | 19 | 1.30E-01 | -0.002 | 8.90E-01 |
| FinnGen | Thigh subcutaneous AT | Pulmonary embolism | Inverse variance weighted | 37.77 | 20 | 9.50E-03 |  |  |
| FinnGen | Thigh subcutaneous AT | Pulmonary embolism | MR Egger | 37.10 | 19 | 7.70E-03 | 0.010 | 5.70E-01 |
| FinnGen | Thigh subcutaneous AT | Rheumatoid arthritis | Inverse variance weighted | 36.64 | 20 | 1.30E-02 |  |  |
| FinnGen | Thigh subcutaneous AT | Rheumatoid arthritis | MR Egger | 36.41 | 19 | 9.40E-03 | 0.005 | 7.30E-01 |
| FinnGen | Thigh subcutaneous AT | Stroke | Inverse variance weighted | 32.24 | 20 | 4.10E-02 |  |  |
| FinnGen | Thigh subcutaneous AT | Stroke | MR Egger | 31.71 | 19 | 3.40E-02 | -0.008 | 5.80E-01 |
| FinnGen | Thigh subcutaneous AT | Type 2 diabetes | Inverse variance weighted | 620.07 | 20 | 1.70E-118 |  |  |
| FinnGen | Thigh subcutaneous AT | Type 2 diabetes | MR Egger | 607.12 | 19 | 1.60E-116 |  |  |
| FinnGen | Thigh intermuscular AT | Alzheimer's disease | Inverse variance weighted | 20.68 | 12 | 5.50E-02 |  |  |
| FinnGen | Thigh intermuscular AT | Alzheimer's disease | MR Egger | 19.99 | 11 | 4.60E-02 | 0.019 | 5.50E-01 |
| FinnGen | Thigh intermuscular AT | Aortic aneurysm | Inverse variance weighted | 13.81 | 12 | 3.10E-01 |  |  |
| FinnGen | Thigh intermuscular AT | Aortic aneurysm | MR Egger | 12.48 | 11 | 3.30E-01 | -0.028 | 3.00E-01 |
| FinnGen | Thigh intermuscular AT | Asthma | Inverse variance weighted | 54.67 | 12 | 2.10E-07 |  |  |
| FinnGen | Thigh intermuscular AT | Asthma | MR Egger | 50.91 | 11 | 4.30E-07 | -0.023 | 3.90E-01 |
| FinnGen | Thigh intermuscular AT | Atrial fibrillation | Inverse variance weighted | 23.49 | 12 | 2.40E-02 |  |  |
| FinnGen | Thigh intermuscular AT | Atrial fibrillation | MR Egger | 19.86 | 11 | 4.70E-02 | -0.027 | 1.80E-01 |
| FinnGen | Thigh intermuscular AT | Cholelithiasis | Inverse variance weighted | 35.95 | 12 | 3.30E-04 |  |  |
| FinnGen | Thigh intermuscular AT | Cholelithiasis | MR Egger | 34.51 | 11 | 3.00E-04 | 0.014 | 5.10E-01 |
| FinnGen | Thigh intermuscular AT | Chronic kidney disease | Inverse variance weighted | 20.67 | 12 | 5.50E-02 |  |  |
| FinnGen | Thigh intermuscular AT | Chronic kidney disease | MR Egger | 20.07 | 11 | 4.50E-02 | -0.017 | 5.80E-01 |
| FinnGen | Thigh intermuscular AT | Coronary artery disease | Inverse variance weighted | 12.58 | 12 | 4.00E-01 |  |  |
| FinnGen | Thigh intermuscular AT | Coronary artery disease | MR Egger | 11.83 | 11 | 3.80E-01 | 0.016 | 4.20E-01 |
| FinnGen | Thigh intermuscular AT | Deep vein thrombosis | Inverse variance weighted | 9.61 | 12 | 6.50E-01 |  |  |
| FinnGen | Thigh intermuscular AT | Deep vein thrombosis | MR Egger | 9.61 | 11 | 5.70E-01 | 0.001 | 9.80E-01 |
| FinnGen | Thigh intermuscular AT | Depression | Inverse variance weighted | 8.82 | 12 | 7.20E-01 |  |  |
| FinnGen | Thigh intermuscular AT | Depression | MR Egger | 8.81 | 11 | 6.40E-01 | -0.001 | 9.20E-01 |
| FinnGen | Thigh intermuscular AT | Gastroesophageal reflux disease | Inverse variance weighted | 13.01 | 12 | 3.70E-01 |  |  |
| FinnGen | Thigh intermuscular AT | Gastroesophageal reflux disease | MR Egger | 11.18 | 11 | 4.30E-01 | -0.018 | 2.10E-01 |
| FinnGen | Thigh intermuscular AT | Gout | Inverse variance weighted | 29.85 | 12 | 3.00E-03 |  |  |
| FinnGen | Thigh intermuscular AT | Gout | MR Egger | 22.87 | 11 | 1.80E-02 | 0.081 | 9.40E-02 |
| FinnGen | Thigh intermuscular AT | Heart failure | Inverse variance weighted | 14.24 | 12 | 2.90E-01 |  |  |
| FinnGen | Thigh intermuscular AT | Heart failure | MR Egger | 12.11 | 11 | 3.60E-01 | -0.020 | 1.90E-01 |
| FinnGen | Thigh intermuscular AT | Hip osteoarthritis | Inverse variance weighted | 41.22 | 12 | 4.50E-05 |  |  |
| FinnGen | Thigh intermuscular AT | Hip osteoarthritis | MR Egger | 38.79 | 11 | 5.80E-05 | -0.024 | 4.20E-01 |
| FinnGen | Thigh intermuscular AT | Hypertension | Inverse variance weighted | 34.99 | 12 | 4.70E-04 |  |  |
| FinnGen | Thigh intermuscular AT | Hypertension | MR Egger | 34.37 | 11 | 3.10E-04 | -0.007 | 6.70E-01 |
| FinnGen | Thigh intermuscular AT | Knee osteoarthritis | Inverse variance weighted | 42.59 | 12 | 2.70E-05 |  |  |
| FinnGen | Thigh intermuscular AT | Knee osteoarthritis | MR Egger | 42.40 | 11 | 1.40E-05 | 0.005 | 8.30E-01 |
| FinnGen | Thigh intermuscular AT | MASLD | Inverse variance weighted | 13.99 | 12 | 3.00E-01 |  |  |
| FinnGen | Thigh intermuscular AT | MASLD | MR Egger | 13.96 | 11 | 2.40E-01 | -0.007 | 9.00E-01 |
| FinnGen | Thigh intermuscular AT | Myocardial infarction | Inverse variance weighted | 16.27 | 12 | 1.80E-01 |  |  |
| FinnGen | Thigh intermuscular AT | Myocardial infarction | MR Egger | 12.53 | 11 | 3.30E-01 | -0.030 | 9.70E-02 |
| FinnGen | Thigh intermuscular AT | Osteoporosis | Inverse variance weighted | 15.44 | 12 | 2.20E-01 |  |  |
| FinnGen | Thigh intermuscular AT | Osteoporosis | MR Egger | 14.49 | 11 | 2.10E-01 | 0.024 | 4.10E-01 |
| FinnGen | Thigh intermuscular AT | Parkinson's disease | Inverse variance weighted | 13.65 | 12 | 3.20E-01 |  |  |
| FinnGen | Thigh intermuscular AT | Parkinson's disease | MR Egger | 13.53 | 11 | 2.60E-01 | -0.011 | 7.60E-01 |
| FinnGen | Thigh intermuscular AT | Peripheral artery disease | Inverse variance weighted | 22.42 | 13 | 4.90E-02 |  |  |
| FinnGen | Thigh intermuscular AT | Peripheral artery disease | MR Egger | 22.33 | 12 | 3.40E-02 | 0.006 | 8.30E-01 |
| FinnGen | Thigh intermuscular AT | Polycystic ovary syndrome | Inverse variance weighted | 12.66 | 12 | 3.90E-01 |  |  |
| FinnGen | Thigh intermuscular AT | Polycystic ovary syndrome | MR Egger | 12.66 | 11 | 3.20E-01 | 0.002 | 9.80E-01 |
| FinnGen | Thigh intermuscular AT | Psoriasis | Inverse variance weighted | 11.65 | 12 | 4.70E-01 |  |  |
| FinnGen | Thigh intermuscular AT | Psoriasis | MR Egger | 11.54 | 11 | 4.00E-01 | 0.007 | 7.50E-01 |
| FinnGen | Thigh intermuscular AT | Pulmonary embolism | Inverse variance weighted | 19.40 | 12 | 7.90E-02 |  |  |
| FinnGen | Thigh intermuscular AT | Pulmonary embolism | MR Egger | 16.84 | 11 | 1.10E-01 | -0.035 | 2.20E-01 |
| FinnGen | Thigh intermuscular AT | Rheumatoid arthritis | Inverse variance weighted | 19.29 | 12 | 8.20E-02 |  |  |
| FinnGen | Thigh intermuscular AT | Rheumatoid arthritis | MR Egger | 16.38 | 11 | 1.30E-01 | 0.033 | 1.90E-01 |
| FinnGen | Thigh intermuscular AT | Stroke | Inverse variance weighted | 29.96 | 13 | 4.80E-03 |  |  |
| FinnGen | Thigh intermuscular AT | Stroke | MR Egger | 27.02 | 12 | 7.70E-03 | -0.035 | 2.80E-01 |
| FinnGen | Thigh intermuscular AT | Type 2 diabetes | Inverse variance weighted | 129.60 | 12 | 7.40E-22 |  |  |
| FinnGen | Thigh intermuscular AT | Type 2 diabetes | MR Egger | 115.54 | 11 | 1.40E-19 | 0.037 | 2.70E-01 |
| FinnGen | Visceral AT | Alzheimer's disease | Inverse variance weighted | 6.72 | 5 | 2.40E-01 |  |  |
| FinnGen | Visceral AT | Alzheimer's disease | MR Egger | 6.05 | 4 | 2.00E-01 | 0.043 | 5.40E-01 |
| FinnGen | Visceral AT | Aortic aneurysm | Inverse variance weighted | 7.09 | 5 | 2.10E-01 |  |  |
| FinnGen | Visceral AT | Aortic aneurysm | MR Egger | 5.21 | 4 | 2.70E-01 | 0.077 | 3.00E-01 |
| FinnGen | Visceral AT | Asthma | Inverse variance weighted | 3.63 | 5 | 6.00E-01 |  |  |
| FinnGen | Visceral AT | Asthma | MR Egger | 2.69 | 4 | 6.10E-01 | 0.027 | 3.90E-01 |
| FinnGen | Visceral AT | Atrial fibrillation | Inverse variance weighted | 43.50 | 5 | 2.90E-08 |  |  |
| FinnGen | Visceral AT | Atrial fibrillation | MR Egger | 23.14 | 4 | 1.20E-04 | 0.147 | 1.30E-01 |
| FinnGen | Visceral AT | Cholelithiasis | Inverse variance weighted | 20.60 | 5 | 9.60E-04 |  |  |
| FinnGen | Visceral AT | Cholelithiasis | MR Egger | 20.60 | 4 | 3.80E-04 | 0.002 | 9.70E-01 |
| FinnGen | Visceral AT | Chronic kidney disease | Inverse variance weighted | 9.28 | 5 | 9.80E-02 |  |  |
| FinnGen | Visceral AT | Chronic kidney disease | MR Egger | 8.91 | 4 | 6.40E-02 | -0.031 | 7.00E-01 |
| FinnGen | Visceral AT | Coronary artery disease | Inverse variance weighted | 14.95 | 5 | 1.10E-02 |  |  |
| FinnGen | Visceral AT | Coronary artery disease | MR Egger | 0.58 | 4 | 9.70E-01 | 0.154 | 1.90E-02 |
| FinnGen | Visceral AT | Deep vein thrombosis | Inverse variance weighted | 11.95 | 5 | 3.50E-02 |  |  |
| FinnGen | Visceral AT | Deep vein thrombosis | MR Egger | 8.30 | 4 | 8.10E-02 | -0.117 | 2.60E-01 |
| FinnGen | Visceral AT | Depression | Inverse variance weighted | 17.76 | 5 | 3.30E-03 |  |  |
| FinnGen | Visceral AT | Depression | MR Egger | 16.45 | 4 | 2.50E-03 | 0.028 | 6.00E-01 |
| FinnGen | Visceral AT | Gastroesophageal reflux disease | Inverse variance weighted | 15.24 | 5 | 9.40E-03 |  |  |
| FinnGen | Visceral AT | Gastroesophageal reflux disease | MR Egger | 5.94 | 4 | 2.00E-01 | 0.092 | 6.70E-02 |
| FinnGen | Visceral AT | Gout | Inverse variance weighted | 7.78 | 5 | 1.70E-01 |  |  |
| FinnGen | Visceral AT | Gout | MR Egger | 6.89 | 4 | 1.40E-01 | 0.065 | 5.10E-01 |
| FinnGen | Visceral AT | Heart failure | Inverse variance weighted | 11.13 | 5 | 4.90E-02 |  |  |
| FinnGen | Visceral AT | Heart failure | MR Egger | 8.28 | 4 | 8.20E-02 | 0.053 | 3.10E-01 |
| FinnGen | Visceral AT | Hip osteoarthritis | Inverse variance weighted | 42.82 | 5 | 4.00E-08 |  |  |
| FinnGen | Visceral AT | Hip osteoarthritis | MR Egger | 19.87 | 4 | 5.30E-04 | 0.165 | 9.80E-02 |
| FinnGen | Visceral AT | Hypertension | Inverse variance weighted | 139.35 | 5 | 2.50E-28 |  |  |
| FinnGen | Visceral AT | Hypertension | MR Egger | 62.03 | 4 | 1.10E-12 | 0.168 | 8.90E-02 |
| FinnGen | Visceral AT | Knee osteoarthritis | Inverse variance weighted | 95.44 | 5 | 4.80E-19 |  |  |
| FinnGen | Visceral AT | Knee osteoarthritis | MR Egger | 43.03 | 4 | 1.00E-08 | 0.186 | 9.20E-02 |
| FinnGen | Visceral AT | MASLD | Inverse variance weighted | 18.16 | 5 | 2.80E-03 |  |  |
| FinnGen | Visceral AT | MASLD | MR Egger | 14.52 | 4 | 5.80E-03 | 0.185 | 3.70E-01 |
| FinnGen | Visceral AT | Myocardial infarction | Inverse variance weighted | 12.90 | 5 | 2.40E-02 |  |  |
| FinnGen | Visceral AT | Myocardial infarction | MR Egger | 7.07 | 4 | 1.30E-01 | 0.085 | 1.40E-01 |
| FinnGen | Visceral AT | Osteoporosis | Inverse variance weighted | 7.51 | 5 | 1.90E-01 |  |  |
| FinnGen | Visceral AT | Osteoporosis | MR Egger | 7.49 | 4 | 1.10E-01 | 0.009 | 9.10E-01 |
| FinnGen | Visceral AT | Parkinson's disease | Inverse variance weighted | 1.63 | 5 | 9.00E-01 |  |  |
| FinnGen | Visceral AT | Parkinson's disease | MR Egger | 1.62 | 4 | 8.00E-01 | 0.005 | 9.50E-01 |
| FinnGen | Visceral AT | Peripheral artery disease | Inverse variance weighted | 13.35 | 5 | 2.00E-02 |  |  |
| FinnGen | Visceral AT | Peripheral artery disease | MR Egger | 2.35 | 4 | 6.70E-01 | 0.156 | 3.00E-02 |
| FinnGen | Visceral AT | Polycystic ovary syndrome | Inverse variance weighted | 10.97 | 5 | 5.20E-02 |  |  |
| FinnGen | Visceral AT | Polycystic ovary syndrome | MR Egger | 10.54 | 4 | 3.20E-02 | 0.080 | 7.10E-01 |
| FinnGen | Visceral AT | Psoriasis | Inverse variance weighted | 3.99 | 5 | 5.50E-01 |  |  |
| FinnGen | Visceral AT | Psoriasis | MR Egger | 3.20 | 4 | 5.30E-01 | -0.043 | 4.30E-01 |
| FinnGen | Visceral AT | Pulmonary embolism | Inverse variance weighted | 12.64 | 5 | 2.70E-02 |  |  |
| FinnGen | Visceral AT | Pulmonary embolism | MR Egger | 12.47 | 4 | 1.40E-02 | 0.020 | 8.30E-01 |
| FinnGen | Visceral AT | Rheumatoid arthritis | Inverse variance weighted | 23.84 | 5 | 2.30E-04 |  |  |
| FinnGen | Visceral AT | Rheumatoid arthritis | MR Egger | 21.74 | 4 | 2.30E-04 | 0.064 | 5.70E-01 |
| FinnGen | Visceral AT | Stroke | Inverse variance weighted | 29.34 | 5 | 2.00E-05 |  |  |
| FinnGen | Visceral AT | Stroke | MR Egger | 16.24 | 4 | 2.70E-03 | 0.170 | 1.50E-01 |
| FinnGen | Visceral AT | Type 2 diabetes | Inverse variance weighted | 318.29 | 5 | 1.20E-66 |  |  |
| FinnGen | Visceral AT | Type 2 diabetes | MR Egger | 201.62 | 4 | 1.70E-42 | 0.244 | 2.00E-01 |
| FinnGen | Liver PDFF | Alzheimer's disease | Inverse variance weighted | 3056.41 | 8 | 0.00E+00 |  |  |
| FinnGen | Liver PDFF | Alzheimer's disease | MR Egger | 2917.36 | 7 | 0.00E+00 | -0.136 | 5.80E-01 |
| FinnGen | Liver PDFF | Aortic aneurysm | Inverse variance weighted | 25.83 | 8 | 1.10E-03 |  |  |
| FinnGen | Liver PDFF | Aortic aneurysm | MR Egger | 21.34 | 7 | 3.30E-03 | 0.027 | 2.60E-01 |
| FinnGen | Liver PDFF | Asthma | Inverse variance weighted | 47.55 | 8 | 1.20E-07 |  |  |
| FinnGen | Liver PDFF | Asthma | MR Egger | 36.42 | 7 | 6.10E-06 | 0.021 | 1.90E-01 |
| FinnGen | Liver PDFF | Atrial fibrillation | Inverse variance weighted | 11.01 | 8 | 2.00E-01 |  |  |
| FinnGen | Liver PDFF | Atrial fibrillation | MR Egger | 10.14 | 7 | 1.80E-01 | 0.007 | 4.70E-01 |
| FinnGen | Liver PDFF | Cholelithiasis | Inverse variance weighted | 153.72 | 8 | 3.30E-29 |  |  |
| FinnGen | Liver PDFF | Cholelithiasis | MR Egger | 124.77 | 7 | 7.80E-24 | -0.032 | 2.40E-01 |
| FinnGen | Liver PDFF | Chronic kidney disease | Inverse variance weighted | 27.45 | 8 | 5.90E-04 |  |  |
| FinnGen | Liver PDFF | Chronic kidney disease | MR Egger | 24.35 | 7 | 9.90E-04 | 0.020 | 3.80E-01 |
| FinnGen | Liver PDFF | Coronary artery disease | Inverse variance weighted | 49.26 | 8 | 5.70E-08 |  |  |
| FinnGen | Liver PDFF | Coronary artery disease | MR Egger | 15.19 | 7 | 3.40E-02 | 0.053 | 5.40E-03 |
| FinnGen | Liver PDFF | Deep vein thrombosis | Inverse variance weighted | 7.17 | 8 | 5.20E-01 |  |  |
| FinnGen | Liver PDFF | Deep vein thrombosis | MR Egger | 6.44 | 7 | 4.90E-01 | 0.012 | 4.20E-01 |
| FinnGen | Liver PDFF | Depression | Inverse variance weighted | 16.22 | 8 | 3.90E-02 |  |  |
| FinnGen | Liver PDFF | Depression | MR Egger | 13.92 | 7 | 5.30E-02 | 0.008 | 3.20E-01 |
| FinnGen | Liver PDFF | Gastroesophageal reflux disease | Inverse variance weighted | 11.81 | 8 | 1.60E-01 |  |  |
| FinnGen | Liver PDFF | Gastroesophageal reflux disease | MR Egger | 9.94 | 7 | 1.90E-01 | 0.009 | 2.90E-01 |
| FinnGen | Liver PDFF | Gout | Inverse variance weighted | 93.89 | 8 | 7.50E-17 |  |  |
| FinnGen | Liver PDFF | Gout | MR Egger | 60.90 | 7 | 1.00E-10 | 0.089 | 9.30E-02 |
| FinnGen | Liver PDFF | Heart failure | Inverse variance weighted | 28.08 | 8 | 4.60E-04 |  |  |
| FinnGen | Liver PDFF | Heart failure | MR Egger | 26.85 | 7 | 3.60E-04 | 0.008 | 5.90E-01 |
| FinnGen | Liver PDFF | Hip osteoarthritis | Inverse variance weighted | 24.77 | 8 | 1.70E-03 |  |  |
| FinnGen | Liver PDFF | Hip osteoarthritis | MR Egger | 19.49 | 7 | 6.80E-03 | 0.018 | 2.10E-01 |
| FinnGen | Liver PDFF | Hypertension | Inverse variance weighted | 62.98 | 8 | 1.20E-10 |  |  |
| FinnGen | Liver PDFF | Hypertension | MR Egger | 62.43 | 7 | 4.90E-11 | 0.003 | 8.10E-01 |
| FinnGen | Liver PDFF | Knee osteoarthritis | Inverse variance weighted | 42.21 | 8 | 1.20E-06 |  |  |
| FinnGen | Liver PDFF | Knee osteoarthritis | MR Egger | 34.34 | 7 | 1.50E-05 | 0.016 | 2.50E-01 |
| FinnGen | Liver PDFF | MASLD | Inverse variance weighted | 22.05 | 8 | 4.80E-03 |  |  |
| FinnGen | Liver PDFF | MASLD | MR Egger | 20.03 | 7 | 5.50E-03 | 0.030 | 4.30E-01 |
| FinnGen | Liver PDFF | Myocardial infarction | Inverse variance weighted | 48.26 | 8 | 8.80E-08 |  |  |
| FinnGen | Liver PDFF | Myocardial infarction | MR Egger | 27.81 | 7 | 2.40E-04 | 0.036 | 5.80E-02 |
| FinnGen | Liver PDFF | Osteoporosis | Inverse variance weighted | 21.83 | 8 | 5.30E-03 |  |  |
| FinnGen | Liver PDFF | Osteoporosis | MR Egger | 21.69 | 7 | 2.90E-03 | -0.005 | 8.40E-01 |
| FinnGen | Liver PDFF | Parkinson's disease | Inverse variance weighted | 8.01 | 8 | 4.30E-01 |  |  |
| FinnGen | Liver PDFF | Parkinson's disease | MR Egger | 5.56 | 7 | 5.90E-01 | -0.025 | 1.60E-01 |
| FinnGen | Liver PDFF | Peripheral artery disease | Inverse variance weighted | 34.03 | 8 | 4.00E-05 |  |  |
| FinnGen | Liver PDFF | Peripheral artery disease | MR Egger | 9.62 | 7 | 2.10E-01 | 0.052 | 4.00E-03 |
| FinnGen | Liver PDFF | Polycystic ovary syndrome | Inverse variance weighted | 14.58 | 8 | 6.80E-02 |  |  |
| FinnGen | Liver PDFF | Polycystic ovary syndrome | MR Egger | 14.49 | 7 | 4.30E-02 | 0.008 | 8.40E-01 |
| FinnGen | Liver PDFF | Psoriasis | Inverse variance weighted | 22.49 | 8 | 4.10E-03 |  |  |
| FinnGen | Liver PDFF | Psoriasis | MR Egger | 22.47 | 7 | 2.10E-03 | 0.002 | 9.40E-01 |
| FinnGen | Liver PDFF | Pulmonary embolism | Inverse variance weighted | 28.99 | 8 | 3.20E-04 |  |  |
| FinnGen | Liver PDFF | Pulmonary embolism | MR Egger | 27.50 | 7 | 2.70E-04 | 0.014 | 5.60E-01 |
| FinnGen | Liver PDFF | Rheumatoid arthritis | Inverse variance weighted | 10.62 | 8 | 2.20E-01 |  |  |
| FinnGen | Liver PDFF | Rheumatoid arthritis | MR Egger | 6.33 | 7 | 5.00E-01 | 0.020 | 7.70E-02 |
| FinnGen | Liver PDFF | Stroke | Inverse variance weighted | 8.47 | 8 | 3.90E-01 |  |  |
| FinnGen | Liver PDFF | Stroke | MR Egger | 8.28 | 7 | 3.10E-01 | 0.005 | 7.00E-01 |
| FinnGen | Liver PDFF | Type 2 diabetes | Inverse variance weighted | 179.35 | 8 | 1.40E-34 |  |  |
| FinnGen | Liver PDFF | Type 2 diabetes | MR Egger | 147.99 | 7 | 1.10E-28 | -0.028 | 2.60E-01 |
| FinnGen | Pancreas PDFF | Alzheimer's disease | Inverse variance weighted | 17.54 | 12 | 1.30E-01 |  |  |
| FinnGen | Pancreas PDFF | Alzheimer's disease | MR Egger | 16.00 | 11 | 1.40E-01 | 0.021 | 3.30E-01 |
| FinnGen | Pancreas PDFF | Aortic aneurysm | Inverse variance weighted | 11.54 | 12 | 4.80E-01 |  |  |
| FinnGen | Pancreas PDFF | Aortic aneurysm | MR Egger | 11.32 | 11 | 4.20E-01 | 0.009 | 6.50E-01 |
| FinnGen | Pancreas PDFF | Asthma | Inverse variance weighted | 37.29 | 12 | 2.00E-04 |  |  |
| FinnGen | Pancreas PDFF | Asthma | MR Egger | 30.41 | 11 | 1.40E-03 | -0.024 | 1.40E-01 |
| FinnGen | Pancreas PDFF | Atrial fibrillation | Inverse variance weighted | 39.93 | 12 | 7.40E-05 |  |  |
| FinnGen | Pancreas PDFF | Atrial fibrillation | MR Egger | 38.85 | 11 | 5.60E-05 | -0.011 | 5.90E-01 |
| FinnGen | Pancreas PDFF | Cholelithiasis | Inverse variance weighted | 116.08 | 12 | 3.70E-19 |  |  |
| FinnGen | Pancreas PDFF | Cholelithiasis | MR Egger | 115.43 | 11 | 1.50E-19 | 0.007 | 8.10E-01 |
| FinnGen | Pancreas PDFF | Chronic kidney disease | Inverse variance weighted | 7.60 | 12 | 8.20E-01 |  |  |
| FinnGen | Pancreas PDFF | Chronic kidney disease | MR Egger | 7.60 | 11 | 7.50E-01 | -0.001 | 9.60E-01 |
| FinnGen | Pancreas PDFF | Coronary artery disease | Inverse variance weighted | 34.82 | 12 | 5.00E-04 |  |  |
| FinnGen | Pancreas PDFF | Coronary artery disease | MR Egger | 34.16 | 11 | 3.40E-04 | -0.011 | 6.60E-01 |
| FinnGen | Pancreas PDFF | Deep vein thrombosis | Inverse variance weighted | 221.11 | 12 | 1.40E-40 |  |  |
| FinnGen | Pancreas PDFF | Deep vein thrombosis | MR Egger | 220.00 | 11 | 5.20E-41 | -0.021 | 8.20E-01 |
| FinnGen | Pancreas PDFF | Depression | Inverse variance weighted | 21.87 | 12 | 3.90E-02 |  |  |
| FinnGen | Pancreas PDFF | Depression | MR Egger | 21.47 | 11 | 2.90E-02 | 0.005 | 6.60E-01 |
| FinnGen | Pancreas PDFF | Gastroesophageal reflux disease | Inverse variance weighted | 18.25 | 12 | 1.10E-01 |  |  |
| FinnGen | Pancreas PDFF | Gastroesophageal reflux disease | MR Egger | 16.79 | 11 | 1.10E-01 | -0.012 | 3.50E-01 |
| FinnGen | Pancreas PDFF | Gout | Inverse variance weighted | 19.86 | 12 | 7.00E-02 |  |  |
| FinnGen | Pancreas PDFF | Gout | MR Egger | 16.62 | 11 | 1.20E-01 | -0.040 | 1.70E-01 |
| FinnGen | Pancreas PDFF | Heart failure | Inverse variance weighted | 33.56 | 12 | 7.90E-04 |  |  |
| FinnGen | Pancreas PDFF | Heart failure | MR Egger | 32.57 | 11 | 6.20E-04 | -0.010 | 5.80E-01 |
| FinnGen | Pancreas PDFF | Hip osteoarthritis | Inverse variance weighted | 27.33 | 12 | 6.90E-03 |  |  |
| FinnGen | Pancreas PDFF | Hip osteoarthritis | MR Egger | 27.28 | 11 | 4.20E-03 | 0.002 | 8.90E-01 |
| FinnGen | Pancreas PDFF | Hypertension | Inverse variance weighted | 41.94 | 12 | 3.40E-05 |  |  |
| FinnGen | Pancreas PDFF | Hypertension | MR Egger | 41.15 | 11 | 2.30E-05 | 0.006 | 6.50E-01 |
| FinnGen | Pancreas PDFF | Knee osteoarthritis | Inverse variance weighted | 26.06 | 12 | 1.10E-02 |  |  |
| FinnGen | Pancreas PDFF | Knee osteoarthritis | MR Egger | 25.89 | 11 | 6.80E-03 | -0.004 | 7.90E-01 |
| FinnGen | Pancreas PDFF | MASLD | Inverse variance weighted | 16.54 | 12 | 1.70E-01 |  |  |
| FinnGen | Pancreas PDFF | MASLD | MR Egger | 15.40 | 11 | 1.70E-01 | 0.034 | 3.90E-01 |
| FinnGen | Pancreas PDFF | Myocardial infarction | Inverse variance weighted | 44.09 | 12 | 1.50E-05 |  |  |
| FinnGen | Pancreas PDFF | Myocardial infarction | MR Egger | 43.30 | 11 | 9.60E-06 | -0.010 | 6.60E-01 |
| FinnGen | Pancreas PDFF | Osteoporosis | Inverse variance weighted | 14.22 | 12 | 2.90E-01 |  |  |
| FinnGen | Pancreas PDFF | Osteoporosis | MR Egger | 13.41 | 11 | 2.70E-01 | -0.016 | 4.30E-01 |
| FinnGen | Pancreas PDFF | Parkinson's disease | Inverse variance weighted | 22.78 | 12 | 3.00E-02 |  |  |
| FinnGen | Pancreas PDFF | Parkinson's disease | MR Egger | 22.59 | 11 | 2.00E-02 | -0.010 | 7.70E-01 |
| FinnGen | Pancreas PDFF | Peripheral artery disease | Inverse variance weighted | 20.87 | 11 | 3.50E-02 |  |  |
| FinnGen | Pancreas PDFF | Peripheral artery disease | MR Egger | 20.67 | 10 | 2.40E-02 | 0.007 | 7.60E-01 |
| FinnGen | Pancreas PDFF | Polycystic ovary syndrome | Inverse variance weighted | 14.75 | 12 | 2.60E-01 |  |  |
| FinnGen | Pancreas PDFF | Polycystic ovary syndrome | MR Egger | 13.53 | 11 | 2.60E-01 | 0.044 | 3.40E-01 |
| FinnGen | Pancreas PDFF | Psoriasis | Inverse variance weighted | 29.92 | 12 | 2.90E-03 |  |  |
| FinnGen | Pancreas PDFF | Psoriasis | MR Egger | 29.47 | 11 | 1.90E-03 | -0.011 | 6.90E-01 |
| FinnGen | Pancreas PDFF | Pulmonary embolism | Inverse variance weighted | 168.71 | 12 | 8.80E-30 |  |  |
| FinnGen | Pancreas PDFF | Pulmonary embolism | MR Egger | 162.25 | 11 | 4.60E-29 | -0.041 | 5.20E-01 |
| FinnGen | Pancreas PDFF | Rheumatoid arthritis | Inverse variance weighted | 52.57 | 12 | 4.90E-07 |  |  |
| FinnGen | Pancreas PDFF | Rheumatoid arthritis | MR Egger | 50.40 | 11 | 5.30E-07 | 0.021 | 5.10E-01 |
| FinnGen | Pancreas PDFF | Stroke | Inverse variance weighted | 23.00 | 11 | 1.80E-02 |  |  |
| FinnGen | Pancreas PDFF | Stroke | MR Egger | 22.83 | 10 | 1.10E-02 | -0.006 | 7.90E-01 |
| FinnGen | Pancreas PDFF | Type 2 diabetes | Inverse variance weighted | 178.58 | 12 | 8.40E-32 |  |  |
| FinnGen | Pancreas PDFF | Type 2 diabetes | MR Egger | 172.49 | 11 | 3.60E-31 | 0.018 | 5.50E-01 |
| FinnGen | Paraspinal AT | Alzheimer's disease | Inverse variance weighted | 9.32 | 7 | 2.30E-01 |  |  |
| FinnGen | Paraspinal AT | Alzheimer's disease | MR Egger | 8.27 | 6 | 2.20E-01 | -0.024 | 4.20E-01 |
| FinnGen | Paraspinal AT | Aortic aneurysm | Inverse variance weighted | 8.21 | 7 | 3.10E-01 |  |  |
| FinnGen | Paraspinal AT | Aortic aneurysm | MR Egger | 5.74 | 6 | 4.50E-01 | 0.040 | 1.70E-01 |
| FinnGen | Paraspinal AT | Asthma | Inverse variance weighted | 26.49 | 7 | 4.10E-04 |  |  |
| FinnGen | Paraspinal AT | Asthma | MR Egger | 24.81 | 6 | 3.70E-04 | -0.016 | 5.50E-01 |
| FinnGen | Paraspinal AT | Atrial fibrillation | Inverse variance weighted | 30.18 | 7 | 8.80E-05 |  |  |
| FinnGen | Paraspinal AT | Atrial fibrillation | MR Egger | 24.39 | 6 | 4.40E-04 | 0.035 | 2.80E-01 |
| FinnGen | Paraspinal AT | Cholelithiasis | Inverse variance weighted | 46.26 | 7 | 7.80E-08 |  |  |
| FinnGen | Paraspinal AT | Cholelithiasis | MR Egger | 27.26 | 6 | 1.30E-04 | 0.052 | 8.70E-02 |
| FinnGen | Paraspinal AT | Chronic kidney disease | Inverse variance weighted | 15.55 | 7 | 3.00E-02 |  |  |
| FinnGen | Paraspinal AT | Chronic kidney disease | MR Egger | 12.22 | 6 | 5.70E-02 | 0.041 | 2.50E-01 |
| FinnGen | Paraspinal AT | Coronary artery disease | Inverse variance weighted | 15.97 | 7 | 2.50E-02 |  |  |
| FinnGen | Paraspinal AT | Coronary artery disease | MR Egger | 10.52 | 6 | 1.00E-01 | 0.043 | 1.30E-01 |
| FinnGen | Paraspinal AT | Deep vein thrombosis | Inverse variance weighted | 9.69 | 7 | 2.10E-01 |  |  |
| FinnGen | Paraspinal AT | Deep vein thrombosis | MR Egger | 9.58 | 6 | 1.40E-01 | 0.009 | 8.10E-01 |
| FinnGen | Paraspinal AT | Depression | Inverse variance weighted | 9.54 | 7 | 2.20E-01 |  |  |
| FinnGen | Paraspinal AT | Depression | MR Egger | 8.93 | 6 | 1.80E-01 | 0.009 | 5.50E-01 |
| FinnGen | Paraspinal AT | Gastroesophageal reflux disease | Inverse variance weighted | 12.72 | 7 | 7.90E-02 |  |  |
| FinnGen | Paraspinal AT | Gastroesophageal reflux disease | MR Egger | 12.59 | 6 | 5.00E-02 | 0.005 | 8.10E-01 |
| FinnGen | Paraspinal AT | Gout | Inverse variance weighted | 14.01 | 7 | 5.10E-02 |  |  |
| FinnGen | Paraspinal AT | Gout | MR Egger | 14.01 | 6 | 3.00E-02 | 0.000 | 1.00E+00 |
| FinnGen | Paraspinal AT | Heart failure | Inverse variance weighted | 18.50 | 7 | 9.90E-03 |  |  |
| FinnGen | Paraspinal AT | Heart failure | MR Egger | 18.23 | 6 | 5.70E-03 | -0.007 | 7.80E-01 |
| FinnGen | Paraspinal AT | Hip osteoarthritis | Inverse variance weighted | 30.33 | 7 | 8.20E-05 |  |  |
| FinnGen | Paraspinal AT | Hip osteoarthritis | MR Egger | 26.19 | 6 | 2.10E-04 | 0.032 | 3.70E-01 |
| FinnGen | Paraspinal AT | Hypertension | Inverse variance weighted | 102.61 | 7 | 3.10E-19 |  |  |
| FinnGen | Paraspinal AT | Hypertension | MR Egger | 77.63 | 6 | 1.10E-14 | 0.043 | 2.10E-01 |
| FinnGen | Paraspinal AT | Knee osteoarthritis | Inverse variance weighted | 73.76 | 7 | 2.60E-13 |  |  |
| FinnGen | Paraspinal AT | Knee osteoarthritis | MR Egger | 52.11 | 6 | 1.80E-09 | 0.054 | 1.70E-01 |
| FinnGen | Paraspinal AT | MASLD | Inverse variance weighted | 27.05 | 7 | 3.30E-04 |  |  |
| FinnGen | Paraspinal AT | MASLD | MR Egger | 22.74 | 6 | 8.90E-04 | 0.091 | 3.30E-01 |
| FinnGen | Paraspinal AT | Myocardial infarction | Inverse variance weighted | 6.91 | 7 | 4.40E-01 |  |  |
| FinnGen | Paraspinal AT | Myocardial infarction | MR Egger | 6.90 | 6 | 3.30E-01 | 0.001 | 9.50E-01 |
| FinnGen | Paraspinal AT | Osteoporosis | Inverse variance weighted | 13.02 | 7 | 7.20E-02 |  |  |
| FinnGen | Paraspinal AT | Osteoporosis | MR Egger | 12.51 | 6 | 5.20E-02 | -0.018 | 6.40E-01 |
| FinnGen | Paraspinal AT | Parkinson's disease | Inverse variance weighted | 4.13 | 7 | 7.70E-01 |  |  |
| FinnGen | Paraspinal AT | Parkinson's disease | MR Egger | 3.91 | 6 | 6.90E-01 | 0.015 | 6.60E-01 |
| FinnGen | Paraspinal AT | Peripheral artery disease | Inverse variance weighted | 14.50 | 7 | 4.30E-02 |  |  |
| FinnGen | Paraspinal AT | Peripheral artery disease | MR Egger | 9.28 | 6 | 1.60E-01 | 0.049 | 1.20E-01 |
| FinnGen | Paraspinal AT | Polycystic ovary syndrome | Inverse variance weighted | 11.42 | 7 | 1.20E-01 |  |  |
| FinnGen | Paraspinal AT | Polycystic ovary syndrome | MR Egger | 7.63 | 6 | 2.70E-01 | 0.107 | 1.40E-01 |
| FinnGen | Paraspinal AT | Psoriasis | Inverse variance weighted | 6.36 | 7 | 5.00E-01 |  |  |
| FinnGen | Paraspinal AT | Psoriasis | MR Egger | 6.17 | 6 | 4.00E-01 | 0.009 | 6.90E-01 |
| FinnGen | Paraspinal AT | Pulmonary embolism | Inverse variance weighted | 5.78 | 7 | 5.70E-01 |  |  |
| FinnGen | Paraspinal AT | Pulmonary embolism | MR Egger | 3.45 | 6 | 7.50E-01 | -0.034 | 1.80E-01 |
| FinnGen | Paraspinal AT | Rheumatoid arthritis | Inverse variance weighted | 6.63 | 7 | 4.70E-01 |  |  |
| FinnGen | Paraspinal AT | Rheumatoid arthritis | MR Egger | 6.54 | 6 | 3.70E-01 | 0.006 | 7.80E-01 |
| FinnGen | Paraspinal AT | Stroke | Inverse variance weighted | 5.18 | 7 | 6.40E-01 |  |  |
| FinnGen | Paraspinal AT | Stroke | MR Egger | 4.73 | 6 | 5.80E-01 | 0.014 | 5.30E-01 |
| FinnGen | Paraspinal AT | Type 2 diabetes | Inverse variance weighted | 399.07 | 7 | 3.80E-82 |  |  |
| FinnGen | Paraspinal AT | Type 2 diabetes | MR Egger | 263.56 | 6 | 5.20E-54 | 0.119 | 1.30E-01 |
| FinnGen | Pelvic bone marrow fat | Alzheimer's disease | Inverse variance weighted | 45.07 | 26 | 1.20E-02 |  |  |
| FinnGen | Pelvic bone marrow fat | Alzheimer's disease | MR Egger | 45.02 | 25 | 8.30E-03 | 0.002 | 8.70E-01 |
| FinnGen | Pelvic bone marrow fat | Aortic aneurysm | Inverse variance weighted | 43.37 | 26 | 1.80E-02 |  |  |
| FinnGen | Pelvic bone marrow fat | Aortic aneurysm | MR Egger | 41.88 | 25 | 1.90E-02 | -0.015 | 3.50E-01 |
| FinnGen | Pelvic bone marrow fat | Asthma | Inverse variance weighted | 91.85 | 26 | 2.80E-09 |  |  |
| FinnGen | Pelvic bone marrow fat | Asthma | MR Egger | 83.52 | 25 | 3.20E-08 | -0.017 | 1.30E-01 |
| FinnGen | Pelvic bone marrow fat | Atrial fibrillation | Inverse variance weighted | 123.86 | 26 | 1.00E-14 |  |  |
| FinnGen | Pelvic bone marrow fat | Atrial fibrillation | MR Egger | 122.85 | 25 | 6.90E-15 | -0.007 | 6.60E-01 |
| FinnGen | Pelvic bone marrow fat | Cholelithiasis | Inverse variance weighted | 120.45 | 26 | 4.10E-14 |  |  |
| FinnGen | Pelvic bone marrow fat | Cholelithiasis | MR Egger | 120.34 | 25 | 1.90E-14 | -0.002 | 8.80E-01 |
| FinnGen | Pelvic bone marrow fat | Chronic kidney disease | Inverse variance weighted | 37.53 | 26 | 6.70E-02 |  |  |
| FinnGen | Pelvic bone marrow fat | Chronic kidney disease | MR Egger | 37.24 | 25 | 5.50E-02 | -0.006 | 6.60E-01 |
| FinnGen | Pelvic bone marrow fat | Coronary artery disease | Inverse variance weighted | 75.08 | 26 | 1.20E-06 |  |  |
| FinnGen | Pelvic bone marrow fat | Coronary artery disease | MR Egger | 66.76 | 25 | 1.20E-05 | -0.026 | 9.00E-02 |
| FinnGen | Pelvic bone marrow fat | Deep vein thrombosis | Inverse variance weighted | 65.76 | 26 | 2.70E-05 |  |  |
| FinnGen | Pelvic bone marrow fat | Deep vein thrombosis | MR Egger | 64.14 | 25 | 2.70E-05 | 0.017 | 4.40E-01 |
| FinnGen | Pelvic bone marrow fat | Depression | Inverse variance weighted | 40.13 | 26 | 3.80E-02 |  |  |
| FinnGen | Pelvic bone marrow fat | Depression | MR Egger | 39.84 | 25 | 3.00E-02 | -0.003 | 6.70E-01 |
| FinnGen | Pelvic bone marrow fat | Gastroesophageal reflux disease | Inverse variance weighted | 48.30 | 26 | 5.00E-03 |  |  |
| FinnGen | Pelvic bone marrow fat | Gastroesophageal reflux disease | MR Egger | 47.70 | 25 | 4.10E-03 | -0.005 | 5.80E-01 |
| FinnGen | Pelvic bone marrow fat | Gout | Inverse variance weighted | 32.38 | 26 | 1.80E-01 |  |  |
| FinnGen | Pelvic bone marrow fat | Gout | MR Egger | 32.19 | 25 | 1.50E-01 | -0.007 | 7.00E-01 |
| FinnGen | Pelvic bone marrow fat | Heart failure | Inverse variance weighted | 47.94 | 26 | 5.50E-03 |  |  |
| FinnGen | Pelvic bone marrow fat | Heart failure | MR Egger | 39.77 | 25 | 3.10E-02 | -0.020 | 3.20E-02 |
| FinnGen | Pelvic bone marrow fat | Hip osteoarthritis | Inverse variance weighted | 60.28 | 26 | 1.50E-04 |  |  |
| FinnGen | Pelvic bone marrow fat | Hip osteoarthritis | MR Egger | 52.96 | 25 | 9.10E-04 | -0.020 | 7.50E-02 |
| FinnGen | Pelvic bone marrow fat | Hypertension | Inverse variance weighted | 219.50 | 26 | 1.60E-32 |  |  |
| FinnGen | Pelvic bone marrow fat | Hypertension | MR Egger | 214.25 | 25 | 5.40E-32 | -0.009 | 4.40E-01 |
| FinnGen | Pelvic bone marrow fat | Knee osteoarthritis | Inverse variance weighted | 121.69 | 26 | 2.50E-14 |  |  |
| FinnGen | Pelvic bone marrow fat | Knee osteoarthritis | MR Egger | 113.91 | 25 | 2.60E-13 | -0.016 | 2.00E-01 |
| FinnGen | Pelvic bone marrow fat | MASLD | Inverse variance weighted | 32.49 | 26 | 1.80E-01 |  |  |
| FinnGen | Pelvic bone marrow fat | MASLD | MR Egger | 30.68 | 25 | 2.00E-01 | -0.028 | 2.40E-01 |
| FinnGen | Pelvic bone marrow fat | Myocardial infarction | Inverse variance weighted | 92.35 | 26 | 2.30E-09 |  |  |
| FinnGen | Pelvic bone marrow fat | Myocardial infarction | MR Egger | 91.77 | 25 | 1.50E-09 | -0.006 | 7.00E-01 |
| FinnGen | Pelvic bone marrow fat | Osteoporosis | Inverse variance weighted | 109.66 | 26 | 3.00E-12 |  |  |
| FinnGen | Pelvic bone marrow fat | Osteoporosis | MR Egger | 109.50 | 25 | 1.50E-12 | -0.005 | 8.50E-01 |
| FinnGen | Pelvic bone marrow fat | Parkinson's disease | Inverse variance weighted | 23.36 | 26 | 6.10E-01 |  |  |
| FinnGen | Pelvic bone marrow fat | Parkinson's disease | MR Egger | 21.81 | 25 | 6.50E-01 | -0.020 | 2.30E-01 |
| FinnGen | Pelvic bone marrow fat | Peripheral artery disease | Inverse variance weighted | 87.04 | 26 | 1.70E-08 |  |  |
| FinnGen | Pelvic bone marrow fat | Peripheral artery disease | MR Egger | 84.89 | 25 | 1.90E-08 | -0.015 | 4.30E-01 |
| FinnGen | Pelvic bone marrow fat | Polycystic ovary syndrome | Inverse variance weighted | 36.80 | 26 | 7.80E-02 |  |  |
| FinnGen | Pelvic bone marrow fat | Polycystic ovary syndrome | MR Egger | 35.75 | 25 | 7.60E-02 | 0.027 | 4.00E-01 |
| FinnGen | Pelvic bone marrow fat | Psoriasis | Inverse variance weighted | 78.50 | 26 | 3.60E-07 |  |  |
| FinnGen | Pelvic bone marrow fat | Psoriasis | MR Egger | 74.17 | 25 | 9.10E-07 | -0.022 | 2.40E-01 |
| FinnGen | Pelvic bone marrow fat | Pulmonary embolism | Inverse variance weighted | 45.32 | 26 | 1.10E-02 |  |  |
| FinnGen | Pelvic bone marrow fat | Pulmonary embolism | MR Egger | 44.59 | 25 | 9.30E-03 | -0.009 | 5.30E-01 |
| FinnGen | Pelvic bone marrow fat | Rheumatoid arthritis | Inverse variance weighted | 94.34 | 26 | 1.10E-09 |  |  |
| FinnGen | Pelvic bone marrow fat | Rheumatoid arthritis | MR Egger | 94.16 | 25 | 5.90E-10 | 0.004 | 8.30E-01 |
| FinnGen | Pelvic bone marrow fat | Stroke | Inverse variance weighted | 34.43 | 26 | 1.30E-01 |  |  |
| FinnGen | Pelvic bone marrow fat | Stroke | MR Egger | 34.41 | 25 | 9.90E-02 | 0.001 | 9.10E-01 |
| FinnGen | Pelvic bone marrow fat | Type 2 diabetes | Inverse variance weighted | 182.78 | 26 | 1.70E-25 |  |  |
| FinnGen | Pelvic bone marrow fat | Type 2 diabetes | MR Egger | 182.68 | 25 | 6.30E-26 | 0.002 | 9.10E-01 |
| FinnGen | Thigh bone marrow fat | Alzheimer's disease | Inverse variance weighted | 28.23 | 28 | 4.50E-01 |  |  |
| FinnGen | Thigh bone marrow fat | Alzheimer's disease | MR Egger | 28.23 | 27 | 4.00E-01 | 0.000 | 9.70E-01 |
| FinnGen | Thigh bone marrow fat | Aortic aneurysm | Inverse variance weighted | 33.66 | 28 | 2.10E-01 |  |  |
| FinnGen | Thigh bone marrow fat | Aortic aneurysm | MR Egger | 28.13 | 27 | 4.00E-01 | -0.027 | 2.90E-02 |
| FinnGen | Thigh bone marrow fat | Asthma | Inverse variance weighted | 48.61 | 28 | 9.20E-03 |  |  |
| FinnGen | Thigh bone marrow fat | Asthma | MR Egger | 48.60 | 27 | 6.60E-03 | 0.000 | 9.60E-01 |
| FinnGen | Thigh bone marrow fat | Atrial fibrillation | Inverse variance weighted | 129.77 | 28 | 4.80E-15 |  |  |
| FinnGen | Thigh bone marrow fat | Atrial fibrillation | MR Egger | 125.93 | 27 | 1.00E-14 | -0.013 | 3.70E-01 |
| FinnGen | Thigh bone marrow fat | Cholelithiasis | Inverse variance weighted | 121.17 | 28 | 1.50E-13 |  |  |
| FinnGen | Thigh bone marrow fat | Cholelithiasis | MR Egger | 104.73 | 27 | 4.30E-11 | 0.023 | 4.90E-02 |
| FinnGen | Thigh bone marrow fat | Chronic kidney disease | Inverse variance weighted | 40.57 | 28 | 5.90E-02 |  |  |
| FinnGen | Thigh bone marrow fat | Chronic kidney disease | MR Egger | 36.72 | 27 | 1.00E-01 | 0.021 | 1.00E-01 |
| FinnGen | Thigh bone marrow fat | Coronary artery disease | Inverse variance weighted | 64.63 | 28 | 1.00E-04 |  |  |
| FinnGen | Thigh bone marrow fat | Coronary artery disease | MR Egger | 64.63 | 27 | 6.30E-05 | 0.000 | 9.80E-01 |
| FinnGen | Thigh bone marrow fat | Deep vein thrombosis | Inverse variance weighted | 38.99 | 28 | 8.10E-02 |  |  |
| FinnGen | Thigh bone marrow fat | Deep vein thrombosis | MR Egger | 38.37 | 27 | 7.20E-02 | -0.010 | 5.20E-01 |
| FinnGen | Thigh bone marrow fat | Depression | Inverse variance weighted | 52.75 | 28 | 3.10E-03 |  |  |
| FinnGen | Thigh bone marrow fat | Depression | MR Egger | 52.62 | 27 | 2.20E-03 | 0.002 | 8.00E-01 |
| FinnGen | Thigh bone marrow fat | Gastroesophageal reflux disease | Inverse variance weighted | 29.77 | 28 | 3.70E-01 |  |  |
| FinnGen | Thigh bone marrow fat | Gastroesophageal reflux disease | MR Egger | 28.78 | 27 | 3.70E-01 | -0.006 | 3.40E-01 |
| FinnGen | Thigh bone marrow fat | Gout | Inverse variance weighted | 44.07 | 28 | 2.70E-02 |  |  |
| FinnGen | Thigh bone marrow fat | Gout | MR Egger | 43.43 | 27 | 2.40E-02 | 0.012 | 5.40E-01 |
| FinnGen | Thigh bone marrow fat | Heart failure | Inverse variance weighted | 45.16 | 28 | 2.10E-02 |  |  |
| FinnGen | Thigh bone marrow fat | Heart failure | MR Egger | 40.02 | 27 | 5.10E-02 | -0.015 | 7.30E-02 |
| FinnGen | Thigh bone marrow fat | Hip osteoarthritis | Inverse variance weighted | 86.67 | 28 | 6.50E-08 |  |  |
| FinnGen | Thigh bone marrow fat | Hip osteoarthritis | MR Egger | 86.55 | 27 | 3.70E-08 | -0.003 | 8.50E-01 |
| FinnGen | Thigh bone marrow fat | Hypertension | Inverse variance weighted | 136.97 | 28 | 2.60E-16 |  |  |
| FinnGen | Thigh bone marrow fat | Hypertension | MR Egger | 136.96 | 27 | 1.10E-16 | 0.000 | 9.80E-01 |
| FinnGen | Thigh bone marrow fat | Knee osteoarthritis | Inverse variance weighted | 106.64 | 28 | 4.10E-11 |  |  |
| FinnGen | Thigh bone marrow fat | Knee osteoarthritis | MR Egger | 105.38 | 27 | 3.30E-11 | 0.006 | 5.80E-01 |
| FinnGen | Thigh bone marrow fat | MASLD | Inverse variance weighted | 56.28 | 28 | 1.20E-03 |  |  |
| FinnGen | Thigh bone marrow fat | MASLD | MR Egger | 50.49 | 27 | 4.00E-03 | 0.049 | 9.00E-02 |
| FinnGen | Thigh bone marrow fat | Myocardial infarction | Inverse variance weighted | 35.95 | 28 | 1.40E-01 |  |  |
| FinnGen | Thigh bone marrow fat | Myocardial infarction | MR Egger | 35.92 | 27 | 1.20E-01 | -0.001 | 8.70E-01 |
| FinnGen | Thigh bone marrow fat | Osteoporosis | Inverse variance weighted | 91.78 | 28 | 1.00E-08 |  |  |
| FinnGen | Thigh bone marrow fat | Osteoporosis | MR Egger | 89.77 | 27 | 1.20E-08 | -0.016 | 4.40E-01 |
| FinnGen | Thigh bone marrow fat | Parkinson's disease | Inverse variance weighted | 31.29 | 28 | 3.00E-01 |  |  |
| FinnGen | Thigh bone marrow fat | Parkinson's disease | MR Egger | 31.13 | 27 | 2.70E-01 | 0.006 | 7.10E-01 |
| FinnGen | Thigh bone marrow fat | Peripheral artery disease | Inverse variance weighted | 56.09 | 28 | 1.30E-03 |  |  |
| FinnGen | Thigh bone marrow fat | Peripheral artery disease | MR Egger | 55.69 | 27 | 9.40E-04 | 0.006 | 6.60E-01 |
| FinnGen | Thigh bone marrow fat | Polycystic ovary syndrome | Inverse variance weighted | 27.97 | 28 | 4.70E-01 |  |  |
| FinnGen | Thigh bone marrow fat | Polycystic ovary syndrome | MR Egger | 26.50 | 27 | 4.90E-01 | -0.031 | 2.40E-01 |
| FinnGen | Thigh bone marrow fat | Psoriasis | Inverse variance weighted | 30.28 | 28 | 3.50E-01 |  |  |
| FinnGen | Thigh bone marrow fat | Psoriasis | MR Egger | 25.86 | 27 | 5.30E-01 | 0.021 | 4.50E-02 |
| FinnGen | Thigh bone marrow fat | Pulmonary embolism | Inverse variance weighted | 45.28 | 28 | 2.10E-02 |  |  |
| FinnGen | Thigh bone marrow fat | Pulmonary embolism | MR Egger | 39.66 | 27 | 5.50E-02 | -0.025 | 6.10E-02 |
| FinnGen | Thigh bone marrow fat | Rheumatoid arthritis | Inverse variance weighted | 34.85 | 28 | 1.70E-01 |  |  |
| FinnGen | Thigh bone marrow fat | Rheumatoid arthritis | MR Egger | 34.85 | 27 | 1.40E-01 | 0.000 | 9.70E-01 |
| FinnGen | Thigh bone marrow fat | Stroke | Inverse variance weighted | 32.90 | 28 | 2.40E-01 |  |  |
| FinnGen | Thigh bone marrow fat | Stroke | MR Egger | 30.60 | 27 | 2.90E-01 | 0.015 | 1.70E-01 |
| FinnGen | Thigh bone marrow fat | Type 2 diabetes | Inverse variance weighted | 687.35 | 28 | 8.60E-127 |  |  |
| FinnGen | Thigh bone marrow fat | Type 2 diabetes | MR Egger | 593.25 | 27 | 7.30E-108 | 0.046 | 4.80E-02 |
| FinnGen | Vertebrae bone marrow fat | Alzheimer's disease | Inverse variance weighted | 11.42 | 4 | 2.20E-02 |  |  |
| FinnGen | Vertebrae bone marrow fat | Alzheimer's disease | MR Egger | 5.30 | 3 | 1.50E-01 | -0.182 | 1.60E-01 |
| FinnGen | Vertebrae bone marrow fat | Aortic aneurysm | Inverse variance weighted | 4.33 | 4 | 3.60E-01 |  |  |
| FinnGen | Vertebrae bone marrow fat | Aortic aneurysm | MR Egger | 1.12 | 3 | 7.70E-01 | -0.143 | 1.70E-01 |
| FinnGen | Vertebrae bone marrow fat | Asthma | Inverse variance weighted | 6.39 | 4 | 1.70E-01 |  |  |
| FinnGen | Vertebrae bone marrow fat | Asthma | MR Egger | 5.62 | 3 | 1.30E-01 | -0.034 | 5.70E-01 |
| FinnGen | Vertebrae bone marrow fat | Atrial fibrillation | Inverse variance weighted | 14.52 | 4 | 5.80E-03 |  |  |
| FinnGen | Vertebrae bone marrow fat | Atrial fibrillation | MR Egger | 14.14 | 3 | 2.70E-03 | 0.028 | 8.00E-01 |
| FinnGen | Vertebrae bone marrow fat | Cholelithiasis | Inverse variance weighted | 1.35 | 4 | 8.50E-01 |  |  |
| FinnGen | Vertebrae bone marrow fat | Cholelithiasis | MR Egger | 1.32 | 3 | 7.30E-01 | -0.007 | 8.60E-01 |
| FinnGen | Vertebrae bone marrow fat | Chronic kidney disease | Inverse variance weighted | 2.00 | 4 | 7.40E-01 |  |  |
| FinnGen | Vertebrae bone marrow fat | Chronic kidney disease | MR Egger | 0.58 | 3 | 9.00E-01 | 0.085 | 3.20E-01 |
| FinnGen | Vertebrae bone marrow fat | Coronary artery disease | Inverse variance weighted | 2.56 | 4 | 6.30E-01 |  |  |
| FinnGen | Vertebrae bone marrow fat | Coronary artery disease | MR Egger | 2.56 | 3 | 4.70E-01 | 0.000 | 1.00E+00 |
| FinnGen | Vertebrae bone marrow fat | Deep vein thrombosis | Inverse variance weighted | 10.43 | 4 | 3.40E-02 |  |  |
| FinnGen | Vertebrae bone marrow fat | Deep vein thrombosis | MR Egger | 10.07 | 3 | 1.80E-02 | 0.052 | 7.70E-01 |
| FinnGen | Vertebrae bone marrow fat | Depression | Inverse variance weighted | 6.73 | 4 | 1.50E-01 |  |  |
| FinnGen | Vertebrae bone marrow fat | Depression | MR Egger | 6.52 | 3 | 8.90E-02 | 0.016 | 7.80E-01 |
| FinnGen | Vertebrae bone marrow fat | Gastroesophageal reflux disease | Inverse variance weighted | 2.89 | 4 | 5.80E-01 |  |  |
| FinnGen | Vertebrae bone marrow fat | Gastroesophageal reflux disease | MR Egger | 0.40 | 3 | 9.40E-01 | -0.068 | 2.10E-01 |
| FinnGen | Vertebrae bone marrow fat | Gout | Inverse variance weighted | 6.44 | 4 | 1.70E-01 |  |  |
| FinnGen | Vertebrae bone marrow fat | Gout | MR Egger | 4.07 | 3 | 2.50E-01 | -0.152 | 2.80E-01 |
| FinnGen | Vertebrae bone marrow fat | Heart failure | Inverse variance weighted | 6.24 | 4 | 1.80E-01 |  |  |
| FinnGen | Vertebrae bone marrow fat | Heart failure | MR Egger | 3.00 | 3 | 3.90E-01 | -0.081 | 1.70E-01 |
| FinnGen | Vertebrae bone marrow fat | Hip osteoarthritis | Inverse variance weighted | 6.69 | 4 | 1.50E-01 |  |  |
| FinnGen | Vertebrae bone marrow fat | Hip osteoarthritis | MR Egger | 6.44 | 3 | 9.20E-02 | 0.025 | 7.50E-01 |
| FinnGen | Vertebrae bone marrow fat | Hypertension | Inverse variance weighted | 6.84 | 4 | 1.50E-01 |  |  |
| FinnGen | Vertebrae bone marrow fat | Hypertension | MR Egger | 6.18 | 3 | 1.00E-01 | 0.022 | 6.10E-01 |
| FinnGen | Vertebrae bone marrow fat | Knee osteoarthritis | Inverse variance weighted | 2.37 | 4 | 6.70E-01 |  |  |
| FinnGen | Vertebrae bone marrow fat | Knee osteoarthritis | MR Egger | 1.74 | 3 | 6.30E-01 | -0.029 | 4.90E-01 |
| FinnGen | Vertebrae bone marrow fat | MASLD | Inverse variance weighted | 0.99 | 4 | 9.10E-01 |  |  |
| FinnGen | Vertebrae bone marrow fat | MASLD | MR Egger | 0.93 | 3 | 8.20E-01 | 0.032 | 8.30E-01 |
| FinnGen | Vertebrae bone marrow fat | Myocardial infarction | Inverse variance weighted | 2.76 | 4 | 6.00E-01 |  |  |
| FinnGen | Vertebrae bone marrow fat | Myocardial infarction | MR Egger | 0.62 | 3 | 8.90E-01 | -0.073 | 2.40E-01 |
| FinnGen | Vertebrae bone marrow fat | Osteoporosis | Inverse variance weighted | 12.37 | 4 | 1.50E-02 |  |  |
| FinnGen | Vertebrae bone marrow fat | Osteoporosis | MR Egger | 11.83 | 3 | 8.00E-03 | -0.058 | 7.40E-01 |
| FinnGen | Vertebrae bone marrow fat | Parkinson's disease | Inverse variance weighted | 3.19 | 4 | 5.30E-01 |  |  |
| FinnGen | Vertebrae bone marrow fat | Parkinson's disease | MR Egger | 2.93 | 3 | 4.00E-01 | 0.052 | 6.50E-01 |
| FinnGen | Vertebrae bone marrow fat | Peripheral artery disease | Inverse variance weighted | 4.17 | 3 | 2.40E-01 |  |  |
| FinnGen | Vertebrae bone marrow fat | Peripheral artery disease | MR Egger | 4.16 | 2 | 1.30E-01 | -0.008 | 9.40E-01 |
| FinnGen | Vertebrae bone marrow fat | Polycystic ovary syndrome | Inverse variance weighted | 9.95 | 4 | 4.10E-02 |  |  |
| FinnGen | Vertebrae bone marrow fat | Polycystic ovary syndrome | MR Egger | 6.39 | 3 | 9.40E-02 | 0.328 | 2.90E-01 |
| FinnGen | Vertebrae bone marrow fat | Psoriasis | Inverse variance weighted | 2.93 | 4 | 5.70E-01 |  |  |
| FinnGen | Vertebrae bone marrow fat | Psoriasis | MR Egger | 2.62 | 3 | 4.50E-01 | 0.039 | 6.20E-01 |
| FinnGen | Vertebrae bone marrow fat | Pulmonary embolism | Inverse variance weighted | 19.52 | 4 | 6.20E-04 |  |  |
| FinnGen | Vertebrae bone marrow fat | Pulmonary embolism | MR Egger | 12.22 | 3 | 6.70E-03 | -0.191 | 2.70E-01 |
| FinnGen | Vertebrae bone marrow fat | Rheumatoid arthritis | Inverse variance weighted | 10.05 | 4 | 4.00E-02 |  |  |
| FinnGen | Vertebrae bone marrow fat | Rheumatoid arthritis | MR Egger | 8.12 | 3 | 4.40E-02 | 0.087 | 4.60E-01 |
| FinnGen | Vertebrae bone marrow fat | Stroke | Inverse variance weighted | 5.11 | 3 | 1.60E-01 |  |  |
| FinnGen | Vertebrae bone marrow fat | Stroke | MR Egger | 2.06 | 2 | 3.60E-01 | 0.120 | 2.30E-01 |
| FinnGen | Vertebrae bone marrow fat | Type 2 diabetes | Inverse variance weighted | 18.88 | 4 | 8.30E-04 |  |  |
| FinnGen | Vertebrae bone marrow fat | Type 2 diabetes | MR Egger | 8.69 | 3 | 3.40E-02 | 0.103 | 1.60E-01 |
| Pubgwas | Abdominal subcutaneous AT | Alzheimer's disease | Inverse variance weighted | 3.05 | 1 | 8.10E-02 |  |  |
| Pubgwas | Abdominal subcutaneous AT | Aortic aneurysm | Inverse variance weighted | 0.26 | 1 | 6.10E-01 |  |  |
| Pubgwas | Abdominal subcutaneous AT | Asthma | Inverse variance weighted | 0.25 | 1 | 6.20E-01 |  |  |
| Pubgwas | Abdominal subcutaneous AT | Atrial fibrillation | Inverse variance weighted | 6.75 | 1 | 9.40E-03 |  |  |
| Pubgwas | Abdominal subcutaneous AT | Cholelithiasis | Inverse variance weighted | 7.21 | 1 | 7.20E-03 |  |  |
| Pubgwas | Abdominal subcutaneous AT | Chronic kidney disease | Inverse variance weighted | 2.34 | 1 | 1.30E-01 |  |  |
| Pubgwas | Abdominal subcutaneous AT | Coronary artery disease | Inverse variance weighted | 10.26 | 1 | 1.40E-03 |  |  |
| Pubgwas | Abdominal subcutaneous AT | Deep vein thrombosis | Inverse variance weighted | 0.11 | 1 | 7.40E-01 |  |  |
| Pubgwas | Abdominal subcutaneous AT | Depression | Inverse variance weighted | 0.00 | 1 | 9.70E-01 |  |  |
| Pubgwas | Abdominal subcutaneous AT | Gastroesophageal reflux disease | Inverse variance weighted | 0.46 | 1 | 5.00E-01 |  |  |
| Pubgwas | Abdominal subcutaneous AT | Gout | Inverse variance weighted | 0.08 | 1 | 7.80E-01 |  |  |
| Pubgwas | Abdominal subcutaneous AT | Heart failure | Inverse variance weighted | 6.73 | 1 | 9.50E-03 |  |  |
| Pubgwas | Abdominal subcutaneous AT | Hip osteoarthritis | Inverse variance weighted | 2.30 | 1 | 1.30E-01 |  |  |
| Pubgwas | Abdominal subcutaneous AT | Hypertension | Inverse variance weighted | 7.82 | 1 | 5.20E-03 |  |  |
| Pubgwas | Abdominal subcutaneous AT | Knee osteoarthritis | Inverse variance weighted | 1.29 | 1 | 2.60E-01 |  |  |
| Pubgwas | Abdominal subcutaneous AT | MASLD | Inverse variance weighted | 1.42 | 1 | 2.30E-01 |  |  |
| Pubgwas | Abdominal subcutaneous AT | Myocardial infarction | Inverse variance weighted | 4.08 | 1 | 4.30E-02 |  |  |
| Pubgwas | Abdominal subcutaneous AT | Osteoporosis | Inverse variance weighted | 1.01 | 1 | 3.10E-01 |  |  |
| Pubgwas | Abdominal subcutaneous AT | Parkinson's disease | Inverse variance weighted | 3.25 | 1 | 7.20E-02 |  |  |
| Pubgwas | Abdominal subcutaneous AT | Peripheral artery disease | Inverse variance weighted | 8.75 | 1 | 3.10E-03 |  |  |
| Pubgwas | Abdominal subcutaneous AT | Polycystic ovary syndrome | Inverse variance weighted | 0.49 | 1 | 4.90E-01 |  |  |
| Pubgwas | Abdominal subcutaneous AT | Psoriasis | Inverse variance weighted | 13.06 | 1 | 3.00E-04 |  |  |
| Pubgwas | Abdominal subcutaneous AT | Pulmonary embolism | Inverse variance weighted | 0.10 | 1 | 7.50E-01 |  |  |
| Pubgwas | Abdominal subcutaneous AT | Rheumatoid arthritis | Inverse variance weighted | 8.30 | 1 | 4.00E-03 |  |  |
| Pubgwas | Abdominal subcutaneous AT | Stroke | Inverse variance weighted | 0.86 | 1 | 3.50E-01 |  |  |
| Pubgwas | Abdominal subcutaneous AT | Type 2 diabetes | Inverse variance weighted | 96.34 | 1 | 9.70E-23 |  |  |
| Pubgwas | Thigh subcutaneous AT | Alzheimer's disease | Inverse variance weighted | 22.76 | 20 | 3.00E-01 |  |  |
| Pubgwas | Thigh subcutaneous AT | Alzheimer's disease | MR Egger | 20.51 | 19 | 3.60E-01 | -0.011 | 1.70E-01 |
| Pubgwas | Thigh subcutaneous AT | Aortic aneurysm | Inverse variance weighted | 37.19 | 22 | 2.30E-02 |  |  |
| Pubgwas | Thigh subcutaneous AT | Aortic aneurysm | MR Egger | 37.19 | 21 | 1.60E-02 | -0.001 | 9.70E-01 |
| Pubgwas | Thigh subcutaneous AT | Asthma | Inverse variance weighted | 67.93 | 22 | 1.40E-06 |  |  |
| Pubgwas | Thigh subcutaneous AT | Asthma | MR Egger | 67.64 | 21 | 8.40E-07 | 0.003 | 7.70E-01 |
| Pubgwas | Thigh subcutaneous AT | Atrial fibrillation | Inverse variance weighted | 110.50 | 22 | 9.00E-14 |  |  |
| Pubgwas | Thigh subcutaneous AT | Atrial fibrillation | MR Egger | 105.49 | 21 | 3.00E-13 | 0.013 | 3.30E-01 |
| Pubgwas | Thigh subcutaneous AT | Cholelithiasis | Inverse variance weighted | 61.56 | 22 | 1.30E-05 |  |  |
| Pubgwas | Thigh subcutaneous AT | Cholelithiasis | MR Egger | 51.54 | 21 | 2.20E-04 | 0.023 | 5.60E-02 |
| Pubgwas | Thigh subcutaneous AT | Chronic kidney disease | Inverse variance weighted | 37.82 | 18 | 4.10E-03 |  |  |
| Pubgwas | Thigh subcutaneous AT | Chronic kidney disease | MR Egger | 37.66 | 17 | 2.70E-03 | -0.003 | 7.90E-01 |
| Pubgwas | Thigh subcutaneous AT | Coronary artery disease | Inverse variance weighted | 65.70 | 20 | 9.00E-07 |  |  |
| Pubgwas | Thigh subcutaneous AT | Coronary artery disease | MR Egger | 65.25 | 19 | 5.60E-07 | 0.006 | 7.20E-01 |
| Pubgwas | Thigh subcutaneous AT | Deep vein thrombosis | Inverse variance weighted | 23.99 | 22 | 3.50E-01 |  |  |
| Pubgwas | Thigh subcutaneous AT | Deep vein thrombosis | MR Egger | 23.94 | 21 | 3.00E-01 | 0.000 | 8.40E-01 |
| Pubgwas | Thigh subcutaneous AT | Depression | Inverse variance weighted | 20.59 | 22 | 5.50E-01 |  |  |
| Pubgwas | Thigh subcutaneous AT | Depression | MR Egger | 20.53 | 21 | 4.90E-01 | -0.003 | 8.20E-01 |
| Pubgwas | Thigh subcutaneous AT | Gastroesophageal reflux disease | Inverse variance weighted | 118.07 | 17 | 3.60E-17 |  |  |
| Pubgwas | Thigh subcutaneous AT | Gastroesophageal reflux disease | MR Egger | 115.87 | 16 | 3.40E-17 | -0.008 | 5.90E-01 |
| Pubgwas | Thigh subcutaneous AT | Gout | Inverse variance weighted | 16.90 | 17 | 4.60E-01 |  |  |
| Pubgwas | Thigh subcutaneous AT | Gout | MR Egger | 16.87 | 16 | 3.90E-01 | 0.007 | 8.60E-01 |
| Pubgwas | Thigh subcutaneous AT | Heart failure | Inverse variance weighted | 89.82 | 21 | 1.70E-10 |  |  |
| Pubgwas | Thigh subcutaneous AT | Heart failure | MR Egger | 89.65 | 20 | 8.60E-11 | 0.003 | 8.40E-01 |
| Pubgwas | Thigh subcutaneous AT | Hip osteoarthritis | Inverse variance weighted | 66.98 | 22 | 2.00E-06 |  |  |
| Pubgwas | Thigh subcutaneous AT | Hip osteoarthritis | MR Egger | 66.52 | 21 | 1.30E-06 | 0.007 | 7.10E-01 |
| Pubgwas | Thigh subcutaneous AT | Hypertension | Inverse variance weighted | 213.24 | 22 | 2.90E-33 |  |  |
| Pubgwas | Thigh subcutaneous AT | Hypertension | MR Egger | 201.70 | 21 | 1.70E-31 | 0.002 | 2.90E-01 |
| Pubgwas | Thigh subcutaneous AT | Knee osteoarthritis | Inverse variance weighted | 65.74 | 22 | 3.00E-06 |  |  |
| Pubgwas | Thigh subcutaneous AT | Knee osteoarthritis | MR Egger | 65.62 | 21 | 1.70E-06 | 0.003 | 8.50E-01 |
| Pubgwas | Thigh subcutaneous AT | MASLD | Inverse variance weighted | 54.76 | 19 | 2.50E-05 |  |  |
| Pubgwas | Thigh subcutaneous AT | MASLD | MR Egger | 47.17 | 18 | 2.00E-04 | 0.040 | 1.10E-01 |
| Pubgwas | Thigh subcutaneous AT | Myocardial infarction | Inverse variance weighted | 55.05 | 21 | 7.00E-05 |  |  |
| Pubgwas | Thigh subcutaneous AT | Myocardial infarction | MR Egger | 54.65 | 20 | 4.60E-05 | -0.006 | 7.10E-01 |
| Pubgwas | Thigh subcutaneous AT | Osteoporosis | Inverse variance weighted | 56.71 | 22 | 6.80E-05 |  |  |
| Pubgwas | Thigh subcutaneous AT | Osteoporosis | MR Egger | 38.61 | 21 | 1.10E-02 | -0.001 | 5.00E-03 |
| Pubgwas | Thigh subcutaneous AT | Parkinson's disease | Inverse variance weighted | 34.62 | 21 | 3.10E-02 |  |  |
| Pubgwas | Thigh subcutaneous AT | Parkinson's disease | MR Egger | 32.71 | 20 | 3.60E-02 | -0.022 | 2.90E-01 |
| Pubgwas | Thigh subcutaneous AT | Peripheral artery disease | Inverse variance weighted | 30.85 | 22 | 9.90E-02 |  |  |
| Pubgwas | Thigh subcutaneous AT | Peripheral artery disease | MR Egger | 28.84 | 21 | 1.20E-01 | 0.018 | 2.40E-01 |
| Pubgwas | Thigh subcutaneous AT | Polycystic ovary syndrome | Inverse variance weighted | 25.31 | 21 | 2.30E-01 |  |  |
| Pubgwas | Thigh subcutaneous AT | Polycystic ovary syndrome | MR Egger | 20.03 | 20 | 4.60E-01 | 0.048 | 3.30E-02 |
| Pubgwas | Thigh subcutaneous AT | Psoriasis | Inverse variance weighted | 34.78 | 19 | 1.50E-02 |  |  |
| Pubgwas | Thigh subcutaneous AT | Psoriasis | MR Egger | 34.70 | 18 | 1.00E-02 | -0.004 | 8.40E-01 |
| Pubgwas | Thigh subcutaneous AT | Pulmonary embolism | Inverse variance weighted | 37.04 | 22 | 2.30E-02 |  |  |
| Pubgwas | Thigh subcutaneous AT | Pulmonary embolism | MR Egger | 37.03 | 21 | 1.70E-02 | -0.002 | 9.40E-01 |
| Pubgwas | Thigh subcutaneous AT | Rheumatoid arthritis | Inverse variance weighted | 89.60 | 21 | 1.90E-10 |  |  |
| Pubgwas | Thigh subcutaneous AT | Rheumatoid arthritis | MR Egger | 87.24 | 20 | 2.20E-10 | -0.018 | 4.70E-01 |
| Pubgwas | Thigh subcutaneous AT | Stroke | Inverse variance weighted | 39.65 | 21 | 8.20E-03 |  |  |
| Pubgwas | Thigh subcutaneous AT | Stroke | MR Egger | 39.42 | 20 | 5.90E-03 | 0.004 | 7.40E-01 |
| Pubgwas | Thigh subcutaneous AT | Type 2 diabetes | Inverse variance weighted | 760.88 | 19 | 4.40E-149 |  |  |
| Pubgwas | Thigh subcutaneous AT | Type 2 diabetes | MR Egger | 755.79 | 18 | 8.00E-149 | 0.012 | 7.30E-01 |
| Pubgwas | Thigh intermuscular AT | Alzheimer's disease | Inverse variance weighted | 14.31 | 13 | 3.50E-01 |  |  |
| Pubgwas | Thigh intermuscular AT | Alzheimer's disease | MR Egger | 11.79 | 12 | 4.60E-01 | 0.033 | 4.90E-01 |
| Pubgwas | Thigh intermuscular AT | Aortic aneurysm | Inverse variance weighted | 16.86 | 13 | 2.10E-01 |  |  |
| Pubgwas | Thigh intermuscular AT | Aortic aneurysm | MR Egger | 16.37 | 12 | 1.70E-01 | 0.063 | 5.10E-01 |
| Pubgwas | Thigh intermuscular AT | Asthma | Inverse variance weighted | 10.92 | 13 | 6.20E-01 |  |  |
| Pubgwas | Thigh intermuscular AT | Asthma | MR Egger | 10.91 | 12 | 5.40E-01 | -0.022 | 4.40E-01 |
| Pubgwas | Thigh intermuscular AT | Atrial fibrillation | Inverse variance weighted | 34.45 | 13 | 1.00E-03 |  |  |
| Pubgwas | Thigh intermuscular AT | Atrial fibrillation | MR Egger | 30.49 | 12 | 2.40E-03 | 0.027 | 6.60E-01 |
| Pubgwas | Thigh intermuscular AT | Cholelithiasis | Inverse variance weighted | 15.23 | 13 | 2.90E-01 |  |  |
| Pubgwas | Thigh intermuscular AT | Cholelithiasis | MR Egger | 12.78 | 12 | 3.90E-01 | 0.033 | 6.10E-01 |
| Pubgwas | Thigh intermuscular AT | Chronic kidney disease | Inverse variance weighted | 27.13 | 13 | 1.20E-02 |  |  |
| Pubgwas | Thigh intermuscular AT | Chronic kidney disease | MR Egger | 25.92 | 12 | 1.10E-02 | 0.035 | 6.50E-01 |
| Pubgwas | Thigh intermuscular AT | Coronary artery disease | Inverse variance weighted | 19.97 | 13 | 9.60E-02 |  |  |
| Pubgwas | Thigh intermuscular AT | Coronary artery disease | MR Egger | 19.33 | 12 | 8.10E-02 | 0.168 | 7.80E-03 |
| Pubgwas | Thigh intermuscular AT | Deep vein thrombosis | Inverse variance weighted | 17.12 | 13 | 1.90E-01 |  |  |
| Pubgwas | Thigh intermuscular AT | Deep vein thrombosis | MR Egger | 16.93 | 12 | 1.50E-01 | 0.000 | 6.70E-01 |
| Pubgwas | Thigh intermuscular AT | Depression | Inverse variance weighted | 12.64 | 13 | 4.80E-01 |  |  |
| Pubgwas | Thigh intermuscular AT | Depression | MR Egger | 12.36 | 12 | 4.20E-01 | 0.073 | 1.80E-01 |
| Pubgwas | Thigh intermuscular AT | Gastroesophageal reflux disease | Inverse variance weighted | 45.70 | 12 | 7.80E-06 |  |  |
| Pubgwas | Thigh intermuscular AT | Gastroesophageal reflux disease | MR Egger | 45.46 | 11 | 4.00E-06 | 0.079 | 8.00E-02 |
| Pubgwas | Thigh intermuscular AT | Gout | Inverse variance weighted | 19.58 | 12 | 7.50E-02 |  |  |
| Pubgwas | Thigh intermuscular AT | Gout | MR Egger | 15.82 | 11 | 1.50E-01 | 0.174 | 3.40E-01 |
| Pubgwas | Thigh intermuscular AT | Heart failure | Inverse variance weighted | 4.84 | 13 | 9.80E-01 |  |  |
| Pubgwas | Thigh intermuscular AT | Heart failure | MR Egger | 4.81 | 12 | 9.60E-01 | 0.132 | 5.10E-02 |
| Pubgwas | Thigh intermuscular AT | Hip osteoarthritis | Inverse variance weighted | 9.54 | 13 | 7.30E-01 |  |  |
| Pubgwas | Thigh intermuscular AT | Hip osteoarthritis | MR Egger | 9.52 | 12 | 6.60E-01 | 0.125 | 6.60E-02 |
| Pubgwas | Thigh intermuscular AT | Hypertension | Inverse variance weighted | 68.44 | 13 | 1.60E-09 |  |  |
| Pubgwas | Thigh intermuscular AT | Hypertension | MR Egger | 66.76 | 12 | 1.30E-09 | 0.017 | 1.60E-01 |
| Pubgwas | Thigh intermuscular AT | Knee osteoarthritis | Inverse variance weighted | 28.63 | 13 | 7.40E-03 |  |  |
| Pubgwas | Thigh intermuscular AT | Knee osteoarthritis | MR Egger | 28.00 | 12 | 5.50E-03 | 0.131 | 1.70E-02 |
| Pubgwas | Thigh intermuscular AT | MASLD | Inverse variance weighted | 7.08 | 13 | 9.00E-01 |  |  |
| Pubgwas | Thigh intermuscular AT | MASLD | MR Egger | 6.46 | 12 | 8.90E-01 | 0.191 | 8.30E-02 |
| Pubgwas | Thigh intermuscular AT | Myocardial infarction | Inverse variance weighted | 24.24 | 13 | 2.90E-02 |  |  |
| Pubgwas | Thigh intermuscular AT | Myocardial infarction | MR Egger | 23.90 | 12 | 2.10E-02 | 0.061 | 3.80E-01 |
| Pubgwas | Thigh intermuscular AT | Osteoporosis | Inverse variance weighted | 28.81 | 13 | 7.00E-03 |  |  |
| Pubgwas | Thigh intermuscular AT | Osteoporosis | MR Egger | 27.97 | 12 | 5.60E-03 | 0.001 | 3.30E-01 |
| Pubgwas | Thigh intermuscular AT | Parkinson's disease | Inverse variance weighted | 8.72 | 13 | 7.90E-01 |  |  |
| Pubgwas | Thigh intermuscular AT | Parkinson's disease | MR Egger | 8.62 | 12 | 7.30E-01 | -0.133 | 1.80E-01 |
| Pubgwas | Thigh intermuscular AT | Peripheral artery disease | Inverse variance weighted | 21.18 | 13 | 6.90E-02 |  |  |
| Pubgwas | Thigh intermuscular AT | Peripheral artery disease | MR Egger | 19.65 | 12 | 7.40E-02 | 0.074 | 2.10E-01 |
| Pubgwas | Thigh intermuscular AT | Polycystic ovary syndrome | Inverse variance weighted | 23.71 | 13 | 3.40E-02 |  |  |
| Pubgwas | Thigh intermuscular AT | Polycystic ovary syndrome | MR Egger | 21.77 | 12 | 4.00E-02 | 0.164 | 1.10E-01 |
| Pubgwas | Thigh intermuscular AT | Psoriasis | Inverse variance weighted | 16.70 | 13 | 2.10E-01 |  |  |
| Pubgwas | Thigh intermuscular AT | Psoriasis | MR Egger | 16.66 | 12 | 1.60E-01 | 0.007 | 9.60E-01 |
| Pubgwas | Thigh intermuscular AT | Pulmonary embolism | Inverse variance weighted | 12.67 | 13 | 4.70E-01 |  |  |
| Pubgwas | Thigh intermuscular AT | Pulmonary embolism | MR Egger | 12.65 | 12 | 3.90E-01 | -0.053 | 5.50E-01 |
| Pubgwas | Thigh intermuscular AT | Rheumatoid arthritis | Inverse variance weighted | 28.83 | 13 | 6.90E-03 |  |  |
| Pubgwas | Thigh intermuscular AT | Rheumatoid arthritis | MR Egger | 26.87 | 12 | 8.10E-03 | -0.112 | 3.70E-01 |
| Pubgwas | Thigh intermuscular AT | Stroke | Inverse variance weighted | 20.17 | 13 | 9.10E-02 |  |  |
| Pubgwas | Thigh intermuscular AT | Stroke | MR Egger | 19.28 | 12 | 8.20E-02 | 0.063 | 1.60E-01 |
| Pubgwas | Thigh intermuscular AT | Type 2 diabetes | Inverse variance weighted | 163.07 | 13 | 4.70E-28 |  |  |
| Pubgwas | Thigh intermuscular AT | Type 2 diabetes | MR Egger | 157.27 | 12 | 1.90E-27 | 0.299 | 1.20E-01 |
| Pubgwas | Visceral AT | Alzheimer's disease | Inverse variance weighted | 10.73 | 5 | 5.70E-02 |  |  |
| Pubgwas | Visceral AT | Alzheimer's disease | MR Egger | 9.37 | 4 | 5.30E-02 | 0.021 | 3.50E-02 |
| Pubgwas | Visceral AT | Aortic aneurysm | Inverse variance weighted | 7.43 | 6 | 2.80E-01 |  |  |
| Pubgwas | Visceral AT | Aortic aneurysm | MR Egger | 6.76 | 5 | 2.40E-01 | 0.023 | 3.70E-01 |
| Pubgwas | Visceral AT | Asthma | Inverse variance weighted | 7.56 | 6 | 2.70E-01 |  |  |
| Pubgwas | Visceral AT | Asthma | MR Egger | 6.62 | 5 | 2.50E-01 | 0.012 | 1.50E-01 |
| Pubgwas | Visceral AT | Atrial fibrillation | Inverse variance weighted | 32.10 | 6 | 1.60E-05 |  |  |
| Pubgwas | Visceral AT | Atrial fibrillation | MR Egger | 30.77 | 5 | 1.00E-05 | 0.002 | 7.40E-01 |
| Pubgwas | Visceral AT | Cholelithiasis | Inverse variance weighted | 23.72 | 6 | 5.90E-04 |  |  |
| Pubgwas | Visceral AT | Cholelithiasis | MR Egger | 22.38 | 5 | 4.40E-04 | -0.028 | 3.30E-01 |
| Pubgwas | Visceral AT | Chronic kidney disease | Inverse variance weighted | 21.61 | 6 | 1.40E-03 |  |  |
| Pubgwas | Visceral AT | Chronic kidney disease | MR Egger | 20.62 | 5 | 9.50E-04 | -0.032 | 8.60E-02 |
| Pubgwas | Visceral AT | Coronary artery disease | Inverse variance weighted | 28.29 | 6 | 8.30E-05 |  |  |
| Pubgwas | Visceral AT | Coronary artery disease | MR Egger | 6.06 | 5 | 3.00E-01 | 0.024 | 3.30E-01 |
| Pubgwas | Visceral AT | Deep vein thrombosis | Inverse variance weighted | 5.73 | 6 | 4.50E-01 |  |  |
| Pubgwas | Visceral AT | Deep vein thrombosis | MR Egger | 5.52 | 5 | 3.60E-01 | 0.000 | 5.80E-01 |
| Pubgwas | Visceral AT | Depression | Inverse variance weighted | 6.33 | 6 | 3.90E-01 |  |  |
| Pubgwas | Visceral AT | Depression | MR Egger | 3.86 | 5 | 5.70E-01 | 0.003 | 7.80E-01 |
| Pubgwas | Visceral AT | Gastroesophageal reflux disease | Inverse variance weighted | 24.32 | 5 | 1.90E-04 |  |  |
| Pubgwas | Visceral AT | Gastroesophageal reflux disease | MR Egger | 10.31 | 4 | 3.60E-02 | -0.002 | 8.00E-01 |
| Pubgwas | Visceral AT | Gout | Inverse variance weighted | 8.05 | 5 | 1.50E-01 |  |  |
| Pubgwas | Visceral AT | Gout | MR Egger | 6.22 | 4 | 1.80E-01 | 0.074 | 4.20E-01 |
| Pubgwas | Visceral AT | Heart failure | Inverse variance weighted | 39.79 | 6 | 5.00E-07 |  |  |
| Pubgwas | Visceral AT | Heart failure | MR Egger | 17.31 | 5 | 3.90E-03 | 0.020 | 6.80E-02 |
| Pubgwas | Visceral AT | Hip osteoarthritis | Inverse variance weighted | 18.93 | 6 | 4.30E-03 |  |  |
| Pubgwas | Visceral AT | Hip osteoarthritis | MR Egger | 9.03 | 5 | 1.10E-01 | -0.006 | 7.00E-01 |
| Pubgwas | Visceral AT | Hypertension | Inverse variance weighted | 106.87 | 6 | 9.20E-21 |  |  |
| Pubgwas | Visceral AT | Hypertension | MR Egger | 69.82 | 5 | 1.10E-13 | 0.002 | 3.20E-01 |
| Pubgwas | Visceral AT | Knee osteoarthritis | Inverse variance weighted | 24.13 | 6 | 4.90E-04 |  |  |
| Pubgwas | Visceral AT | Knee osteoarthritis | MR Egger | 7.02 | 5 | 2.20E-01 | 0.005 | 6.90E-01 |
| Pubgwas | Visceral AT | MASLD | Inverse variance weighted | 24.44 | 6 | 4.30E-04 |  |  |
| Pubgwas | Visceral AT | MASLD | MR Egger | 12.64 | 5 | 2.70E-02 | 0.013 | 5.60E-01 |
| Pubgwas | Visceral AT | Myocardial infarction | Inverse variance weighted | 17.89 | 6 | 6.50E-03 |  |  |
| Pubgwas | Visceral AT | Myocardial infarction | MR Egger | 15.11 | 5 | 9.90E-03 | 0.040 | 2.30E-01 |
| Pubgwas | Visceral AT | Osteoporosis | Inverse variance weighted | 14.68 | 6 | 2.30E-02 |  |  |
| Pubgwas | Visceral AT | Osteoporosis | MR Egger | 11.88 | 5 | 3.60E-02 | 0.000 | 5.90E-01 |
| Pubgwas | Visceral AT | Parkinson's disease | Inverse variance weighted | 6.47 | 5 | 2.60E-01 |  |  |
| Pubgwas | Visceral AT | Parkinson's disease | MR Egger | 3.77 | 4 | 4.40E-01 | -0.050 | 4.10E-02 |
| Pubgwas | Visceral AT | Peripheral artery disease | Inverse variance weighted | 7.37 | 6 | 2.90E-01 |  |  |
| Pubgwas | Visceral AT | Peripheral artery disease | MR Egger | 5.23 | 5 | 3.90E-01 | 0.010 | 4.30E-01 |
| Pubgwas | Visceral AT | Polycystic ovary syndrome | Inverse variance weighted | 6.44 | 6 | 3.80E-01 |  |  |
| Pubgwas | Visceral AT | Polycystic ovary syndrome | MR Egger | 2.75 | 5 | 7.40E-01 | -0.011 | 5.80E-01 |
| Pubgwas | Visceral AT | Psoriasis | Inverse variance weighted | 25.54 | 6 | 2.70E-04 |  |  |
| Pubgwas | Visceral AT | Psoriasis | MR Egger | 25.53 | 5 | 1.10E-04 | 0.004 | 7.90E-01 |
| Pubgwas | Visceral AT | Pulmonary embolism | Inverse variance weighted | 3.65 | 6 | 7.20E-01 |  |  |
| Pubgwas | Visceral AT | Pulmonary embolism | MR Egger | 3.24 | 5 | 6.60E-01 | 0.026 | 3.40E-01 |
| Pubgwas | Visceral AT | Rheumatoid arthritis | Inverse variance weighted | 25.57 | 6 | 2.70E-04 |  |  |
| Pubgwas | Visceral AT | Rheumatoid arthritis | MR Egger | 21.46 | 5 | 6.60E-04 | 0.007 | 5.00E-01 |
| Pubgwas | Visceral AT | Stroke | Inverse variance weighted | 6.96 | 6 | 3.20E-01 |  |  |
| Pubgwas | Visceral AT | Stroke | MR Egger | 4.20 | 5 | 5.20E-01 | 0.014 | 1.40E-01 |
| Pubgwas | Visceral AT | Type 2 diabetes | Inverse variance weighted | 451.43 | 6 | 2.40E-94 |  |  |
| Pubgwas | Visceral AT | Type 2 diabetes | MR Egger | 266.34 | 5 | 1.70E-55 | -0.044 | 7.90E-02 |
| Pubgwas | Liver PDFF | Alzheimer's disease | Inverse variance weighted | 18.42 | 7 | 1.00E-02 |  |  |
| Pubgwas | Liver PDFF | Alzheimer's disease | MR Egger | 8.28 | 6 | 2.20E-01 | 0.008 | 5.00E-01 |
| Pubgwas | Liver PDFF | Aortic aneurysm | Inverse variance weighted | 15.19 | 8 | 5.60E-02 |  |  |
| Pubgwas | Liver PDFF | Aortic aneurysm | MR Egger | 13.40 | 7 | 6.30E-02 | 0.051 | 1.20E-01 |
| Pubgwas | Liver PDFF | Asthma | Inverse variance weighted | 21.79 | 8 | 5.30E-03 |  |  |
| Pubgwas | Liver PDFF | Asthma | MR Egger | 15.83 | 7 | 2.70E-02 | -0.008 | 5.90E-01 |
| Pubgwas | Liver PDFF | Atrial fibrillation | Inverse variance weighted | 10.14 | 8 | 2.60E-01 |  |  |
| Pubgwas | Liver PDFF | Atrial fibrillation | MR Egger | 9.97 | 7 | 1.90E-01 | 0.005 | 7.40E-01 |
| Pubgwas | Liver PDFF | Cholelithiasis | Inverse variance weighted | 141.76 | 8 | 1.00E-26 |  |  |
| Pubgwas | Liver PDFF | Cholelithiasis | MR Egger | 122.88 | 7 | 1.90E-23 | 0.000 | 9.90E-01 |
| Pubgwas | Liver PDFF | Chronic kidney disease | Inverse variance weighted | 44.48 | 7 | 1.70E-07 |  |  |
| Pubgwas | Liver PDFF | Chronic kidney disease | MR Egger | 26.17 | 6 | 2.10E-04 | 0.034 | 1.60E-01 |
| Pubgwas | Liver PDFF | Coronary artery disease | Inverse variance weighted | 64.14 | 7 | 2.20E-11 |  |  |
| Pubgwas | Liver PDFF | Coronary artery disease | MR Egger | 54.16 | 6 | 6.80E-10 | 0.020 | 3.50E-01 |
| Pubgwas | Liver PDFF | Deep vein thrombosis | Inverse variance weighted | 5.55 | 8 | 7.00E-01 |  |  |
| Pubgwas | Liver PDFF | Deep vein thrombosis | MR Egger | 5.22 | 7 | 6.30E-01 | -0.002 | 3.60E-01 |
| Pubgwas | Liver PDFF | Depression | Inverse variance weighted | 8.10 | 8 | 4.20E-01 |  |  |
| Pubgwas | Liver PDFF | Depression | MR Egger | 8.01 | 7 | 3.30E-01 | -0.001 | 9.50E-01 |
| Pubgwas | Liver PDFF | Gastroesophageal reflux disease | Inverse variance weighted | 8.59 | 4 | 7.20E-02 |  |  |
| Pubgwas | Liver PDFF | Gastroesophageal reflux disease | MR Egger | 8.37 | 3 | 3.90E-02 | -0.010 | 7.20E-01 |
| Pubgwas | Liver PDFF | Gout | Inverse variance weighted | 25.72 | 4 | 3.60E-05 |  |  |
| Pubgwas | Liver PDFF | Gout | MR Egger | 19.90 | 3 | 1.80E-04 | -0.044 | 5.80E-01 |
| Pubgwas | Liver PDFF | Heart failure | Inverse variance weighted | 23.11 | 7 | 1.60E-03 |  |  |
| Pubgwas | Liver PDFF | Heart failure | MR Egger | 12.67 | 6 | 4.90E-02 | 0.000 | 1.00E+00 |
| Pubgwas | Liver PDFF | Hip osteoarthritis | Inverse variance weighted | 16.27 | 8 | 3.90E-02 |  |  |
| Pubgwas | Liver PDFF | Hip osteoarthritis | MR Egger | 15.89 | 7 | 2.60E-02 | -0.010 | 5.70E-01 |
| Pubgwas | Liver PDFF | Hypertension | Inverse variance weighted | 80.89 | 8 | 3.20E-14 |  |  |
| Pubgwas | Liver PDFF | Hypertension | MR Egger | 69.55 | 7 | 1.80E-12 | 0.008 | 3.20E-02 |
| Pubgwas | Liver PDFF | Knee osteoarthritis | Inverse variance weighted | 16.38 | 8 | 3.70E-02 |  |  |
| Pubgwas | Liver PDFF | Knee osteoarthritis | MR Egger | 15.98 | 7 | 2.50E-02 | 0.000 | 9.90E-01 |
| Pubgwas | Liver PDFF | MASLD | Inverse variance weighted | 13.72 | 6 | 3.30E-02 |  |  |
| Pubgwas | Liver PDFF | MASLD | MR Egger | 12.73 | 5 | 2.60E-02 | 0.043 | 2.00E-01 |
| Pubgwas | Liver PDFF | Myocardial infarction | Inverse variance weighted | 113.10 | 8 | 8.80E-21 |  |  |
| Pubgwas | Liver PDFF | Myocardial infarction | MR Egger | 90.51 | 7 | 9.70E-17 | 0.021 | 4.90E-01 |
| Pubgwas | Liver PDFF | Osteoporosis | Inverse variance weighted | 10.49 | 8 | 2.30E-01 |  |  |
| Pubgwas | Liver PDFF | Osteoporosis | MR Egger | 10.03 | 7 | 1.90E-01 | 0.000 | 4.90E-01 |
| Pubgwas | Liver PDFF | Parkinson's disease | Inverse variance weighted | 27.78 | 8 | 5.20E-04 |  |  |
| Pubgwas | Liver PDFF | Parkinson's disease | MR Egger | 14.70 | 7 | 4.00E-02 | -0.043 | 1.80E-01 |
| Pubgwas | Liver PDFF | Peripheral artery disease | Inverse variance weighted | 8.93 | 8 | 3.50E-01 |  |  |
| Pubgwas | Liver PDFF | Peripheral artery disease | MR Egger | 8.12 | 7 | 3.20E-01 | 0.017 | 5.90E-01 |
| Pubgwas | Liver PDFF | Polycystic ovary syndrome | Inverse variance weighted | 4.36 | 8 | 8.20E-01 |  |  |
| Pubgwas | Liver PDFF | Polycystic ovary syndrome | MR Egger | 4.02 | 7 | 7.80E-01 | -0.007 | 8.70E-01 |
| Pubgwas | Liver PDFF | Psoriasis | Inverse variance weighted | 5.68 | 7 | 5.80E-01 |  |  |
| Pubgwas | Liver PDFF | Psoriasis | MR Egger | 5.60 | 6 | 4.70E-01 | 0.054 | 3.20E-01 |
| Pubgwas | Liver PDFF | Pulmonary embolism | Inverse variance weighted | 14.82 | 8 | 6.30E-02 |  |  |
| Pubgwas | Liver PDFF | Pulmonary embolism | MR Egger | 12.90 | 7 | 7.50E-02 | -0.006 | 9.60E-01 |
| Pubgwas | Liver PDFF | Rheumatoid arthritis | Inverse variance weighted | 4.80 | 7 | 6.80E-01 |  |  |
| Pubgwas | Liver PDFF | Rheumatoid arthritis | MR Egger | 4.29 | 6 | 6.40E-01 | -0.022 | 4.10E-01 |
| Pubgwas | Liver PDFF | Stroke | Inverse variance weighted | 6.31 | 7 | 5.00E-01 |  |  |
| Pubgwas | Liver PDFF | Stroke | MR Egger | 3.36 | 6 | 7.60E-01 | 0.015 | 3.60E-01 |
| Pubgwas | Liver PDFF | Type 2 diabetes | Inverse variance weighted | 206.44 | 8 | 2.80E-40 |  |  |
| Pubgwas | Liver PDFF | Type 2 diabetes | MR Egger | 128.90 | 7 | 1.10E-24 | 0.017 | 6.90E-01 |
| Pubgwas | Pancreas PDFF | Alzheimer's disease | Inverse variance weighted | 16.43 | 12 | 1.70E-01 |  |  |
| Pubgwas | Pancreas PDFF | Alzheimer's disease | MR Egger | 15.74 | 11 | 1.50E-01 | 0.004 | 8.00E-01 |
| Pubgwas | Pancreas PDFF | Aortic aneurysm | Inverse variance weighted | 11.95 | 12 | 4.50E-01 |  |  |
| Pubgwas | Pancreas PDFF | Aortic aneurysm | MR Egger | 9.16 | 11 | 6.10E-01 | 0.006 | 8.70E-01 |
| Pubgwas | Pancreas PDFF | Asthma | Inverse variance weighted | 41.84 | 12 | 3.60E-05 |  |  |
| Pubgwas | Pancreas PDFF | Asthma | MR Egger | 40.67 | 11 | 2.80E-05 | -0.002 | 8.80E-01 |
| Pubgwas | Pancreas PDFF | Atrial fibrillation | Inverse variance weighted | 26.58 | 12 | 8.90E-03 |  |  |
| Pubgwas | Pancreas PDFF | Atrial fibrillation | MR Egger | 26.31 | 11 | 5.80E-03 | 0.019 | 3.60E-01 |
| Pubgwas | Pancreas PDFF | Cholelithiasis | Inverse variance weighted | 82.37 | 12 | 1.50E-12 |  |  |
| Pubgwas | Pancreas PDFF | Cholelithiasis | MR Egger | 82.37 | 11 | 5.10E-13 | 0.040 | 9.00E-02 |
| Pubgwas | Pancreas PDFF | Chronic kidney disease | Inverse variance weighted | 17.27 | 8 | 2.70E-02 |  |  |
| Pubgwas | Pancreas PDFF | Chronic kidney disease | MR Egger | 12.75 | 7 | 7.80E-02 | -0.015 | 5.40E-01 |
| Pubgwas | Pancreas PDFF | Coronary artery disease | Inverse variance weighted | 16.96 | 10 | 7.50E-02 |  |  |
| Pubgwas | Pancreas PDFF | Coronary artery disease | MR Egger | 15.33 | 9 | 8.20E-02 | 0.012 | 6.40E-01 |
| Pubgwas | Pancreas PDFF | Deep vein thrombosis | Inverse variance weighted | 318.22 | 12 | 7.00E-61 |  |  |
| Pubgwas | Pancreas PDFF | Deep vein thrombosis | MR Egger | 293.45 | 11 | 2.10E-56 | 0.000 | 8.70E-01 |
| Pubgwas | Pancreas PDFF | Depression | Inverse variance weighted | 9.75 | 12 | 6.40E-01 |  |  |
| Pubgwas | Pancreas PDFF | Depression | MR Egger | 9.75 | 11 | 5.50E-01 | 0.005 | 8.10E-01 |
| Pubgwas | Pancreas PDFF | Gastroesophageal reflux disease | Inverse variance weighted | 9.80 | 4 | 4.40E-02 |  |  |
| Pubgwas | Pancreas PDFF | Gastroesophageal reflux disease | MR Egger | 9.31 | 3 | 2.50E-02 | 0.006 | 8.20E-01 |
| Pubgwas | Pancreas PDFF | Gout | Inverse variance weighted | 1.54 | 5 | 9.10E-01 |  |  |
| Pubgwas | Pancreas PDFF | Gout | MR Egger | 1.16 | 4 | 8.80E-01 | 0.188 | 4.10E-02 |
| Pubgwas | Pancreas PDFF | Heart failure | Inverse variance weighted | 52.76 | 11 | 2.00E-07 |  |  |
| Pubgwas | Pancreas PDFF | Heart failure | MR Egger | 52.76 | 10 | 8.30E-08 | 0.013 | 6.70E-01 |
| Pubgwas | Pancreas PDFF | Hip osteoarthritis | Inverse variance weighted | 14.46 | 12 | 2.70E-01 |  |  |
| Pubgwas | Pancreas PDFF | Hip osteoarthritis | MR Egger | 14.03 | 11 | 2.30E-01 | 0.017 | 6.60E-01 |
| Pubgwas | Pancreas PDFF | Hypertension | Inverse variance weighted | 157.75 | 12 | 1.50E-27 |  |  |
| Pubgwas | Pancreas PDFF | Hypertension | MR Egger | 101.83 | 11 | 7.70E-17 | 0.001 | 7.20E-01 |
| Pubgwas | Pancreas PDFF | Knee osteoarthritis | Inverse variance weighted | 19.51 | 12 | 7.70E-02 |  |  |
| Pubgwas | Pancreas PDFF | Knee osteoarthritis | MR Egger | 19.51 | 11 | 5.20E-02 | 0.026 | 4.20E-01 |
| Pubgwas | Pancreas PDFF | MASLD | Inverse variance weighted | 16.23 | 10 | 9.30E-02 |  |  |
| Pubgwas | Pancreas PDFF | MASLD | MR Egger | 13.33 | 9 | 1.50E-01 | 0.094 | 2.50E-02 |
| Pubgwas | Pancreas PDFF | Myocardial infarction | Inverse variance weighted | 33.61 | 11 | 4.20E-04 |  |  |
| Pubgwas | Pancreas PDFF | Myocardial infarction | MR Egger | 31.98 | 10 | 4.00E-04 | -0.010 | 6.20E-01 |
| Pubgwas | Pancreas PDFF | Osteoporosis | Inverse variance weighted | 13.98 | 12 | 3.00E-01 |  |  |
| Pubgwas | Pancreas PDFF | Osteoporosis | MR Egger | 13.35 | 11 | 2.70E-01 | -0.001 | 6.50E-02 |
| Pubgwas | Pancreas PDFF | Parkinson's disease | Inverse variance weighted | 9.06 | 10 | 5.30E-01 |  |  |
| Pubgwas | Pancreas PDFF | Parkinson's disease | MR Egger | 6.96 | 9 | 6.40E-01 | -0.016 | 6.10E-01 |
| Pubgwas | Pancreas PDFF | Peripheral artery disease | Inverse variance weighted | 29.16 | 12 | 3.70E-03 |  |  |
| Pubgwas | Pancreas PDFF | Peripheral artery disease | MR Egger | 28.37 | 11 | 2.80E-03 | 0.062 | 8.50E-02 |
| Pubgwas | Pancreas PDFF | Polycystic ovary syndrome | Inverse variance weighted | 16.26 | 12 | 1.80E-01 |  |  |
| Pubgwas | Pancreas PDFF | Polycystic ovary syndrome | MR Egger | 16.22 | 11 | 1.30E-01 | 0.105 | 7.30E-02 |
| Pubgwas | Pancreas PDFF | Psoriasis | Inverse variance weighted | 37.03 | 10 | 5.60E-05 |  |  |
| Pubgwas | Pancreas PDFF | Psoriasis | MR Egger | 32.92 | 9 | 1.40E-04 | 0.044 | 4.00E-01 |
| Pubgwas | Pancreas PDFF | Pulmonary embolism | Inverse variance weighted | 175.51 | 12 | 3.60E-31 |  |  |
| Pubgwas | Pancreas PDFF | Pulmonary embolism | MR Egger | 175.47 | 11 | 8.80E-32 | -0.013 | 7.60E-01 |
| Pubgwas | Pancreas PDFF | Rheumatoid arthritis | Inverse variance weighted | 20.00 | 12 | 6.70E-02 |  |  |
| Pubgwas | Pancreas PDFF | Rheumatoid arthritis | MR Egger | 18.76 | 11 | 6.50E-02 | 0.002 | 9.50E-01 |
| Pubgwas | Pancreas PDFF | Stroke | Inverse variance weighted | 7.57 | 10 | 6.70E-01 |  |  |
| Pubgwas | Pancreas PDFF | Stroke | MR Egger | 6.62 | 9 | 6.80E-01 | -0.025 | 3.50E-01 |
| Pubgwas | Pancreas PDFF | Type 2 diabetes | Inverse variance weighted | 128.49 | 9 | 2.40E-23 |  |  |
| Pubgwas | Pancreas PDFF | Type 2 diabetes | MR Egger | 125.83 | 8 | 2.10E-23 | 0.150 | 6.30E-02 |
| Pubgwas | Paraspinal AT | Alzheimer's disease | Inverse variance weighted | 7.82 | 7 | 3.50E-01 |  |  |
| Pubgwas | Paraspinal AT | Alzheimer's disease | MR Egger | 7.73 | 6 | 2.60E-01 | 0.019 | 1.40E-01 |
| Pubgwas | Paraspinal AT | Aortic aneurysm | Inverse variance weighted | 4.26 | 7 | 7.50E-01 |  |  |
| Pubgwas | Paraspinal AT | Aortic aneurysm | MR Egger | 4.23 | 6 | 6.50E-01 | -0.024 | 5.60E-01 |
| Pubgwas | Paraspinal AT | Asthma | Inverse variance weighted | 5.78 | 7 | 5.70E-01 |  |  |
| Pubgwas | Paraspinal AT | Asthma | MR Egger | 5.75 | 6 | 4.50E-01 | -0.001 | 9.30E-01 |
| Pubgwas | Paraspinal AT | Atrial fibrillation | Inverse variance weighted | 22.80 | 7 | 1.80E-03 |  |  |
| Pubgwas | Paraspinal AT | Atrial fibrillation | MR Egger | 19.64 | 6 | 3.20E-03 | -0.020 | 2.40E-01 |
| Pubgwas | Paraspinal AT | Cholelithiasis | Inverse variance weighted | 22.98 | 7 | 1.70E-03 |  |  |
| Pubgwas | Paraspinal AT | Cholelithiasis | MR Egger | 13.69 | 6 | 3.30E-02 | 0.020 | 1.60E-01 |
| Pubgwas | Paraspinal AT | Chronic kidney disease | Inverse variance weighted | 15.01 | 7 | 3.60E-02 |  |  |
| Pubgwas | Paraspinal AT | Chronic kidney disease | MR Egger | 14.00 | 6 | 3.00E-02 | -0.015 | 4.70E-01 |
| Pubgwas | Paraspinal AT | Coronary artery disease | Inverse variance weighted | 16.03 | 7 | 2.50E-02 |  |  |
| Pubgwas | Paraspinal AT | Coronary artery disease | MR Egger | 15.40 | 6 | 1.70E-02 | -0.011 | 5.40E-01 |
| Pubgwas | Paraspinal AT | Deep vein thrombosis | Inverse variance weighted | 18.47 | 7 | 1.00E-02 |  |  |
| Pubgwas | Paraspinal AT | Deep vein thrombosis | MR Egger | 18.38 | 6 | 5.40E-03 | 0.000 | 7.20E-01 |
| Pubgwas | Paraspinal AT | Depression | Inverse variance weighted | 3.57 | 7 | 8.30E-01 |  |  |
| Pubgwas | Paraspinal AT | Depression | MR Egger | 3.51 | 6 | 7.40E-01 | -0.011 | 6.10E-01 |
| Pubgwas | Paraspinal AT | Gastroesophageal reflux disease | Inverse variance weighted | 26.94 | 5 | 5.90E-05 |  |  |
| Pubgwas | Paraspinal AT | Gastroesophageal reflux disease | MR Egger | 26.55 | 4 | 2.50E-05 | -0.004 | 8.20E-01 |
| Pubgwas | Paraspinal AT | Gout | Inverse variance weighted | 10.69 | 5 | 5.80E-02 |  |  |
| Pubgwas | Paraspinal AT | Gout | MR Egger | 1.79 | 4 | 7.70E-01 | 0.096 | 1.30E-01 |
| Pubgwas | Paraspinal AT | Heart failure | Inverse variance weighted | 34.03 | 7 | 1.70E-05 |  |  |
| Pubgwas | Paraspinal AT | Heart failure | MR Egger | 32.93 | 6 | 1.10E-05 | -0.002 | 8.70E-01 |
| Pubgwas | Paraspinal AT | Hip osteoarthritis | Inverse variance weighted | 23.75 | 7 | 1.30E-03 |  |  |
| Pubgwas | Paraspinal AT | Hip osteoarthritis | MR Egger | 22.94 | 6 | 8.20E-04 | -0.003 | 8.80E-01 |
| Pubgwas | Paraspinal AT | Hypertension | Inverse variance weighted | 47.69 | 7 | 4.10E-08 |  |  |
| Pubgwas | Paraspinal AT | Hypertension | MR Egger | 46.55 | 6 | 2.30E-08 | 0.002 | 5.90E-01 |
| Pubgwas | Paraspinal AT | Knee osteoarthritis | Inverse variance weighted | 26.42 | 7 | 4.20E-04 |  |  |
| Pubgwas | Paraspinal AT | Knee osteoarthritis | MR Egger | 23.44 | 6 | 6.60E-04 | 0.011 | 6.10E-01 |
| Pubgwas | Paraspinal AT | MASLD | Inverse variance weighted | 22.98 | 7 | 1.70E-03 |  |  |
| Pubgwas | Paraspinal AT | MASLD | MR Egger | 9.31 | 6 | 1.60E-01 | 0.019 | 4.50E-01 |
| Pubgwas | Paraspinal AT | Myocardial infarction | Inverse variance weighted | 8.33 | 7 | 3.00E-01 |  |  |
| Pubgwas | Paraspinal AT | Myocardial infarction | MR Egger | 7.96 | 6 | 2.40E-01 | -0.009 | 6.90E-01 |
| Pubgwas | Paraspinal AT | Osteoporosis | Inverse variance weighted | 12.21 | 7 | 9.40E-02 |  |  |
| Pubgwas | Paraspinal AT | Osteoporosis | MR Egger | 6.60 | 6 | 3.60E-01 | 0.000 | 5.60E-01 |
| Pubgwas | Paraspinal AT | Parkinson's disease | Inverse variance weighted | 5.48 | 7 | 6.00E-01 |  |  |
| Pubgwas | Paraspinal AT | Parkinson's disease | MR Egger | 5.19 | 6 | 5.20E-01 | -0.009 | 7.60E-01 |
| Pubgwas | Paraspinal AT | Peripheral artery disease | Inverse variance weighted | 18.14 | 7 | 1.10E-02 |  |  |
| Pubgwas | Paraspinal AT | Peripheral artery disease | MR Egger | 10.62 | 6 | 1.00E-01 | 0.028 | 3.50E-01 |
| Pubgwas | Paraspinal AT | Polycystic ovary syndrome | Inverse variance weighted | 17.00 | 7 | 1.70E-02 |  |  |
| Pubgwas | Paraspinal AT | Polycystic ovary syndrome | MR Egger | 9.51 | 6 | 1.50E-01 | 0.051 | 3.20E-01 |
| Pubgwas | Paraspinal AT | Psoriasis | Inverse variance weighted | 20.37 | 7 | 4.80E-03 |  |  |
| Pubgwas | Paraspinal AT | Psoriasis | MR Egger | 17.90 | 6 | 6.50E-03 | -0.005 | 8.80E-01 |
| Pubgwas | Paraspinal AT | Pulmonary embolism | Inverse variance weighted | 6.59 | 7 | 4.70E-01 |  |  |
| Pubgwas | Paraspinal AT | Pulmonary embolism | MR Egger | 6.48 | 6 | 3.70E-01 | 0.004 | 9.20E-01 |
| Pubgwas | Paraspinal AT | Rheumatoid arthritis | Inverse variance weighted | 13.86 | 7 | 5.40E-02 |  |  |
| Pubgwas | Paraspinal AT | Rheumatoid arthritis | MR Egger | 13.84 | 6 | 3.10E-02 | 0.030 | 3.70E-01 |
| Pubgwas | Paraspinal AT | Stroke | Inverse variance weighted | 17.38 | 7 | 1.50E-02 |  |  |
| Pubgwas | Paraspinal AT | Stroke | MR Egger | 14.81 | 6 | 2.20E-02 | 0.014 | 4.70E-01 |
| Pubgwas | Paraspinal AT | Type 2 diabetes | Inverse variance weighted | 471.32 | 7 | 1.20E-97 |  |  |
| Pubgwas | Paraspinal AT | Type 2 diabetes | MR Egger | 252.43 | 6 | 1.20E-51 | 0.023 | 5.20E-01 |
| Pubgwas | Pelvic bone marrow fat | Alzheimer's disease | Inverse variance weighted | 49.20 | 27 | 5.60E-03 |  |  |
| Pubgwas | Pelvic bone marrow fat | Alzheimer's disease | MR Egger | 48.49 | 26 | 4.70E-03 | 0.004 | 5.40E-01 |
| Pubgwas | Pelvic bone marrow fat | Aortic aneurysm | Inverse variance weighted | 31.85 | 28 | 2.80E-01 |  |  |
| Pubgwas | Pelvic bone marrow fat | Aortic aneurysm | MR Egger | 29.44 | 27 | 3.40E-01 | 0.023 | 1.50E-01 |
| Pubgwas | Pelvic bone marrow fat | Asthma | Inverse variance weighted | 155.69 | 28 | 1.20E-19 |  |  |
| Pubgwas | Pelvic bone marrow fat | Asthma | MR Egger | 153.90 | 27 | 1.00E-19 | -0.006 | 5.80E-01 |
| Pubgwas | Pelvic bone marrow fat | Atrial fibrillation | Inverse variance weighted | 87.27 | 28 | 5.30E-08 |  |  |
| Pubgwas | Pelvic bone marrow fat | Atrial fibrillation | MR Egger | 84.29 | 27 | 8.30E-08 | -0.008 | 3.40E-01 |
| Pubgwas | Pelvic bone marrow fat | Cholelithiasis | Inverse variance weighted | 51.93 | 28 | 3.90E-03 |  |  |
| Pubgwas | Pelvic bone marrow fat | Cholelithiasis | MR Egger | 49.57 | 27 | 5.10E-03 | -0.008 | 2.70E-01 |
| Pubgwas | Pelvic bone marrow fat | Chronic kidney disease | Inverse variance weighted | 44.80 | 27 | 1.70E-02 |  |  |
| Pubgwas | Pelvic bone marrow fat | Chronic kidney disease | MR Egger | 44.17 | 26 | 1.40E-02 | -0.005 | 5.50E-01 |
| Pubgwas | Pelvic bone marrow fat | Coronary artery disease | Inverse variance weighted | 89.10 | 27 | 1.50E-08 |  |  |
| Pubgwas | Pelvic bone marrow fat | Coronary artery disease | MR Egger | 89.10 | 26 | 7.80E-09 | 0.000 | 9.80E-01 |
| Pubgwas | Pelvic bone marrow fat | Deep vein thrombosis | Inverse variance weighted | 57.10 | 28 | 9.40E-04 |  |  |
| Pubgwas | Pelvic bone marrow fat | Deep vein thrombosis | MR Egger | 56.85 | 27 | 6.70E-04 | 0.000 | 7.30E-01 |
| Pubgwas | Pelvic bone marrow fat | Depression | Inverse variance weighted | 31.99 | 28 | 2.70E-01 |  |  |
| Pubgwas | Pelvic bone marrow fat | Depression | MR Egger | 31.97 | 27 | 2.30E-01 | -0.001 | 9.00E-01 |
| Pubgwas | Pelvic bone marrow fat | Gastroesophageal reflux disease | Inverse variance weighted | 46.06 | 18 | 2.90E-04 |  |  |
| Pubgwas | Pelvic bone marrow fat | Gastroesophageal reflux disease | MR Egger | 42.61 | 17 | 5.50E-04 | -0.007 | 2.60E-01 |
| Pubgwas | Pelvic bone marrow fat | Gout | Inverse variance weighted | 16.65 | 20 | 6.80E-01 |  |  |
| Pubgwas | Pelvic bone marrow fat | Gout | MR Egger | 14.63 | 19 | 7.50E-01 | 0.033 | 1.70E-01 |
| Pubgwas | Pelvic bone marrow fat | Heart failure | Inverse variance weighted | 62.48 | 27 | 1.20E-04 |  |  |
| Pubgwas | Pelvic bone marrow fat | Heart failure | MR Egger | 62.41 | 26 | 7.90E-05 | 0.001 | 8.70E-01 |
| Pubgwas | Pelvic bone marrow fat | Hip osteoarthritis | Inverse variance weighted | 74.34 | 28 | 4.50E-06 |  |  |
| Pubgwas | Pelvic bone marrow fat | Hip osteoarthritis | MR Egger | 70.13 | 27 | 1.10E-05 | -0.016 | 2.10E-01 |
| Pubgwas | Pelvic bone marrow fat | Hypertension | Inverse variance weighted | 354.24 | 28 | 3.50E-58 |  |  |
| Pubgwas | Pelvic bone marrow fat | Hypertension | MR Egger | 349.44 | 27 | 8.90E-58 | -0.001 | 5.50E-01 |
| Pubgwas | Pelvic bone marrow fat | Knee osteoarthritis | Inverse variance weighted | 144.27 | 28 | 1.30E-17 |  |  |
| Pubgwas | Pelvic bone marrow fat | Knee osteoarthritis | MR Egger | 143.01 | 27 | 9.40E-18 | -0.007 | 6.30E-01 |
| Pubgwas | Pelvic bone marrow fat | MASLD | Inverse variance weighted | 29.01 | 26 | 3.10E-01 |  |  |
| Pubgwas | Pelvic bone marrow fat | MASLD | MR Egger | 28.71 | 25 | 2.80E-01 | 0.006 | 6.10E-01 |
| Pubgwas | Pelvic bone marrow fat | Myocardial infarction | Inverse variance weighted | 102.43 | 28 | 2.00E-10 |  |  |
| Pubgwas | Pelvic bone marrow fat | Myocardial infarction | MR Egger | 102.40 | 27 | 1.00E-10 | 0.001 | 9.30E-01 |
| Pubgwas | Pelvic bone marrow fat | Osteoporosis | Inverse variance weighted | 211.45 | 28 | 4.60E-30 |  |  |
| Pubgwas | Pelvic bone marrow fat | Osteoporosis | MR Egger | 209.45 | 27 | 3.90E-30 | 0.000 | 6.20E-01 |
| Pubgwas | Pelvic bone marrow fat | Parkinson's disease | Inverse variance weighted | 20.36 | 27 | 8.20E-01 |  |  |
| Pubgwas | Pelvic bone marrow fat | Parkinson's disease | MR Egger | 20.36 | 26 | 7.70E-01 | 0.000 | 9.90E-01 |
| Pubgwas | Pelvic bone marrow fat | Peripheral artery disease | Inverse variance weighted | 39.99 | 28 | 6.60E-02 |  |  |
| Pubgwas | Pelvic bone marrow fat | Peripheral artery disease | MR Egger | 39.87 | 27 | 5.30E-02 | -0.003 | 7.90E-01 |
| Pubgwas | Pelvic bone marrow fat | Polycystic ovary syndrome | Inverse variance weighted | 44.00 | 27 | 2.10E-02 |  |  |
| Pubgwas | Pelvic bone marrow fat | Polycystic ovary syndrome | MR Egger | 39.17 | 26 | 4.70E-02 | 0.038 | 8.50E-02 |
| Pubgwas | Pelvic bone marrow fat | Psoriasis | Inverse variance weighted | 672.63 | 27 | 2.00E-124 |  |  |
| Pubgwas | Pelvic bone marrow fat | Psoriasis | MR Egger | 670.83 | 26 | 9.40E-125 | -0.015 | 7.90E-01 |
| Pubgwas | Pelvic bone marrow fat | Pulmonary embolism | Inverse variance weighted | 34.05 | 28 | 2.00E-01 |  |  |
| Pubgwas | Pelvic bone marrow fat | Pulmonary embolism | MR Egger | 30.97 | 27 | 2.70E-01 | -0.027 | 1.10E-01 |
| Pubgwas | Pelvic bone marrow fat | Rheumatoid arthritis | Inverse variance weighted | 261.50 | 27 | 3.00E-40 |  |  |
| Pubgwas | Pelvic bone marrow fat | Rheumatoid arthritis | MR Egger | 259.35 | 26 | 2.50E-40 | -0.012 | 6.50E-01 |
| Pubgwas | Pelvic bone marrow fat | Stroke | Inverse variance weighted | 72.80 | 27 | 4.40E-06 |  |  |
| Pubgwas | Pelvic bone marrow fat | Stroke | MR Egger | 66.49 | 26 | 2.10E-05 | -0.017 | 1.30E-01 |
| Pubgwas | Pelvic bone marrow fat | Type 2 diabetes | Inverse variance weighted | 120.65 | 24 | 7.40E-15 |  |  |
| Pubgwas | Pelvic bone marrow fat | Type 2 diabetes | MR Egger | 120.54 | 23 | 3.30E-15 | 0.002 | 8.80E-01 |
| Pubgwas | Thigh bone marrow fat | Alzheimer's disease | Inverse variance weighted | 51.22 | 27 | 3.30E-03 |  |  |
| Pubgwas | Thigh bone marrow fat | Alzheimer's disease | MR Egger | 50.70 | 26 | 2.60E-03 | -0.005 | 6.10E-01 |
| Pubgwas | Thigh bone marrow fat | Aortic aneurysm | Inverse variance weighted | 37.10 | 29 | 1.40E-01 |  |  |
| Pubgwas | Thigh bone marrow fat | Aortic aneurysm | MR Egger | 36.00 | 28 | 1.40E-01 | 0.018 | 3.60E-01 |
| Pubgwas | Thigh bone marrow fat | Asthma | Inverse variance weighted | 55.52 | 29 | 2.20E-03 |  |  |
| Pubgwas | Thigh bone marrow fat | Asthma | MR Egger | 53.25 | 28 | 2.70E-03 | 0.007 | 2.80E-01 |
| Pubgwas | Thigh bone marrow fat | Atrial fibrillation | Inverse variance weighted | 112.16 | 28 | 5.00E-12 |  |  |
| Pubgwas | Thigh bone marrow fat | Atrial fibrillation | MR Egger | 111.57 | 27 | 3.00E-12 | -0.004 | 7.10E-01 |
| Pubgwas | Thigh bone marrow fat | Cholelithiasis | Inverse variance weighted | 113.83 | 29 | 5.40E-12 |  |  |
| Pubgwas | Thigh bone marrow fat | Cholelithiasis | MR Egger | 106.00 | 28 | 5.30E-11 | 0.018 | 1.60E-01 |
| Pubgwas | Thigh bone marrow fat | Chronic kidney disease | Inverse variance weighted | 30.58 | 27 | 2.90E-01 |  |  |
| Pubgwas | Thigh bone marrow fat | Chronic kidney disease | MR Egger | 29.73 | 26 | 2.80E-01 | 0.007 | 4.00E-01 |
| Pubgwas | Thigh bone marrow fat | Coronary artery disease | Inverse variance weighted | 39.32 | 27 | 5.90E-02 |  |  |
| Pubgwas | Thigh bone marrow fat | Coronary artery disease | MR Egger | 38.91 | 26 | 5.00E-02 | -0.005 | 6.10E-01 |
| Pubgwas | Thigh bone marrow fat | Deep vein thrombosis | Inverse variance weighted | 47.82 | 29 | 1.50E-02 |  |  |
| Pubgwas | Thigh bone marrow fat | Deep vein thrombosis | MR Egger | 44.53 | 28 | 2.50E-02 | 0.000 | 1.60E-01 |
| Pubgwas | Thigh bone marrow fat | Depression | Inverse variance weighted | 43.64 | 29 | 4.00E-02 |  |  |
| Pubgwas | Thigh bone marrow fat | Depression | MR Egger | 43.08 | 28 | 3.40E-02 | -0.007 | 5.50E-01 |
| Pubgwas | Thigh bone marrow fat | Gastroesophageal reflux disease | Inverse variance weighted | 26.06 | 19 | 1.30E-01 |  |  |
| Pubgwas | Thigh bone marrow fat | Gastroesophageal reflux disease | MR Egger | 19.94 | 18 | 3.40E-01 | -0.013 | 3.00E-02 |
| Pubgwas | Thigh bone marrow fat | Gout | Inverse variance weighted | 23.40 | 19 | 2.20E-01 |  |  |
| Pubgwas | Thigh bone marrow fat | Gout | MR Egger | 22.48 | 18 | 2.10E-01 | 0.035 | 4.00E-01 |
| Pubgwas | Thigh bone marrow fat | Heart failure | Inverse variance weighted | 36.84 | 27 | 9.80E-02 |  |  |
| Pubgwas | Thigh bone marrow fat | Heart failure | MR Egger | 34.94 | 26 | 1.10E-01 | 0.009 | 2.50E-01 |
| Pubgwas | Thigh bone marrow fat | Hip osteoarthritis | Inverse variance weighted | 74.21 | 29 | 7.90E-06 |  |  |
| Pubgwas | Thigh bone marrow fat | Hip osteoarthritis | MR Egger | 73.18 | 28 | 6.60E-06 | 0.009 | 5.40E-01 |
| Pubgwas | Thigh bone marrow fat | Hypertension | Inverse variance weighted | 208.49 | 29 | 4.70E-29 |  |  |
| Pubgwas | Thigh bone marrow fat | Hypertension | MR Egger | 207.65 | 28 | 2.40E-29 | 0.001 | 7.40E-01 |
| Pubgwas | Thigh bone marrow fat | Knee osteoarthritis | Inverse variance weighted | 60.78 | 29 | 4.90E-04 |  |  |
| Pubgwas | Thigh bone marrow fat | Knee osteoarthritis | MR Egger | 59.61 | 28 | 4.60E-04 | 0.007 | 4.60E-01 |
| Pubgwas | Thigh bone marrow fat | MASLD | Inverse variance weighted | 40.23 | 26 | 3.70E-02 |  |  |
| Pubgwas | Thigh bone marrow fat | MASLD | MR Egger | 31.54 | 25 | 1.70E-01 | 0.038 | 1.50E-02 |
| Pubgwas | Thigh bone marrow fat | Myocardial infarction | Inverse variance weighted | 56.80 | 29 | 1.50E-03 |  |  |
| Pubgwas | Thigh bone marrow fat | Myocardial infarction | MR Egger | 55.26 | 28 | 1.60E-03 | 0.010 | 3.90E-01 |
| Pubgwas | Thigh bone marrow fat | Osteoporosis | Inverse variance weighted | 199.34 | 29 | 2.50E-27 |  |  |
| Pubgwas | Thigh bone marrow fat | Osteoporosis | MR Egger | 189.05 | 28 | 7.90E-26 | -0.001 | 2.30E-01 |
| Pubgwas | Thigh bone marrow fat | Parkinson's disease | Inverse variance weighted | 19.24 | 25 | 7.90E-01 |  |  |
| Pubgwas | Thigh bone marrow fat | Parkinson's disease | MR Egger | 17.83 | 24 | 8.10E-01 | 0.020 | 2.50E-01 |
| Pubgwas | Thigh bone marrow fat | Peripheral artery disease | Inverse variance weighted | 35.18 | 29 | 2.00E-01 |  |  |
| Pubgwas | Thigh bone marrow fat | Peripheral artery disease | MR Egger | 34.93 | 28 | 1.70E-01 | 0.006 | 6.60E-01 |
| Pubgwas | Thigh bone marrow fat | Polycystic ovary syndrome | Inverse variance weighted | 50.32 | 28 | 6.00E-03 |  |  |
| Pubgwas | Thigh bone marrow fat | Polycystic ovary syndrome | MR Egger | 50.22 | 27 | 4.30E-03 | -0.006 | 8.20E-01 |
| Pubgwas | Thigh bone marrow fat | Psoriasis | Inverse variance weighted | 64.76 | 27 | 6.10E-05 |  |  |
| Pubgwas | Thigh bone marrow fat | Psoriasis | MR Egger | 60.49 | 26 | 1.40E-04 | 0.030 | 1.90E-01 |
| Pubgwas | Thigh bone marrow fat | Pulmonary embolism | Inverse variance weighted | 38.61 | 29 | 1.10E-01 |  |  |
| Pubgwas | Thigh bone marrow fat | Pulmonary embolism | MR Egger | 38.61 | 28 | 8.70E-02 | 0.001 | 9.70E-01 |
| Pubgwas | Thigh bone marrow fat | Rheumatoid arthritis | Inverse variance weighted | 34.93 | 26 | 1.10E-01 |  |  |
| Pubgwas | Thigh bone marrow fat | Rheumatoid arthritis | MR Egger | 33.67 | 25 | 1.20E-01 | 0.014 | 3.40E-01 |
| Pubgwas | Thigh bone marrow fat | Stroke | Inverse variance weighted | 24.26 | 27 | 6.20E-01 |  |  |
| Pubgwas | Thigh bone marrow fat | Stroke | MR Egger | 24.07 | 26 | 5.70E-01 | 0.004 | 6.60E-01 |
| Pubgwas | Thigh bone marrow fat | Type 2 diabetes | Inverse variance weighted | 447.30 | 26 | 2.60E-78 |  |  |
| Pubgwas | Thigh bone marrow fat | Type 2 diabetes | MR Egger | 415.54 | 25 | 2.00E-72 | 0.030 | 1.80E-01 |
| Pubgwas | Vertebrae bone marrow fat | Alzheimer's disease | Inverse variance weighted | 1.18 | 4 | 8.80E-01 |  |  |
| Pubgwas | Vertebrae bone marrow fat | Alzheimer's disease | MR Egger | 0.21 | 3 | 9.80E-01 | 0.040 | 4.00E-01 |
| Pubgwas | Vertebrae bone marrow fat | Aortic aneurysm | Inverse variance weighted | 6.51 | 4 | 1.60E-01 |  |  |
| Pubgwas | Vertebrae bone marrow fat | Aortic aneurysm | MR Egger | 5.77 | 3 | 1.20E-01 | -0.093 | 5.80E-01 |
| Pubgwas | Vertebrae bone marrow fat | Asthma | Inverse variance weighted | 20.66 | 4 | 3.70E-04 |  |  |
| Pubgwas | Vertebrae bone marrow fat | Asthma | MR Egger | 14.65 | 3 | 2.10E-03 | -0.077 | 3.50E-01 |
| Pubgwas | Vertebrae bone marrow fat | Atrial fibrillation | Inverse variance weighted | 6.55 | 4 | 1.60E-01 |  |  |
| Pubgwas | Vertebrae bone marrow fat | Atrial fibrillation | MR Egger | 6.25 | 3 | 1.00E-01 | 0.018 | 7.30E-01 |
| Pubgwas | Vertebrae bone marrow fat | Cholelithiasis | Inverse variance weighted | 2.00 | 4 | 7.40E-01 |  |  |
| Pubgwas | Vertebrae bone marrow fat | Cholelithiasis | MR Egger | 2.00 | 3 | 5.70E-01 | -0.002 | 9.70E-01 |
| Pubgwas | Vertebrae bone marrow fat | Chronic kidney disease | Inverse variance weighted | 0.58 | 3 | 9.00E-01 |  |  |
| Pubgwas | Vertebrae bone marrow fat | Chronic kidney disease | MR Egger | 0.03 | 2 | 9.80E-01 | -0.037 | 5.40E-01 |
| Pubgwas | Vertebrae bone marrow fat | Coronary artery disease | Inverse variance weighted | 1.99 | 4 | 7.40E-01 |  |  |
| Pubgwas | Vertebrae bone marrow fat | Coronary artery disease | MR Egger | 1.97 | 3 | 5.80E-01 | -0.007 | 8.90E-01 |
| Pubgwas | Vertebrae bone marrow fat | Deep vein thrombosis | Inverse variance weighted | 6.45 | 4 | 1.70E-01 |  |  |
| Pubgwas | Vertebrae bone marrow fat | Deep vein thrombosis | MR Egger | 1.56 | 3 | 6.70E-01 | -0.003 | 1.10E-01 |
| Pubgwas | Vertebrae bone marrow fat | Depression | Inverse variance weighted | 4.84 | 4 | 3.00E-01 |  |  |
| Pubgwas | Vertebrae bone marrow fat | Depression | MR Egger | 4.14 | 3 | 2.50E-01 | 0.057 | 5.30E-01 |
| Pubgwas | Vertebrae bone marrow fat | Gastroesophageal reflux disease | Inverse variance weighted | 0.07 | 2 | 9.70E-01 |  |  |
| Pubgwas | Vertebrae bone marrow fat | Gastroesophageal reflux disease | MR Egger | 0.01 | 1 | 9.10E-01 | 0.015 | 8.50E-01 |
| Pubgwas | Vertebrae bone marrow fat | Gout | Inverse variance weighted | 1.06 | 3 | 7.90E-01 |  |  |
| Pubgwas | Vertebrae bone marrow fat | Gout | MR Egger | 0.42 | 2 | 8.10E-01 | 0.246 | 5.10E-01 |
| Pubgwas | Vertebrae bone marrow fat | Heart failure | Inverse variance weighted | 0.64 | 4 | 9.60E-01 |  |  |
| Pubgwas | Vertebrae bone marrow fat | Heart failure | MR Egger | 0.53 | 3 | 9.10E-01 | 0.013 | 7.60E-01 |
| Pubgwas | Vertebrae bone marrow fat | Hip osteoarthritis | Inverse variance weighted | 5.09 | 4 | 2.80E-01 |  |  |
| Pubgwas | Vertebrae bone marrow fat | Hip osteoarthritis | MR Egger | 4.92 | 3 | 1.80E-01 | 0.023 | 7.70E-01 |
| Pubgwas | Vertebrae bone marrow fat | Hypertension | Inverse variance weighted | 4.47 | 4 | 3.50E-01 |  |  |
| Pubgwas | Vertebrae bone marrow fat | Hypertension | MR Egger | 3.04 | 3 | 3.90E-01 | -0.005 | 3.20E-01 |
| Pubgwas | Vertebrae bone marrow fat | Knee osteoarthritis | Inverse variance weighted | 10.08 | 4 | 3.90E-02 |  |  |
| Pubgwas | Vertebrae bone marrow fat | Knee osteoarthritis | MR Egger | 0.50 | 3 | 9.20E-01 | 0.137 | 5.40E-02 |
| Pubgwas | Vertebrae bone marrow fat | MASLD | Inverse variance weighted | 1.66 | 4 | 8.00E-01 |  |  |
| Pubgwas | Vertebrae bone marrow fat | MASLD | MR Egger | 0.59 | 3 | 9.00E-01 | 0.080 | 3.80E-01 |
| Pubgwas | Vertebrae bone marrow fat | Myocardial infarction | Inverse variance weighted | 2.57 | 4 | 6.30E-01 |  |  |
| Pubgwas | Vertebrae bone marrow fat | Myocardial infarction | MR Egger | 2.48 | 3 | 4.80E-01 | -0.016 | 7.80E-01 |
| Pubgwas | Vertebrae bone marrow fat | Osteoporosis | Inverse variance weighted | 9.29 | 4 | 5.40E-02 |  |  |
| Pubgwas | Vertebrae bone marrow fat | Osteoporosis | MR Egger | 9.28 | 3 | 2.60E-02 | 0.000 | 9.50E-01 |
| Pubgwas | Vertebrae bone marrow fat | Parkinson's disease | Inverse variance weighted | 5.46 | 4 | 2.40E-01 |  |  |
| Pubgwas | Vertebrae bone marrow fat | Parkinson's disease | MR Egger | 5.15 | 3 | 1.60E-01 | 0.059 | 7.00E-01 |
| Pubgwas | Vertebrae bone marrow fat | Peripheral artery disease | Inverse variance weighted | 2.09 | 4 | 7.20E-01 |  |  |
| Pubgwas | Vertebrae bone marrow fat | Peripheral artery disease | MR Egger | 1.39 | 3 | 7.10E-01 | 0.058 | 4.70E-01 |
| Pubgwas | Vertebrae bone marrow fat | Polycystic ovary syndrome | Inverse variance weighted | 5.58 | 4 | 2.30E-01 |  |  |
| Pubgwas | Vertebrae bone marrow fat | Polycystic ovary syndrome | MR Egger | 2.80 | 3 | 4.20E-01 | 0.198 | 1.90E-01 |
| Pubgwas | Vertebrae bone marrow fat | Psoriasis | Inverse variance weighted | 3.97 | 4 | 4.10E-01 |  |  |
| Pubgwas | Vertebrae bone marrow fat | Psoriasis | MR Egger | 3.97 | 3 | 2.60E-01 | 0.002 | 9.80E-01 |
| Pubgwas | Vertebrae bone marrow fat | Pulmonary embolism | Inverse variance weighted | 5.15 | 4 | 2.70E-01 |  |  |
| Pubgwas | Vertebrae bone marrow fat | Pulmonary embolism | MR Egger | 4.35 | 3 | 2.30E-01 | -0.103 | 5.10E-01 |
| Pubgwas | Vertebrae bone marrow fat | Rheumatoid arthritis | Inverse variance weighted | 17.66 | 4 | 1.40E-03 |  |  |
| Pubgwas | Vertebrae bone marrow fat | Rheumatoid arthritis | MR Egger | 17.30 | 3 | 6.10E-04 | 0.037 | 8.20E-01 |
| Pubgwas | Vertebrae bone marrow fat | Stroke | Inverse variance weighted | 1.72 | 4 | 7.90E-01 |  |  |
| Pubgwas | Vertebrae bone marrow fat | Stroke | MR Egger | 1.50 | 3 | 6.80E-01 | 0.024 | 6.70E-01 |
| Pubgwas | Vertebrae bone marrow fat | Type 2 diabetes | Inverse variance weighted | 12.10 | 3 | 7.00E-03 |  |  |
| Pubgwas | Vertebrae bone marrow fat | Type 2 diabetes | MR Egger | 1.58 | 2 | 4.50E-01 | 0.108 | 8.30E-02 |

**Supplementary table 13. MR-PRESSO results for the effect of ten IDPs on 26 disease outcomes from FinnGen and published GWAS.** MR Analysis: the type of Mendelian randomization performed, Raw which include all the genetic variants and outlier-corrected after removing variants identified as outliers; Causal Est: the effect size before and after correcting for the identified outliers; Sd: the standard deviation of the effect size; T_stat: the statistic al significance of the effect size; n_Outliers: the number of genetic variants identified as outliers; BHP: Benjamini-Hochberg p-value.

| **Source** | **Exposure** | **Outcome** | **MR_Analysis** | **Causal_Est** | **Sd** | **T_stat** | **P_value** | **Number_of_Outliers** | **BHP** |
| --- | --- | --- | --- | --- | --- | --- | --- | --- | --- |
| FinnGen | Thigh subcutaneous AT | Type 2 diabetes | Raw | -0.461 | 0.19 | -2.37 | 2.8E-02 | 7 | 2.1E-01 |
| FinnGen | Thigh subcutaneous AT | Type 2 diabetes | Outlier-corrected | -0.484 | 0.07 | -7.02 | 9.0E-06 | 7 | 2.4E-03 |
| FinnGen | Thigh subcutaneous AT | Polycystic ovary syndrome | Raw | -0.392 | 0.19 | -2.07 | 5.1E-02 | NA | NA |
| FinnGen | Thigh subcutaneous AT | Polycystic ovary syndrome | Outlier-corrected | NA | NA | NA | NA | NA | NA |
| FinnGen | Thigh subcutaneous AT | MASLD | Raw | -0.500 | 0.21 | -2.34 | 3.0E-02 | 1 | 2.2E-01 |
| FinnGen | Thigh subcutaneous AT | MASLD | Outlier-corrected | -0.611 | 0.17 | -3.55 | 2.2E-03 | 1 | 5.8E-02 |
| FinnGen | Thigh subcutaneous AT | Chronic kidney disease | Raw | -0.093 | 0.10 | -0.98 | 3.4E-01 | NA | NA |
| FinnGen | Thigh subcutaneous AT | Chronic kidney disease | Outlier-corrected | NA | NA | NA | NA | NA | NA |
| FinnGen | Thigh subcutaneous AT | Hypertension | Raw | -0.215 | 0.11 | -2.02 | 5.7E-02 | 6 | 3.1E-01 |
| FinnGen | Thigh subcutaneous AT | Hypertension | Outlier-corrected | -0.199 | 0.04 | -4.75 | 3.1E-04 | 6 | 2.8E-02 |
| FinnGen | Thigh subcutaneous AT | Coronary artery disease | Raw | -0.232 | 0.09 | -2.47 | 2.3E-02 | 1 | 2.0E-01 |
| FinnGen | Thigh subcutaneous AT | Coronary artery disease | Outlier-corrected | -0.269 | 0.09 | -3.15 | 5.3E-03 | 1 | 8.8E-02 |
| FinnGen | Thigh subcutaneous AT | Stroke | Raw | -0.068 | 0.09 | -0.74 | 4.7E-01 | 1 | 8.3E-01 |
| FinnGen | Thigh subcutaneous AT | Stroke | Outlier-corrected | NA | NA | NA | NA | 1 | NA |
| FinnGen | Thigh subcutaneous AT | Myocardial infarction | Raw | -0.233 | 0.09 | -2.56 | 1.9E-02 | 2 | 1.9E-01 |
| FinnGen | Thigh subcutaneous AT | Myocardial infarction | Outlier-corrected | -0.157 | 0.07 | -2.14 | 4.6E-02 | 2 | 2.8E-01 |
| FinnGen | Thigh subcutaneous AT | Aortic aneurysm | Raw | -0.255 | 0.11 | -2.25 | 3.6E-02 | 1 | 2.4E-01 |
| FinnGen | Thigh subcutaneous AT | Aortic aneurysm | Outlier-corrected | NA | NA | NA | NA | 1 | NA |
| FinnGen | Thigh subcutaneous AT | Heart failure | Raw | 0.076 | 0.07 | 1.05 | 3.1E-01 | 1 | 7.2E-01 |
| FinnGen | Thigh subcutaneous AT | Heart failure | Outlier-corrected | 0.042 | 0.06 | 0.68 | 5.0E-01 | 1 | 8.6E-01 |
| FinnGen | Thigh subcutaneous AT | Atrial fibrillation | Raw | 0.046 | 0.12 | 0.39 | 7.0E-01 | 3 | 9.2E-01 |
| FinnGen | Thigh subcutaneous AT | Atrial fibrillation | Outlier-corrected | -0.042 | 0.07 | -0.60 | 5.6E-01 | 3 | 8.6E-01 |
| FinnGen | Thigh subcutaneous AT | Peripheral artery disease | Raw | -0.258 | 0.10 | -2.54 | 1.9E-02 | 1 | 1.9E-01 |
| FinnGen | Thigh subcutaneous AT | Peripheral artery disease | Outlier-corrected | NA | NA | NA | NA | 1 | NA |
| FinnGen | Thigh subcutaneous AT | Deep vein thrombosis | Raw | 0.310 | 0.14 | 2.21 | 3.9E-02 | 2 | 2.5E-01 |
| FinnGen | Thigh subcutaneous AT | Deep vein thrombosis | Outlier-corrected | 0.273 | 0.10 | 2.60 | 1.8E-02 | 2 | 1.9E-01 |
| FinnGen | Thigh subcutaneous AT | Pulmonary embolism | Raw | 0.231 | 0.11 | 2.19 | 4.1E-02 | 1 | 2.6E-01 |
| FinnGen | Thigh subcutaneous AT | Pulmonary embolism | Outlier-corrected | 0.289 | 0.08 | 3.45 | 2.7E-03 | 1 | 6.6E-02 |
| FinnGen | Thigh subcutaneous AT | Gout | Raw | -0.250 | 0.16 | -1.57 | 1.3E-01 | 1 | 5.0E-01 |
| FinnGen | Thigh subcutaneous AT | Gout | Outlier-corrected | -0.334 | 0.14 | -2.45 | 2.4E-02 | 1 | 2.0E-01 |
| FinnGen | Thigh subcutaneous AT | Knee osteoarthritis | Raw | 0.248 | 0.11 | 2.32 | 3.1E-02 | 3 | 2.2E-01 |
| FinnGen | Thigh subcutaneous AT | Knee osteoarthritis | Outlier-corrected | 0.196 | 0.06 | 3.11 | 6.4E-03 | 3 | 9.7E-02 |
| FinnGen | Thigh subcutaneous AT | Hip osteoarthritis | Raw | -0.053 | 0.11 | -0.46 | 6.5E-01 | 3 | 8.9E-01 |
| FinnGen | Thigh subcutaneous AT | Hip osteoarthritis | Outlier-corrected | 0.004 | 0.08 | 0.06 | 9.6E-01 | 3 | 9.7E-01 |
| FinnGen | Thigh subcutaneous AT | Rheumatoid arthritis | Raw | 0.127 | 0.09 | 1.38 | 1.8E-01 | 1 | 5.9E-01 |
| FinnGen | Thigh subcutaneous AT | Rheumatoid arthritis | Outlier-corrected | 0.189 | 0.09 | 2.21 | 4.0E-02 | 1 | 2.5E-01 |
| FinnGen | Thigh subcutaneous AT | Osteoporosis | Raw | 0.257 | 0.11 | 2.45 | 2.4E-02 | NA | NA |
| FinnGen | Thigh subcutaneous AT | Osteoporosis | Outlier-corrected | NA | NA | NA | NA | NA | NA |
| FinnGen | Thigh subcutaneous AT | Asthma | Raw | 0.171 | 0.06 | 2.70 | 1.4E-02 | 1 | 1.8E-01 |
| FinnGen | Thigh subcutaneous AT | Asthma | Outlier-corrected | 0.206 | 0.06 | 3.54 | 2.2E-03 | 1 | 5.8E-02 |
| FinnGen | Thigh subcutaneous AT | Psoriasis | Raw | -0.126 | 0.09 | -1.46 | 1.6E-01 | NA | NA |
| FinnGen | Thigh subcutaneous AT | Psoriasis | Outlier-corrected | NA | NA | NA | NA | NA | NA |
| FinnGen | Thigh subcutaneous AT | Depression | Raw | 0.085 | 0.04 | 2.02 | 5.7E-02 | NA | NA |
| FinnGen | Thigh subcutaneous AT | Depression | Outlier-corrected | NA | NA | NA | NA | NA | NA |
| FinnGen | Thigh subcutaneous AT | Parkinson's disease | Raw | -0.095 | 0.15 | -0.64 | 5.3E-01 | 1 | 8.6E-01 |
| FinnGen | Thigh subcutaneous AT | Parkinson's disease | Outlier-corrected | NA | NA | NA | NA | 1 | NA |
| FinnGen | Thigh subcutaneous AT | Alzheimer’s disease | Raw | -0.094 | 0.08 | -1.18 | 2.5E-01 | NA | NA |
| FinnGen | Thigh subcutaneous AT | Alzheimer’s disease | Outlier-corrected | NA | NA | NA | NA | NA | NA |
| FinnGen | Thigh subcutaneous AT | Cholelithiasis | Raw | 0.018 | 0.08 | 0.24 | 8.1E-01 | 2 | 9.5E-01 |
| FinnGen | Thigh subcutaneous AT | Cholelithiasis | Outlier-corrected | 0.007 | 0.06 | 0.13 | 9.0E-01 | 2 | 9.6E-01 |
| FinnGen | Thigh subcutaneous AT | Gastroesophageal reflux disease | Raw | 0.025 | 0.07 | 0.37 | 7.1E-01 | 1 | 9.2E-01 |
| FinnGen | Thigh subcutaneous AT | Gastroesophageal reflux disease | Outlier-corrected | -0.006 | 0.06 | -0.10 | 9.2E-01 | 1 | 9.7E-01 |
| FinnGen | Thigh intermuscular AT | Type 2 diabetes | Raw | -0.592 | 0.13 | -4.52 | 7.0E-04 | 3 | 3.1E-02 |
| FinnGen | Thigh intermuscular AT | Type 2 diabetes | Outlier-corrected | -0.444 | 0.06 | -7.10 | 5.7E-05 | 3 | 7.7E-03 |
| FinnGen | Thigh intermuscular AT | Polycystic ovary syndrome | Raw | -0.496 | 0.22 | -2.25 | 4.4E-02 | NA | NA |
| FinnGen | Thigh intermuscular AT | Polycystic ovary syndrome | Outlier-corrected | NA | NA | NA | NA | NA | NA |
| FinnGen | Thigh intermuscular AT | MASLD | Raw | -0.752 | 0.18 | -4.08 | 1.5E-03 | NA | NA |
| FinnGen | Thigh intermuscular AT | MASLD | Outlier-corrected | NA | NA | NA | NA | NA | NA |
| FinnGen | Thigh intermuscular AT | Chronic kidney disease | Raw | -0.155 | 0.12 | -1.34 | 2.0E-01 | NA | NA |
| FinnGen | Thigh intermuscular AT | Chronic kidney disease | Outlier-corrected | NA | NA | NA | NA | NA | NA |
| FinnGen | Thigh intermuscular AT | Hypertension | Raw | -0.272 | 0.06 | -4.74 | 4.8E-04 | 1 | 2.9E-02 |
| FinnGen | Thigh intermuscular AT | Hypertension | Outlier-corrected | -0.237 | 0.05 | -4.83 | 5.3E-04 | 1 | 2.9E-02 |
| FinnGen | Thigh intermuscular AT | Coronary artery disease | Raw | -0.262 | 0.07 | -3.56 | 3.9E-03 | NA | NA |
| FinnGen | Thigh intermuscular AT | Coronary artery disease | Outlier-corrected | NA | NA | NA | NA | NA | NA |
| FinnGen | Thigh intermuscular AT | Stroke | Raw | -0.212 | 0.12 | -1.75 | 1.0E-01 | 1 | 4.2E-01 |
| FinnGen | Thigh intermuscular AT | Stroke | Outlier-corrected | -0.140 | 0.10 | -1.36 | 2.0E-01 | 1 | 6.3E-01 |
| FinnGen | Thigh intermuscular AT | Myocardial infarction | Raw | -0.294 | 0.07 | -4.08 | 1.5E-03 | NA | NA |
| FinnGen | Thigh intermuscular AT | Myocardial infarction | Outlier-corrected | NA | NA | NA | NA | NA | NA |
| FinnGen | Thigh intermuscular AT | Aortic aneurysm | Raw | -0.066 | 0.11 | -0.62 | 5.4E-01 | NA | NA |
| FinnGen | Thigh intermuscular AT | Aortic aneurysm | Outlier-corrected | NA | NA | NA | NA | NA | NA |
| FinnGen | Thigh intermuscular AT | Heart failure | Raw | -0.001 | 0.06 | -0.02 | 9.8E-01 | NA | NA |
| FinnGen | Thigh intermuscular AT | Heart failure | Outlier-corrected | NA | NA | NA | NA | NA | NA |
| FinnGen | Thigh intermuscular AT | Atrial fibrillation | Raw | -0.052 | 0.08 | -0.65 | 5.3E-01 | 1 | 8.6E-01 |
| FinnGen | Thigh intermuscular AT | Atrial fibrillation | Outlier-corrected | NA | NA | NA | NA | 1 | NA |
| FinnGen | Thigh intermuscular AT | Peripheral artery disease | Raw | -0.170 | 0.11 | -1.61 | 1.3E-01 | NA | NA |
| FinnGen | Thigh intermuscular AT | Peripheral artery disease | Outlier-corrected | NA | NA | NA | NA | NA | NA |
| FinnGen | Thigh intermuscular AT | Deep vein thrombosis | Raw | 0.228 | 0.10 | 2.35 | 3.7E-02 | NA | NA |
| FinnGen | Thigh intermuscular AT | Deep vein thrombosis | Outlier-corrected | NA | NA | NA | NA | NA | NA |
| FinnGen | Thigh intermuscular AT | Pulmonary embolism | Raw | 0.173 | 0.11 | 1.56 | 1.5E-01 | NA | NA |
| FinnGen | Thigh intermuscular AT | Pulmonary embolism | Outlier-corrected | NA | NA | NA | NA | NA | NA |
| FinnGen | Thigh intermuscular AT | Gout | Raw | -0.206 | 0.19 | -1.07 | 3.0E-01 | 1 | 7.2E-01 |
| FinnGen | Thigh intermuscular AT | Gout | Outlier-corrected | -0.317 | 0.18 | -1.81 | 9.8E-02 | 1 | 4.2E-01 |
| FinnGen | Thigh intermuscular AT | Knee osteoarthritis | Raw | 0.044 | 0.09 | 0.51 | 6.2E-01 | 1 | 8.9E-01 |
| FinnGen | Thigh intermuscular AT | Knee osteoarthritis | Outlier-corrected | 0.115 | 0.05 | 2.09 | 6.0E-02 | 1 | 3.1E-01 |
| FinnGen | Thigh intermuscular AT | Hip osteoarthritis | Raw | -0.013 | 0.11 | -0.11 | 9.1E-01 | 1 | 9.6E-01 |
| FinnGen | Thigh intermuscular AT | Hip osteoarthritis | Outlier-corrected | NA | NA | NA | NA | 1 | NA |
| FinnGen | Thigh intermuscular AT | Rheumatoid arthritis | Raw | 0.047 | 0.10 | 0.48 | 6.4E-01 | NA | NA |
| FinnGen | Thigh intermuscular AT | Rheumatoid arthritis | Outlier-corrected | NA | NA | NA | NA | NA | NA |
| FinnGen | Thigh intermuscular AT | Osteoporosis | Raw | 0.016 | 0.11 | 0.15 | 8.9E-01 | NA | NA |
| FinnGen | Thigh intermuscular AT | Osteoporosis | Outlier-corrected | NA | NA | NA | NA | NA | NA |
| FinnGen | Thigh intermuscular AT | Asthma | Raw | 0.048 | 0.10 | 0.47 | 6.5E-01 | 4 | 8.9E-01 |
| FinnGen | Thigh intermuscular AT | Asthma | Outlier-corrected | 0.020 | 0.08 | 0.26 | 8.0E-01 | 4 | 9.5E-01 |
| FinnGen | Thigh intermuscular AT | Psoriasis | Raw | -0.034 | 0.08 | -0.40 | 6.9E-01 | NA | NA |
| FinnGen | Thigh intermuscular AT | Psoriasis | Outlier-corrected | NA | NA | NA | NA | NA | NA |
| FinnGen | Thigh intermuscular AT | Depression | Raw | -0.006 | 0.04 | -0.16 | 8.8E-01 | NA | NA |
| FinnGen | Thigh intermuscular AT | Depression | Outlier-corrected | NA | NA | NA | NA | NA | NA |
| FinnGen | Thigh intermuscular AT | Parkinson's disease | Raw | -0.124 | 0.14 | -0.91 | 3.8E-01 | NA | NA |
| FinnGen | Thigh intermuscular AT | Parkinson's disease | Outlier-corrected | NA | NA | NA | NA | NA | NA |
| FinnGen | Thigh intermuscular AT | Alzheimer’s disease | Raw | -0.089 | 0.12 | -0.74 | 4.7E-01 | NA | NA |
| FinnGen | Thigh intermuscular AT | Alzheimer’s disease | Outlier-corrected | NA | NA | NA | NA | NA | NA |
| FinnGen | Thigh intermuscular AT | Cholelithiasis | Raw | 0.016 | 0.08 | 0.20 | 8.5E-01 | 1 | 9.6E-01 |
| FinnGen | Thigh intermuscular AT | Cholelithiasis | Outlier-corrected | 0.075 | 0.08 | 0.94 | 3.7E-01 | 1 | 7.5E-01 |
| FinnGen | Thigh intermuscular AT | Gastroesophageal reflux disease | Raw | -0.085 | 0.06 | -1.55 | 1.5E-01 | NA | NA |
| FinnGen | Thigh intermuscular AT | Gastroesophageal reflux disease | Outlier-corrected | NA | NA | NA | NA | NA | NA |
| FinnGen | Visceral AT | Type 2 diabetes | Raw | 0.330 | 0.55 | 0.60 | 5.8E-01 | 4 | 8.6E-01 |
| FinnGen | Visceral AT | Type 2 diabetes | Outlier-corrected | 0.358 | 0.01 | 26.78 | 2.4E-02 | 4 | 2.0E-01 |
| FinnGen | Visceral AT | Polycystic ovary syndrome | Raw | 0.441 | 0.56 | 0.79 | 4.6E-01 | NA | NA |
| FinnGen | Visceral AT | Polycystic ovary syndrome | Outlier-corrected | NA | NA | NA | NA | NA | NA |
| FinnGen | Visceral AT | MASLD | Raw | 0.085 | 0.57 | 0.15 | 8.9E-01 | 1 | 9.6E-01 |
| FinnGen | Visceral AT | MASLD | Outlier-corrected | -0.345 | 0.40 | -0.87 | 4.3E-01 | 1 | 8.0E-01 |
| FinnGen | Visceral AT | Chronic kidney disease | Raw | 0.050 | 0.21 | 0.24 | 8.2E-01 | NA | NA |
| FinnGen | Visceral AT | Chronic kidney disease | Outlier-corrected | NA | NA | NA | NA | NA | NA |
| FinnGen | Visceral AT | Hypertension | Raw | 0.286 | 0.31 | 0.92 | 4.0E-01 | 4 | 7.7E-01 |
| FinnGen | Visceral AT | Hypertension | Outlier-corrected | 0.040 | 0.03 | 1.39 | 4.0E-01 | 4 | 7.7E-01 |
| FinnGen | Visceral AT | Coronary artery disease | Raw | 0.094 | 0.22 | 0.43 | 6.8E-01 | 1 | 9.1E-01 |
| FinnGen | Visceral AT | Coronary artery disease | Outlier-corrected | NA | NA | NA | NA | 1 | NA |
| FinnGen | Visceral AT | Stroke | Raw | 0.049 | 0.35 | 0.14 | 8.9E-01 | 1 | 9.6E-01 |
| FinnGen | Visceral AT | Stroke | Outlier-corrected | 0.364 | 0.20 | 1.78 | 1.5E-01 | 1 | 5.4E-01 |
| FinnGen | Visceral AT | Myocardial infarction | Raw | -0.245 | 0.17 | -1.41 | 2.2E-01 | 1 | 6.6E-01 |
| FinnGen | Visceral AT | Myocardial infarction | Outlier-corrected | -0.377 | 0.12 | -3.14 | 3.5E-02 | 1 | 2.4E-01 |
| FinnGen | Visceral AT | Aortic aneurysm | Raw | -0.024 | 0.20 | -0.12 | 9.1E-01 | NA | NA |
| FinnGen | Visceral AT | Aortic aneurysm | Outlier-corrected | NA | NA | NA | NA | NA | NA |
| FinnGen | Visceral AT | Heart failure | Raw | 0.161 | 0.14 | 1.12 | 3.2E-01 | NA | NA |
| FinnGen | Visceral AT | Heart failure | Outlier-corrected | NA | NA | NA | NA | NA | NA |
| FinnGen | Visceral AT | Atrial fibrillation | Raw | 0.170 | 0.30 | 0.57 | 5.9E-01 | 2 | 8.7E-01 |
| FinnGen | Visceral AT | Atrial fibrillation | Outlier-corrected | 0.048 | 0.12 | 0.40 | 7.2E-01 | 2 | 9.2E-01 |
| FinnGen | Visceral AT | Peripheral artery disease | Raw | 0.154 | 0.24 | 0.65 | 5.4E-01 | 1 | 8.6E-01 |
| FinnGen | Visceral AT | Peripheral artery disease | Outlier-corrected | NA | NA | NA | NA | 1 | NA |
| FinnGen | Visceral AT | Deep vein thrombosis | Raw | 0.344 | 0.29 | 1.18 | 2.9E-01 | NA | NA |
| FinnGen | Visceral AT | Deep vein thrombosis | Outlier-corrected | NA | NA | NA | NA | NA | NA |
| FinnGen | Visceral AT | Pulmonary embolism | Raw | 0.210 | 0.24 | 0.87 | 4.2E-01 | NA | NA |
| FinnGen | Visceral AT | Pulmonary embolism | Outlier-corrected | NA | NA | NA | NA | NA | NA |
| FinnGen | Visceral AT | Gout | Raw | 0.205 | 0.27 | 0.77 | 4.8E-01 | NA | NA |
| FinnGen | Visceral AT | Gout | Outlier-corrected | NA | NA | NA | NA | NA | NA |
| FinnGen | Visceral AT | Knee osteoarthritis | Raw | 0.383 | 0.35 | 1.11 | 3.2E-01 | 4 | 7.3E-01 |
| FinnGen | Visceral AT | Knee osteoarthritis | Outlier-corrected | 0.164 | 0.07 | 2.51 | 2.4E-01 | 4 | 6.8E-01 |
| FinnGen | Visceral AT | Hip osteoarthritis | Raw | 0.291 | 0.31 | 0.94 | 3.9E-01 | 2 | 7.7E-01 |
| FinnGen | Visceral AT | Hip osteoarthritis | Outlier-corrected | 0.236 | 0.16 | 1.50 | 2.3E-01 | 2 | 6.7E-01 |
| FinnGen | Visceral AT | Rheumatoid arthritis | Raw | -0.144 | 0.30 | -0.49 | 6.5E-01 | 2 | 8.9E-01 |
| FinnGen | Visceral AT | Rheumatoid arthritis | Outlier-corrected | -0.064 | 0.23 | -0.28 | 8.0E-01 | 2 | 9.5E-01 |
| FinnGen | Visceral AT | Osteoporosis | Raw | -0.175 | 0.21 | -0.83 | 4.4E-01 | NA | NA |
| FinnGen | Visceral AT | Osteoporosis | Outlier-corrected | NA | NA | NA | NA | NA | NA |
| FinnGen | Visceral AT | Asthma | Raw | 0.412 | 0.07 | 5.71 | 2.3E-03 | NA | NA |
| FinnGen | Visceral AT | Asthma | Outlier-corrected | NA | NA | NA | NA | NA | NA |
| FinnGen | Visceral AT | Psoriasis | Raw | 0.120 | 0.13 | 0.90 | 4.1E-01 | NA | NA |
| FinnGen | Visceral AT | Psoriasis | Outlier-corrected | NA | NA | NA | NA | NA | NA |
| FinnGen | Visceral AT | Depression | Raw | 0.084 | 0.14 | 0.59 | 5.8E-01 | 1 | 8.6E-01 |
| FinnGen | Visceral AT | Depression | Outlier-corrected | 0.166 | 0.13 | 1.24 | 2.8E-01 | 1 | 7.2E-01 |
| FinnGen | Visceral AT | Parkinson's disease | Raw | -0.105 | 0.13 | -0.82 | 4.5E-01 | NA | NA |
| FinnGen | Visceral AT | Parkinson's disease | Outlier-corrected | NA | NA | NA | NA | NA | NA |
| FinnGen | Visceral AT | Alzheimer’s disease | Raw | -0.285 | 0.18 | -1.55 | 1.8E-01 | NA | NA |
| FinnGen | Visceral AT | Alzheimer’s disease | Outlier-corrected | NA | NA | NA | NA | NA | NA |
| FinnGen | Visceral AT | Cholelithiasis | Raw | 0.414 | 0.17 | 2.51 | 5.4E-02 | 2 | 3.0E-01 |
| FinnGen | Visceral AT | Cholelithiasis | Outlier-corrected | 0.371 | 0.07 | 5.07 | 1.5E-02 | 2 | 1.8E-01 |
| FinnGen | Visceral AT | Gastroesophageal reflux disease | Raw | -0.020 | 0.16 | -0.12 | 9.1E-01 | 1 | 9.6E-01 |
| FinnGen | Visceral AT | Gastroesophageal reflux disease | Outlier-corrected | 0.106 | 0.13 | 0.83 | 4.5E-01 | 1 | 8.2E-01 |
| FinnGen | Liver PDFF | Type 2 diabetes | Raw | 0.179 | 0.10 | 1.71 | 1.3E-01 | 5 | 4.9E-01 |
| FinnGen | Liver PDFF | Type 2 diabetes | Outlier-corrected | 0.222 | 0.04 | 6.17 | 8.6E-03 | 5 | 1.2E-01 |
| FinnGen | Liver PDFF | Polycystic ovary syndrome | Raw | -0.002 | 0.16 | -0.01 | 9.9E-01 | NA | NA |
| FinnGen | Liver PDFF | Polycystic ovary syndrome | Outlier-corrected | NA | NA | NA | NA | NA | NA |
| FinnGen | Liver PDFF | MASLD | Raw | 1.765 | 0.14 | 12.33 | 1.8E-06 | NA | NA |
| FinnGen | Liver PDFF | MASLD | Outlier-corrected | NA | NA | NA | NA | NA | NA |
| FinnGen | Liver PDFF | Chronic kidney disease | Raw | -0.001 | 0.09 | -0.01 | 9.9E-01 | 2 | 9.9E-01 |
| FinnGen | Liver PDFF | Chronic kidney disease | Outlier-corrected | -0.008 | 0.08 | -0.10 | 9.2E-01 | 2 | 9.7E-01 |
| FinnGen | Liver PDFF | Hypertension | Raw | 0.046 | 0.05 | 0.87 | 4.1E-01 | 5 | 7.8E-01 |
| FinnGen | Liver PDFF | Hypertension | Outlier-corrected | 0.134 | 0.01 | 11.46 | 1.4E-03 | 5 | 4.8E-02 |
| FinnGen | Liver PDFF | Coronary artery disease | Raw | -0.094 | 0.10 | -0.94 | 3.8E-01 | 3 | 7.6E-01 |
| FinnGen | Liver PDFF | Coronary artery disease | Outlier-corrected | -0.087 | 0.08 | -1.14 | 3.1E-01 | 3 | 7.2E-01 |
| FinnGen | Liver PDFF | Stroke | Raw | -0.049 | 0.05 | -1.03 | 3.3E-01 | NA | NA |
| FinnGen | Liver PDFF | Stroke | Outlier-corrected | NA | NA | NA | NA | NA | NA |
| FinnGen | Liver PDFF | Myocardial infarction | Raw | -0.243 | 0.09 | -2.84 | 2.2E-02 | 2 | 2.0E-01 |
| FinnGen | Liver PDFF | Myocardial infarction | Outlier-corrected | -0.245 | 0.06 | -4.08 | 6.5E-03 | 2 | 9.7E-02 |
| FinnGen | Liver PDFF | Aortic aneurysm | Raw | -0.149 | 0.10 | -1.50 | 1.7E-01 | 1 | 5.8E-01 |
| FinnGen | Liver PDFF | Aortic aneurysm | Outlier-corrected | -0.188 | 0.07 | -2.83 | 2.6E-02 | 1 | 2.1E-01 |
| FinnGen | Liver PDFF | Heart failure | Raw | -0.010 | 0.06 | -0.17 | 8.7E-01 | 2 | 9.6E-01 |
| FinnGen | Liver PDFF | Heart failure | Outlier-corrected | -0.005 | 0.03 | -0.14 | 8.9E-01 | 2 | 9.6E-01 |
| FinnGen | Liver PDFF | Atrial fibrillation | Raw | -0.064 | 0.04 | -1.70 | 1.3E-01 | NA | NA |
| FinnGen | Liver PDFF | Atrial fibrillation | Outlier-corrected | NA | NA | NA | NA | NA | NA |
| FinnGen | Liver PDFF | Peripheral artery disease | Raw | -0.059 | 0.10 | -0.61 | 5.6E-01 | 2 | 8.6E-01 |
| FinnGen | Liver PDFF | Peripheral artery disease | Outlier-corrected | -0.138 | 0.06 | -2.31 | 6.0E-02 | 2 | 3.1E-01 |
| FinnGen | Liver PDFF | Deep vein thrombosis | Raw | -0.124 | 0.06 | -2.17 | 6.2E-02 | NA | NA |
| FinnGen | Liver PDFF | Deep vein thrombosis | Outlier-corrected | NA | NA | NA | NA | NA | NA |
| FinnGen | Liver PDFF | Pulmonary embolism | Raw | -0.092 | 0.09 | -0.99 | 3.5E-01 | 2 | 7.4E-01 |
| FinnGen | Liver PDFF | Pulmonary embolism | Outlier-corrected | -0.037 | 0.07 | -0.51 | 6.3E-01 | 2 | 8.9E-01 |
| FinnGen | Liver PDFF | Gout | Raw | -0.085 | 0.24 | -0.36 | 7.3E-01 | 3 | 9.3E-01 |
| FinnGen | Liver PDFF | Gout | Outlier-corrected | -0.035 | 0.15 | -0.24 | 8.2E-01 | 3 | 9.5E-01 |
| FinnGen | Liver PDFF | Knee osteoarthritis | Raw | 0.057 | 0.06 | 0.97 | 3.6E-01 | 2 | 7.4E-01 |
| FinnGen | Liver PDFF | Knee osteoarthritis | Outlier-corrected | 0.055 | 0.04 | 1.52 | 1.8E-01 | 2 | 5.9E-01 |
| FinnGen | Liver PDFF | Hip osteoarthritis | Raw | 0.016 | 0.06 | 0.27 | 8.0E-01 | 2 | 9.5E-01 |
| FinnGen | Liver PDFF | Hip osteoarthritis | Outlier-corrected | 0.018 | 0.02 | 0.89 | 4.1E-01 | 2 | 7.8E-01 |
| FinnGen | Liver PDFF | Rheumatoid arthritis | Raw | 0.011 | 0.05 | 0.23 | 8.2E-01 | NA | NA |
| FinnGen | Liver PDFF | Rheumatoid arthritis | Outlier-corrected | NA | NA | NA | NA | NA | NA |
| FinnGen | Liver PDFF | Osteoporosis | Raw | 0.057 | 0.09 | 0.63 | 5.5E-01 | 1 | 8.6E-01 |
| FinnGen | Liver PDFF | Osteoporosis | Outlier-corrected | 0.046 | 0.06 | 0.73 | 4.9E-01 | 1 | 8.5E-01 |
| FinnGen | Liver PDFF | Asthma | Raw | 0.008 | 0.07 | 0.12 | 9.1E-01 | 3 | 9.6E-01 |
| FinnGen | Liver PDFF | Asthma | Outlier-corrected | -0.001 | 0.05 | -0.03 | 9.8E-01 | 3 | 9.9E-01 |
| FinnGen | Liver PDFF | Psoriasis | Raw | 0.180 | 0.08 | 2.25 | 5.5E-02 | 1 | 3.0E-01 |
| FinnGen | Liver PDFF | Psoriasis | Outlier-corrected | 0.154 | 0.06 | 2.40 | 4.7E-02 | 1 | 2.8E-01 |
| FinnGen | Liver PDFF | Depression | Raw | -0.027 | 0.03 | -0.79 | 4.5E-01 | NA | NA |
| FinnGen | Liver PDFF | Depression | Outlier-corrected | NA | NA | NA | NA | NA | NA |
| FinnGen | Liver PDFF | Parkinson's disease | Raw | -0.129 | 0.07 | -1.81 | 1.1E-01 | NA | NA |
| FinnGen | Liver PDFF | Parkinson's disease | Outlier-corrected | NA | NA | NA | NA | NA | NA |
| FinnGen | Liver PDFF | Alzheimer’s disease | Raw | -0.997 | 0.98 | -1.01 | 3.4E-01 | 7 | 7.4E-01 |
| FinnGen | Liver PDFF | Alzheimer’s disease | Outlier-corrected | 0.202 | 0.24 | 0.85 | 5.5E-01 | 7 | 8.6E-01 |
| FinnGen | Liver PDFF | Cholelithiasis | Raw | -0.150 | 0.11 | -1.31 | 2.3E-01 | 4 | 6.7E-01 |
| FinnGen | Liver PDFF | Cholelithiasis | Outlier-corrected | -0.030 | 0.03 | -1.07 | 3.5E-01 | 4 | 7.4E-01 |
| FinnGen | Liver PDFF | Gastroesophageal reflux disease | Raw | -0.006 | 0.04 | -0.16 | 8.8E-01 | NA | NA |
| FinnGen | Liver PDFF | Gastroesophageal reflux disease | Outlier-corrected | NA | NA | NA | NA | NA | NA |
| FinnGen | Pancreas PDFF | Type 2 diabetes | Raw | 0.026 | 0.14 | 0.19 | 8.6E-01 | 7 | 9.6E-01 |
| FinnGen | Pancreas PDFF | Type 2 diabetes | Outlier-corrected | -0.091 | 0.10 | -0.93 | 3.9E-01 | 7 | 7.7E-01 |
| FinnGen | Pancreas PDFF | Polycystic ovary syndrome | Raw | 0.132 | 0.22 | 0.61 | 5.5E-01 | NA | NA |
| FinnGen | Pancreas PDFF | Polycystic ovary syndrome | Outlier-corrected | NA | NA | NA | NA | NA | NA |
| FinnGen | Pancreas PDFF | MASLD | Raw | 0.075 | 0.18 | 0.41 | 6.9E-01 | NA | NA |
| FinnGen | Pancreas PDFF | MASLD | Outlier-corrected | NA | NA | NA | NA | NA | NA |
| FinnGen | Pancreas PDFF | Chronic kidney disease | Raw | -0.123 | 0.06 | -1.93 | 7.8E-02 | NA | NA |
| FinnGen | Pancreas PDFF | Chronic kidney disease | Outlier-corrected | NA | NA | NA | NA | NA | NA |
| FinnGen | Pancreas PDFF | Hypertension | Raw | -0.010 | 0.06 | -0.17 | 8.7E-01 | 3 | 9.6E-01 |
| FinnGen | Pancreas PDFF | Hypertension | Outlier-corrected | 0.026 | 0.04 | 0.67 | 5.2E-01 | 3 | 8.6E-01 |
| FinnGen | Pancreas PDFF | Coronary artery disease | Raw | -0.051 | 0.11 | -0.46 | 6.5E-01 | 1 | 8.9E-01 |
| FinnGen | Pancreas PDFF | Coronary artery disease | Outlier-corrected | -0.149 | 0.07 | -2.01 | 7.0E-02 | 1 | 3.5E-01 |
| FinnGen | Pancreas PDFF | Stroke | Raw | 0.136 | 0.11 | 1.21 | 2.5E-01 | 1 | 6.8E-01 |
| FinnGen | Pancreas PDFF | Stroke | Outlier-corrected | 0.059 | 0.08 | 0.77 | 4.6E-01 | 1 | 8.2E-01 |
| FinnGen | Pancreas PDFF | Myocardial infarction | Raw | -0.008 | 0.11 | -0.07 | 9.4E-01 | 1 | 9.7E-01 |
| FinnGen | Pancreas PDFF | Myocardial infarction | Outlier-corrected | -0.101 | 0.07 | -1.35 | 2.0E-01 | 1 | 6.3E-01 |
| FinnGen | Pancreas PDFF | Aortic aneurysm | Raw | 0.021 | 0.09 | 0.24 | 8.1E-01 | NA | NA |
| FinnGen | Pancreas PDFF | Aortic aneurysm | Outlier-corrected | NA | NA | NA | NA | NA | NA |
| FinnGen | Pancreas PDFF | Heart failure | Raw | 0.088 | 0.08 | 1.03 | 3.2E-01 | 1 | 7.3E-01 |
| FinnGen | Pancreas PDFF | Heart failure | Outlier-corrected | 0.016 | 0.06 | 0.26 | 8.0E-01 | 1 | 9.5E-01 |
| FinnGen | Pancreas PDFF | Atrial fibrillation | Raw | 0.176 | 0.10 | 1.84 | 9.0E-02 | 1 | 4.1E-01 |
| FinnGen | Pancreas PDFF | Atrial fibrillation | Outlier-corrected | 0.092 | 0.06 | 1.48 | 1.7E-01 | 1 | 5.8E-01 |
| FinnGen | Pancreas PDFF | Peripheral artery disease | Raw | -0.050 | 0.11 | -0.46 | 6.5E-01 | 1 | 8.9E-01 |
| FinnGen | Pancreas PDFF | Peripheral artery disease | Outlier-corrected | -0.140 | 0.08 | -1.82 | 9.9E-02 | 1 | 4.2E-01 |
| FinnGen | Pancreas PDFF | Deep vein thrombosis | Raw | 0.917 | 0.42 | 2.18 | 5.0E-02 | 2 | 2.9E-01 |
| FinnGen | Pancreas PDFF | Deep vein thrombosis | Outlier-corrected | 0.528 | 0.14 | 3.71 | 4.0E-03 | 2 | 7.2E-02 |
| FinnGen | Pancreas PDFF | Pulmonary embolism | Raw | 0.530 | 0.30 | 1.78 | 1.0E-01 | 1 | 4.2E-01 |
| FinnGen | Pancreas PDFF | Pulmonary embolism | Outlier-corrected | 0.207 | 0.11 | 1.91 | 8.2E-02 | 1 | 4.0E-01 |
| FinnGen | Pancreas PDFF | Gout | Raw | -0.105 | 0.14 | -0.73 | 4.8E-01 | NA | NA |
| FinnGen | Pancreas PDFF | Gout | Outlier-corrected | NA | NA | NA | NA | NA | NA |
| FinnGen | Pancreas PDFF | Knee osteoarthritis | Raw | 0.030 | 0.06 | 0.49 | 6.3E-01 | 1 | 8.9E-01 |
| FinnGen | Pancreas PDFF | Knee osteoarthritis | Outlier-corrected | -0.012 | 0.05 | -0.23 | 8.2E-01 | 1 | 9.5E-01 |
| FinnGen | Pancreas PDFF | Hip osteoarthritis | Raw | -0.038 | 0.08 | -0.45 | 6.6E-01 | 1 | 8.9E-01 |
| FinnGen | Pancreas PDFF | Hip osteoarthritis | Outlier-corrected | -0.083 | 0.08 | -1.09 | 3.0E-01 | 1 | 7.2E-01 |
| FinnGen | Pancreas PDFF | Rheumatoid arthritis | Raw | -0.179 | 0.15 | -1.21 | 2.5E-01 | 2 | 6.8E-01 |
| FinnGen | Pancreas PDFF | Rheumatoid arthritis | Outlier-corrected | 0.019 | 0.08 | 0.22 | 8.3E-01 | 2 | 9.5E-01 |
| FinnGen | Pancreas PDFF | Osteoporosis | Raw | -0.065 | 0.10 | -0.67 | 5.2E-01 | NA | NA |
| FinnGen | Pancreas PDFF | Osteoporosis | Outlier-corrected | NA | NA | NA | NA | NA | NA |
| FinnGen | Pancreas PDFF | Asthma | Raw | 0.135 | 0.08 | 1.73 | 1.1E-01 | 1 | 4.4E-01 |
| FinnGen | Pancreas PDFF | Asthma | Outlier-corrected | 0.077 | 0.06 | 1.22 | 2.5E-01 | 1 | 6.8E-01 |
| FinnGen | Pancreas PDFF | Psoriasis | Raw | -0.082 | 0.12 | -0.66 | 5.2E-01 | 1 | 8.6E-01 |
| FinnGen | Pancreas PDFF | Psoriasis | Outlier-corrected | 0.016 | 0.08 | 0.20 | 8.5E-01 | 1 | 9.6E-01 |
| FinnGen | Pancreas PDFF | Depression | Raw | -0.025 | 0.05 | -0.47 | 6.4E-01 | NA | NA |
| FinnGen | Pancreas PDFF | Depression | Outlier-corrected | NA | NA | NA | NA | NA | NA |
| FinnGen | Pancreas PDFF | Parkinson's disease | Raw | -0.436 | 0.16 | -2.71 | 1.9E-02 | 1 | 1.9E-01 |
| FinnGen | Pancreas PDFF | Parkinson's disease | Outlier-corrected | NA | NA | NA | NA | 1 | NA |
| FinnGen | Pancreas PDFF | Alzheimer’s disease | Raw | -0.136 | 0.10 | -1.34 | 2.0E-01 | NA | NA |
| FinnGen | Pancreas PDFF | Alzheimer’s disease | Outlier-corrected | NA | NA | NA | NA | NA | NA |
| FinnGen | Pancreas PDFF | Cholelithiasis | Raw | 0.249 | 0.13 | 1.88 | 8.5E-02 | 3 | 4.0E-01 |
| FinnGen | Pancreas PDFF | Cholelithiasis | Outlier-corrected | 0.131 | 0.07 | 1.87 | 9.4E-02 | 3 | 4.2E-01 |
| FinnGen | Pancreas PDFF | Gastroesophageal reflux disease | Raw | -0.031 | 0.06 | -0.53 | 6.1E-01 | NA | NA |
| FinnGen | Pancreas PDFF | Gastroesophageal reflux disease | Outlier-corrected | NA | NA | NA | NA | NA | NA |
| FinnGen | Paraspinal AT | Type 2 diabetes | Raw | -0.075 | 0.31 | -0.24 | 8.2E-01 | 2 | 9.5E-01 |
| FinnGen | Paraspinal AT | Type 2 diabetes | Outlier-corrected | -0.100 | 0.08 | -1.23 | 2.7E-01 | 2 | 7.1E-01 |
| FinnGen | Paraspinal AT | Polycystic ovary syndrome | Raw | -0.178 | 0.29 | -0.62 | 5.5E-01 | NA | NA |
| FinnGen | Paraspinal AT | Polycystic ovary syndrome | Outlier-corrected | NA | NA | NA | NA | NA | NA |
| FinnGen | Paraspinal AT | MASLD | Raw | -0.021 | 0.35 | -0.06 | 9.5E-01 | 3 | 9.7E-01 |
| FinnGen | Paraspinal AT | MASLD | Outlier-corrected | -0.243 | 0.22 | -1.11 | 3.3E-01 | 3 | 7.4E-01 |
| FinnGen | Paraspinal AT | Chronic kidney disease | Raw | -0.125 | 0.14 | -0.92 | 3.9E-01 | NA | NA |
| FinnGen | Paraspinal AT | Chronic kidney disease | Outlier-corrected | NA | NA | NA | NA | NA | NA |
| FinnGen | Paraspinal AT | Hypertension | Raw | 0.097 | 0.13 | 0.73 | 4.9E-01 | 4 | 8.5E-01 |
| FinnGen | Paraspinal AT | Hypertension | Outlier-corrected | 0.029 | 0.08 | 0.37 | 7.4E-01 | 4 | 9.3E-01 |
| FinnGen | Paraspinal AT | Coronary artery disease | Raw | -0.059 | 0.11 | -0.52 | 6.2E-01 | 1 | 8.9E-01 |
| FinnGen | Paraspinal AT | Coronary artery disease | Outlier-corrected | 0.053 | 0.09 | 0.58 | 5.8E-01 | 1 | 8.6E-01 |
| FinnGen | Paraspinal AT | Stroke | Raw | -0.075 | 0.07 | -1.01 | 3.4E-01 | NA | NA |
| FinnGen | Paraspinal AT | Stroke | Outlier-corrected | NA | NA | NA | NA | NA | NA |
| FinnGen | Paraspinal AT | Myocardial infarction | Raw | -0.149 | 0.06 | -2.33 | 5.2E-02 | NA | NA |
| FinnGen | Paraspinal AT | Myocardial infarction | Outlier-corrected | NA | NA | NA | NA | NA | NA |
| FinnGen | Paraspinal AT | Aortic aneurysm | Raw | 0.114 | 0.11 | 1.03 | 3.4E-01 | NA | NA |
| FinnGen | Paraspinal AT | Aortic aneurysm | Outlier-corrected | NA | NA | NA | NA | NA | NA |
| FinnGen | Paraspinal AT | Heart failure | Raw | -0.065 | 0.09 | -0.69 | 5.1E-01 | 1 | 8.6E-01 |
| FinnGen | Paraspinal AT | Heart failure | Outlier-corrected | -0.124 | 0.07 | -1.86 | 1.1E-01 | 1 | 4.4E-01 |
| FinnGen | Paraspinal AT | Atrial fibrillation | Raw | 0.169 | 0.12 | 1.36 | 2.1E-01 | 1 | 6.6E-01 |
| FinnGen | Paraspinal AT | Atrial fibrillation | Outlier-corrected | 0.078 | 0.06 | 1.29 | 2.5E-01 | 1 | 6.8E-01 |
| FinnGen | Paraspinal AT | Peripheral artery disease | Raw | -0.073 | 0.12 | -0.59 | 5.8E-01 | 1 | 8.6E-01 |
| FinnGen | Paraspinal AT | Peripheral artery disease | Outlier-corrected | 0.042 | 0.11 | 0.39 | 7.1E-01 | 1 | 9.2E-01 |
| FinnGen | Paraspinal AT | Deep vein thrombosis | Raw | 0.081 | 0.13 | 0.61 | 5.6E-01 | NA | NA |
| FinnGen | Paraspinal AT | Deep vein thrombosis | Outlier-corrected | NA | NA | NA | NA | NA | NA |
| FinnGen | Paraspinal AT | Pulmonary embolism | Raw | 0.162 | 0.08 | 1.96 | 9.1E-02 | NA | NA |
| FinnGen | Paraspinal AT | Pulmonary embolism | Outlier-corrected | NA | NA | NA | NA | NA | NA |
| FinnGen | Paraspinal AT | Gout | Raw | -0.275 | 0.18 | -1.54 | 1.7E-01 | NA | NA |
| FinnGen | Paraspinal AT | Gout | Outlier-corrected | NA | NA | NA | NA | NA | NA |
| FinnGen | Paraspinal AT | Knee osteoarthritis | Raw | 0.175 | 0.15 | 1.15 | 2.9E-01 | 1 | 7.2E-01 |
| FinnGen | Paraspinal AT | Knee osteoarthritis | Outlier-corrected | 0.059 | 0.06 | 0.94 | 3.8E-01 | 1 | 7.6E-01 |
| FinnGen | Paraspinal AT | Hip osteoarthritis | Raw | 0.254 | 0.13 | 1.93 | 9.5E-02 | 2 | 4.2E-01 |
| FinnGen | Paraspinal AT | Hip osteoarthritis | Outlier-corrected | 0.245 | 0.10 | 2.39 | 6.3E-02 | 2 | 3.2E-01 |
| FinnGen | Paraspinal AT | Rheumatoid arthritis | Raw | 0.007 | 0.08 | 0.09 | 9.3E-01 | NA | NA |
| FinnGen | Paraspinal AT | Rheumatoid arthritis | Outlier-corrected | NA | NA | NA | NA | NA | NA |
| FinnGen | Paraspinal AT | Osteoporosis | Raw | 0.252 | 0.14 | 1.81 | 1.1E-01 | NA | NA |
| FinnGen | Paraspinal AT | Osteoporosis | Outlier-corrected | NA | NA | NA | NA | NA | NA |
| FinnGen | Paraspinal AT | Asthma | Raw | -0.027 | 0.10 | -0.28 | 7.9E-01 | 2 | 9.5E-01 |
| FinnGen | Paraspinal AT | Asthma | Outlier-corrected | -0.030 | 0.08 | -0.35 | 7.4E-01 | 2 | 9.3E-01 |
| FinnGen | Paraspinal AT | Psoriasis | Raw | -0.002 | 0.09 | -0.02 | 9.8E-01 | NA | NA |
| FinnGen | Paraspinal AT | Psoriasis | Outlier-corrected | NA | NA | NA | NA | NA | NA |
| FinnGen | Paraspinal AT | Depression | Raw | 0.004 | 0.05 | 0.07 | 9.5E-01 | NA | NA |
| FinnGen | Paraspinal AT | Depression | Outlier-corrected | NA | NA | NA | NA | NA | NA |
| FinnGen | Paraspinal AT | Parkinson's disease | Raw | -0.039 | 0.10 | -0.38 | 7.2E-01 | NA | NA |
| FinnGen | Paraspinal AT | Parkinson's disease | Outlier-corrected | NA | NA | NA | NA | NA | NA |
| FinnGen | Paraspinal AT | Alzheimer’s disease | Raw | -0.006 | 0.11 | -0.05 | 9.6E-01 | NA | NA |
| FinnGen | Paraspinal AT | Alzheimer’s disease | Outlier-corrected | NA | NA | NA | NA | NA | NA |
| FinnGen | Paraspinal AT | Cholelithiasis | Raw | 0.040 | 0.12 | 0.32 | 7.6E-01 | 3 | 9.3E-01 |
| FinnGen | Paraspinal AT | Cholelithiasis | Outlier-corrected | 0.146 | 0.08 | 1.91 | 1.3E-01 | 3 | 5.0E-01 |
| FinnGen | Paraspinal AT | Gastroesophageal reflux disease | Raw | -0.062 | 0.07 | -0.83 | 4.3E-01 | NA | NA |
| FinnGen | Paraspinal AT | Gastroesophageal reflux disease | Outlier-corrected | NA | NA | NA | NA | NA | NA |
| FinnGen | Pelvic bone marrow fat | Type 2 diabetes | Raw | 0.004 | 0.07 | 0.07 | 9.5E-01 | 4 | 9.7E-01 |
| FinnGen | Pelvic bone marrow fat | Type 2 diabetes | Outlier-corrected | -0.009 | 0.03 | -0.25 | 8.0E-01 | 4 | 9.5E-01 |
| FinnGen | Pelvic bone marrow fat | Polycystic ovary syndrome | Raw | 0.108 | 0.16 | 0.66 | 5.2E-01 | NA | NA |
| FinnGen | Pelvic bone marrow fat | Polycystic ovary syndrome | Outlier-corrected | NA | NA | NA | NA | NA | NA |
| FinnGen | Pelvic bone marrow fat | MASLD | Raw | -0.118 | 0.12 | -0.97 | 3.4E-01 | NA | NA |
| FinnGen | Pelvic bone marrow fat | MASLD | Outlier-corrected | NA | NA | NA | NA | NA | NA |
| FinnGen | Pelvic bone marrow fat | Chronic kidney disease | Raw | -0.090 | 0.07 | -1.34 | 1.9E-01 | NA | NA |
| FinnGen | Pelvic bone marrow fat | Chronic kidney disease | Outlier-corrected | NA | NA | NA | NA | NA | NA |
| FinnGen | Pelvic bone marrow fat | Hypertension | Raw | -0.037 | 0.06 | -0.60 | 5.5E-01 | 4 | 8.6E-01 |
| FinnGen | Pelvic bone marrow fat | Hypertension | Outlier-corrected | -0.028 | 0.03 | -0.82 | 4.2E-01 | 4 | 7.9E-01 |
| FinnGen | Pelvic bone marrow fat | Coronary artery disease | Raw | -0.046 | 0.08 | -0.59 | 5.6E-01 | 1 | 8.6E-01 |
| FinnGen | Pelvic bone marrow fat | Coronary artery disease | Outlier-corrected | 0.010 | 0.06 | 0.17 | 8.7E-01 | 1 | 9.6E-01 |
| FinnGen | Pelvic bone marrow fat | Stroke | Raw | -0.041 | 0.06 | -0.68 | 5.0E-01 | NA | NA |
| FinnGen | Pelvic bone marrow fat | Stroke | Outlier-corrected | NA | NA | NA | NA | NA | NA |
| FinnGen | Pelvic bone marrow fat | Myocardial infarction | Raw | -0.001 | 0.07 | -0.01 | 9.9E-01 | 2 | 9.9E-01 |
| FinnGen | Pelvic bone marrow fat | Myocardial infarction | Outlier-corrected | 0.020 | 0.05 | 0.37 | 7.1E-01 | 2 | 9.2E-01 |
| FinnGen | Pelvic bone marrow fat | Aortic aneurysm | Raw | 0.054 | 0.08 | 0.66 | 5.1E-01 | 1 | 8.6E-01 |
| FinnGen | Pelvic bone marrow fat | Aortic aneurysm | Outlier-corrected | NA | NA | NA | NA | 1 | NA |
| FinnGen | Pelvic bone marrow fat | Heart failure | Raw | -0.046 | 0.05 | -0.96 | 3.5E-01 | 1 | 7.4E-01 |
| FinnGen | Pelvic bone marrow fat | Heart failure | Outlier-corrected | -0.067 | 0.04 | -1.50 | 1.5E-01 | 1 | 5.4E-01 |
| FinnGen | Pelvic bone marrow fat | Atrial fibrillation | Raw | -0.054 | 0.08 | -0.67 | 5.1E-01 | 4 | 8.6E-01 |
| FinnGen | Pelvic bone marrow fat | Atrial fibrillation | Outlier-corrected | -0.035 | 0.06 | -0.63 | 5.4E-01 | 4 | 8.6E-01 |
| FinnGen | Pelvic bone marrow fat | Peripheral artery disease | Raw | 0.034 | 0.10 | 0.34 | 7.3E-01 | 2 | 9.3E-01 |
| FinnGen | Pelvic bone marrow fat | Peripheral artery disease | Outlier-corrected | 0.059 | 0.07 | 0.79 | 4.4E-01 | 2 | 8.0E-01 |
| FinnGen | Pelvic bone marrow fat | Deep vein thrombosis | Raw | 0.126 | 0.11 | 1.15 | 2.6E-01 | 1 | 7.0E-01 |
| FinnGen | Pelvic bone marrow fat | Deep vein thrombosis | Outlier-corrected | 0.077 | 0.10 | 0.75 | 4.6E-01 | 1 | 8.2E-01 |
| FinnGen | Pelvic bone marrow fat | Pulmonary embolism | Raw | 0.069 | 0.07 | 0.94 | 3.6E-01 | 1 | 7.4E-01 |
| FinnGen | Pelvic bone marrow fat | Pulmonary embolism | Outlier-corrected | NA | NA | NA | NA | 1 | NA |
| FinnGen | Pelvic bone marrow fat | Gout | Raw | -0.220 | 0.09 | -2.52 | 1.8E-02 | NA | NA |
| FinnGen | Pelvic bone marrow fat | Gout | Outlier-corrected | NA | NA | NA | NA | NA | NA |
| FinnGen | Pelvic bone marrow fat | Knee osteoarthritis | Raw | -0.141 | 0.06 | -2.24 | 3.4E-02 | 3 | 2.4E-01 |
| FinnGen | Pelvic bone marrow fat | Knee osteoarthritis | Outlier-corrected | -0.064 | 0.04 | -1.83 | 8.0E-02 | 3 | 3.9E-01 |
| FinnGen | Pelvic bone marrow fat | Hip osteoarthritis | Raw | -0.081 | 0.06 | -1.36 | 1.8E-01 | 1 | 5.9E-01 |
| FinnGen | Pelvic bone marrow fat | Hip osteoarthritis | Outlier-corrected | -0.056 | 0.05 | -1.02 | 3.2E-01 | 1 | 7.3E-01 |
| FinnGen | Pelvic bone marrow fat | Rheumatoid arthritis | Raw | -0.229 | 0.09 | -2.43 | 2.2E-02 | 3 | 2.0E-01 |
| FinnGen | Pelvic bone marrow fat | Rheumatoid arthritis | Outlier-corrected | -0.155 | 0.06 | -2.51 | 1.9E-02 | 3 | 1.9E-01 |
| FinnGen | Pelvic bone marrow fat | Osteoporosis | Raw | 0.183 | 0.13 | 1.41 | 1.7E-01 | 4 | 5.8E-01 |
| FinnGen | Pelvic bone marrow fat | Osteoporosis | Outlier-corrected | 0.122 | 0.11 | 1.12 | 2.7E-01 | 4 | 7.1E-01 |
| FinnGen | Pelvic bone marrow fat | Asthma | Raw | -0.061 | 0.06 | -1.04 | 3.1E-01 | 1 | 7.2E-01 |
| FinnGen | Pelvic bone marrow fat | Asthma | Outlier-corrected | -0.012 | 0.04 | -0.32 | 7.5E-01 | 1 | 9.3E-01 |
| FinnGen | Pelvic bone marrow fat | Psoriasis | Raw | -0.089 | 0.10 | -0.93 | 3.6E-01 | 2 | 7.4E-01 |
| FinnGen | Pelvic bone marrow fat | Psoriasis | Outlier-corrected | -0.074 | 0.08 | -0.97 | 3.4E-01 | 2 | 7.4E-01 |
| FinnGen | Pelvic bone marrow fat | Depression | Raw | -0.036 | 0.03 | -1.04 | 3.1E-01 | 1 | 7.2E-01 |
| FinnGen | Pelvic bone marrow fat | Depression | Outlier-corrected | NA | NA | NA | NA | 1 | NA |
| FinnGen | Pelvic bone marrow fat | Parkinson's disease | Raw | -0.010 | 0.08 | -0.13 | 9.0E-01 | NA | NA |
| FinnGen | Pelvic bone marrow fat | Parkinson's disease | Outlier-corrected | NA | NA | NA | NA | NA | NA |
| FinnGen | Pelvic bone marrow fat | Alzheimer’s disease | Raw | -0.017 | 0.08 | -0.22 | 8.3E-01 | 1 | 9.5E-01 |
| FinnGen | Pelvic bone marrow fat | Alzheimer’s disease | Outlier-corrected | -0.065 | 0.07 | -1.00 | 3.3E-01 | 1 | 7.4E-01 |
| FinnGen | Pelvic bone marrow fat | Cholelithiasis | Raw | -0.109 | 0.06 | -1.69 | 1.0E-01 | 2 | 4.2E-01 |
| FinnGen | Pelvic bone marrow fat | Cholelithiasis | Outlier-corrected | -0.037 | 0.04 | -0.84 | 4.1E-01 | 2 | 7.8E-01 |
| FinnGen | Pelvic bone marrow fat | Gastroesophageal reflux disease | Raw | -0.015 | 0.05 | -0.31 | 7.6E-01 | 1 | 9.3E-01 |
| FinnGen | Pelvic bone marrow fat | Gastroesophageal reflux disease | Outlier-corrected | NA | NA | NA | NA | 1 | NA |
| FinnGen | Thigh bone marrow fat | Type 2 diabetes | Raw | -0.176 | 0.14 | -1.30 | 2.0E-01 | 7 | 6.3E-01 |
| FinnGen | Thigh bone marrow fat | Type 2 diabetes | Outlier-corrected | -0.075 | 0.03 | -2.72 | 1.3E-02 | 7 | 1.7E-01 |
| FinnGen | Thigh bone marrow fat | Polycystic ovary syndrome | Raw | -0.366 | 0.15 | -2.49 | 1.9E-02 | NA | NA |
| FinnGen | Thigh bone marrow fat | Polycystic ovary syndrome | Outlier-corrected | NA | NA | NA | NA | NA | NA |
| FinnGen | Thigh bone marrow fat | MASLD | Raw | -0.104 | 0.17 | -0.63 | 5.4E-01 | 1 | 8.6E-01 |
| FinnGen | Thigh bone marrow fat | MASLD | Outlier-corrected | -0.163 | 0.15 | -1.10 | 2.8E-01 | 1 | 7.2E-01 |
| FinnGen | Thigh bone marrow fat | Chronic kidney disease | Raw | 0.068 | 0.07 | 0.94 | 3.5E-01 | NA | NA |
| FinnGen | Thigh bone marrow fat | Chronic kidney disease | Outlier-corrected | NA | NA | NA | NA | NA | NA |
| FinnGen | Thigh bone marrow fat | Hypertension | Raw | 0.014 | 0.05 | 0.28 | 7.8E-01 | 6 | 9.5E-01 |
| FinnGen | Thigh bone marrow fat | Hypertension | Outlier-corrected | 0.011 | 0.03 | 0.33 | 7.5E-01 | 6 | 9.3E-01 |
| FinnGen | Thigh bone marrow fat | Coronary artery disease | Raw | 0.011 | 0.07 | 0.14 | 8.9E-01 | 1 | 9.6E-01 |
| FinnGen | Thigh bone marrow fat | Coronary artery disease | Outlier-corrected | 0.048 | 0.07 | 0.67 | 5.1E-01 | 1 | 8.6E-01 |
| FinnGen | Thigh bone marrow fat | Stroke | Raw | 0.001 | 0.06 | 0.02 | 9.8E-01 | NA | NA |
| FinnGen | Thigh bone marrow fat | Stroke | Outlier-corrected | NA | NA | NA | NA | NA | NA |
| FinnGen | Thigh bone marrow fat | Myocardial infarction | Raw | 0.031 | 0.05 | 0.65 | 5.2E-01 | NA | NA |
| FinnGen | Thigh bone marrow fat | Myocardial infarction | Outlier-corrected | NA | NA | NA | NA | NA | NA |
| FinnGen | Thigh bone marrow fat | Aortic aneurysm | Raw | -0.029 | 0.07 | -0.40 | 6.9E-01 | NA | NA |
| FinnGen | Thigh bone marrow fat | Aortic aneurysm | Outlier-corrected | NA | NA | NA | NA | NA | NA |
| FinnGen | Thigh bone marrow fat | Heart failure | Raw | 0.047 | 0.05 | 0.97 | 3.4E-01 | 1 | 7.4E-01 |
| FinnGen | Thigh bone marrow fat | Heart failure | Outlier-corrected | 0.019 | 0.04 | 0.45 | 6.6E-01 | 1 | 8.9E-01 |
| FinnGen | Thigh bone marrow fat | Atrial fibrillation | Raw | 0.096 | 0.08 | 1.14 | 2.6E-01 | 3 | 7.0E-01 |
| FinnGen | Thigh bone marrow fat | Atrial fibrillation | Outlier-corrected | 0.075 | 0.07 | 1.09 | 2.9E-01 | 3 | 7.2E-01 |
| FinnGen | Thigh bone marrow fat | Peripheral artery disease | Raw | -0.033 | 0.08 | -0.41 | 6.9E-01 | 1 | 9.1E-01 |
| FinnGen | Thigh bone marrow fat | Peripheral artery disease | Outlier-corrected | NA | NA | NA | NA | 1 | NA |
| FinnGen | Thigh bone marrow fat | Deep vein thrombosis | Raw | 0.043 | 0.09 | 0.49 | 6.3E-01 | NA | NA |
| FinnGen | Thigh bone marrow fat | Deep vein thrombosis | Outlier-corrected | NA | NA | NA | NA | NA | NA |
| FinnGen | Thigh bone marrow fat | Pulmonary embolism | Raw | 0.038 | 0.08 | 0.50 | 6.2E-01 | 1 | 8.9E-01 |
| FinnGen | Thigh bone marrow fat | Pulmonary embolism | Outlier-corrected | NA | NA | NA | NA | 1 | NA |
| FinnGen | Thigh bone marrow fat | Gout | Raw | -0.110 | 0.10 | -1.05 | 3.0E-01 | 1 | 7.2E-01 |
| FinnGen | Thigh bone marrow fat | Gout | Outlier-corrected | NA | NA | NA | NA | 1 | NA |
| FinnGen | Thigh bone marrow fat | Knee osteoarthritis | Raw | -0.190 | 0.06 | -3.14 | 4.0E-03 | 4 | 7.2E-02 |
| FinnGen | Thigh bone marrow fat | Knee osteoarthritis | Outlier-corrected | -0.135 | 0.04 | -3.18 | 4.0E-03 | 4 | 7.2E-02 |
| FinnGen | Thigh bone marrow fat | Hip osteoarthritis | Raw | -0.078 | 0.07 | -1.07 | 2.9E-01 | 2 | 7.2E-01 |
| FinnGen | Thigh bone marrow fat | Hip osteoarthritis | Outlier-corrected | -0.089 | 0.06 | -1.41 | 1.7E-01 | 2 | 5.8E-01 |
| FinnGen | Thigh bone marrow fat | Rheumatoid arthritis | Raw | -0.030 | 0.06 | -0.51 | 6.1E-01 | NA | NA |
| FinnGen | Thigh bone marrow fat | Rheumatoid arthritis | Outlier-corrected | NA | NA | NA | NA | NA | NA |
| FinnGen | Thigh bone marrow fat | Osteoporosis | Raw | 0.436 | 0.12 | 3.58 | 1.3E-03 | 4 | 4.8E-02 |
| FinnGen | Thigh bone marrow fat | Osteoporosis | Outlier-corrected | 0.344 | 0.11 | 3.18 | 4.0E-03 | 4 | 7.2E-02 |
| FinnGen | Thigh bone marrow fat | Asthma | Raw | 0.077 | 0.04 | 1.76 | 8.9E-02 | 1 | 4.1E-01 |
| FinnGen | Thigh bone marrow fat | Asthma | Outlier-corrected | NA | NA | NA | NA | 1 | NA |
| FinnGen | Thigh bone marrow fat | Psoriasis | Raw | -0.021 | 0.06 | -0.35 | 7.3E-01 | NA | NA |
| FinnGen | Thigh bone marrow fat | Psoriasis | Outlier-corrected | NA | NA | NA | NA | NA | NA |
| FinnGen | Thigh bone marrow fat | Depression | Raw | -0.050 | 0.04 | -1.22 | 2.3E-01 | 2 | 6.7E-01 |
| FinnGen | Thigh bone marrow fat | Depression | Outlier-corrected | -0.017 | 0.03 | -0.53 | 6.0E-01 | 2 | 8.7E-01 |
| FinnGen | Thigh bone marrow fat | Parkinson's disease | Raw | -0.138 | 0.09 | -1.49 | 1.5E-01 | NA | NA |
| FinnGen | Thigh bone marrow fat | Parkinson's disease | Outlier-corrected | NA | NA | NA | NA | NA | NA |
| FinnGen | Thigh bone marrow fat | Alzheimer’s disease | Raw | -0.100 | 0.06 | -1.60 | 1.2E-01 | NA | NA |
| FinnGen | Thigh bone marrow fat | Alzheimer’s disease | Outlier-corrected | NA | NA | NA | NA | NA | NA |
| FinnGen | Thigh bone marrow fat | Cholelithiasis | Raw | -0.036 | 0.07 | -0.54 | 5.9E-01 | 3 | 8.7E-01 |
| FinnGen | Thigh bone marrow fat | Cholelithiasis | Outlier-corrected | -0.019 | 0.05 | -0.38 | 7.1E-01 | 3 | 9.2E-01 |
| FinnGen | Thigh bone marrow fat | Gastroesophageal reflux disease | Raw | 0.004 | 0.04 | 0.10 | 9.2E-01 | NA | NA |
| FinnGen | Thigh bone marrow fat | Gastroesophageal reflux disease | Outlier-corrected | NA | NA | NA | NA | NA | NA |
| FinnGen | Vertebrae bone marrow fat | Type 2 diabetes | Raw | 0.037 | 0.11 | 0.33 | 7.6E-01 | 1 | 9.3E-01 |
| FinnGen | Vertebrae bone marrow fat | Type 2 diabetes | Outlier-corrected | -0.039 | 0.06 | -0.64 | 5.7E-01 | 1 | 8.6E-01 |
| FinnGen | Vertebrae bone marrow fat | Polycystic ovary syndrome | Raw | 0.226 | 0.44 | 0.51 | 6.4E-01 | NA | NA |
| FinnGen | Vertebrae bone marrow fat | Polycystic ovary syndrome | Outlier-corrected | NA | NA | NA | NA | NA | NA |
| FinnGen | Vertebrae bone marrow fat | MASLD | Raw | -0.067 | 0.11 | -0.61 | 5.7E-01 | NA | NA |
| FinnGen | Vertebrae bone marrow fat | MASLD | Outlier-corrected | NA | NA | NA | NA | NA | NA |
| FinnGen | Vertebrae bone marrow fat | Chronic kidney disease | Raw | 0.163 | 0.08 | 2.01 | 1.2E-01 | NA | NA |
| FinnGen | Vertebrae bone marrow fat | Chronic kidney disease | Outlier-corrected | NA | NA | NA | NA | NA | NA |
| FinnGen | Vertebrae bone marrow fat | Hypertension | Raw | 0.024 | 0.06 | 0.42 | 7.0E-01 | NA | NA |
| FinnGen | Vertebrae bone marrow fat | Hypertension | Outlier-corrected | NA | NA | NA | NA | NA | NA |
| FinnGen | Vertebrae bone marrow fat | Coronary artery disease | Raw | 0.022 | 0.07 | 0.30 | 7.8E-01 | NA | NA |
| FinnGen | Vertebrae bone marrow fat | Coronary artery disease | Outlier-corrected | NA | NA | NA | NA | NA | NA |
| FinnGen | Vertebrae bone marrow fat | Stroke | Raw | -0.014 | 0.16 | -0.09 | 9.3E-01 | NA | NA |
| FinnGen | Vertebrae bone marrow fat | Stroke | Outlier-corrected | NA | NA | NA | NA | NA | NA |
| FinnGen | Vertebrae bone marrow fat | Myocardial infarction | Raw | 0.014 | 0.07 | 0.21 | 8.5E-01 | NA | NA |
| FinnGen | Vertebrae bone marrow fat | Myocardial infarction | Outlier-corrected | NA | NA | NA | NA | NA | NA |
| FinnGen | Vertebrae bone marrow fat | Aortic aneurysm | Raw | -0.122 | 0.13 | -0.91 | 4.1E-01 | NA | NA |
| FinnGen | Vertebrae bone marrow fat | Aortic aneurysm | Outlier-corrected | NA | NA | NA | NA | NA | NA |
| FinnGen | Vertebrae bone marrow fat | Heart failure | Raw | -0.159 | 0.09 | -1.75 | 1.5E-01 | NA | NA |
| FinnGen | Vertebrae bone marrow fat | Heart failure | Outlier-corrected | NA | NA | NA | NA | NA | NA |
| FinnGen | Vertebrae bone marrow fat | Atrial fibrillation | Raw | -0.074 | 0.14 | -0.52 | 6.3E-01 | 1 | 8.9E-01 |
| FinnGen | Vertebrae bone marrow fat | Atrial fibrillation | Outlier-corrected | 0.085 | 0.06 | 1.48 | 2.3E-01 | 1 | 6.7E-01 |
| FinnGen | Vertebrae bone marrow fat | Peripheral artery disease | Raw | 0.026 | 0.15 | 0.18 | 8.7E-01 | NA | NA |
| FinnGen | Vertebrae bone marrow fat | Peripheral artery disease | Outlier-corrected | NA | NA | NA | NA | NA | NA |
| FinnGen | Vertebrae bone marrow fat | Deep vein thrombosis | Raw | -0.177 | 0.23 | -0.78 | 4.8E-01 | NA | NA |
| FinnGen | Vertebrae bone marrow fat | Deep vein thrombosis | Outlier-corrected | NA | NA | NA | NA | NA | NA |
| FinnGen | Vertebrae bone marrow fat | Pulmonary embolism | Raw | 0.012 | 0.25 | 0.05 | 9.6E-01 | 3 | 9.8E-01 |
| FinnGen | Vertebrae bone marrow fat | Pulmonary embolism | Outlier-corrected | -0.271 | 0.10 | -2.58 | 2.4E-01 | 3 | 6.7E-01 |
| FinnGen | Vertebrae bone marrow fat | Gout | Raw | 0.061 | 0.20 | 0.30 | 7.8E-01 | NA | NA |
| FinnGen | Vertebrae bone marrow fat | Gout | Outlier-corrected | NA | NA | NA | NA | NA | NA |
| FinnGen | Vertebrae bone marrow fat | Knee osteoarthritis | Raw | 0.022 | 0.05 | 0.49 | 6.5E-01 | NA | NA |
| FinnGen | Vertebrae bone marrow fat | Knee osteoarthritis | Outlier-corrected | NA | NA | NA | NA | NA | NA |
| FinnGen | Vertebrae bone marrow fat | Hip osteoarthritis | Raw | 0.214 | 0.10 | 2.09 | 1.1E-01 | NA | NA |
| FinnGen | Vertebrae bone marrow fat | Hip osteoarthritis | Outlier-corrected | NA | NA | NA | NA | NA | NA |
| FinnGen | Vertebrae bone marrow fat | Rheumatoid arthritis | Raw | -0.090 | 0.16 | -0.56 | 6.0E-01 | NA | NA |
| FinnGen | Vertebrae bone marrow fat | Rheumatoid arthritis | Outlier-corrected | NA | NA | NA | NA | NA | NA |
| FinnGen | Vertebrae bone marrow fat | Osteoporosis | Raw | -0.235 | 0.23 | -1.04 | 3.6E-01 | 1 | 7.4E-01 |
| FinnGen | Vertebrae bone marrow fat | Osteoporosis | Outlier-corrected | -0.469 | 0.14 | -3.27 | 4.7E-02 | 1 | 2.8E-01 |
| FinnGen | Vertebrae bone marrow fat | Asthma | Raw | -0.106 | 0.08 | -1.33 | 2.5E-01 | NA | NA |
| FinnGen | Vertebrae bone marrow fat | Asthma | Outlier-corrected | NA | NA | NA | NA | NA | NA |
| FinnGen | Vertebrae bone marrow fat | Psoriasis | Raw | -0.093 | 0.10 | -0.97 | 3.9E-01 | NA | NA |
| FinnGen | Vertebrae bone marrow fat | Psoriasis | Outlier-corrected | NA | NA | NA | NA | NA | NA |
| FinnGen | Vertebrae bone marrow fat | Depression | Raw | -0.018 | 0.07 | -0.24 | 8.2E-01 | NA | NA |
| FinnGen | Vertebrae bone marrow fat | Depression | Outlier-corrected | NA | NA | NA | NA | NA | NA |
| FinnGen | Vertebrae bone marrow fat | Parkinson's disease | Raw | 0.116 | 0.15 | 0.78 | 4.8E-01 | NA | NA |
| FinnGen | Vertebrae bone marrow fat | Parkinson's disease | Outlier-corrected | NA | NA | NA | NA | NA | NA |
| FinnGen | Vertebrae bone marrow fat | Alzheimer’s disease | Raw | 0.162 | 0.20 | 0.81 | 4.6E-01 | NA | NA |
| FinnGen | Vertebrae bone marrow fat | Alzheimer’s disease | Outlier-corrected | NA | NA | NA | NA | NA | NA |
| FinnGen | Vertebrae bone marrow fat | Cholelithiasis | Raw | -0.035 | 0.04 | -0.99 | 3.8E-01 | NA | NA |
| FinnGen | Vertebrae bone marrow fat | Cholelithiasis | Outlier-corrected | NA | NA | NA | NA | NA | NA |
| FinnGen | Vertebrae bone marrow fat | Gastroesophageal reflux disease | Raw | 0.099 | 0.06 | 1.68 | 1.7E-01 | NA | NA |
| FinnGen | Vertebrae bone marrow fat | Gastroesophageal reflux disease | Outlier-corrected | NA | NA | NA | NA | NA | NA |
| Pubgwas | Thigh subcutaneous AT | Type 2 diabetes | Raw | -0.476 | 0.22 | -2.17 | 4.3E-02 | 9 | 2.9E-01 |
| Pubgwas | Thigh subcutaneous AT | Type 2 diabetes | Outlier-corrected | -0.498 | 0.08 | -6.62 | 5.9E-05 | 9 | 3.6E-03 |
| Pubgwas | Thigh subcutaneous AT | Polycystic ovary syndrome | Raw | -0.158 | 0.14 | -1.09 | 2.9E-01 | NA | NA |
| Pubgwas | Thigh subcutaneous AT | Polycystic ovary syndrome | Outlier-corrected | NA | NA | NA | NA | NA | NA |
| Pubgwas | Thigh subcutaneous AT | MASLD | Raw | -0.117 | 0.15 | -0.77 | 4.5E-01 | 1 | 7.9E-01 |
| Pubgwas | Thigh subcutaneous AT | MASLD | Outlier-corrected | -0.194 | 0.12 | -1.56 | 1.4E-01 | 1 | 4.6E-01 |
| Pubgwas | Thigh subcutaneous AT | Chronic kidney disease | Raw | -0.057 | 0.08 | -0.75 | 4.6E-01 | 0 | NA |
| Pubgwas | Thigh subcutaneous AT | Chronic kidney disease | Outlier-corrected | NA | NA | NA | NA | 0 | NA |
| Pubgwas | Thigh subcutaneous AT | Hypertension | Raw | -0.072 | 0.01 | -5.39 | 2.1E-05 | 5 | 1.7E-03 |
| Pubgwas | Thigh subcutaneous AT | Hypertension | Outlier-corrected | -0.075 | 0.01 | -9.13 | 5.7E-08 | 5 | 1.4E-05 |
| Pubgwas | Thigh subcutaneous AT | Coronary artery disease | Raw | -0.311 | 0.09 | -3.30 | 3.6E-03 | 2 | 4.9E-02 |
| Pubgwas | Thigh subcutaneous AT | Coronary artery disease | Outlier-corrected | -0.308 | 0.09 | -3.46 | 2.8E-03 | 2 | 4.1E-02 |
| Pubgwas | Thigh subcutaneous AT | Stroke | Raw | -0.089 | 0.08 | -1.17 | 2.6E-01 | 1 | 6.2E-01 |
| Pubgwas | Thigh subcutaneous AT | Stroke | Outlier-corrected | -0.135 | 0.06 | -2.16 | 4.3E-02 | 1 | 2.9E-01 |
| Pubgwas | Thigh subcutaneous AT | Myocardial infarction | Raw | -0.373 | 0.09 | -4.07 | 5.5E-04 | 2 | 1.5E-02 |
| Pubgwas | Thigh subcutaneous AT | Myocardial infarction | Outlier-corrected | -0.302 | 0.08 | -3.85 | 1.1E-03 | 2 | 2.4E-02 |
| Pubgwas | Thigh subcutaneous AT | Aortic aneurysm | Raw | -0.502 | 0.15 | -3.25 | 3.6E-03 | 0 | NA |
| Pubgwas | Thigh subcutaneous AT | Aortic aneurysm | Outlier-corrected | NA | NA | NA | NA | 0 | NA |
| Pubgwas | Thigh subcutaneous AT | Heart failure | Raw | 0.022 | 0.09 | 0.26 | 8.0E-01 | 3 | 9.3E-01 |
| Pubgwas | Thigh subcutaneous AT | Heart failure | Outlier-corrected | -0.091 | 0.06 | -1.59 | 1.3E-01 | 3 | 4.6E-01 |
| Pubgwas | Thigh subcutaneous AT | Atrial fibrillation | Raw | 0.098 | 0.08 | 1.21 | 2.4E-01 | 3 | 6.2E-01 |
| Pubgwas | Thigh subcutaneous AT | Atrial fibrillation | Outlier-corrected | 0.093 | 0.05 | 1.93 | 6.8E-02 | 3 | 3.5E-01 |
| Pubgwas | Thigh subcutaneous AT | Peripheral artery disease | Raw | -0.178 | 0.09 | -1.96 | 6.3E-02 | NA | NA |
| Pubgwas | Thigh subcutaneous AT | Peripheral artery disease | Outlier-corrected | NA | NA | NA | NA | NA | NA |
| Pubgwas | Thigh subcutaneous AT | Deep vein thrombosis | Raw | 0.001 | 0.00 | 0.40 | 6.9E-01 | NA | NA |
| Pubgwas | Thigh subcutaneous AT | Deep vein thrombosis | Outlier-corrected | NA | NA | NA | NA | NA | NA |
| Pubgwas | Thigh subcutaneous AT | Pulmonary embolism | Raw | 0.234 | 0.16 | 1.45 | 1.6E-01 | 0 | NA |
| Pubgwas | Thigh subcutaneous AT | Pulmonary embolism | Outlier-corrected | NA | NA | NA | NA | 0 | NA |
| Pubgwas | Thigh subcutaneous AT | Gout | Raw | -0.189 | 0.19 | -0.99 | 3.3E-01 | NA | NA |
| Pubgwas | Thigh subcutaneous AT | Gout | Outlier-corrected | NA | NA | NA | NA | NA | NA |
| Pubgwas | Thigh subcutaneous AT | Knee osteoarthritis | Raw | 0.308 | 0.08 | 3.69 | 1.3E-03 | 3 | 2.6E-02 |
| Pubgwas | Thigh subcutaneous AT | Knee osteoarthritis | Outlier-corrected | 0.269 | 0.07 | 4.01 | 7.5E-04 | 3 | 1.8E-02 |
| Pubgwas | Thigh subcutaneous AT | Hip osteoarthritis | Raw | 0.041 | 0.11 | 0.39 | 7.0E-01 | 2 | 8.9E-01 |
| Pubgwas | Thigh subcutaneous AT | Hip osteoarthritis | Outlier-corrected | -0.070 | 0.07 | -0.94 | 3.6E-01 | 2 | 7.2E-01 |
| Pubgwas | Thigh subcutaneous AT | Rheumatoid arthritis | Raw | -0.067 | 0.14 | -0.47 | 6.5E-01 | 1 | 8.7E-01 |
| Pubgwas | Thigh subcutaneous AT | Rheumatoid arthritis | Outlier-corrected | -0.015 | 0.06 | -0.25 | 8.1E-01 | 1 | 9.3E-01 |
| Pubgwas | Thigh subcutaneous AT | Osteoporosis | Raw | 0.006 | 0.00 | 2.97 | 7.1E-03 | 1 | 8.6E-02 |
| Pubgwas | Thigh subcutaneous AT | Osteoporosis | Outlier-corrected | 0.007 | 0.00 | 3.39 | 2.8E-03 | 1 | 4.1E-02 |
| Pubgwas | Thigh subcutaneous AT | Asthma | Raw | -0.033 | 0.06 | -0.56 | 5.8E-01 | 3 | 8.4E-01 |
| Pubgwas | Thigh subcutaneous AT | Asthma | Outlier-corrected | -0.061 | 0.04 | -1.39 | 1.8E-01 | 3 | 5.6E-01 |
| Pubgwas | Thigh subcutaneous AT | Psoriasis | Raw | 0.032 | 0.13 | 0.25 | 8.0E-01 | 2 | 9.3E-01 |
| Pubgwas | Thigh subcutaneous AT | Psoriasis | Outlier-corrected | -0.103 | 0.08 | -1.27 | 2.2E-01 | 2 | 5.9E-01 |
| Pubgwas | Thigh subcutaneous AT | Depression | Raw | 0.008 | 0.07 | 0.12 | 9.1E-01 | NA | NA |
| Pubgwas | Thigh subcutaneous AT | Depression | Outlier-corrected | NA | NA | NA | NA | NA | NA |
| Pubgwas | Thigh subcutaneous AT | Parkinson's disease | Raw | 0.050 | 0.12 | 0.41 | 6.9E-01 | 2 | 8.8E-01 |
| Pubgwas | Thigh subcutaneous AT | Parkinson's disease | Outlier-corrected | 0.017 | 0.08 | 0.21 | 8.4E-01 | 2 | 9.4E-01 |
| Pubgwas | Thigh subcutaneous AT | Alzheimer’s disease | Raw | 0.024 | 0.05 | 0.52 | 6.1E-01 | NA | NA |
| Pubgwas | Thigh subcutaneous AT | Alzheimer’s disease | Outlier-corrected | NA | NA | NA | NA | NA | NA |
| Pubgwas | Thigh subcutaneous AT | Cholelithiasis | Raw | 0.066 | 0.07 | 0.89 | 3.8E-01 | 2 | 7.4E-01 |
| Pubgwas | Thigh subcutaneous AT | Cholelithiasis | Outlier-corrected | 0.059 | 0.04 | 1.31 | 2.1E-01 | 2 | 5.8E-01 |
| Pubgwas | Thigh subcutaneous AT | Gastroesophageal reflux disease | Raw | 0.132 | 0.07 | 1.79 | 9.1E-02 | 2 | 3.8E-01 |
| Pubgwas | Thigh subcutaneous AT | Gastroesophageal reflux disease | Outlier-corrected | 0.048 | 0.04 | 1.08 | 3.0E-01 | 2 | 6.5E-01 |
| Pubgwas | Thigh intermuscular AT | Type 2 diabetes | Raw | -0.646 | 0.13 | -4.90 | 2.9E-04 | 4 | 1.0E-02 |
| Pubgwas | Thigh intermuscular AT | Type 2 diabetes | Outlier-corrected | -0.552 | 0.06 | -8.81 | 1.0E-05 | 4 | 1.2E-03 |
| Pubgwas | Thigh intermuscular AT | Polycystic ovary syndrome | Raw | -0.504 | 0.20 | -2.55 | 2.4E-02 | 1 | 1.8E-01 |
| Pubgwas | Thigh intermuscular AT | Polycystic ovary syndrome | Outlier-corrected | -0.323 | 0.17 | -1.85 | 8.9E-02 | 1 | 3.8E-01 |
| Pubgwas | Thigh intermuscular AT | MASLD | Raw | -0.391 | 0.07 | -5.62 | 8.3E-05 | NA | NA |
| Pubgwas | Thigh intermuscular AT | MASLD | Outlier-corrected | NA | NA | NA | NA | NA | NA |
| Pubgwas | Thigh intermuscular AT | Chronic kidney disease | Raw | -0.063 | 0.08 | -0.82 | 4.3E-01 | 2 | 7.8E-01 |
| Pubgwas | Thigh intermuscular AT | Chronic kidney disease | Outlier-corrected | 0.021 | 0.05 | 0.42 | 6.8E-01 | 2 | 8.8E-01 |
| Pubgwas | Thigh intermuscular AT | Hypertension | Raw | -0.057 | 0.01 | -5.09 | 2.1E-04 | 2 | 8.4E-03 |
| Pubgwas | Thigh intermuscular AT | Hypertension | Outlier-corrected | -0.059 | 0.01 | -6.07 | 8.1E-05 | 2 | 3.9E-03 |
| Pubgwas | Thigh intermuscular AT | Coronary artery disease | Raw | -0.302 | 0.07 | -4.48 | 6.2E-04 | NA | NA |
| Pubgwas | Thigh intermuscular AT | Coronary artery disease | Outlier-corrected | NA | NA | NA | NA | NA | NA |
| Pubgwas | Thigh intermuscular AT | Stroke | Raw | -0.087 | 0.07 | -1.21 | 2.5E-01 | NA | NA |
| Pubgwas | Thigh intermuscular AT | Stroke | Outlier-corrected | NA | NA | NA | NA | NA | NA |
| Pubgwas | Thigh intermuscular AT | Myocardial infarction | Raw | -0.103 | 0.09 | -1.20 | 2.5E-01 | 1 | 6.2E-01 |
| Pubgwas | Thigh intermuscular AT | Myocardial infarction | Outlier-corrected | -0.042 | 0.06 | -0.65 | 5.3E-01 | 1 | 8.3E-01 |
| Pubgwas | Thigh intermuscular AT | Aortic aneurysm | Raw | -0.283 | 0.15 | -1.88 | 8.3E-02 | NA | NA |
| Pubgwas | Thigh intermuscular AT | Aortic aneurysm | Outlier-corrected | NA | NA | NA | NA | NA | NA |
| Pubgwas | Thigh intermuscular AT | Heart failure | Raw | -0.086 | 0.03 | -3.09 | 8.6E-03 | NA | NA |
| Pubgwas | Thigh intermuscular AT | Heart failure | Outlier-corrected | NA | NA | NA | NA | NA | NA |
| Pubgwas | Thigh intermuscular AT | Atrial fibrillation | Raw | 0.014 | 0.06 | 0.22 | 8.3E-01 | 2 | 9.3E-01 |
| Pubgwas | Thigh intermuscular AT | Atrial fibrillation | Outlier-corrected | -0.002 | 0.05 | -0.04 | 9.7E-01 | 2 | 9.8E-01 |
| Pubgwas | Thigh intermuscular AT | Peripheral artery disease | Raw | -0.192 | 0.11 | -1.74 | 1.0E-01 | NA | NA |
| Pubgwas | Thigh intermuscular AT | Peripheral artery disease | Outlier-corrected | NA | NA | NA | NA | NA | NA |
| Pubgwas | Thigh intermuscular AT | Deep vein thrombosis | Raw | 0.000 | 0.00 | -0.12 | 9.1E-01 | NA | NA |
| Pubgwas | Thigh intermuscular AT | Deep vein thrombosis | Outlier-corrected | NA | NA | NA | NA | NA | NA |
| Pubgwas | Thigh intermuscular AT | Pulmonary embolism | Raw | 0.149 | 0.14 | 1.08 | 3.0E-01 | NA | NA |
| Pubgwas | Thigh intermuscular AT | Pulmonary embolism | Outlier-corrected | NA | NA | NA | NA | NA | NA |
| Pubgwas | Thigh intermuscular AT | Gout | Raw | -0.074 | 0.25 | -0.29 | 7.7E-01 | NA | NA |
| Pubgwas | Thigh intermuscular AT | Gout | Outlier-corrected | NA | NA | NA | NA | NA | NA |
| Pubgwas | Thigh intermuscular AT | Knee osteoarthritis | Raw | 0.018 | 0.08 | 0.23 | 8.2E-01 | 1 | 9.3E-01 |
| Pubgwas | Thigh intermuscular AT | Knee osteoarthritis | Outlier-corrected | 0.057 | 0.08 | 0.76 | 4.6E-01 | 1 | 7.9E-01 |
| Pubgwas | Thigh intermuscular AT | Hip osteoarthritis | Raw | -0.084 | 0.06 | -1.45 | 1.7E-01 | NA | NA |
| Pubgwas | Thigh intermuscular AT | Hip osteoarthritis | Outlier-corrected | NA | NA | NA | NA | NA | NA |
| Pubgwas | Thigh intermuscular AT | Rheumatoid arthritis | Raw | 0.070 | 0.12 | 0.59 | 5.6E-01 | 2 | 8.4E-01 |
| Pubgwas | Thigh intermuscular AT | Rheumatoid arthritis | Outlier-corrected | 0.073 | 0.09 | 0.80 | 4.4E-01 | 2 | 7.9E-01 |
| Pubgwas | Thigh intermuscular AT | Osteoporosis | Raw | 0.003 | 0.00 | 1.29 | 2.2E-01 | 0 | NA |
| Pubgwas | Thigh intermuscular AT | Osteoporosis | Outlier-corrected | NA | NA | NA | NA | 0 | NA |
| Pubgwas | Thigh intermuscular AT | Asthma | Raw | 0.088 | 0.03 | 2.52 | 2.6E-02 | NA | NA |
| Pubgwas | Thigh intermuscular AT | Asthma | Outlier-corrected | NA | NA | NA | NA | NA | NA |
| Pubgwas | Thigh intermuscular AT | Psoriasis | Raw | 0.136 | 0.12 | 1.16 | 2.7E-01 | NA | NA |
| Pubgwas | Thigh intermuscular AT | Psoriasis | Outlier-corrected | NA | NA | NA | NA | NA | NA |
| Pubgwas | Thigh intermuscular AT | Depression | Raw | 0.000 | 0.08 | 0.01 | 1.0E+00 | NA | NA |
| Pubgwas | Thigh intermuscular AT | Depression | Outlier-corrected | NA | NA | NA | NA | NA | NA |
| Pubgwas | Thigh intermuscular AT | Parkinson's disease | Raw | -0.005 | 0.09 | -0.05 | 9.6E-01 | NA | NA |
| Pubgwas | Thigh intermuscular AT | Parkinson's disease | Outlier-corrected | NA | NA | NA | NA | NA | NA |
| Pubgwas | Thigh intermuscular AT | Alzheimer’s disease | Raw | -0.044 | 0.05 | -0.88 | 4.0E-01 | NA | NA |
| Pubgwas | Thigh intermuscular AT | Alzheimer’s disease | Outlier-corrected | NA | NA | NA | NA | NA | NA |
| Pubgwas | Thigh intermuscular AT | Cholelithiasis | Raw | -0.005 | 0.05 | -0.10 | 9.2E-01 | NA | NA |
| Pubgwas | Thigh intermuscular AT | Cholelithiasis | Outlier-corrected | NA | NA | NA | NA | NA | NA |
| Pubgwas | Thigh intermuscular AT | Gastroesophageal reflux disease | Raw | 0.060 | 0.06 | 1.06 | 3.1E-01 | 2 | 6.6E-01 |
| Pubgwas | Thigh intermuscular AT | Gastroesophageal reflux disease | Outlier-corrected | 0.118 | 0.04 | 3.02 | 1.3E-02 | 2 | 1.2E-01 |
| Pubgwas | Visceral AT | Type 2 diabetes | Raw | 0.124 | 0.56 | 0.22 | 8.3E-01 | 4 | 9.4E-01 |
| Pubgwas | Visceral AT | Type 2 diabetes | Outlier-corrected | 0.391 | 0.08 | 4.64 | 4.3E-02 | 4 | 2.9E-01 |
| Pubgwas | Visceral AT | Polycystic ovary syndrome | Raw | 0.169 | 0.26 | 0.66 | 5.4E-01 | NA | NA |
| Pubgwas | Visceral AT | Polycystic ovary syndrome | Outlier-corrected | NA | NA | NA | NA | NA | NA |
| Pubgwas | Visceral AT | MASLD | Raw | 0.155 | 0.33 | 0.47 | 6.5E-01 | 2 | 8.7E-01 |
| Pubgwas | Visceral AT | MASLD | Outlier-corrected | 0.069 | 0.22 | 0.31 | 7.7E-01 | 2 | 9.2E-01 |
| Pubgwas | Visceral AT | Chronic kidney disease | Raw | -0.078 | 0.19 | -0.42 | 6.9E-01 | 1 | 8.8E-01 |
| Pubgwas | Visceral AT | Chronic kidney disease | Outlier-corrected | 0.024 | 0.18 | 0.14 | 9.0E-01 | 1 | 9.6E-01 |
| Pubgwas | Visceral AT | Hypertension | Raw | 0.001 | 0.04 | 0.04 | 9.7E-01 | 3 | 9.8E-01 |
| Pubgwas | Visceral AT | Hypertension | Outlier-corrected | 0.015 | 0.02 | 0.90 | 4.4E-01 | 3 | 7.9E-01 |
| Pubgwas | Visceral AT | Coronary artery disease | Raw | -0.168 | 0.21 | -0.79 | 4.6E-01 | 2 | 7.9E-01 |
| Pubgwas | Visceral AT | Coronary artery disease | Outlier-corrected | -0.477 | 0.07 | -6.53 | 2.8E-03 | 2 | 4.1E-02 |
| Pubgwas | Visceral AT | Stroke | Raw | 0.124 | 0.12 | 1.07 | 3.2E-01 | NA | NA |
| Pubgwas | Visceral AT | Stroke | Outlier-corrected | NA | NA | NA | NA | NA | NA |
| Pubgwas | Visceral AT | Myocardial infarction | Raw | -0.047 | 0.19 | -0.25 | 8.1E-01 | 2 | 9.3E-01 |
| Pubgwas | Visceral AT | Myocardial infarction | Outlier-corrected | -0.294 | 0.06 | -4.87 | 8.2E-03 | 2 | 9.2E-02 |
| Pubgwas | Visceral AT | Aortic aneurysm | Raw | -0.313 | 0.26 | -1.22 | 2.7E-01 | NA | NA |
| Pubgwas | Visceral AT | Aortic aneurysm | Outlier-corrected | NA | NA | NA | NA | NA | NA |
| Pubgwas | Visceral AT | Heart failure | Raw | 0.135 | 0.21 | 0.65 | 5.4E-01 | 2 | 8.3E-01 |
| Pubgwas | Visceral AT | Heart failure | Outlier-corrected | -0.159 | 0.04 | -4.02 | 1.6E-02 | 2 | 1.5E-01 |
| Pubgwas | Visceral AT | Atrial fibrillation | Raw | -0.049 | 0.16 | -0.31 | 7.7E-01 | 2 | 9.2E-01 |
| Pubgwas | Visceral AT | Atrial fibrillation | Outlier-corrected | -0.106 | 0.07 | -1.49 | 2.1E-01 | 2 | 5.8E-01 |
| Pubgwas | Visceral AT | Peripheral artery disease | Raw | 0.064 | 0.17 | 0.39 | 7.1E-01 | NA | NA |
| Pubgwas | Visceral AT | Peripheral artery disease | Outlier-corrected | NA | NA | NA | NA | NA | NA |
| Pubgwas | Visceral AT | Deep vein thrombosis | Raw | 0.005 | 0.00 | 1.93 | 1.0E-01 | NA | NA |
| Pubgwas | Visceral AT | Deep vein thrombosis | Outlier-corrected | NA | NA | NA | NA | NA | NA |
| Pubgwas | Visceral AT | Pulmonary embolism | Raw | 0.374 | 0.19 | 1.99 | 9.4E-02 | NA | NA |
| Pubgwas | Visceral AT | Pulmonary embolism | Outlier-corrected | NA | NA | NA | NA | NA | NA |
| Pubgwas | Visceral AT | Gout | Raw | -0.003 | 0.02 | -0.17 | 8.7E-01 | NA | NA |
| Pubgwas | Visceral AT | Gout | Outlier-corrected | NA | NA | NA | NA | NA | NA |
| Pubgwas | Visceral AT | Knee osteoarthritis | Raw | 0.312 | 0.19 | 1.67 | 1.5E-01 | 1 | 4.9E-01 |
| Pubgwas | Visceral AT | Knee osteoarthritis | Outlier-corrected | 0.189 | 0.16 | 1.20 | 2.8E-01 | 1 | 6.4E-01 |
| Pubgwas | Visceral AT | Hip osteoarthritis | Raw | 0.312 | 0.21 | 1.50 | 1.8E-01 | 1 | 5.6E-01 |
| Pubgwas | Visceral AT | Hip osteoarthritis | Outlier-corrected | 0.138 | 0.12 | 1.19 | 2.9E-01 | 1 | 6.4E-01 |
| Pubgwas | Visceral AT | Rheumatoid arthritis | Raw | -0.073 | 0.29 | -0.26 | 8.1E-01 | 2 | 9.3E-01 |
| Pubgwas | Visceral AT | Rheumatoid arthritis | Outlier-corrected | -0.112 | 0.21 | -0.54 | 6.2E-01 | 2 | 8.6E-01 |
| Pubgwas | Visceral AT | Osteoporosis | Raw | -0.001 | 0.00 | -0.32 | 7.6E-01 | 1 | 9.2E-01 |
| Pubgwas | Visceral AT | Osteoporosis | Outlier-corrected | -0.004 | 0.00 | -1.23 | 2.7E-01 | 1 | 6.3E-01 |
| Pubgwas | Visceral AT | Asthma | Raw | 0.158 | 0.07 | 2.15 | 7.5E-02 | NA | NA |
| Pubgwas | Visceral AT | Asthma | Outlier-corrected | NA | NA | NA | NA | NA | NA |
| Pubgwas | Visceral AT | Psoriasis | Raw | 0.277 | 0.37 | 0.76 | 4.8E-01 | 2 | 7.9E-01 |
| Pubgwas | Visceral AT | Psoriasis | Outlier-corrected | 0.277 | 0.15 | 1.90 | 1.3E-01 | 2 | 4.6E-01 |
| Pubgwas | Visceral AT | Depression | Raw | 0.224 | 0.15 | 1.54 | 1.8E-01 | NA | NA |
| Pubgwas | Visceral AT | Depression | Outlier-corrected | NA | NA | NA | NA | NA | NA |
| Pubgwas | Visceral AT | Parkinson's disease | Raw | 0.118 | 0.25 | 0.48 | 6.5E-01 | NA | NA |
| Pubgwas | Visceral AT | Parkinson's disease | Outlier-corrected | NA | NA | NA | NA | NA | NA |
| Pubgwas | Visceral AT | Alzheimer’s disease | Raw | -0.049 | 0.13 | -0.36 | 7.3E-01 | NA | NA |
| Pubgwas | Visceral AT | Alzheimer’s disease | Outlier-corrected | NA | NA | NA | NA | NA | NA |
| Pubgwas | Visceral AT | Cholelithiasis | Raw | 0.386 | 0.17 | 2.26 | 6.4E-02 | 1 | 3.4E-01 |
| Pubgwas | Visceral AT | Cholelithiasis | Outlier-corrected | 0.244 | 0.10 | 2.48 | 5.6E-02 | 1 | 3.3E-01 |
| Pubgwas | Visceral AT | Gastroesophageal reflux disease | Raw | 0.295 | 0.12 | 2.50 | 5.4E-02 | 1 | 3.3E-01 |
| Pubgwas | Visceral AT | Gastroesophageal reflux disease | Outlier-corrected | 0.196 | 0.08 | 2.55 | 6.4E-02 | 1 | 3.4E-01 |
| Pubgwas | Liver PDFF | Type 2 diabetes | Raw | 0.211 | 0.11 | 1.85 | 1.0E-01 | 4 | 4.0E-01 |
| Pubgwas | Liver PDFF | Type 2 diabetes | Outlier-corrected | 0.215 | 0.06 | 3.68 | 2.1E-02 | 4 | 1.8E-01 |
| Pubgwas | Liver PDFF | Polycystic ovary syndrome | Raw | 0.061 | 0.06 | 0.95 | 3.7E-01 | NA | NA |
| Pubgwas | Liver PDFF | Polycystic ovary syndrome | Outlier-corrected | NA | NA | NA | NA | NA | NA |
| Pubgwas | Liver PDFF | MASLD | Raw | 1.157 | 0.09 | 12.60 | 1.5E-05 | NA | NA |
| Pubgwas | Liver PDFF | MASLD | Outlier-corrected | NA | NA | NA | NA | NA | NA |
| Pubgwas | Liver PDFF | Chronic kidney disease | Raw | -0.045 | 0.09 | -0.52 | 6.2E-01 | 2 | 8.7E-01 |
| Pubgwas | Liver PDFF | Chronic kidney disease | Outlier-corrected | 0.009 | 0.06 | 0.16 | 8.8E-01 | 2 | 9.6E-01 |
| Pubgwas | Liver PDFF | Hypertension | Raw | 0.011 | 0.01 | 1.20 | 2.7E-01 | 4 | 6.3E-01 |
| Pubgwas | Liver PDFF | Hypertension | Outlier-corrected | 0.024 | 0.00 | 4.79 | 8.7E-03 | 4 | 9.2E-02 |
| Pubgwas | Liver PDFF | Coronary artery disease | Raw | -0.092 | 0.10 | -0.91 | 3.9E-01 | 2 | 7.5E-01 |
| Pubgwas | Liver PDFF | Coronary artery disease | Outlier-corrected | -0.075 | 0.05 | -1.44 | 2.1E-01 | 2 | 5.8E-01 |
| Pubgwas | Liver PDFF | Stroke | Raw | -0.038 | 0.03 | -1.10 | 3.1E-01 | NA | NA |
| Pubgwas | Liver PDFF | Stroke | Outlier-corrected | NA | NA | NA | NA | NA | NA |
| Pubgwas | Liver PDFF | Myocardial infarction | Raw | -0.100 | 0.14 | -0.70 | 5.0E-01 | 4 | 8.0E-01 |
| Pubgwas | Liver PDFF | Myocardial infarction | Outlier-corrected | -0.109 | 0.04 | -2.44 | 7.1E-02 | 4 | 3.5E-01 |
| Pubgwas | Liver PDFF | Aortic aneurysm | Raw | 0.084 | 0.10 | 0.83 | 4.3E-01 | NA | NA |
| Pubgwas | Liver PDFF | Aortic aneurysm | Outlier-corrected | NA | NA | NA | NA | NA | NA |
| Pubgwas | Liver PDFF | Heart failure | Raw | -0.016 | 0.05 | -0.31 | 7.6E-01 | 2 | 9.2E-01 |
| Pubgwas | Liver PDFF | Heart failure | Outlier-corrected | 0.012 | 0.05 | 0.24 | 8.2E-01 | 2 | 9.3E-01 |
| Pubgwas | Liver PDFF | Atrial fibrillation | Raw | -0.025 | 0.03 | -0.93 | 3.8E-01 | NA | NA |
| Pubgwas | Liver PDFF | Atrial fibrillation | Outlier-corrected | NA | NA | NA | NA | NA | NA |
| Pubgwas | Liver PDFF | Peripheral artery disease | Raw | -0.166 | 0.05 | -3.38 | 9.7E-03 | NA | NA |
| Pubgwas | Liver PDFF | Peripheral artery disease | Outlier-corrected | NA | NA | NA | NA | NA | NA |
| Pubgwas | Liver PDFF | Deep vein thrombosis | Raw | -0.002 | 0.00 | -2.15 | 6.4E-02 | NA | NA |
| Pubgwas | Liver PDFF | Deep vein thrombosis | Outlier-corrected | NA | NA | NA | NA | NA | NA |
| Pubgwas | Liver PDFF | Pulmonary embolism | Raw | -0.025 | 0.12 | -0.22 | 8.3E-01 | NA | NA |
| Pubgwas | Liver PDFF | Pulmonary embolism | Outlier-corrected | NA | NA | NA | NA | NA | NA |
| Pubgwas | Liver PDFF | Gout | Raw | -0.152 | 0.32 | -0.48 | 6.6E-01 | NA | NA |
| Pubgwas | Liver PDFF | Gout | Outlier-corrected | NA | NA | NA | NA | NA | NA |
| Pubgwas | Liver PDFF | Knee osteoarthritis | Raw | -0.004 | 0.05 | -0.09 | 9.3E-01 | NA | NA |
| Pubgwas | Liver PDFF | Knee osteoarthritis | Outlier-corrected | NA | NA | NA | NA | NA | NA |
| Pubgwas | Liver PDFF | Hip osteoarthritis | Raw | -0.010 | 0.06 | -0.18 | 8.6E-01 | NA | NA |
| Pubgwas | Liver PDFF | Hip osteoarthritis | Outlier-corrected | NA | NA | NA | NA | NA | NA |
| Pubgwas | Liver PDFF | Rheumatoid arthritis | Raw | 0.014 | 0.03 | 0.42 | 6.8E-01 | NA | NA |
| Pubgwas | Liver PDFF | Rheumatoid arthritis | Outlier-corrected | NA | NA | NA | NA | NA | NA |
| Pubgwas | Liver PDFF | Osteoporosis | Raw | 0.003 | 0.00 | 3.00 | 1.7E-02 | NA | NA |
| Pubgwas | Liver PDFF | Osteoporosis | Outlier-corrected | NA | NA | NA | NA | NA | NA |
| Pubgwas | Liver PDFF | Asthma | Raw | 0.003 | 0.04 | 0.07 | 9.5E-01 | 1 | 9.8E-01 |
| Pubgwas | Liver PDFF | Asthma | Outlier-corrected | -0.021 | 0.03 | -0.72 | 4.9E-01 | 1 | 8.0E-01 |
| Pubgwas | Liver PDFF | Psoriasis | Raw | 0.081 | 0.05 | 1.49 | 1.8E-01 | NA | NA |
| Pubgwas | Liver PDFF | Psoriasis | Outlier-corrected | NA | NA | NA | NA | NA | NA |
| Pubgwas | Liver PDFF | Depression | Raw | -0.021 | 0.05 | -0.46 | 6.6E-01 | NA | NA |
| Pubgwas | Liver PDFF | Depression | Outlier-corrected | NA | NA | NA | NA | NA | NA |
| Pubgwas | Liver PDFF | Parkinson's disease | Raw | -0.098 | 0.11 | -0.87 | 4.1E-01 | 1 | 7.6E-01 |
| Pubgwas | Liver PDFF | Parkinson's disease | Outlier-corrected | -0.056 | 0.09 | -0.64 | 5.4E-01 | 1 | 8.3E-01 |
| Pubgwas | Liver PDFF | Alzheimer’s disease | Raw | 0.013 | 0.05 | 0.26 | 8.0E-01 | NA | NA |
| Pubgwas | Liver PDFF | Alzheimer’s disease | Outlier-corrected | NA | NA | NA | NA | NA | NA |
| Pubgwas | Liver PDFF | Cholelithiasis | Raw | -0.141 | 0.12 | -1.22 | 2.6E-01 | 5 | 6.2E-01 |
| Pubgwas | Liver PDFF | Cholelithiasis | Outlier-corrected | 0.014 | 0.06 | 0.25 | 8.2E-01 | 5 | 9.3E-01 |
| Pubgwas | Liver PDFF | Gastroesophageal reflux disease | Raw | -0.024 | 0.03 | -0.88 | 4.3E-01 | NA | NA |
| Pubgwas | Liver PDFF | Gastroesophageal reflux disease | Outlier-corrected | NA | NA | NA | NA | NA | NA |
| Pubgwas | Pancreas PDFF | Type 2 diabetes | Raw | -0.265 | 0.14 | -1.84 | 9.8E-02 | 3 | 4.0E-01 |
| Pubgwas | Pancreas PDFF | Type 2 diabetes | Outlier-corrected | -0.269 | 0.11 | -2.37 | 5.6E-02 | 3 | 3.3E-01 |
| Pubgwas | Pancreas PDFF | Polycystic ovary syndrome | Raw | -0.056 | 0.16 | -0.35 | 7.3E-01 | NA | NA |
| Pubgwas | Pancreas PDFF | Polycystic ovary syndrome | Outlier-corrected | NA | NA | NA | NA | NA | NA |
| Pubgwas | Pancreas PDFF | MASLD | Raw | -0.016 | 0.12 | -0.14 | 8.9E-01 | NA | NA |
| Pubgwas | Pancreas PDFF | MASLD | Outlier-corrected | NA | NA | NA | NA | NA | NA |
| Pubgwas | Pancreas PDFF | Chronic kidney disease | Raw | -0.038 | 0.09 | -0.43 | 6.8E-01 | 1 | 8.8E-01 |
| Pubgwas | Pancreas PDFF | Chronic kidney disease | Outlier-corrected | 0.048 | 0.08 | 0.58 | 5.8E-01 | 1 | 8.4E-01 |
| Pubgwas | Pancreas PDFF | Hypertension | Raw | -0.011 | 0.02 | -0.70 | 5.0E-01 | 4 | 8.0E-01 |
| Pubgwas | Pancreas PDFF | Hypertension | Outlier-corrected | 0.003 | 0.01 | 0.44 | 6.7E-01 | 4 | 8.8E-01 |
| Pubgwas | Pancreas PDFF | Coronary artery disease | Raw | -0.105 | 0.07 | -1.40 | 1.9E-01 | NA | NA |
| Pubgwas | Pancreas PDFF | Coronary artery disease | Outlier-corrected | NA | NA | NA | NA | NA | NA |
| Pubgwas | Pancreas PDFF | Stroke | Raw | 0.064 | 0.05 | 1.25 | 2.4E-01 | NA | NA |
| Pubgwas | Pancreas PDFF | Stroke | Outlier-corrected | NA | NA | NA | NA | NA | NA |
| Pubgwas | Pancreas PDFF | Myocardial infarction | Raw | 0.108 | 0.10 | 1.05 | 3.2E-01 | 1 | 6.6E-01 |
| Pubgwas | Pancreas PDFF | Myocardial infarction | Outlier-corrected | 0.043 | 0.09 | 0.50 | 6.3E-01 | 1 | 8.7E-01 |
| Pubgwas | Pancreas PDFF | Aortic aneurysm | Raw | 0.119 | 0.13 | 0.93 | 3.7E-01 | NA | NA |
| Pubgwas | Pancreas PDFF | Aortic aneurysm | Outlier-corrected | NA | NA | NA | NA | NA | NA |
| Pubgwas | Pancreas PDFF | Heart failure | Raw | 0.060 | 0.10 | 0.63 | 5.4E-01 | 2 | 8.3E-01 |
| Pubgwas | Pancreas PDFF | Heart failure | Outlier-corrected | 0.020 | 0.06 | 0.34 | 7.4E-01 | 2 | 9.2E-01 |
| Pubgwas | Pancreas PDFF | Atrial fibrillation | Raw | 0.098 | 0.06 | 1.65 | 1.2E-01 | 1 | 4.6E-01 |
| Pubgwas | Pancreas PDFF | Atrial fibrillation | Outlier-corrected | 0.058 | 0.05 | 1.19 | 2.6E-01 | 1 | 6.2E-01 |
| Pubgwas | Pancreas PDFF | Peripheral artery disease | Raw | -0.063 | 0.13 | -0.48 | 6.4E-01 | 2 | 8.7E-01 |
| Pubgwas | Pancreas PDFF | Peripheral artery disease | Outlier-corrected | -0.222 | 0.06 | -3.99 | 2.6E-03 | 2 | 4.1E-02 |
| Pubgwas | Pancreas PDFF | Deep vein thrombosis | Raw | 0.011 | 0.01 | 1.37 | 2.0E-01 | 5 | 5.8E-01 |
| Pubgwas | Pancreas PDFF | Deep vein thrombosis | Outlier-corrected | 0.008 | 0.00 | 2.91 | 2.3E-02 | 5 | 1.8E-01 |
| Pubgwas | Pancreas PDFF | Pulmonary embolism | Raw | 0.708 | 0.50 | 1.42 | 1.8E-01 | 2 | 5.6E-01 |
| Pubgwas | Pancreas PDFF | Pulmonary embolism | Outlier-corrected | 0.354 | 0.17 | 2.09 | 6.3E-02 | 2 | 3.4E-01 |
| Pubgwas | Pancreas PDFF | Gout | Raw | -0.506 | 0.13 | -3.93 | 1.1E-02 | NA | NA |
| Pubgwas | Pancreas PDFF | Gout | Outlier-corrected | NA | NA | NA | NA | NA | NA |
| Pubgwas | Pancreas PDFF | Knee osteoarthritis | Raw | 0.093 | 0.06 | 1.44 | 1.7E-01 | NA | NA |
| Pubgwas | Pancreas PDFF | Knee osteoarthritis | Outlier-corrected | NA | NA | NA | NA | NA | NA |
| Pubgwas | Pancreas PDFF | Hip osteoarthritis | Raw | -0.122 | 0.07 | -1.73 | 1.1E-01 | NA | NA |
| Pubgwas | Pancreas PDFF | Hip osteoarthritis | Outlier-corrected | NA | NA | NA | NA | NA | NA |
| Pubgwas | Pancreas PDFF | Rheumatoid arthritis | Raw | -0.001 | 0.11 | -0.01 | 9.9E-01 | NA | NA |
| Pubgwas | Pancreas PDFF | Rheumatoid arthritis | Outlier-corrected | NA | NA | NA | NA | NA | NA |
| Pubgwas | Pancreas PDFF | Osteoporosis | Raw | -0.001 | 0.00 | -0.64 | 5.4E-01 | NA | NA |
| Pubgwas | Pancreas PDFF | Osteoporosis | Outlier-corrected | NA | NA | NA | NA | NA | NA |
| Pubgwas | Pancreas PDFF | Asthma | Raw | 0.020 | 0.07 | 0.30 | 7.7E-01 | 1 | 9.2E-01 |
| Pubgwas | Pancreas PDFF | Asthma | Outlier-corrected | 0.067 | 0.06 | 1.13 | 2.8E-01 | 1 | 6.4E-01 |
| Pubgwas | Pancreas PDFF | Psoriasis | Raw | 0.230 | 0.19 | 1.22 | 2.5E-01 | 2 | 6.2E-01 |
| Pubgwas | Pancreas PDFF | Psoriasis | Outlier-corrected | 0.231 | 0.11 | 2.18 | 6.1E-02 | 2 | 3.4E-01 |
| Pubgwas | Pancreas PDFF | Depression | Raw | -0.017 | 0.07 | -0.25 | 8.1E-01 | NA | NA |
| Pubgwas | Pancreas PDFF | Depression | Outlier-corrected | NA | NA | NA | NA | NA | NA |
| Pubgwas | Pancreas PDFF | Parkinson's disease | Raw | -0.060 | 0.11 | -0.57 | 5.8E-01 | NA | NA |
| Pubgwas | Pancreas PDFF | Parkinson's disease | Outlier-corrected | NA | NA | NA | NA | NA | NA |
| Pubgwas | Pancreas PDFF | Alzheimer’s disease | Raw | 0.032 | 0.05 | 0.62 | 5.5E-01 | NA | NA |
| Pubgwas | Pancreas PDFF | Alzheimer’s disease | Outlier-corrected | NA | NA | NA | NA | NA | NA |
| Pubgwas | Pancreas PDFF | Cholelithiasis | Raw | 0.126 | 0.13 | 1.01 | 3.3E-01 | 2 | 6.9E-01 |
| Pubgwas | Pancreas PDFF | Cholelithiasis | Outlier-corrected | -0.025 | 0.07 | -0.36 | 7.2E-01 | 2 | 9.1E-01 |
| Pubgwas | Pancreas PDFF | Gastroesophageal reflux disease | Raw | 0.041 | 0.06 | 0.73 | 5.1E-01 | NA | NA |
| Pubgwas | Pancreas PDFF | Gastroesophageal reflux disease | Outlier-corrected | NA | NA | NA | NA | NA | NA |
| Pubgwas | Paraspinal AT | Type 2 diabetes | Raw | -0.114 | 0.33 | -0.35 | 7.4E-01 | 3 | 9.2E-01 |
| Pubgwas | Paraspinal AT | Type 2 diabetes | Outlier-corrected | -0.112 | 0.07 | -1.71 | 1.6E-01 | 3 | 5.2E-01 |
| Pubgwas | Paraspinal AT | Polycystic ovary syndrome | Raw | -0.349 | 0.24 | -1.44 | 1.9E-01 | 1 | 5.7E-01 |
| Pubgwas | Paraspinal AT | Polycystic ovary syndrome | Outlier-corrected | -0.123 | 0.21 | -0.60 | 5.7E-01 | 1 | 8.4E-01 |
| Pubgwas | Paraspinal AT | MASLD | Raw | -0.146 | 0.18 | -0.79 | 4.5E-01 | 2 | 7.9E-01 |
| Pubgwas | Paraspinal AT | MASLD | Outlier-corrected | -0.180 | 0.08 | -2.20 | 7.9E-02 | 2 | 3.6E-01 |
| Pubgwas | Paraspinal AT | Chronic kidney disease | Raw | 0.003 | 0.09 | 0.03 | 9.8E-01 | NA | NA |
| Pubgwas | Paraspinal AT | Chronic kidney disease | Outlier-corrected | NA | NA | NA | NA | NA | NA |
| Pubgwas | Paraspinal AT | Hypertension | Raw | -0.012 | 0.01 | -0.88 | 4.1E-01 | 2 | 7.6E-01 |
| Pubgwas | Paraspinal AT | Hypertension | Outlier-corrected | -0.015 | 0.01 | -1.30 | 2.5E-01 | 2 | 6.2E-01 |
| Pubgwas | Paraspinal AT | Coronary artery disease | Raw | -0.155 | 0.09 | -1.73 | 1.3E-01 | 1 | 4.6E-01 |
| Pubgwas | Paraspinal AT | Coronary artery disease | Outlier-corrected | -0.216 | 0.05 | -4.01 | 7.0E-03 | 1 | 8.6E-02 |
| Pubgwas | Paraspinal AT | Stroke | Raw | 0.084 | 0.10 | 0.85 | 4.2E-01 | 1 | 7.8E-01 |
| Pubgwas | Paraspinal AT | Stroke | Outlier-corrected | 0.141 | 0.07 | 2.06 | 8.5E-02 | 1 | 3.8E-01 |
| Pubgwas | Paraspinal AT | Myocardial infarction | Raw | -0.106 | 0.07 | -1.43 | 2.0E-01 | NA | NA |
| Pubgwas | Paraspinal AT | Myocardial infarction | Outlier-corrected | NA | NA | NA | NA | NA | NA |
| Pubgwas | Paraspinal AT | Aortic aneurysm | Raw | -0.203 | 0.11 | -1.88 | 1.0E-01 | NA | NA |
| Pubgwas | Paraspinal AT | Aortic aneurysm | Outlier-corrected | NA | NA | NA | NA | NA | NA |
| Pubgwas | Paraspinal AT | Heart failure | Raw | -0.005 | 0.11 | -0.04 | 9.7E-01 | 1 | 9.8E-01 |
| Pubgwas | Paraspinal AT | Heart failure | Outlier-corrected | -0.084 | 0.05 | -1.72 | 1.4E-01 | 1 | 4.6E-01 |
| Pubgwas | Paraspinal AT | Atrial fibrillation | Raw | 0.150 | 0.08 | 1.99 | 8.7E-02 | 1 | 3.8E-01 |
| Pubgwas | Paraspinal AT | Atrial fibrillation | Outlier-corrected | 0.107 | 0.06 | 1.76 | 1.3E-01 | 1 | 4.6E-01 |
| Pubgwas | Paraspinal AT | Peripheral artery disease | Raw | -0.111 | 0.14 | -0.78 | 4.6E-01 | 1 | 7.9E-01 |
| Pubgwas | Paraspinal AT | Peripheral artery disease | Outlier-corrected | -0.189 | 0.13 | -1.44 | 2.0E-01 | 1 | 5.8E-01 |
| Pubgwas | Paraspinal AT | Deep vein thrombosis | Raw | 0.002 | 0.00 | 0.55 | 6.0E-01 | 2 | 8.5E-01 |
| Pubgwas | Paraspinal AT | Deep vein thrombosis | Outlier-corrected | 0.002 | 0.00 | 0.98 | 3.7E-01 | 2 | 7.4E-01 |
| Pubgwas | Paraspinal AT | Pulmonary embolism | Raw | 0.181 | 0.15 | 1.23 | 2.6E-01 | NA | NA |
| Pubgwas | Paraspinal AT | Pulmonary embolism | Outlier-corrected | NA | NA | NA | NA | NA | NA |
| Pubgwas | Paraspinal AT | Gout | Raw | 0.502 | 0.39 | 1.28 | 2.6E-01 | NA | NA |
| Pubgwas | Paraspinal AT | Gout | Outlier-corrected | NA | NA | NA | NA | NA | NA |
| Pubgwas | Paraspinal AT | Knee osteoarthritis | Raw | 0.048 | 0.11 | 0.42 | 6.9E-01 | 1 | 8.8E-01 |
| Pubgwas | Paraspinal AT | Knee osteoarthritis | Outlier-corrected | -0.025 | 0.08 | -0.31 | 7.7E-01 | 1 | 9.2E-01 |
| Pubgwas | Paraspinal AT | Hip osteoarthritis | Raw | 0.090 | 0.14 | 0.66 | 5.3E-01 | 1 | 8.3E-01 |
| Pubgwas | Paraspinal AT | Hip osteoarthritis | Outlier-corrected | -0.003 | 0.08 | -0.03 | 9.7E-01 | 1 | 9.8E-01 |
| Pubgwas | Paraspinal AT | Rheumatoid arthritis | Raw | -0.105 | 0.11 | -0.92 | 3.9E-01 | NA | NA |
| Pubgwas | Paraspinal AT | Rheumatoid arthritis | Outlier-corrected | NA | NA | NA | NA | NA | NA |
| Pubgwas | Paraspinal AT | Osteoporosis | Raw | 0.000 | 0.00 | 0.02 | 9.9E-01 | NA | NA |
| Pubgwas | Paraspinal AT | Osteoporosis | Outlier-corrected | NA | NA | NA | NA | NA | NA |
| Pubgwas | Paraspinal AT | Asthma | Raw | 0.020 | 0.04 | 0.53 | 6.1E-01 | NA | NA |
| Pubgwas | Paraspinal AT | Asthma | Outlier-corrected | NA | NA | NA | NA | NA | NA |
| Pubgwas | Paraspinal AT | Psoriasis | Raw | 0.029 | 0.19 | 0.15 | 8.9E-01 | 1 | 9.6E-01 |
| Pubgwas | Paraspinal AT | Psoriasis | Outlier-corrected | -0.141 | 0.09 | -1.62 | 1.6E-01 | 1 | 5.1E-01 |
| Pubgwas | Paraspinal AT | Depression | Raw | -0.024 | 0.06 | -0.39 | 7.1E-01 | NA | NA |
| Pubgwas | Paraspinal AT | Depression | Outlier-corrected | NA | NA | NA | NA | NA | NA |
| Pubgwas | Paraspinal AT | Parkinson's disease | Raw | 0.003 | 0.11 | 0.02 | 9.8E-01 | NA | NA |
| Pubgwas | Paraspinal AT | Parkinson's disease | Outlier-corrected | NA | NA | NA | NA | NA | NA |
| Pubgwas | Paraspinal AT | Alzheimer’s disease | Raw | -0.091 | 0.06 | -1.65 | 1.4E-01 | NA | NA |
| Pubgwas | Paraspinal AT | Alzheimer’s disease | Outlier-corrected | NA | NA | NA | NA | NA | NA |
| Pubgwas | Paraspinal AT | Cholelithiasis | Raw | 0.105 | 0.09 | 1.12 | 3.0E-01 | 1 | 6.5E-01 |
| Pubgwas | Paraspinal AT | Cholelithiasis | Outlier-corrected | 0.037 | 0.05 | 0.77 | 4.7E-01 | 1 | 7.9E-01 |
| Pubgwas | Paraspinal AT | Gastroesophageal reflux disease | Raw | 0.182 | 0.09 | 1.99 | 1.0E-01 | 1 | 4.0E-01 |
| Pubgwas | Paraspinal AT | Gastroesophageal reflux disease | Outlier-corrected | 0.103 | 0.04 | 2.84 | 4.7E-02 | 1 | 3.0E-01 |
| Pubgwas | Pelvic bone marrow fat | Type 2 diabetes | Raw | -0.047 | 0.06 | -0.83 | 4.1E-01 | 6 | 7.7E-01 |
| Pubgwas | Pelvic bone marrow fat | Type 2 diabetes | Outlier-corrected | -0.034 | 0.03 | -1.18 | 2.5E-01 | 6 | 6.2E-01 |
| Pubgwas | Pelvic bone marrow fat | Polycystic ovary syndrome | Raw | 0.127 | 0.12 | 1.04 | 3.1E-01 | 1 | 6.6E-01 |
| Pubgwas | Pelvic bone marrow fat | Polycystic ovary syndrome | Outlier-corrected | 0.176 | 0.11 | 1.56 | 1.3E-01 | 1 | 4.6E-01 |
| Pubgwas | Pelvic bone marrow fat | MASLD | Raw | -0.052 | 0.07 | -0.78 | 4.4E-01 | NA | NA |
| Pubgwas | Pelvic bone marrow fat | MASLD | Outlier-corrected | NA | NA | NA | NA | NA | NA |
| Pubgwas | Pelvic bone marrow fat | Chronic kidney disease | Raw | -0.026 | 0.05 | -0.56 | 5.8E-01 | 1 | 8.4E-01 |
| Pubgwas | Pelvic bone marrow fat | Chronic kidney disease | Outlier-corrected | 0.006 | 0.04 | 0.14 | 8.9E-01 | 1 | 9.6E-01 |
| Pubgwas | Pelvic bone marrow fat | Hypertension | Raw | -0.005 | 0.01 | -0.48 | 6.4E-01 | 5 | 8.7E-01 |
| Pubgwas | Pelvic bone marrow fat | Hypertension | Outlier-corrected | -0.001 | 0.01 | -0.23 | 8.2E-01 | 5 | 9.3E-01 |
| Pubgwas | Pelvic bone marrow fat | Coronary artery disease | Raw | -0.050 | 0.06 | -0.78 | 4.4E-01 | 1 | 7.9E-01 |
| Pubgwas | Pelvic bone marrow fat | Coronary artery disease | Outlier-corrected | -0.008 | 0.05 | -0.17 | 8.7E-01 | 1 | 9.6E-01 |
| Pubgwas | Pelvic bone marrow fat | Stroke | Raw | -0.060 | 0.06 | -0.95 | 3.5E-01 | 1 | 7.1E-01 |
| Pubgwas | Pelvic bone marrow fat | Stroke | Outlier-corrected | -0.002 | 0.04 | -0.06 | 9.5E-01 | 1 | 9.8E-01 |
| Pubgwas | Pelvic bone marrow fat | Myocardial infarction | Raw | -0.043 | 0.08 | -0.55 | 5.8E-01 | 2 | 8.4E-01 |
| Pubgwas | Pelvic bone marrow fat | Myocardial infarction | Outlier-corrected | 0.003 | 0.05 | 0.06 | 9.5E-01 | 2 | 9.8E-01 |
| Pubgwas | Pelvic bone marrow fat | Aortic aneurysm | Raw | -0.151 | 0.09 | -1.69 | 1.0E-01 | NA | NA |
| Pubgwas | Pelvic bone marrow fat | Aortic aneurysm | Outlier-corrected | NA | NA | NA | NA | NA | NA |
| Pubgwas | Pelvic bone marrow fat | Heart failure | Raw | -0.086 | 0.05 | -1.85 | 7.5E-02 | 1 | 3.6E-01 |
| Pubgwas | Pelvic bone marrow fat | Heart failure | Outlier-corrected | -0.053 | 0.04 | -1.39 | 1.8E-01 | 1 | 5.6E-01 |
| Pubgwas | Pelvic bone marrow fat | Atrial fibrillation | Raw | -0.019 | 0.05 | -0.43 | 6.7E-01 | 3 | 8.8E-01 |
| Pubgwas | Pelvic bone marrow fat | Atrial fibrillation | Outlier-corrected | -0.027 | 0.04 | -0.72 | 4.8E-01 | 3 | 7.9E-01 |
| Pubgwas | Pelvic bone marrow fat | Peripheral artery disease | Raw | 0.009 | 0.06 | 0.13 | 8.9E-01 | NA | NA |
| Pubgwas | Pelvic bone marrow fat | Peripheral artery disease | Outlier-corrected | NA | NA | NA | NA | NA | NA |
| Pubgwas | Pelvic bone marrow fat | Deep vein thrombosis | Raw | -0.001 | 0.00 | -0.68 | 5.0E-01 | 1 | 8.0E-01 |
| Pubgwas | Pelvic bone marrow fat | Deep vein thrombosis | Outlier-corrected | 0.000 | 0.00 | 0.02 | 9.8E-01 | 1 | 9.9E-01 |
| Pubgwas | Pelvic bone marrow fat | Pulmonary embolism | Raw | -0.027 | 0.10 | -0.27 | 7.9E-01 | NA | NA |
| Pubgwas | Pelvic bone marrow fat | Pulmonary embolism | Outlier-corrected | NA | NA | NA | NA | NA | NA |
| Pubgwas | Pelvic bone marrow fat | Gout | Raw | -0.164 | 0.12 | -1.37 | 1.9E-01 | NA | NA |
| Pubgwas | Pelvic bone marrow fat | Gout | Outlier-corrected | NA | NA | NA | NA | NA | NA |
| Pubgwas | Pelvic bone marrow fat | Knee osteoarthritis | Raw | -0.137 | 0.08 | -1.73 | 9.5E-02 | 2 | 3.9E-01 |
| Pubgwas | Pelvic bone marrow fat | Knee osteoarthritis | Outlier-corrected | -0.063 | 0.05 | -1.29 | 2.1E-01 | 2 | 5.8E-01 |
| Pubgwas | Pelvic bone marrow fat | Hip osteoarthritis | Raw | -0.155 | 0.07 | -2.17 | 3.9E-02 | 1 | 2.9E-01 |
| Pubgwas | Pelvic bone marrow fat | Hip osteoarthritis | Outlier-corrected | -0.120 | 0.06 | -1.88 | 7.1E-02 | 1 | 3.5E-01 |
| Pubgwas | Pelvic bone marrow fat | Rheumatoid arthritis | Raw | -0.033 | 0.14 | -0.23 | 8.2E-01 | 3 | 9.3E-01 |
| Pubgwas | Pelvic bone marrow fat | Rheumatoid arthritis | Outlier-corrected | -0.009 | 0.05 | -0.19 | 8.5E-01 | 3 | 9.4E-01 |
| Pubgwas | Pelvic bone marrow fat | Osteoporosis | Raw | 0.007 | 0.00 | 2.56 | 1.6E-02 | 7 | 1.5E-01 |
| Pubgwas | Pelvic bone marrow fat | Osteoporosis | Outlier-corrected | 0.002 | 0.00 | 0.90 | 3.8E-01 | 7 | 7.4E-01 |
| Pubgwas | Pelvic bone marrow fat | Asthma | Raw | -0.011 | 0.06 | -0.19 | 8.5E-01 | 3 | 9.4E-01 |
| Pubgwas | Pelvic bone marrow fat | Asthma | Outlier-corrected | 0.038 | 0.03 | 1.24 | 2.3E-01 | 3 | 6.0E-01 |
| Pubgwas | Pelvic bone marrow fat | Psoriasis | Raw | -0.279 | 0.31 | -0.89 | 3.8E-01 | 3 | 7.4E-01 |
| Pubgwas | Pelvic bone marrow fat | Psoriasis | Outlier-corrected | 0.004 | 0.08 | 0.05 | 9.6E-01 | 3 | 9.8E-01 |
| Pubgwas | Pelvic bone marrow fat | Depression | Raw | -0.072 | 0.06 | -1.30 | 2.1E-01 | NA | NA |
| Pubgwas | Pelvic bone marrow fat | Depression | Outlier-corrected | NA | NA | NA | NA | NA | NA |
| Pubgwas | Pelvic bone marrow fat | Parkinson's disease | Raw | -0.104 | 0.06 | -1.68 | 1.0E-01 | NA | NA |
| Pubgwas | Pelvic bone marrow fat | Parkinson's disease | Outlier-corrected | NA | NA | NA | NA | NA | NA |
| Pubgwas | Pelvic bone marrow fat | Alzheimer’s disease | Raw | 0.071 | 0.04 | 1.70 | 1.0E-01 | 0 | NA |
| Pubgwas | Pelvic bone marrow fat | Alzheimer’s disease | Outlier-corrected | NA | NA | NA | NA | 0 | NA |
| Pubgwas | Pelvic bone marrow fat | Cholelithiasis | Raw | -0.088 | 0.04 | -2.09 | 4.6E-02 | 1 | 3.0E-01 |
| Pubgwas | Pelvic bone marrow fat | Cholelithiasis | Outlier-corrected | -0.061 | 0.03 | -1.81 | 8.1E-02 | 1 | 3.6E-01 |
| Pubgwas | Pelvic bone marrow fat | Gastroesophageal reflux disease | Raw | 0.005 | 0.03 | 0.15 | 8.8E-01 | 2 | 9.6E-01 |
| Pubgwas | Pelvic bone marrow fat | Gastroesophageal reflux disease | Outlier-corrected | 0.039 | 0.03 | 1.47 | 1.6E-01 | 2 | 5.2E-01 |
| Pubgwas | Thigh bone marrow fat | Type 2 diabetes | Raw | -0.146 | 0.12 | -1.27 | 2.2E-01 | 4 | 5.9E-01 |
| Pubgwas | Thigh bone marrow fat | Type 2 diabetes | Outlier-corrected | -0.052 | 0.03 | -1.60 | 1.2E-01 | 4 | 4.6E-01 |
| Pubgwas | Thigh bone marrow fat | Polycystic ovary syndrome | Raw | -0.037 | 0.14 | -0.26 | 8.0E-01 | 1 | 9.3E-01 |
| Pubgwas | Thigh bone marrow fat | Polycystic ovary syndrome | Outlier-corrected | 0.062 | 0.13 | 0.49 | 6.3E-01 | 1 | 8.7E-01 |
| Pubgwas | Thigh bone marrow fat | MASLD | Raw | -0.103 | 0.09 | -1.18 | 2.5E-01 | 0 | NA |
| Pubgwas | Thigh bone marrow fat | MASLD | Outlier-corrected | NA | NA | NA | NA | 0 | NA |
| Pubgwas | Thigh bone marrow fat | Chronic kidney disease | Raw | 0.048 | 0.04 | 1.12 | 2.7E-01 | NA | NA |
| Pubgwas | Thigh bone marrow fat | Chronic kidney disease | Outlier-corrected | NA | NA | NA | NA | NA | NA |
| Pubgwas | Thigh bone marrow fat | Hypertension | Raw | 0.005 | 0.01 | 0.54 | 5.9E-01 | 8 | 8.5E-01 |
| Pubgwas | Thigh bone marrow fat | Hypertension | Outlier-corrected | 0.005 | 0.01 | 0.86 | 4.0E-01 | 8 | 7.5E-01 |
| Pubgwas | Thigh bone marrow fat | Coronary artery disease | Raw | -0.022 | 0.05 | -0.45 | 6.6E-01 | NA | NA |
| Pubgwas | Thigh bone marrow fat | Coronary artery disease | Outlier-corrected | NA | NA | NA | NA | NA | NA |
| Pubgwas | Thigh bone marrow fat | Stroke | Raw | -0.006 | 0.04 | -0.15 | 8.8E-01 | NA | NA |
| Pubgwas | Thigh bone marrow fat | Stroke | Outlier-corrected | NA | NA | NA | NA | NA | NA |
| Pubgwas | Thigh bone marrow fat | Myocardial infarction | Raw | -0.060 | 0.06 | -0.96 | 3.5E-01 | 1 | 7.1E-01 |
| Pubgwas | Thigh bone marrow fat | Myocardial infarction | Outlier-corrected | -0.032 | 0.06 | -0.58 | 5.6E-01 | 1 | 8.4E-01 |
| Pubgwas | Thigh bone marrow fat | Aortic aneurysm | Raw | -0.008 | 0.11 | -0.08 | 9.4E-01 | NA | NA |
| Pubgwas | Thigh bone marrow fat | Aortic aneurysm | Outlier-corrected | NA | NA | NA | NA | NA | NA |
| Pubgwas | Thigh bone marrow fat | Heart failure | Raw | 0.023 | 0.04 | 0.57 | 5.7E-01 | NA | NA |
| Pubgwas | Thigh bone marrow fat | Heart failure | Outlier-corrected | NA | NA | NA | NA | NA | NA |
| Pubgwas | Thigh bone marrow fat | Atrial fibrillation | Raw | 0.105 | 0.06 | 1.83 | 7.8E-02 | 3 | 3.6E-01 |
| Pubgwas | Thigh bone marrow fat | Atrial fibrillation | Outlier-corrected | 0.086 | 0.04 | 1.91 | 6.7E-02 | 3 | 3.5E-01 |
| Pubgwas | Thigh bone marrow fat | Peripheral artery disease | Raw | -0.048 | 0.07 | -0.69 | 5.0E-01 | NA | NA |
| Pubgwas | Thigh bone marrow fat | Peripheral artery disease | Outlier-corrected | NA | NA | NA | NA | NA | NA |
| Pubgwas | Thigh bone marrow fat | Deep vein thrombosis | Raw | 0.002 | 0.00 | 1.12 | 2.7E-01 | 1 | 6.3E-01 |
| Pubgwas | Thigh bone marrow fat | Deep vein thrombosis | Outlier-corrected | 0.001 | 0.00 | 0.76 | 4.6E-01 | 1 | 7.9E-01 |
| Pubgwas | Thigh bone marrow fat | Pulmonary embolism | Raw | 0.191 | 0.12 | 1.66 | 1.1E-01 | NA | NA |
| Pubgwas | Thigh bone marrow fat | Pulmonary embolism | Outlier-corrected | NA | NA | NA | NA | NA | NA |
| Pubgwas | Thigh bone marrow fat | Gout | Raw | 0.006 | 0.18 | 0.03 | 9.7E-01 | NA | NA |
| Pubgwas | Thigh bone marrow fat | Gout | Outlier-corrected | NA | NA | NA | NA | NA | NA |
| Pubgwas | Thigh bone marrow fat | Knee osteoarthritis | Raw | -0.219 | 0.06 | -3.92 | 4.9E-04 | 0 | NA |
| Pubgwas | Thigh bone marrow fat | Knee osteoarthritis | Outlier-corrected | NA | NA | NA | NA | 0 | NA |
| Pubgwas | Thigh bone marrow fat | Hip osteoarthritis | Raw | -0.196 | 0.08 | -2.52 | 1.8E-02 | 2 | 1.5E-01 |
| Pubgwas | Thigh bone marrow fat | Hip osteoarthritis | Outlier-corrected | -0.182 | 0.06 | -2.84 | 8.4E-03 | 2 | 9.2E-02 |
| Pubgwas | Thigh bone marrow fat | Rheumatoid arthritis | Raw | 0.015 | 0.07 | 0.22 | 8.3E-01 | NA | NA |
| Pubgwas | Thigh bone marrow fat | Rheumatoid arthritis | Outlier-corrected | NA | NA | NA | NA | NA | NA |
| Pubgwas | Thigh bone marrow fat | Osteoporosis | Raw | 0.011 | 0.00 | 3.96 | 4.4E-04 | 7 | 1.4E-02 |
| Pubgwas | Thigh bone marrow fat | Osteoporosis | Outlier-corrected | 0.007 | 0.00 | 3.35 | 2.9E-03 | 7 | 4.1E-02 |
| Pubgwas | Thigh bone marrow fat | Asthma | Raw | 0.034 | 0.04 | 0.92 | 3.7E-01 | 1 | 7.3E-01 |
| Pubgwas | Thigh bone marrow fat | Asthma | Outlier-corrected | 0.017 | 0.03 | 0.52 | 6.1E-01 | 1 | 8.6E-01 |
| Pubgwas | Thigh bone marrow fat | Psoriasis | Raw | -0.050 | 0.11 | -0.45 | 6.6E-01 | 2 | 8.8E-01 |
| Pubgwas | Thigh bone marrow fat | Psoriasis | Outlier-corrected | -0.068 | 0.10 | -0.71 | 4.9E-01 | 2 | 8.0E-01 |
| Pubgwas | Thigh bone marrow fat | Depression | Raw | -0.008 | 0.07 | -0.12 | 9.0E-01 | 0 | NA |
| Pubgwas | Thigh bone marrow fat | Depression | Outlier-corrected | NA | NA | NA | NA | 0 | NA |
| Pubgwas | Thigh bone marrow fat | Parkinson's disease | Raw | -0.047 | 0.07 | -0.66 | 5.2E-01 | NA | NA |
| Pubgwas | Thigh bone marrow fat | Parkinson's disease | Outlier-corrected | NA | NA | NA | NA | NA | NA |
| Pubgwas | Thigh bone marrow fat | Alzheimer’s disease | Raw | 0.002 | 0.05 | 0.04 | 9.7E-01 | 1 | 9.8E-01 |
| Pubgwas | Thigh bone marrow fat | Alzheimer’s disease | Outlier-corrected | 0.020 | 0.04 | 0.46 | 6.5E-01 | 1 | 8.7E-01 |
| Pubgwas | Thigh bone marrow fat | Cholelithiasis | Raw | 0.001 | 0.07 | 0.01 | 9.9E-01 | 1 | 9.9E-01 |
| Pubgwas | Thigh bone marrow fat | Cholelithiasis | Outlier-corrected | -0.047 | 0.05 | -0.99 | 3.3E-01 | 1 | 6.9E-01 |
| Pubgwas | Thigh bone marrow fat | Gastroesophageal reflux disease | Raw | 0.033 | 0.03 | 1.19 | 2.5E-01 | NA | NA |
| Pubgwas | Thigh bone marrow fat | Gastroesophageal reflux disease | Outlier-corrected | NA | NA | NA | NA | NA | NA |
| Pubgwas | Vertebrae bone marrow fat | Type 2 diabetes | Raw | 0.175 | 0.12 | 1.44 | 2.4E-01 | NA | NA |
| Pubgwas | Vertebrae bone marrow fat | Type 2 diabetes | Outlier-corrected | NA | NA | NA | NA | NA | NA |
| Pubgwas | Vertebrae bone marrow fat | Chronic kidney disease | Raw | 0.028 | 0.04 | 0.71 | 5.3E-01 | NA | NA |
| Pubgwas | Vertebrae bone marrow fat | Chronic kidney disease | Outlier-corrected | NA | NA | NA | NA | NA | NA |
| Pubgwas | Vertebrae bone marrow fat | Coronary artery disease | Raw | 0.087 | 0.05 | 1.62 | 1.8E-01 | NA | NA |
| Pubgwas | Vertebrae bone marrow fat | Coronary artery disease | Outlier-corrected | NA | NA | NA | NA | NA | NA |
| Pubgwas | Vertebrae bone marrow fat | Stroke | Raw | -0.042 | 0.05 | -0.78 | 4.8E-01 | NA | NA |
| Pubgwas | Vertebrae bone marrow fat | Stroke | Outlier-corrected | NA | NA | NA | NA | NA | NA |
| Pubgwas | Vertebrae bone marrow fat | Myocardial infarction | Raw | 0.153 | 0.07 | 2.18 | 9.4E-02 | NA | NA |
| Pubgwas | Vertebrae bone marrow fat | Myocardial infarction | Outlier-corrected | NA | NA | NA | NA | NA | NA |
| Pubgwas | Vertebrae bone marrow fat | Heart failure | Raw | -0.019 | 0.03 | -0.73 | 5.0E-01 | NA | NA |
| Pubgwas | Vertebrae bone marrow fat | Heart failure | Outlier-corrected | NA | NA | NA | NA | NA | NA |
| Pubgwas | Vertebrae bone marrow fat | Atrial fibrillation | Raw | -0.032 | 0.07 | -0.43 | 6.9E-01 | NA | NA |
| Pubgwas | Vertebrae bone marrow fat | Atrial fibrillation | Outlier-corrected | NA | NA | NA | NA | NA | NA |
| Pubgwas | Vertebrae bone marrow fat | Knee osteoarthritis | Raw | 0.077 | 0.12 | 0.64 | 5.6E-01 | NA | NA |
| Pubgwas | Vertebrae bone marrow fat | Knee osteoarthritis | Outlier-corrected | NA | NA | NA | NA | NA | NA |
| Pubgwas | Vertebrae bone marrow fat | Hip osteoarthritis | Raw | 0.241 | 0.11 | 2.21 | 9.1E-02 | NA | NA |
| Pubgwas | Vertebrae bone marrow fat | Hip osteoarthritis | Outlier-corrected | NA | NA | NA | NA | NA | NA |


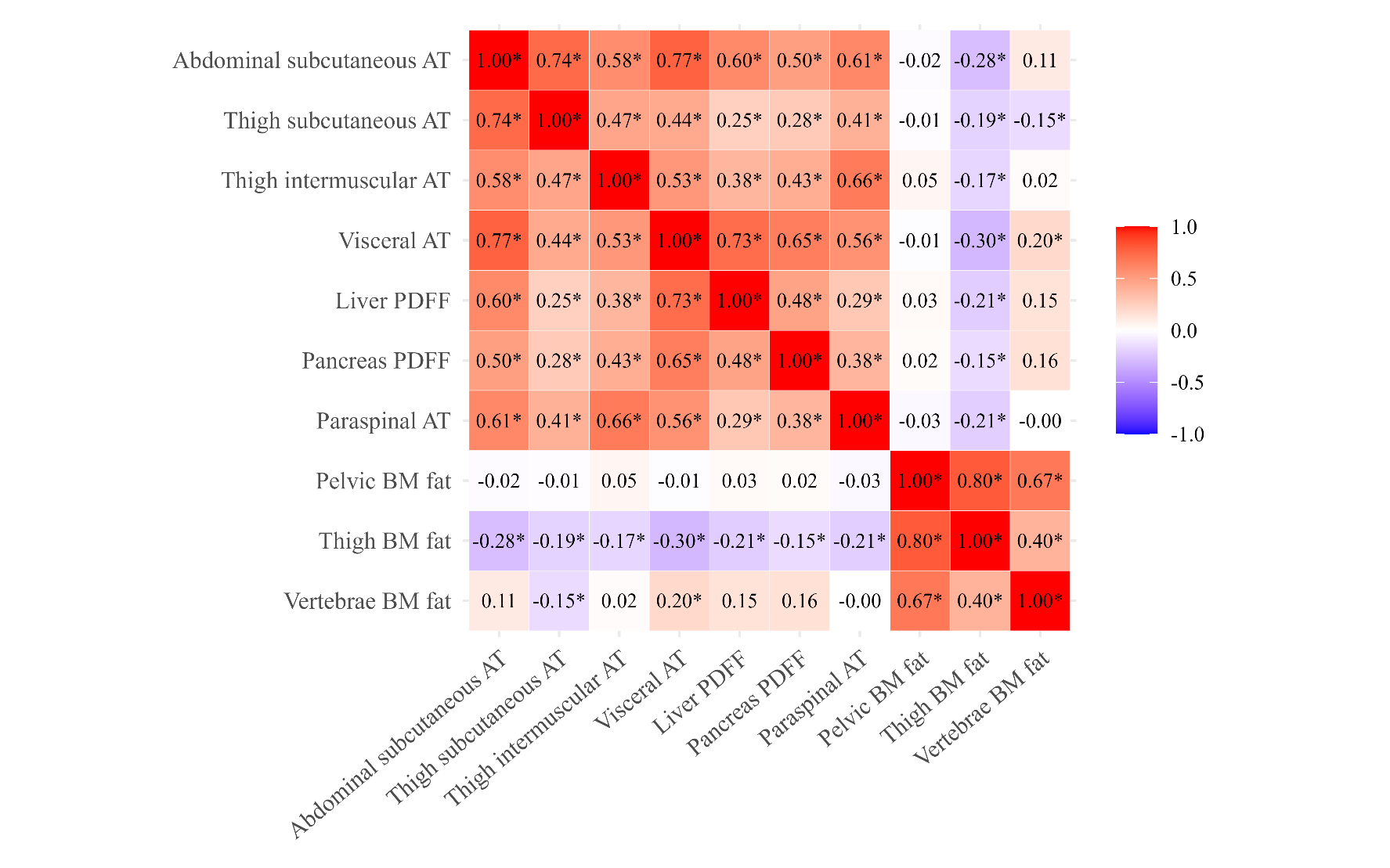


**Supplementary Figure 1. Genetic correlation between IDPs.** The colour and intensity represent the magnitude and direction of correlation. The labels represent genetic correlation (rg) and the asterisk represents statistical significance (p< 0.05).


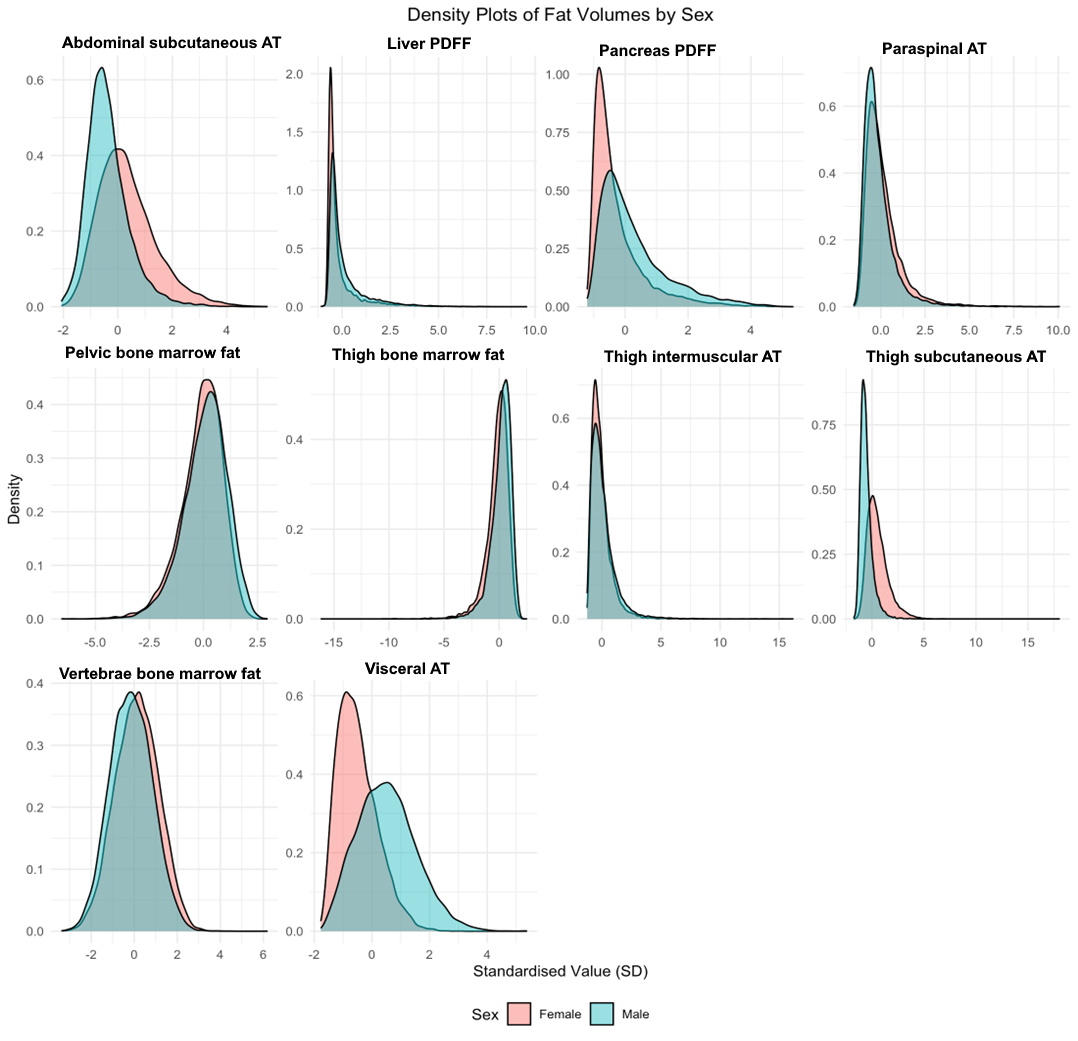


**Supplementary Figure 2. The difference in fat volume across the ten fat depots stratified by sex.** The blue curve represents the distribution of fat volume in males and the pink in famales.


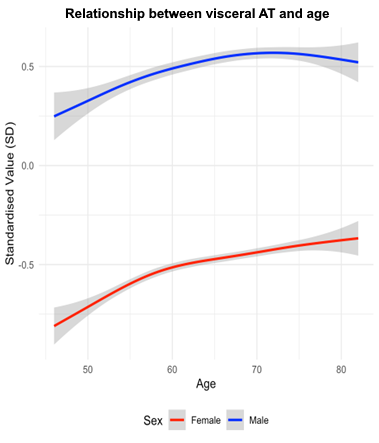

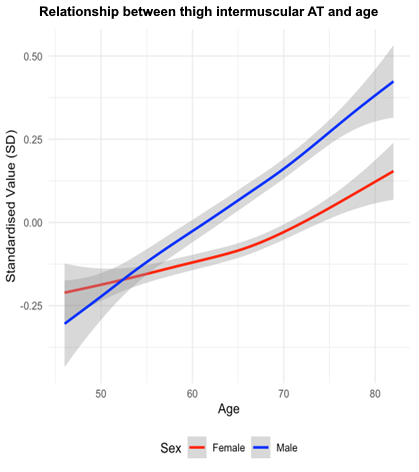

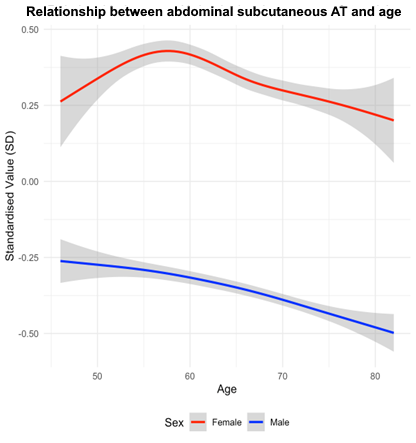

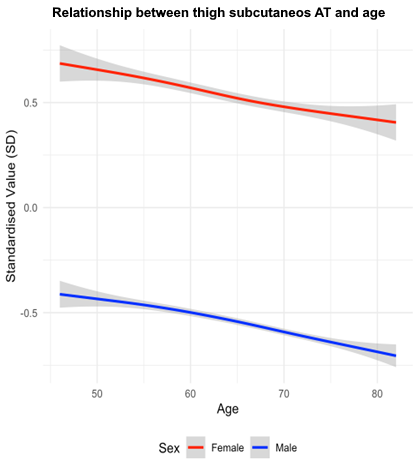

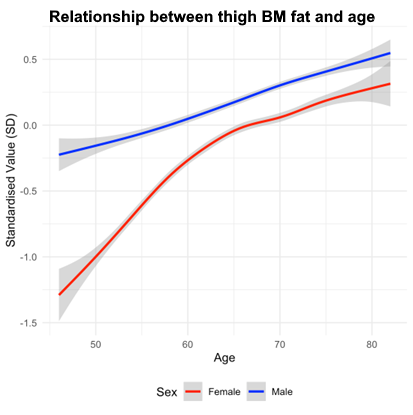

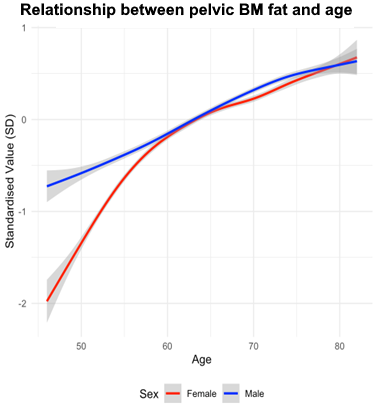

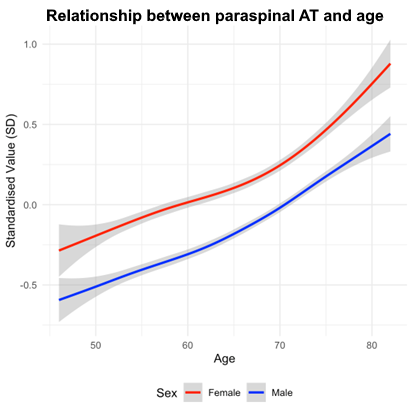

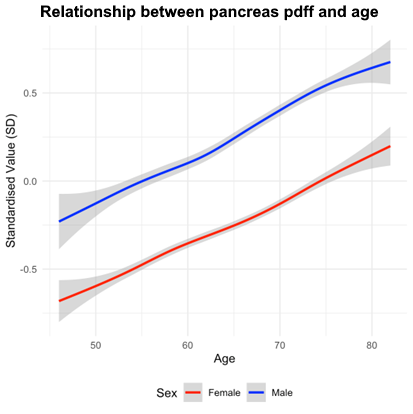

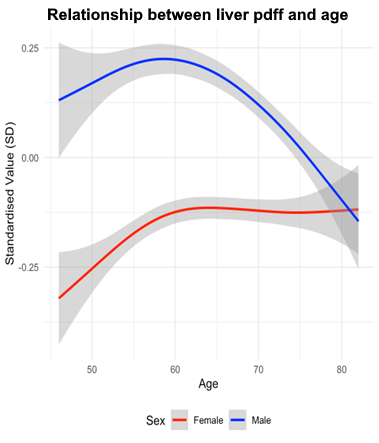

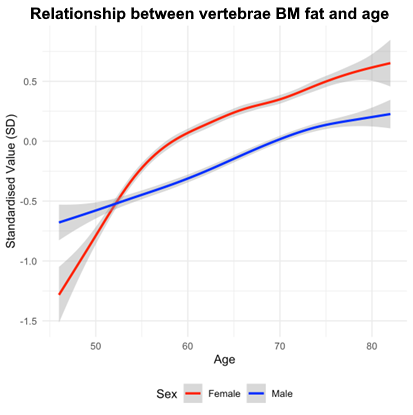


**Supplementary Figure 3. Association between age and adiposity.** The figure explains the variable effect of age on different IDPs in males and females.

| 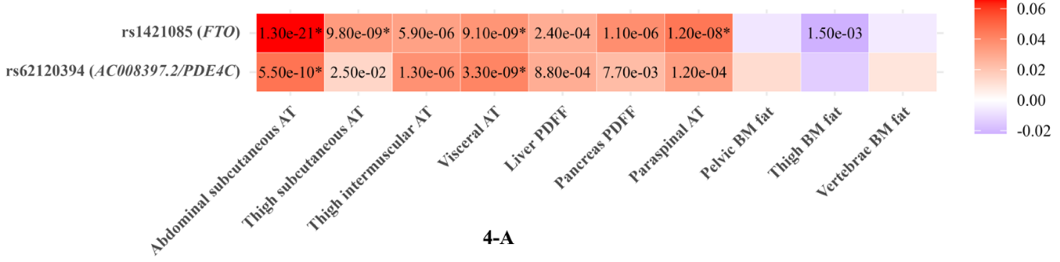 | 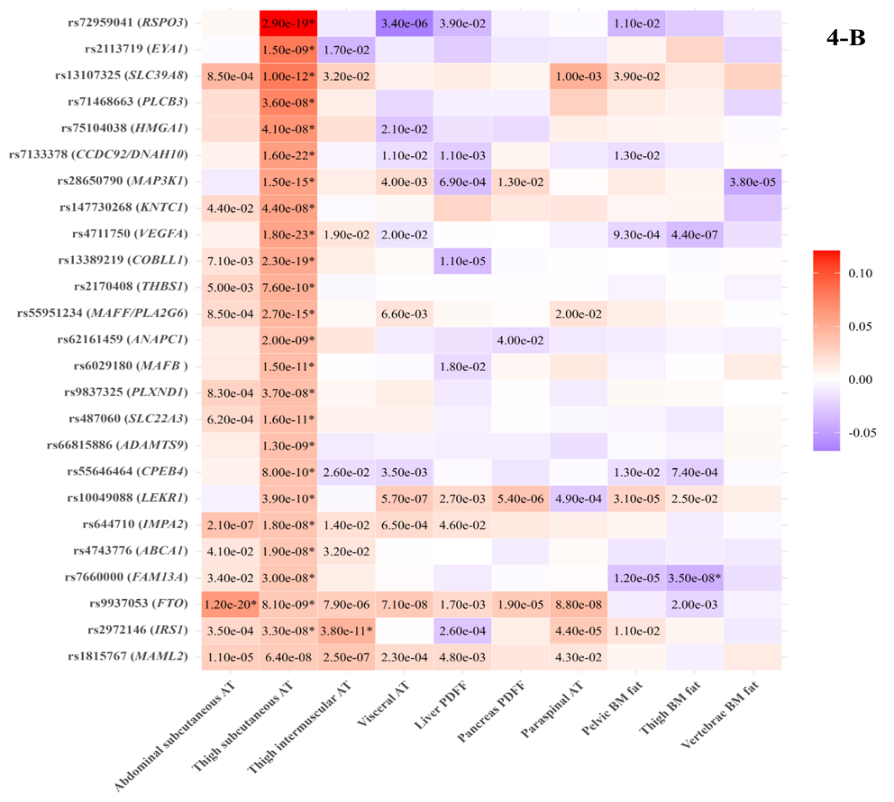 |
| --- | --- |
| 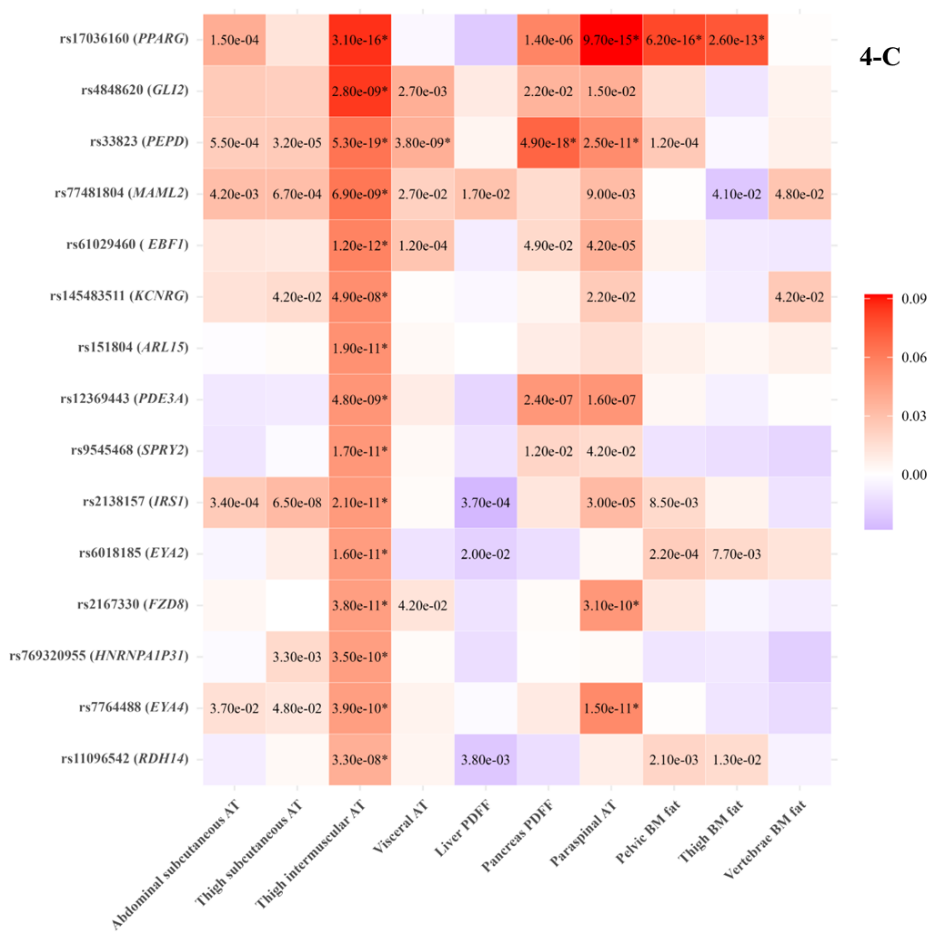 | 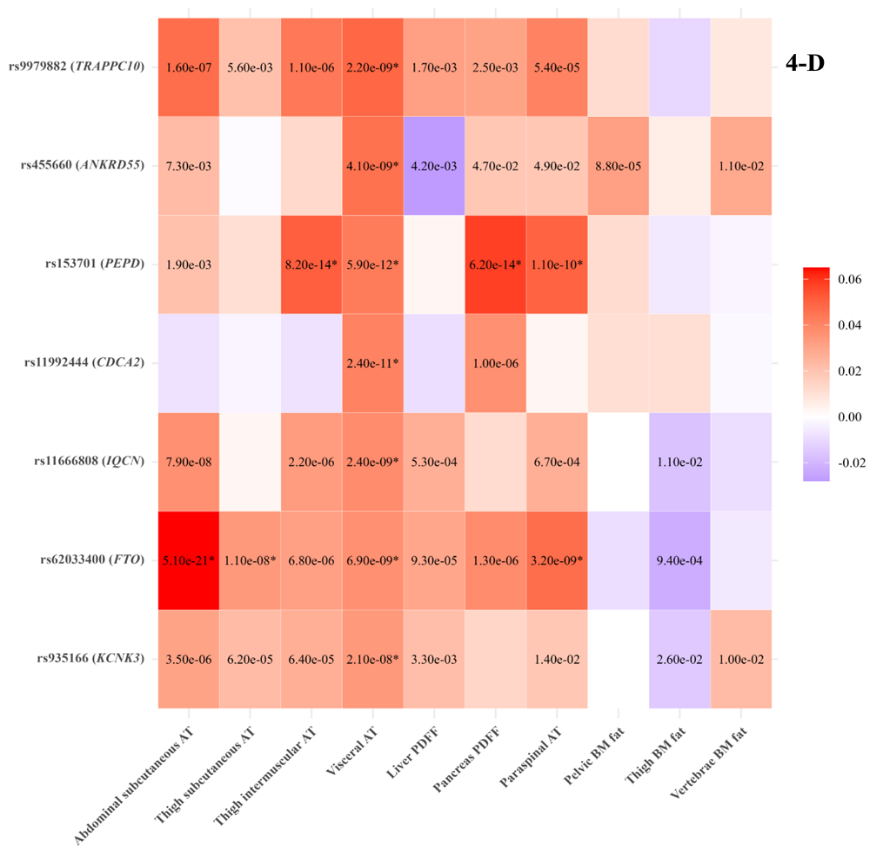 |
| 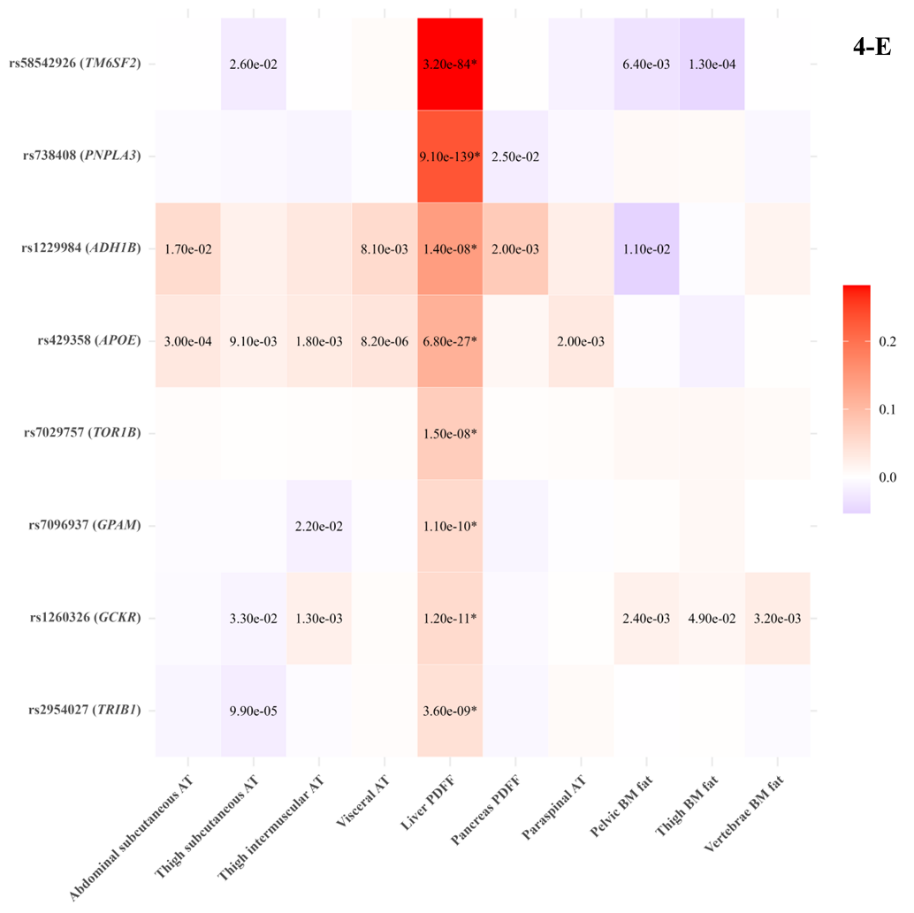 | 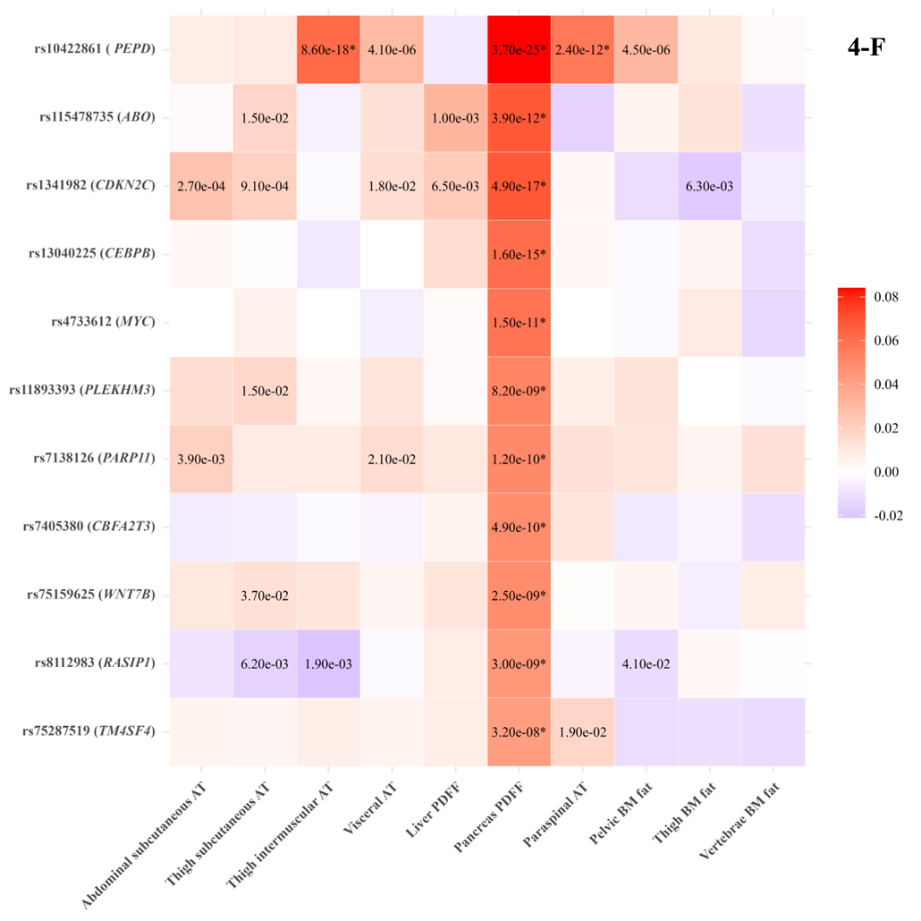 |
| 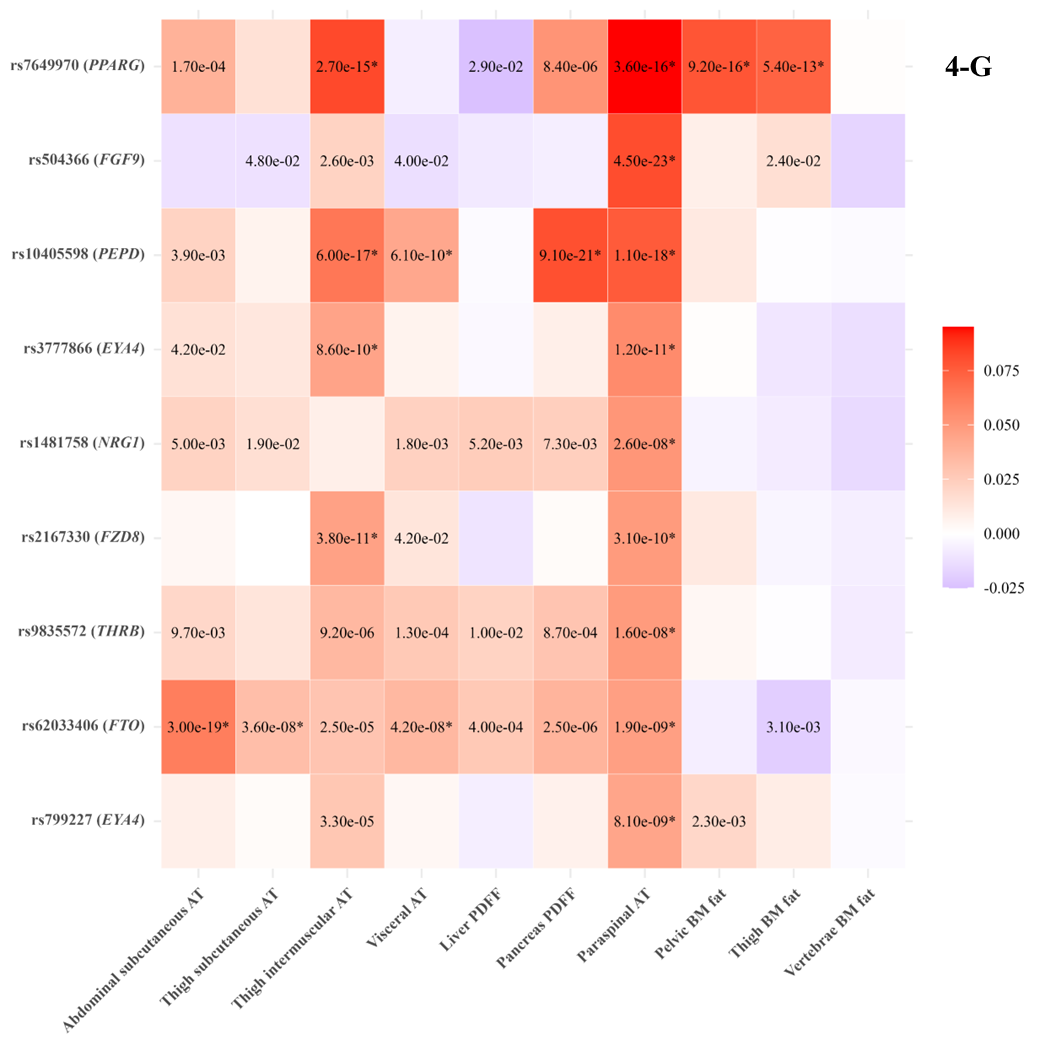 | 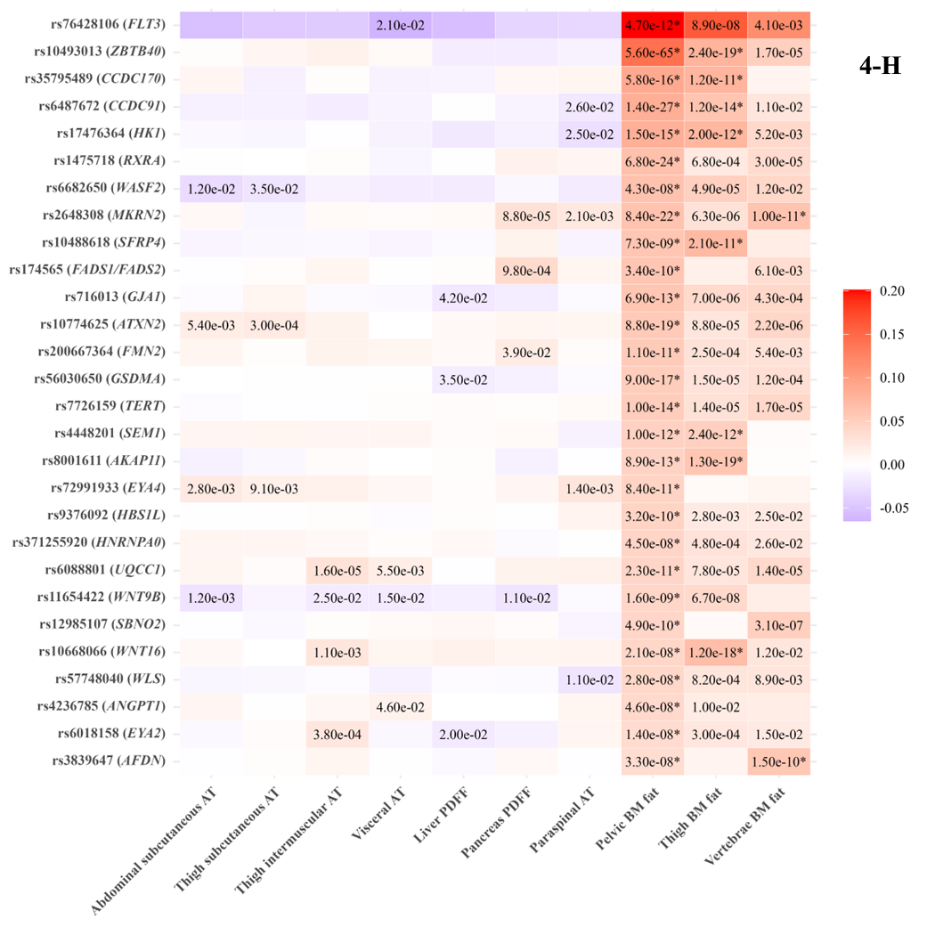 |
| 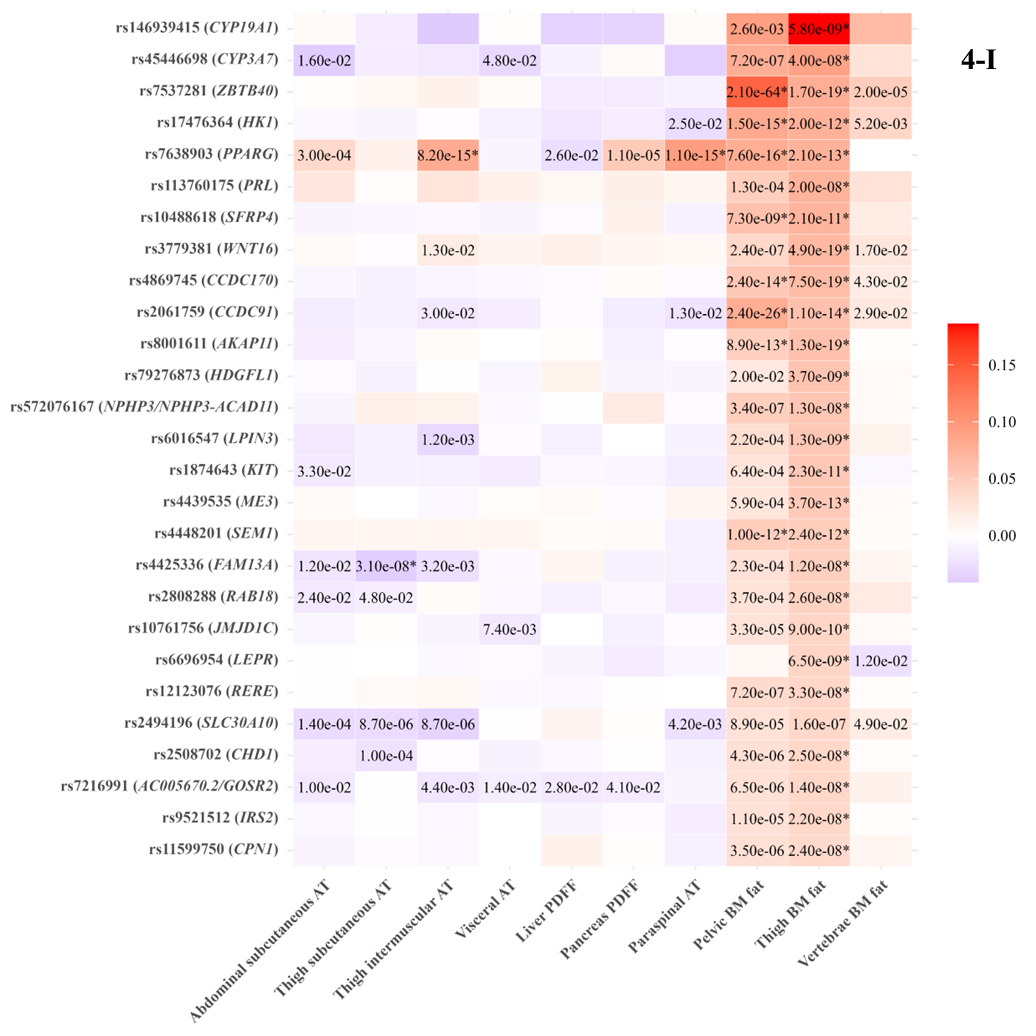 | 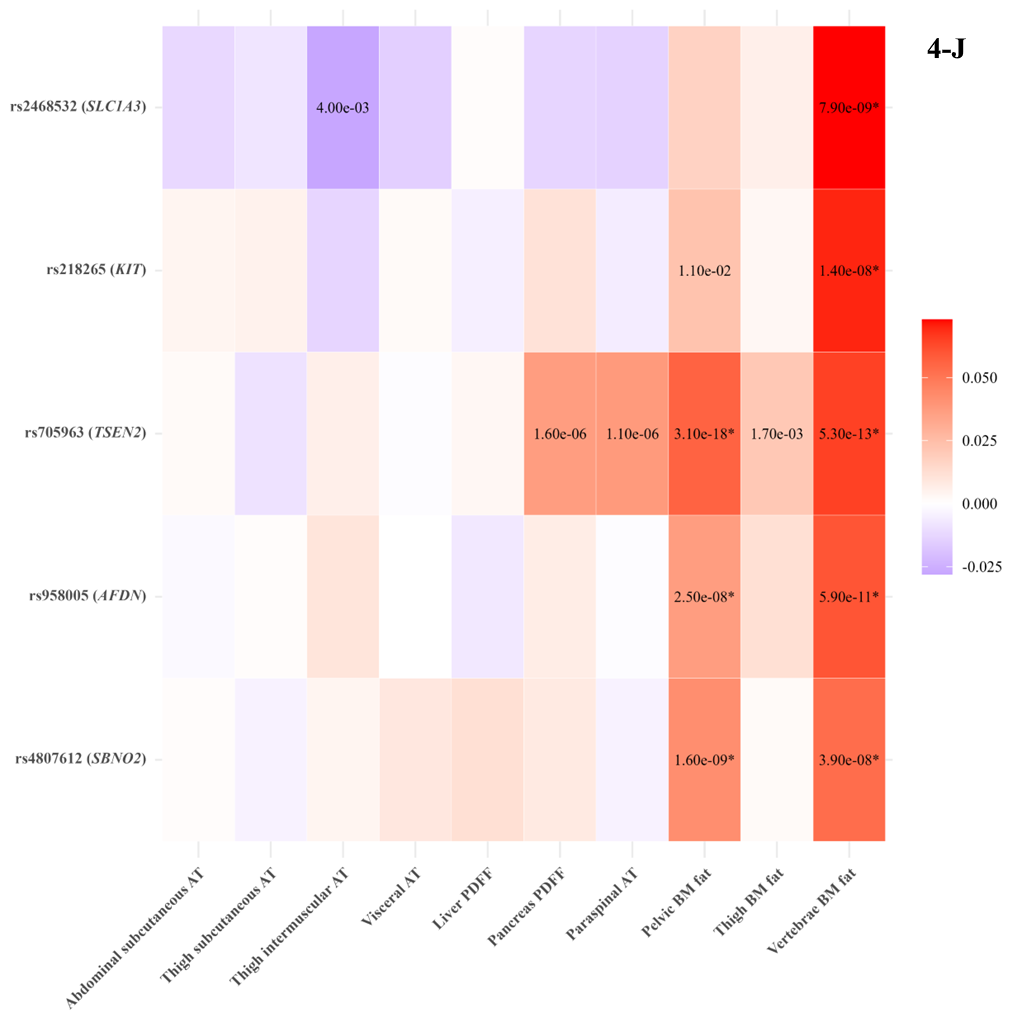 |

**Supplementary Figures 4. The effect of the genetic loci associated with each IDP on other IDPs.**A. abdominal subcutaneous AT; B. thigh subcutaneous AT, C. thigh intermuscular AT, D. visceral AT, E. liver PDFF; F. pancreas PDFF; G. paraspinal AT; H. pelvic bone marrow fat; I. thigh bone marrow fat; J. vertebrae bone marrow fat. The color and intensity of each cell represent the direction and magnitude of the effect, as determined by linear regression in the genome-wide association model. The asterisks indicate associations with a P-value < 5e-08.


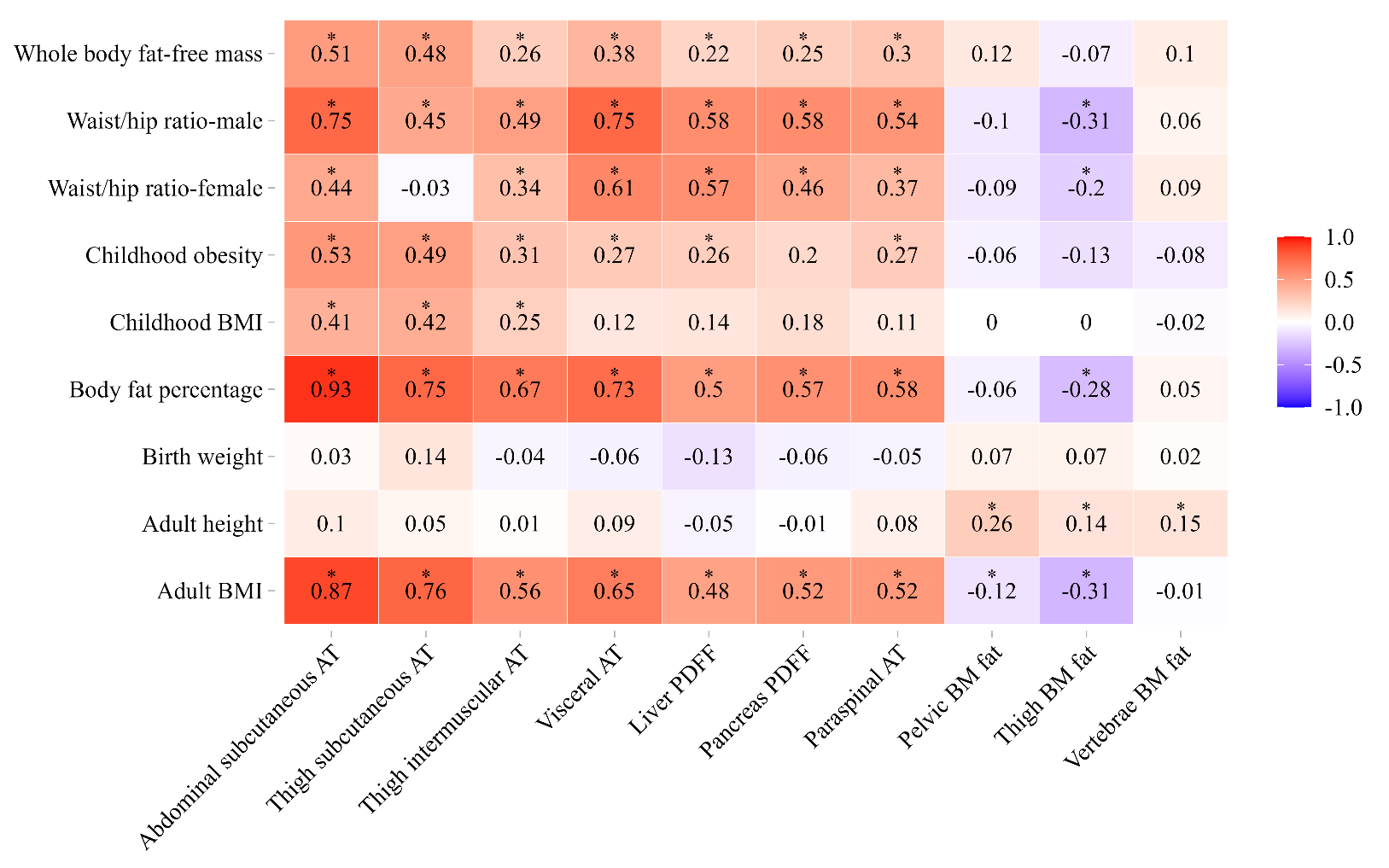


**Supplementary Figure 5. Genetic correlation between IDPs and anthropometric traits.** The color and intensity represent the magnitude and direction of correlation. The labels represent the genetic correlation (rg) and the asterisk represents statistical significance (p< 0.006).

| 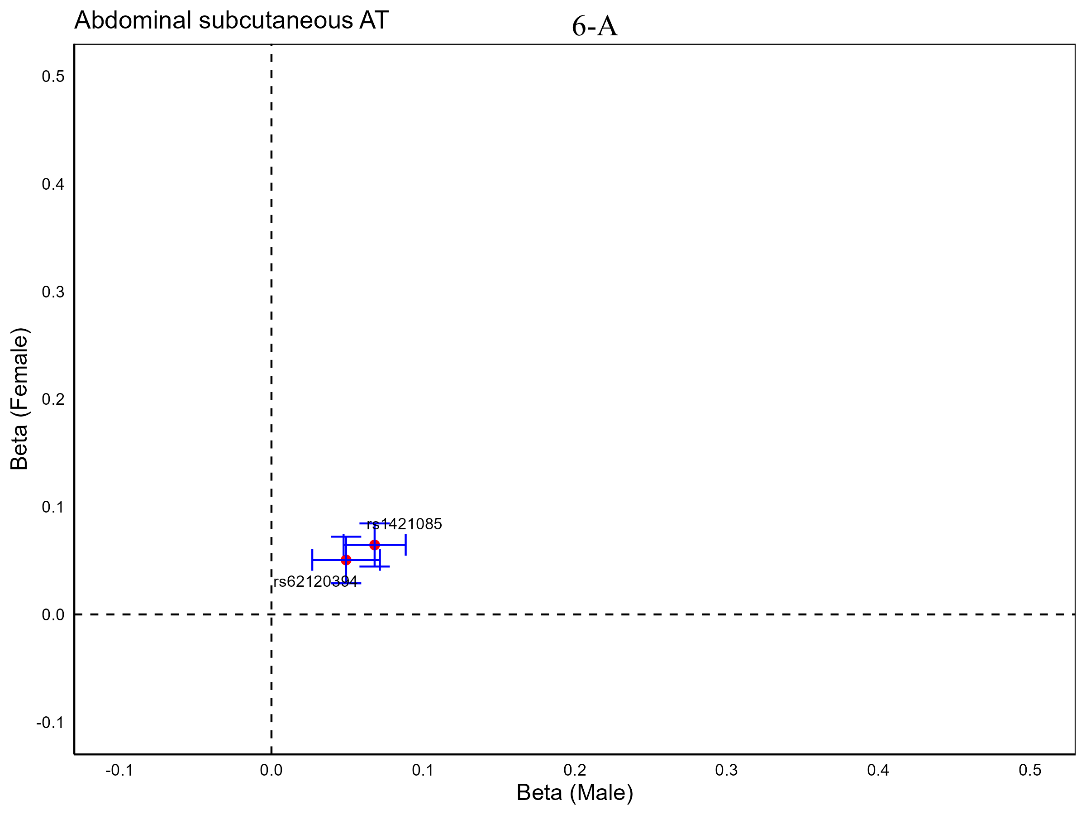 | 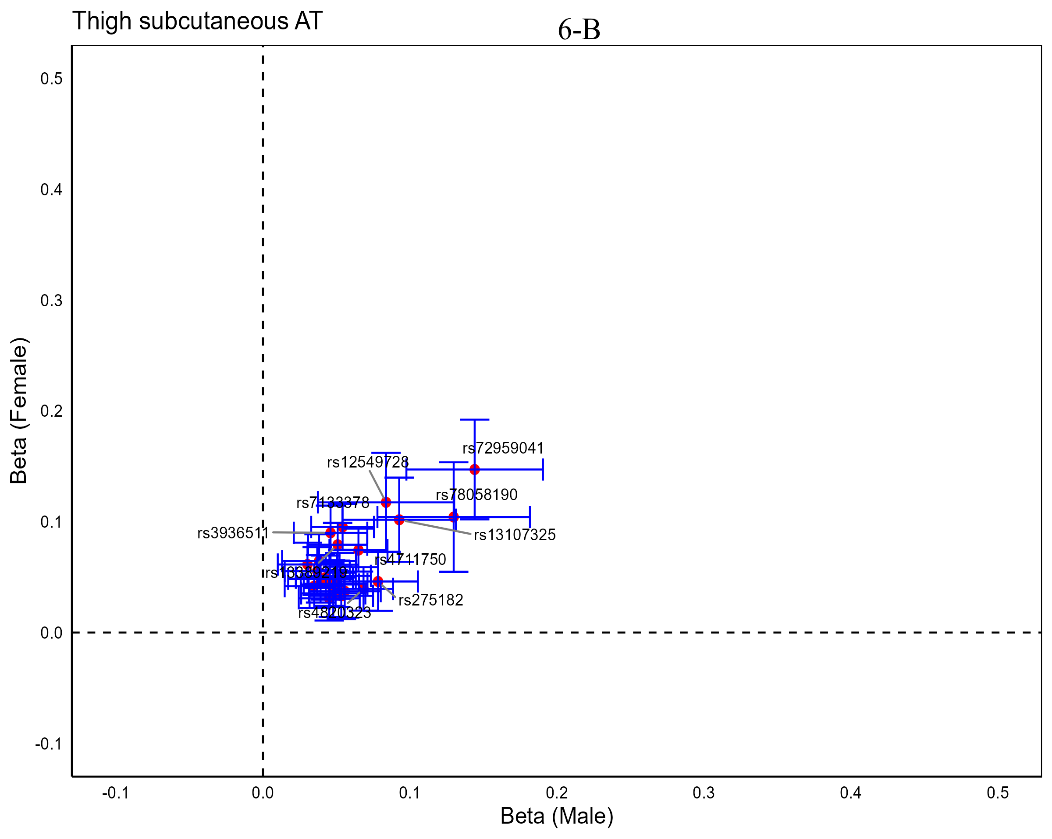 |
| --- | --- |
| 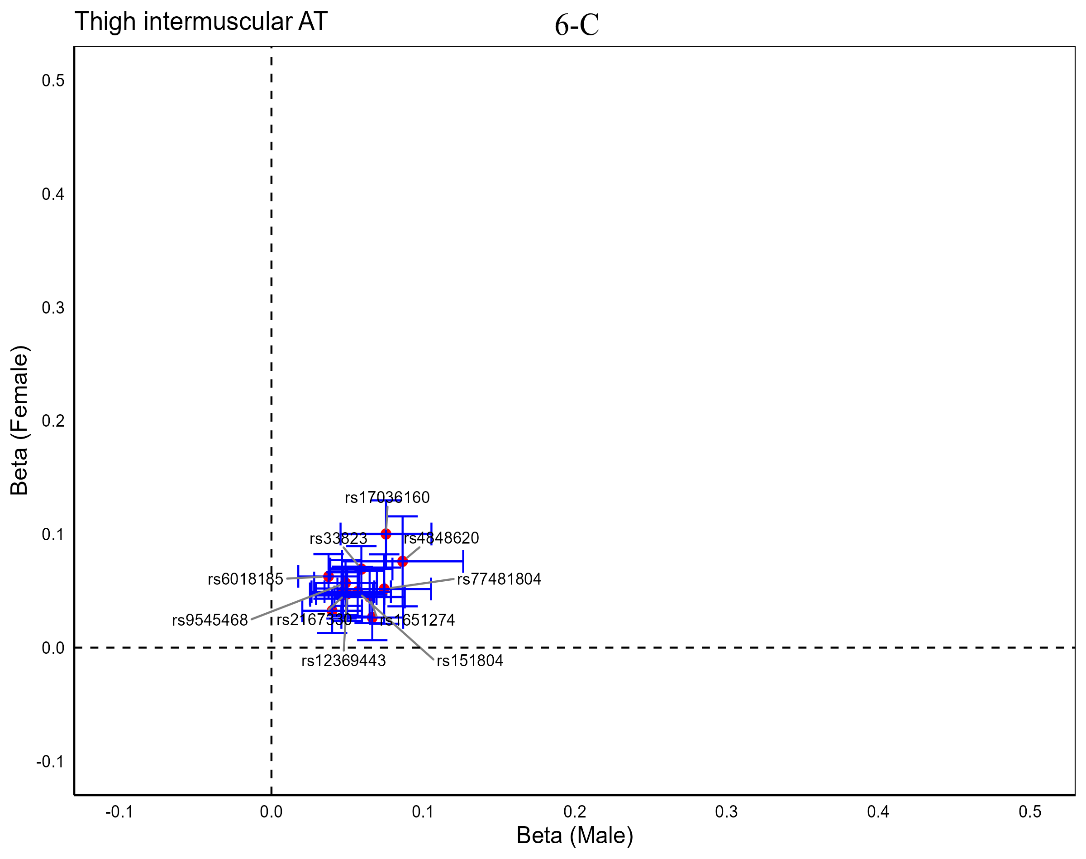 | 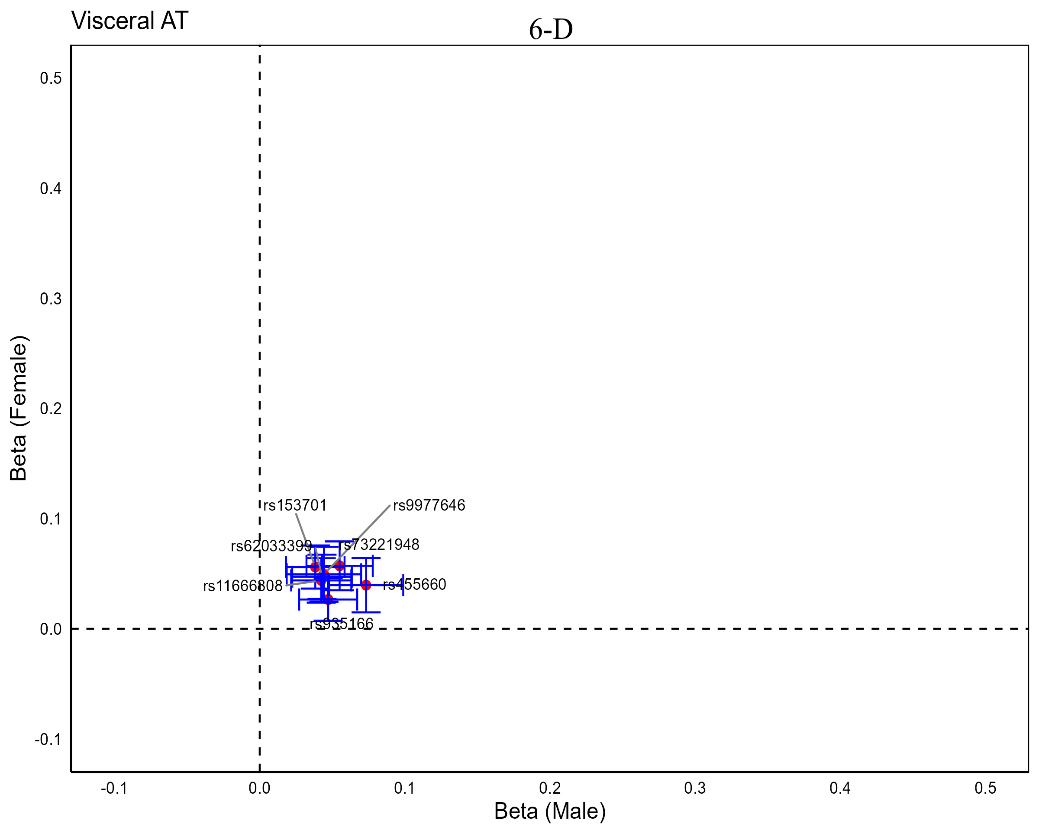 |
| 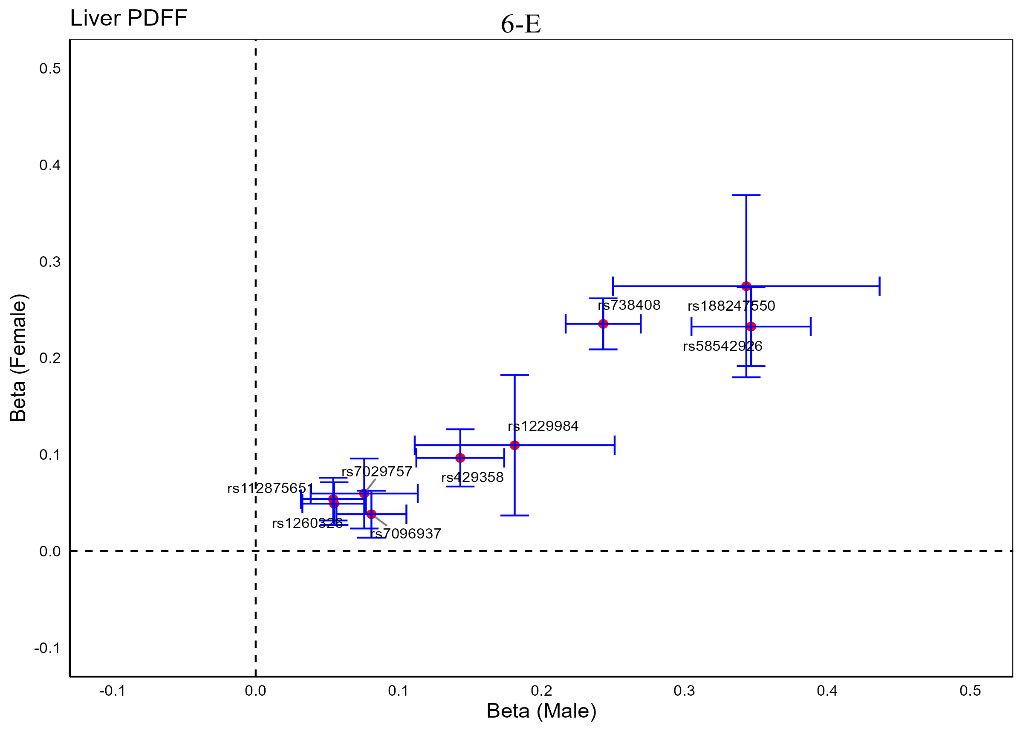 | 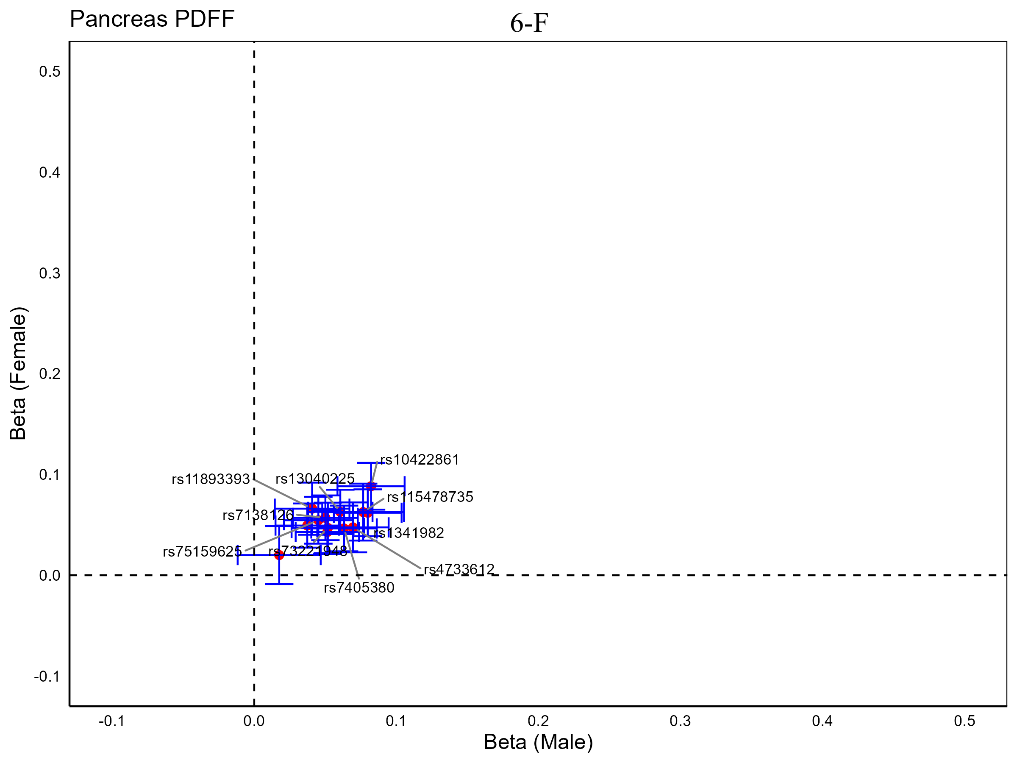 |
| 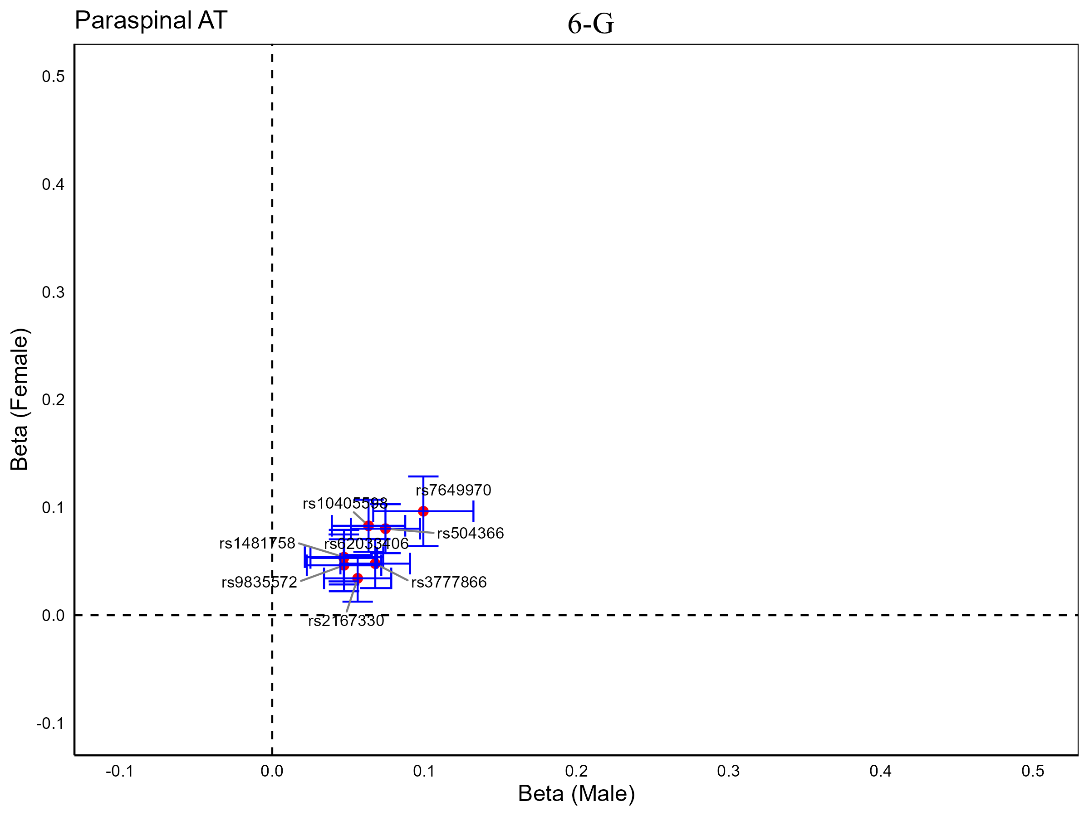 | 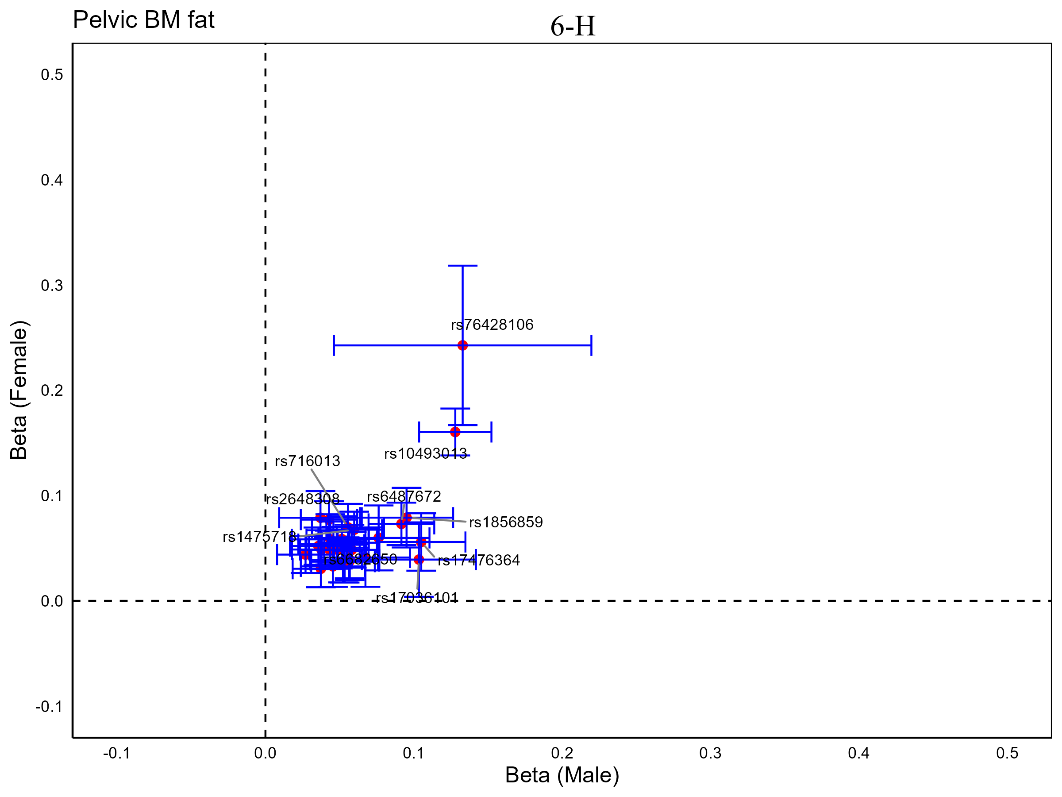 |
| 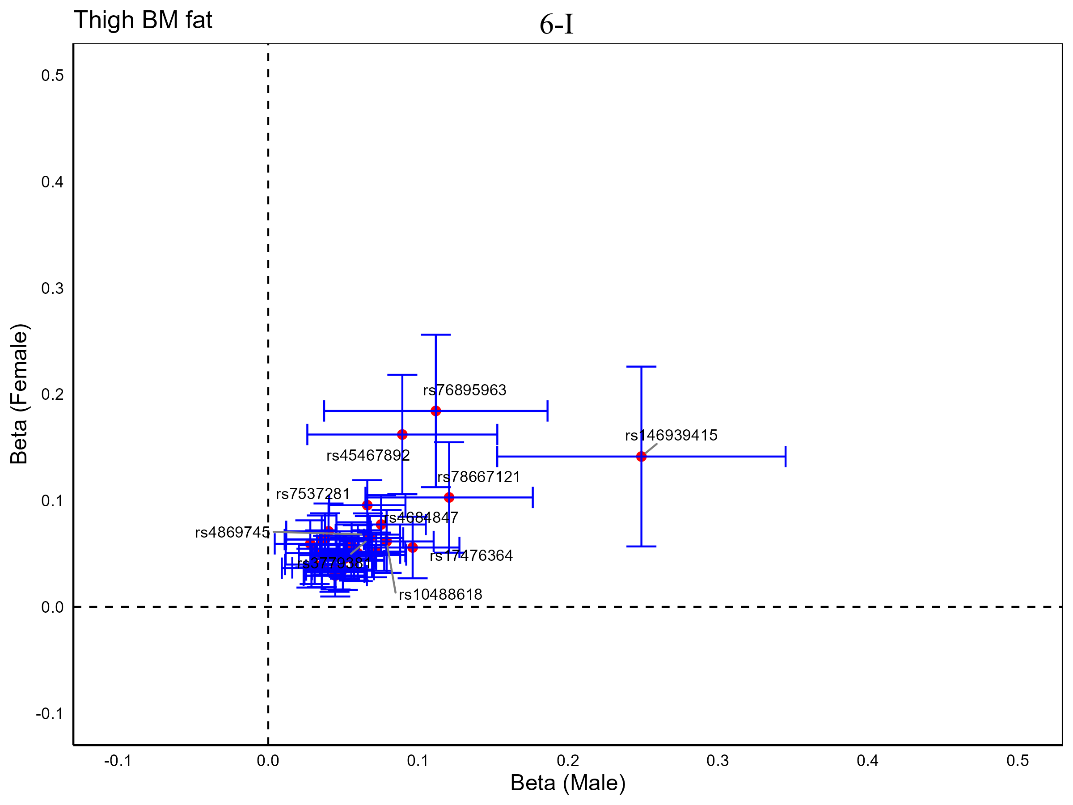 | 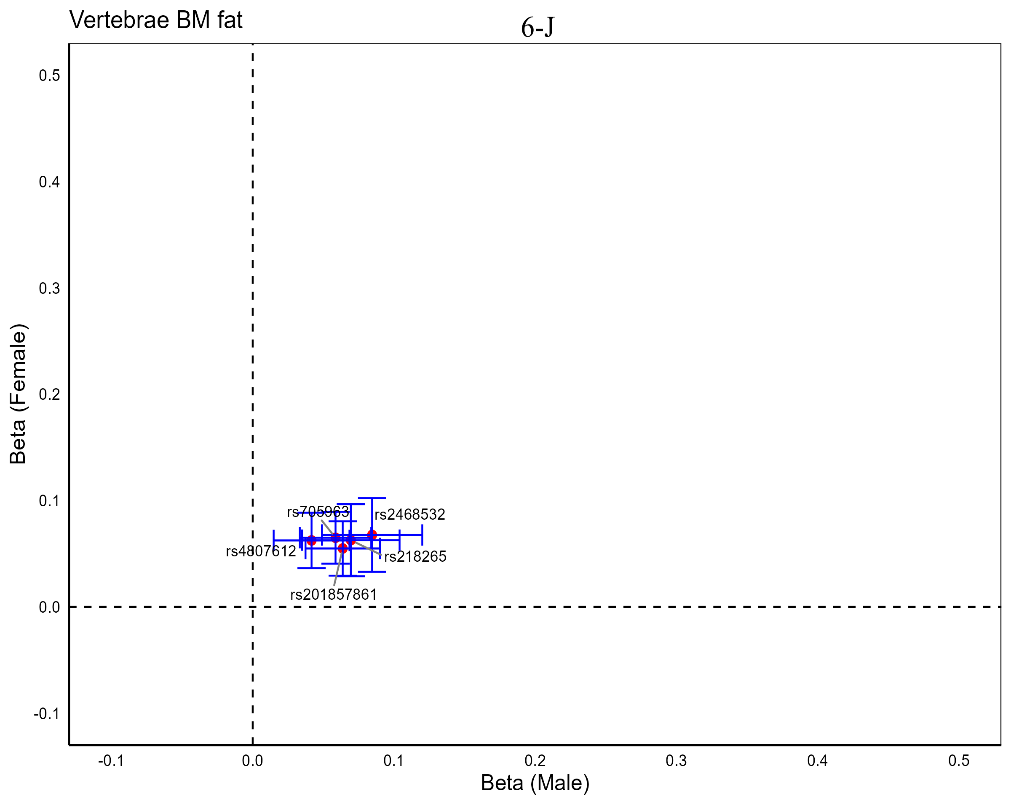 |

**Supplementary Figure 6. The effect of genetic variants associated with the IDPs, stratified by sex.** The X axis represents the effect size (beta) in males and the Y axis represent the effect size in females. The blue lines show the 95% confidence intervals of the effect. A. abdominal subcutaneous AT; B. thigh subcutaneous AT, C. thigh intermuscular AT, D. visceral AT, E. liver PDFF; F. pancreas PDFF; G. paraspinal AT; H. pelvic bone marrow fat; I. thigh bone marrow fat; J. vertebrae bone marrow fat.
